# General Approach to Amides through Decarboxylative Radical Cross-Coupling of Carboxylic Acids and Isocyanides

**DOI:** 10.1021/acs.orglett.4c00872

**Published:** 2024-04-12

**Authors:** Qing Yan, Qing-Jia Yuan, Andrey Shatskiy, Gregory R. Alvey, Elena V. Stepanova, Jian-Quan Liu, Markus D. Kärkäs, Xiang-Shan Wang

**Affiliations:** † School of Chemistry and Materials Science, Jiangsu Key Laboratory of Green Synthesis for Functional Materials, Jiangsu Normal University, Xuzhou, Jiangsu 221116, China; ∥ Department of Chemistry, KTH Royal Institute of Technology, SE-100 44 Stockholm, Sweden; ‡ Research School of Chemistry & Applied Biomedical Sciences, Tomsk Polytechnic University, Lenin Avenue 30, 634050 Tomsk, Russia

## Abstract

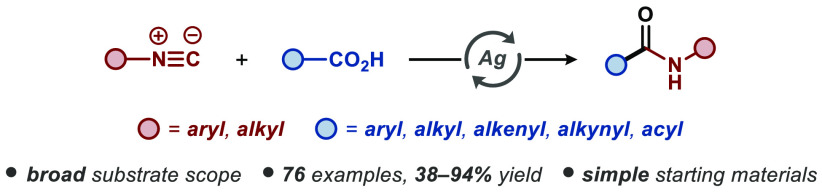

Herein, we report a silver-catalyzed protocol for decarboxylative cross-coupling between carboxylic acids and isocyanides, leading to linear amide products through a free-radical mechanism. The disclosed approach provides a general entry to a variety of decorated amides, accommodating a diverse array of radical precursors, including aryl, heteroaryl, alkynyl, alkenyl, and alkyl carboxylic acids. Notably, the protocol proved to be efficient for decarboxylative late-stage functionalization of several elaborate pharmaceuticals, demonstrating its potential applications.

The amide functionality constitutes a prominent element in nature. In addition to peptides and proteins, amides find applications in a variety of areas—from synthetic polymeric materials, such as nylon or polyacrylamides, to agrochemicals and pharmaceuticals (Figure 1a).^1^ Hence, it is not surprising that the development of new methods for amide bond formation remains a prominent goal in chemical synthesis. A common approach for amide bond formation is the exploitation of (super)stoichiometric quantities of activating agents (Figure 1b) through pre- or in situ activation of the carboxylic acid coupling partner.^2^ Thus, the rather low efficiency and questionable sustainability credentials associated with traditional amide coupling methodologies,^3^ especially at large scale, have stimulated renewed interest in the design of innovative atom efficient and benign catalytic approaches for amide bond formation through the use of nonconventional coupling partners.^4^

**Figure 1:**
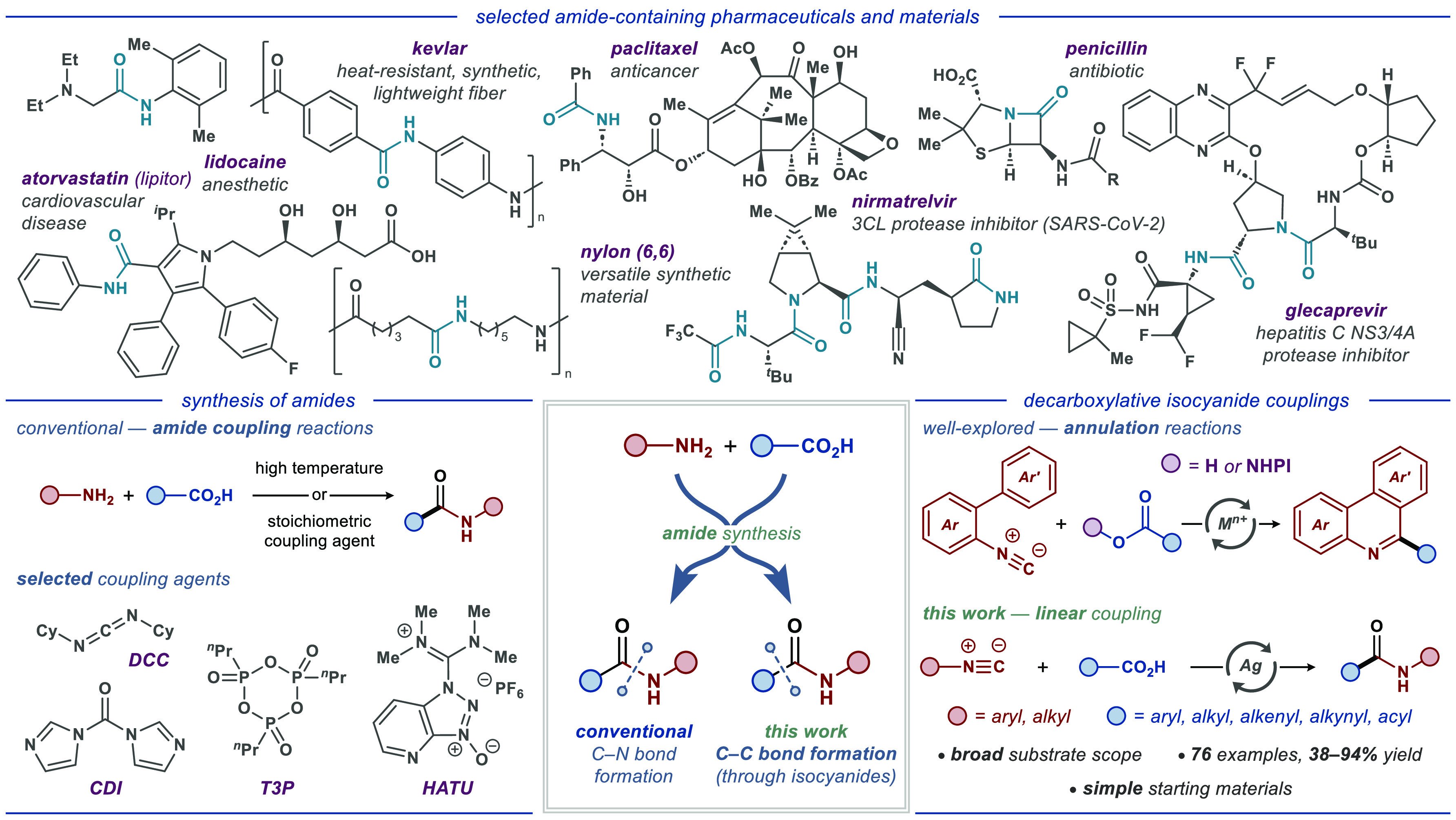
Amides and isocyanides in chemical synthesis and this work.

Isocyanides are widely used and versatile building blocks in chemical synthesis.^5^ They demonstrate carbene-like reactivity, similar to carbon monoxide, making them valuable for various synthetic transformations.^6^ Besides their established role in various polar reaction manifolds, isocyanides have also been extensively explored in radical settings. Specifically, radical cross-coupling reactions involving isocyanides have been predominantly observed as part of tandem cyclization reactions, playing a crucial role in the construction of diverse heterocyclic compounds such as indoles, quinolines, isoquinolines, quinoxalines, and phenanthridines. In contrast, transition-metal-catalyzed radical cross-coupling reactions of isocyanides without subsequent cyclization remain relatively rare.^7^ Consequently, broadening the available scope of direct radical reactions involving isocyanides is highly desirable.

Transition-metal-catalyzed decarboxylative radical cross-coupling reactions serve as a powerful tool for the construction of carbon–carbon and carbon–heteroatom bonds.^8^ However, only a handful of reports on radical cross-coupling reactions of carboxylic acids or their analogues with isocyanides have been disclosed. For example, Grimaud and co-workers reported the radical cross-coupling reaction of diazonium salts with isocyanides and carboxylic acids to generate imides.^9^ However, this reaction is associated with a limited scope and moderate yields of the isolated products (ca. 50%). Subsequently, the Jamison,^10^ Zhou,^11^ Yatham,^12^ and Li^13^ groups reported the oxidative decarboxylative cross-coupling of arylisocyanides with alkyl carboxylic esters or alkyl/aryl carboxylic acids to furnish alkyl/aryl-substituted aromatic aza-heterocycles, respectively (Figure 1c). Meanwhile, the oxidative decarboxylative radical cross-coupling of alkynyl carboxylic acids with isocyanides has been underexploited due to the high energy and short lifetime of the alkynyl radical.^14^ Silver exhibits proficient catalytic activity in radical reactions and isocyanide chemistry.^15^ As part of our interest in developing novel silver-catalyzed reactions involving isocyanides,^16^ herein we report a silver-catalyzed protocol for decarboxylative radical cross-coupling of various carboxylic acids with isocyanides, allowing general entry to decorated amides (Figure 1c).

In designing a general platform for accessing amides, we surmised that decarboxylative cross-coupling would serve as a suitable entry. Carboxylic acids are ubiquitous and can be exploited as versatile sources of radicals, making them competent and easily accessible cross-coupling partners. We envisioned that a metal catalyst could mediate decarboxylation to generate the desired carbon-centered radical. Then, this radical could engage with the isocyanide to furnish a radical that can be intercepted with a suitable oxygen-donating agent, ultimately producing the coveted amide.

The execution of our design commenced using 4-bromophenylisocyanide (**1a**) and cyclohexanecarboxylic acid (**2a**) as the model substrates to screen the reaction conditions (for a detailed discussion, see the Supporting Information). With a suitable set of reaction conditions established, the versatility of the developed protocol was explored (Scheme 1). A series of aromatic isocyanides engaged in the reaction with cyclohexanecarboxylic acid **2a** to deliver the corresponding amide products **3b**–**3u** in 64–89% yields. Among these, *ortho*-, *meta*-, and *para*-substituted aromatic isocyanides bearing either electron-donating (e.g., Me, MeO, and EtO) or electron-withdrawing (e.g., F, Cl, Br, and CF_3_) substituents could be efficiently converted to the desired products in high yields. A range of diversely functionalized aliphatic carboxylic acids were also effective in decarboxylative coupling with 4-bromophenylisocyanide **1a**, furnishing expected products **3v**–**3ag** in moderate to high yields (58–94%). Delightfully, a wide array of aryl and heteroaryl carboxylic acids were well-tolerated, allowing efficient access to corresponding amides **3ah**–**3as**. Additionally, both aliphatic and heteroaromatic isocyanides **1v**–**1aa** could engage in the reaction to provide the corresponding amides **3at**–**3ay** (66–81%). Similarly, cinnamic and α-oxocarboxylic acids furnished the expected products **3az**–**3be** with a high efficiency. Importantly, we found that alkynyl carboxylic acids were also compatible with the disclosed catalytic system, albeit with lower yields, providing a prominent entry to alkyne-based radical transformations. Unfortunately, conducting the reaction with methylenated isocyanides, including ethyl isocyanoacetate and TosMIC, only provided the imidazole products, which is in agreement with the results previously reported in the literature.^17^

**Scheme 1:**
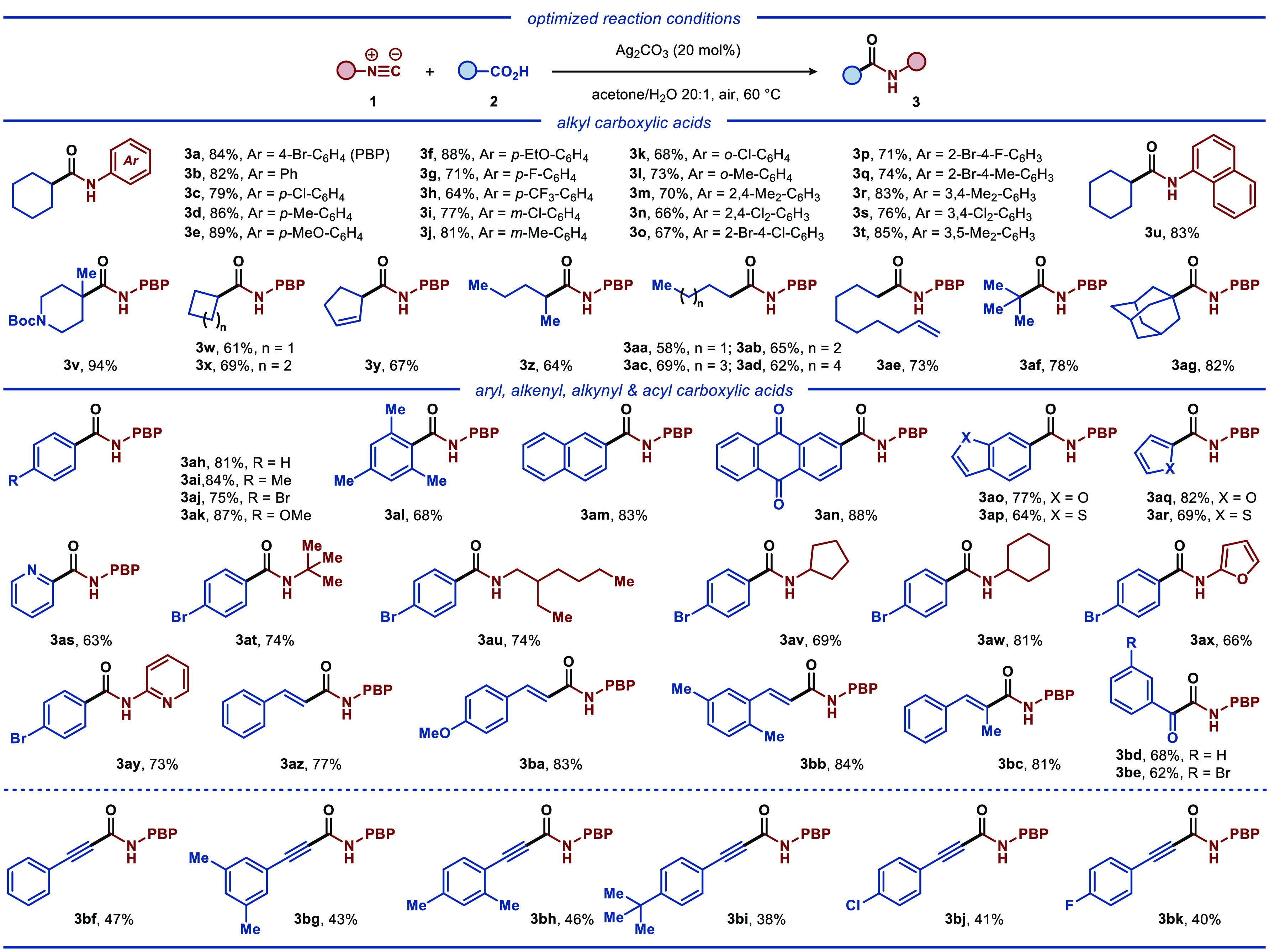
Scope of Carboxylic Acids and Isocyanides Reaction conditions: isocyanide **1** (0.5 mmol, 1.0 equiv), carboxylic acid **2** (1 mmol, 2.0 equiv), Ag_2_CO_3_ (0.10 mmol, 0.2 equiv), acetone (5.0 mL), H_2_O (250 μL), 60 °C, 8 h. All yields are of isolated products. PBP = 4-bromo-C_6_H_4_.

To demonstrate the applicability of the disclosed protocol for late-stage functionalization of bioactive molecules (Scheme 2), a range of pharmaceuticals, including abietic acid, ibuprofen, fenofibric acid, and telmisartan, were subjected to the optimized reaction conditions, smoothly furnishing the desired product **3bl**–**3bs** in 56–83% yields. Employing *N*-protected amino acids as substrates also furnished the desired products (**3bt**–**3bx**) in good yield, demonstrating the robustness of the disclosed protocol. Additionally, we obtained the single crystal X-ray structure of product **3bn** (CCDC no. 2291774), unequivocally confirming the identity of the product.

**Scheme 2:**
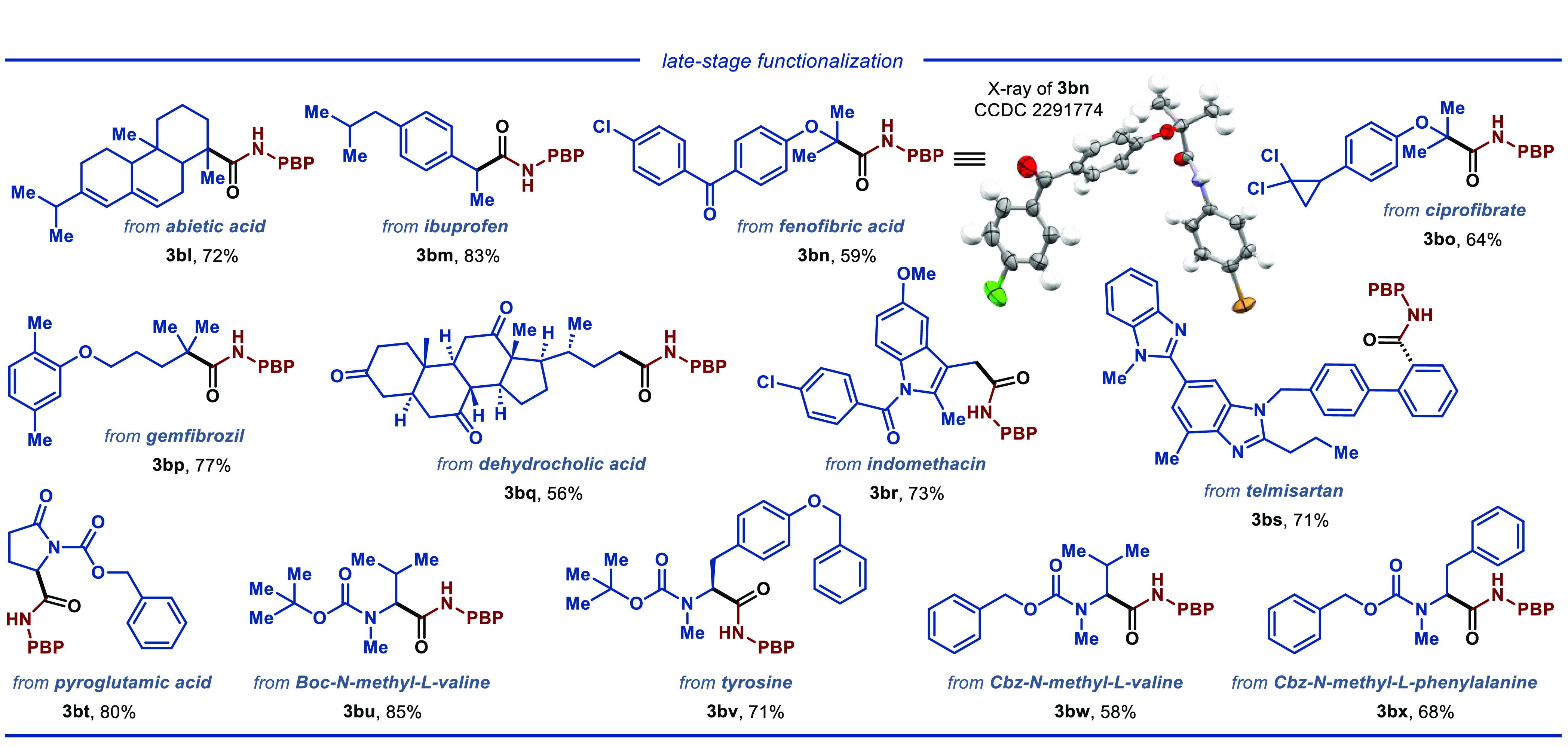
Late-Stage Diversification of Pharmaceuticals, Natural Products, and Biomolecules

For a better understanding of the reaction mechanism, a series of mechanistic experiments were carried out (Scheme 3). First, reacting isocyanide **1ab** with benzoic acid **2n** or phenylpropiolic acid **2af** under optimal conditions furnished the corresponding tandem cyclized products, highlighting that the reaction proceeds through a free-radical process. Next, cyclohexanecarboxylic (**2a**), benzoic (**2n**), and phenylpropiolic acids (**2af**) were subjected to the optimized reaction conditions with 4-bromophenylisocyanide (**1a**) and 2 equiv of TEMPO as a radical scavenger. For all three reactions, formation of the amide product was completely inhibited, indicating that the reaction proceeds through a free-radical pathway. Additionally, TEMPO-based adduct **5** was isolated in 48% yield for reaction with benzoic acid. Further, control experiments with isotopically labeled benzoic acid ([^13^C]-**2n**) resulted in unlabeled product **3ah**, demonstrating that the carbonyl carbon in the product derives from isocyanide.^18^ Conducting the reaction under the atmosphere of ^18^O-labeled dioxygen resulted in [^18^O]-**3ah** in 78% yield with a high degree of ^18^O-incorporation, while excluding oxygen inhibited the reaction (12% yield). Finally, the reaction between **2n** and **1a** in the presence of H_2_^18^O did not yield any labeled product [^18^O]-**3ah**. Importantly, the latter suggests that in the disclosed reaction, water does not act as an oxygen source, in contrast to previously disclosed isocyanide-coupling reactions.^19^

**Scheme 3:**
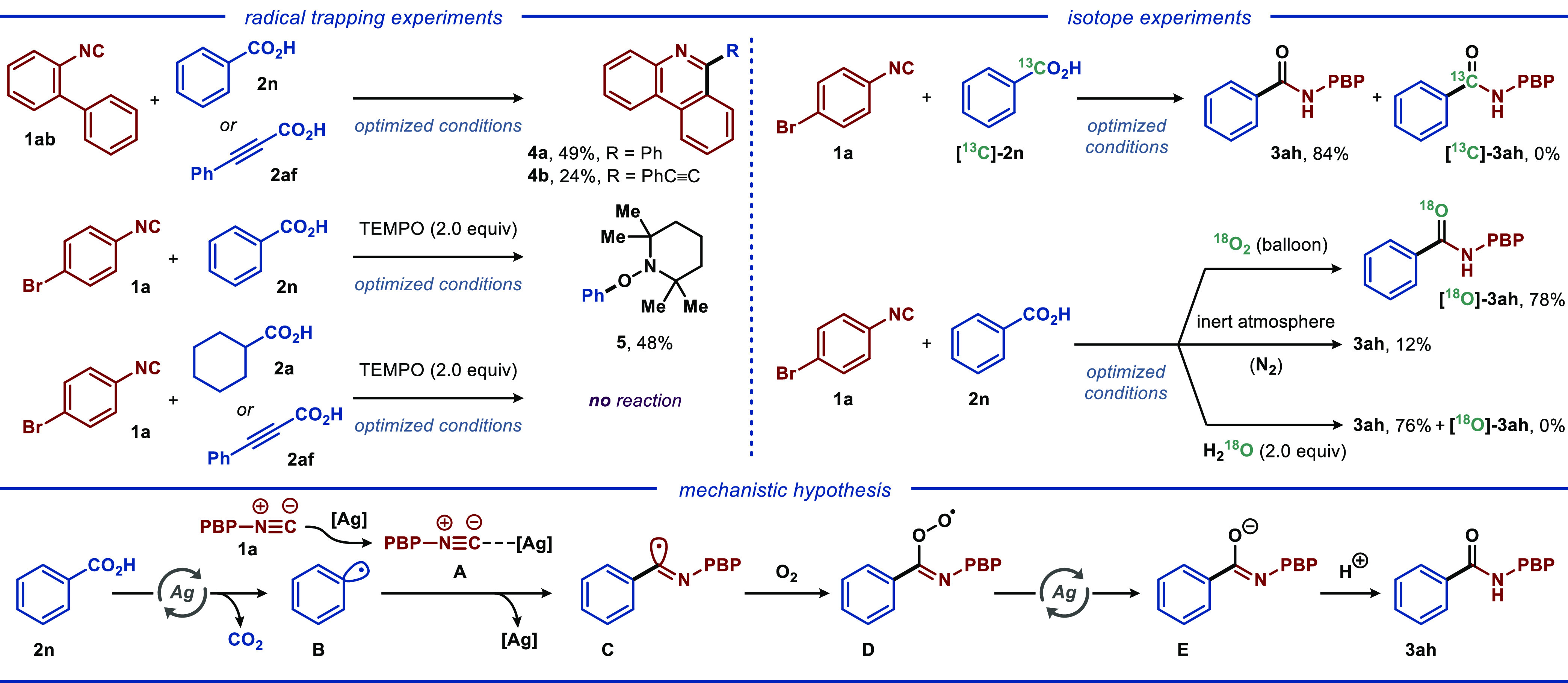
Mechanistic Considerations

Based on the above mechanistic experiments and relevant literature precedents,^6b,6c,15^ a plausible mechanism for the disclosed reaction is proposed with **1a** and **2n** as the model substrates. First, isocyanide **1a** coordinates to the silver catalyst to produce silver intermediate **A**. At the same time, decarboxylation of benzoic acid to phenyl radical **B** is mediated by a silver catalyst, as has been described for other transformations.^20^ Subsequently, phenyl radical **B** undergoes coupling with complex **A** (or free isocyanide) to produce radical imine adduct **C**, which is oxidized by O_2_ to produce peroxide intermediate **D**. This species presumably undergoes rapid silver-mediated interconversion to intermediate **E**. Finally, the protonation of intermediate **E** furnishes the desired product **3ah**. In this sequence, the silver catalyst mediates the decarboxylation step either through a Ag^II^/Ag^I^ catalytic cycle^21^ with oxygen as the terminal oxidant^22^ or enables this step through an inner-sphere Ag^I^-promoted pathway.^23^

In conclusion, an appealing approach for free-radical coupling between carboxylic acids and isocyanides to yield a diverse collection of amides was realized through silver catalysis. The disclosed protocol displays good functional group tolerance, delivering the corresponding amide products in excellent yields for a majority of the evaluated substrates. The developed methodology also expands the currently known scope of use of isocyanides in free-radical chemistry, aiding the development of new catalytic systems.

## Data Availability

The data underlying this study are available in the published article and its online Supporting Information.

## Supporting Information Available

Experimental procedures, X-ray crystallography data of **3bn** as well as characterization data, and copies of NMR spectra for all obtained products (PDF)
FAIR data, including the primary NMR FID files, for compounds **3**, **4**, and **5** (ZIP)
