# Supplementary material for: General Approach to Amides through Decarboxylative Radical Cross-Coupling of Carboxylic Acids and Isocyanides
Source: Org Lett. 2024 Apr 12;26(16):3380–5. doi: 10.1021/acs.orglett.4c00872 (PMC11059110; doi:10.1021/acs.orglett.4c00872)
Supplement: Supplementary file 1 — ol4c00872_si_001.pdf [file ol4c00872_si_001.pdf]

Supporting Information

General Approach to Amides through Decarboxylative Radical Cross-Coupling of Carboxylic Acids and Isocyanides

Qing Yan,[†] Qing-Jia Yuan,[†] Andrey Shatskiy,[‖] Gregory R. Alvey,[‖] Elena V. Stepanova,[‖,‡] Jian-Quan Liu,[†]* Markus D. Kärkäs,[‖]* Xiang-Shan Wang[†]*

† School of Chemistry and Materials Science, Jiangsu Normal University, Xuzhou, Jiangsu 221116, China

‖ Department of Chemistry, KTH Royal Institute of Technology, SE-100 44 Stockholm, Sweden

‡ Research School of Chemistry & Applied Biomedical Sciences, Tomsk Polytechnic University, Lenin Avenue 30, 634050 Tomsk, Russia

* E-mail: liujq316@jsnu.edu.cn (J.-Q.L.); karkas@kth.se (M.D.K.); xswang@jsnu.edu.cn (X.-S.W)

Table of Contents

I. General information **S1**

II. Crystallography **S1**

III. Synthesis and analytical data of compounds 1 and 3 .... **S3**

General procedure for synthesis of isocyanides **1** S3

Reaction optimization S4

N-(4-Bromophenyl)cyclohexanecarboxamide (**3a**) S6

1 mmol scale synthesis of *N*-(4-bromophenyl)cyclohexanecarboxamide (**3a**) S6

N-Phenylcyclohexanecarboxamide (**3b**) S7

N-(4-Chlorophenyl)cyclohexanecarboxamide (**3c**) S7

N-(*p*-Tolyl)cyclohexanecarboxamide (**3d**) S8

N-(4-Methoxyphenyl)cyclohexanecarboxamide (**3e**) S8

N-(4-Ethoxyphenyl)cyclohexanecarboxamide (**3f**) S9

N-(4-Fluorophenyl)cyclohexanecarboxamide (**3g**) S10

N-(4-(Trifluoromethyl)phenyl)cyclohexanecarboxamide (**3h**) S10

N-(3-Chlorophenyl)cyclohexanecarboxamide (**3i**) S11

N-(*m*-Tolyl)cyclohexanecarboxamide (**3j**) S12

N-(2-Chlorophenyl)cyclohexanecarboxamide (**3k**) S12

N-(*o*-Tolyl)cyclohexanecarboxamide (**3l**) S13

N-(2,4-Dimethylphenyl)cyclohexanecarboxamide (**3m**) S14

N-(2,4-Dichlorophenyl)cyclohexanecarboxamide (**3n**) S14

N-(2-Bromo-4-chlorophenyl)cyclohexanecarboxamide (**3o**) S15

N-(2-Bromo-4-fluorophenyl)cyclohexanecarboxamide (**3p**) S15

N-(2-Bromo-4-methylphenyl)cyclohexanecarboxamide (**3q**) S16

N-(3,4-Dimethylphenyl)cyclohexanecarboxamide (**3r**) S17

N-(3,4-Dichlorophenyl)cyclohexanecarboxamide (**3s**) S17

N-(3,5-Dimethylphenyl)cyclohexanecarboxamide (**3t**) S18

N-(Naphthalen-1-yl)cyclohexanecarboxamide (**3u**) S18

tert-Butyl 4-((4-bromophenyl)carbamoyl)-4-methylpiperidine-1-carboxylate (**3v**) S19

N-(4-Bromophenyl)cyclobutanecarboxamide (**3w**) S20

N-(4-Bromophenyl)cyclopentanecarboxamide (**3x**) ... S20

N-(4-Bromophenyl)cyclopent-1-ene-1-carboxamide (**3y**) ... S21

N-(4-Bromophenyl)-2-methylpentanamide (**3z**) ... S21

N-(4-Bromophenyl)pentanamide (**3aa**) ... S22

N-(4-Bromophenyl)hexanamide (**3ab**) ... S23

N-(4-Bromophenyl)heptanamide (**3ac**) ... S23

N-(4-Bromophenyl)octanamide (**3ad**) ... S24

N-(4-Bromophenyl)dec-9-enamide (**3ae**) ... S24

N-(4-Bromophenyl)pivalamide (**3af**) ... S25

(1*r*,3*r*,5*r*,7*r*)-*N*-(4-Bromophenyl)adamantane-2-carboxamide (**3ag**) ... S25

N-(4-Bromophenyl)benzamide (**3ah**) ... S26

N-(4-Bromophenyl)-4-methylbenzamide (**3ai**) ... S27

4-Bromo-*N*-(4-bromophenyl)benzamide (**3aj**) ... S27

N-(4-Bromophenyl)-4-methoxybenzamide (**3ak**) ... S28

N-(4-Bromophenyl)-2,4,6-trimethylbenzamide (**3al**) ... S28

N-(4-Bromophenyl)-2-naphthamide (**3am**) ... S29

N-(4-Bromophenyl)-9,10-dioxo-8a,9,10,10a-tetrahydroanthracene-2-carboxamide (**3an**) ... S29

N-(4-Bromophenyl)benzofuran-6-carboxamide (**3ao**) ... S30

N-(4-Bromophenyl)benzo[b]thiophene-5-carboxamide (**3ap**) ... S31

N-(4-Bromophenyl)furan-2-carboxamide (**3aq**) ... S31

N-(4-Bromophenyl)thiophene-2-carboxamide (**3ar**) ... S32

N-(4-Bromophenyl)picolinamide (**3as**) ... S32

4-Bromo-*N*-(*tert*-butyl)benzamide (**3at**) ... S33

4-Bromo-*N*-(2-ethylhexyl)benzamide (**3au**) ... S33

4-Bromo-*N*-cyclopentylbenzamide (**3av**) ... S34

4-Bromo-*N*-cyclohexylbenzamide (**3aw**) ... S35

4-Bromo-*N*-(furan-2-yl)benzamide (**3ax**) ... S35

4-Bromo-*N*-(pyridin-2-yl)benzamide (**3ay**) ... S36

N-(4-Bromophenyl)cinnamamide (**3az**) ... S36

(*E*)-*N*-(4-Bromophenyl)-3-(4-methoxyphenyl)acrylamide (**3ba**) ... S37

(*E*)-*N*-(4-Bromophenyl)-3-(2,5-dimethylphenyl)acrylamide (**3bb**) S37

(*E*)-*N*-(4-Bromophenyl)-2-methyl-3-phenylacrylamide (**3bc**) S38

N-(4-Bromophenyl)-2-oxo-2-phenylacetamide (**3bd**) S39

2-(3-Bromophenyl)-*N*-(4-bromophenyl)-2-oxoacetamide (**3be**) S39

N-(4-Bromophenyl)-3-phenylpropiolamide (**3bf**) S40

N-(4-Bromophenyl)-3-(3,5-dimethylphenyl)propiolamide (**3bg**) S40

N-(4-Bromophenyl)-3-(2,4-dimethylphenyl)propiolamide (**3bh**) S41

N-(4-Bromophenyl)-3-(4-(*tert*-butyl)phenyl)propiolamide (**3bi**) S42

N-(4-Bromophenyl)-3-(4-chlorophenyl)propiolamide (**3bj**) S42

N-(4-Bromophenyl)-3-(4-fluorophenyl)propiolamide (**3bk**) S43

N-(4-Bromophenyl)-7-isopropyl-1,4a-dimethyl-1,2,3,4,4a,4b,5,6,10,10a-decahydrophenanthrene-1-carboxamide (**3bl**) S44

N-(4-Bromophenyl)-2-(4-isobutylphenyl)propanamide (**3bm**) S44

N-(4-Bromophenyl)-2-(4-(4-chlorobenzoyl)phenoxy)-2-methylpropanamide (**3bn**) S45

N-(4-Bromophenyl)-2-(4-(2,2-dichlorocyclopropyl)phenoxy)-2-methylpropanamide (**3bo**) S45

N-(4-Bromophenyl)-5-(2,5-dimethylphenoxy)-2,2-dimethylpentanamide (**3bp**) S46

(*R)-N*-(4-Bromophenyl)-4-((5*R*,8*R*,9*S*,10*S*,13*R*,14*S*,17*R*)-10,13-dimethyl-3,7,12-trioxohexadecahydro-1*H*-cyclopenta[*a*]phenanthren-17-yl)pentanamide (**3bq**) S47

N-(4-Bromophenyl)-2-(1-(4-chlorobenzoyl)-5-methoxy-2-methyl-1*H*-indol-3-yl)acetamide (**3br**) S47

N-(4-Bromophenyl)-4'-((1,7'-dimethyl-2'-propyl-1*H*,3'*H*-[2,5'-bibenzo[*d*]imidazol]-3'-yl)methyl)-[1,1'-biphenyl]-2-carboxamide (**3bs**) S48

Benzyl 2-((4-bromophenyl)carbamoyl)-5-oxopyrrolidine-1-carboxylate (**3bt**) S49

tert-Butyl (1-((4-bromophenyl)amino)-3-methyl-1-oxobutan-2-yl)(methyl)carbamate (**3bu**) S50

tert-Butyl (3-(4-(benzyloxy)phenyl)-1-((4-bromophenyl)amino)-1-oxopropan-2-yl)(methyl)carbamate (**3bv**) S50

Benzyl (1-((4-bromophenyl)amino)-3-methyl-1-oxobutan-2-yl)(methyl)carbamate (**3bw**) S51

Benzyl (1-((4-bromophenyl)amino)-1-oxo-3-phenylpropan-2-yl)(methyl)carbamate (**3bx**) S52

6-Phenylphenanthridine (**4a**) S52

6-(Phenylethynyl)phenanthridine (**4b**) S53

IV. NMR spectra S54

N-(4-Bromophenyl)cyclohexanecarboxamide (**3a**) S54

N-Phenylcyclohexanecarboxamide (**3b**) S55

N-(4-Chlorophenyl)cyclohexanecarboxamide (**3c**) S56

N-(*p*-Tolyl)cyclohexanecarboxamide (**3d**) S57

N-(4-Methoxyphenyl)cyclohexanecarboxamide (**3e**) S58

N-(4-Ethoxyphenyl)cyclohexanecarboxamide (**3f**) S59

N-(4-Fluorophenyl)cyclohexanecarboxamide (**3g**) S60

N-(4-(Trifluoromethyl)phenyl)cyclohexanecarboxamide (**3h**) S61

N-(3-Chlorophenyl)cyclohexanecarboxamide (**3i**) S62

N-(*m*-Tolyl)cyclohexanecarboxamide (**3j**) S63

N-(2-Chlorophenyl)cyclohexanecarboxamide (**3k**) S64

N-(*o*-Tolyl)cyclohexanecarboxamide (**3l**) S65

N-(2,4-Dimethylphenyl)cyclohexanecarboxamide (**3m**) S66

N-(2,4-Dichlorophenyl)cyclohexanecarboxamide (**3n**) S67

N-(2-Bromo-4-chlorophenyl)cyclohexanecarboxamide (**3o**) S68

N-(2-Bromo-4-fluorophenyl)cyclohexanecarboxamide (**3p**) S69

N-(2-Bromo-4-methylphenyl)cyclohexanecarboxamide (**3q**) S70

N-(3,4-Dimethylphenyl)cyclohexanecarboxamide (**3r**) S71

N-(3,4-Dichlorophenyl)cyclohexanecarboxamide (**3s**) S72

N-(3,5-Dimethylphenyl)cyclohexanecarboxamide (**3t**) S73

N-(Naphthalen-1-yl)cyclohexanecarboxamide (**3u**) S74

tert-Butyl 4-((4-bromophenyl)carbamoyl)-4-methylpiperidine-1-carboxylate (**3v**) S75

N-(4-Bromophenyl)cyclobutanecarboxamide (**3w**) S76

N-(4-Bromophenyl)cyclopentanecarboxamide (**3x**) S77

N-(4-Bromophenyl)cyclopent-1-ene-1-carboxamide (**3y**) S78

N-(4-Bromophenyl)-2-methylpentanamide (**3z**) S79

N-(4-Bromophenyl)pentanamide (**3aa**) S80

N-(4-Bromophenyl)hexanamide (**3ab**) S81

N-(4-Bromophenyl)heptanamide (**3ac**) S82

N-(4-Bromophenyl)octanamide (**3ad**) S83

N-(4-Bromophenyl)dec-9-enamide (**3ae**) S84

N-(4-Bromophenyl)pivalamide (**3af**) S85

(3*r*,5*r*,7*r*)-*N*-(4-Bromophenyl)adamantane-1-carboxamide (**3ag**) S86

N-(4-Bromophenyl)benzamide (**3ah**) S87

N-(4-Bromophenyl)-4-methylbenzamide (**3ai**) S88

4-Bromo-*N*-(4-bromophenyl)benzamide (**3aj**) S89

N-(4-Bromophenyl)-4-methoxybenzamide (**3ak**) S90

N-(4-Bromophenyl)-2,4,6-trimethylbenzamide (**3al**) S91

N-(4-Bromophenyl)-2-naphthamide (**3am**) S92

N-(4-Bromophenyl)-9,10-dioxo-8*a*,9,10,10*a*-tetrahydroanthracene-2-carboxamide (**3an**) S93

N-(4-Bromophenyl)benzofuran-6-carboxamide (**3ao**) S94

N-(4-Bromophenyl)benzo[*b*]thiophene-6-carboxamide (**3ap**) S95

N-(4-Bromophenyl)furan-2-carboxamide (**3aq**) S96

N-(4-Bromophenyl)thiophene-2-carboxamide (**3ar**) S97

N-(4-Bromophenyl)picolinamide (**3as**) S98

4-Bromo-*N*-(*tert*-butyl)benzamide (**3at**) S99

4-Bromo-*N*-(2-ethylhexyl)benzamide (**3au**) S100

4-Bromo-*N*-cyclopentylbenzamide (**3av**) S101

4-Bromo-*N*-cyclohexylbenzamide (**3aw**) S102

4-Bromo-*N*-(furan-2-yl)benzamide (**3ax**) S103

4-Bromo-*N*-(pyridin-2-yl)benzamide (**3ay**) S104

N-(4-Bromophenyl)cinnamamide (**3az**) S105

(*E*)-*N*-(4-Bromophenyl)-3-(4-methoxyphenyl)acrylamide (**3ba**) S106

(*E*)-*N*-(4-Bromophenyl)-3-(2,5-dimethylphenyl)acrylamide (**3bb**) S107

(*E*)-*N*-(4-Bromophenyl)-2-methyl-3-phenylacrylamide (**3bc**) S108

N-(4-Bromophenyl)-2-oxo-2-phenylacetamide (**3bd**) S109

2-(3-Bromophenyl)-*N*-(4-bromophenyl)-2-oxoacetamide (**3be**) S110

N-(4-Bromophenyl)-3-phenylpropiolamide (**3bf**) S111

N-(4-Bromophenyl)-3-(3,5-dimethylphenyl)propiolamide (**3bg**) S112

N-(4-Bromophenyl)-3-(2,4-dimethylphenyl)propiolamide (**3bh**) S113

N-(4-Bromophenyl)-3-(4-(*tert*-butyl)phenyl)propiolamide (**3bi**) S114

N-(4-Bromophenyl)-3-(4-chlorophenyl)propiolamide (**3bj**) S115

N-(4-Bromophenyl)-3-(4-fluorophenyl)propiolamide (**3bk**) S116

(1*R*)-*N*-(4-Bromophenyl)-7-isopropyl-1,4a-dimethyl-1,2,3,4,4a,4b,5,6,10,10a-decahydrophenanthrene-1-carboxamide (**3bl**) S117

N-(4-Bromophenyl)-2-(4-isobutylphenyl)propanamide (**3bm**) S118

N-(4-Bromophenyl)-2-(4-(4-chlorobenzoyl)phenoxy)-2-methylpropanamide (**3bn**) S119

N-(4-Bromophenyl)-2-(4-(2,2-dichlorocyclopropyl)phenoxy)-2-methylpropanamide (**3bo**) S120

N-(4-Bromophenyl)-5-(2,5-dimethylphenoxy)-2,2-dimethylpentanamide (**3bp**) S121

(*R*)-*N*-(4-Bromophenyl)-4-((5*R*,8*R*,9*S*,10*S*,13*R*,14*S*,17*R*)-10,13-dimethyl-3,7,12-trioxohexadecahydro-1*H*-cyclopenta[*a*]phenanthren-17-yl)pentanamide (**3bq**) S122

N-(4-Bromophenyl)-2-(1-(4-chlorobenzoyl)-5-methoxy-2-methyl-1*H*-indol-3-yl)acetamide (**3br**) S123

N-(4-Bromophenyl)-4'-((1,7'-dimethyl-2'-propyl-1*H*,3'*H*-[2,5'-bibenzo[*d*]imidazol]-3'-yl)methyl)-[1,1'-biphenyl]-2-carboxamide (**3bs**) S124

Benzyl 2-((4-bromophenyl)carbamoyl)-5-oxopyrrolidine-1-carboxylate (**3bt**) S125

tert-Butyl (1-((4-bromophenyl)amino)-3-methyl-1-oxobutan-2-yl)(methyl)carbamate (**3bu**) S126

tert-Butyl (3-(4-(benzyloxy)phenyl)-1-((4-bromophenyl)amino)-1-oxopropan-2-yl)(methyl)carbamate (**3bv**) S127

Benzyl (1-((4-bromophenyl)amino)-3-methyl-1-oxobutan-2-yl)(methyl)carbamate (**3bw**) S128

Benzyl (1-((4-bromophenyl)amino)-1-oxo-3-phenylpropan-2-yl)(methyl)carbamate (**3bx**) S129

6-Phenylphenanthridine (**4a**) S130

6-(Phenylethynyl)phenanthridine (**4b**) S131

2,2,6,6-Tetramethyl-1-phenoxypiperidine (**5**) S132

V. References S133

I. General information

All reagents were purchased from commercial sources and used without treatment unless otherwise indicated. The products were purified by column chromatography over silica gel. ^{1}H NMR and ^{13}C NMR spectra were recorded at 25 °C on a Varian spectrometer at 400 MHz and 101 MHz, respectively, with TMS as the internal standard. Mass spectra were recorded on a BRUKER AutoflexIII Smartbeam MS-spectrometer. High-resolution mass spectra (HRMS) were recorded on a Bruker microTof using an ESI-TOF method.

II. Crystallography

Compound **3bn** (50 mg) was dissolved in a centrifuge tube in 150 μL $CDCl_3$. Upon standing for several days (seven days), crystals suitable for X-ray diffraction of compound **3bn** were obtained. Single-crystal X-ray diffraction data for the reported compound was recorded at a temperature of 296(2) K on an Oxford Diffraction Gemini R Ultra diffractometer using a ω scan technique with Mo-Kα radiation (λ = 0.71073 Å). The structures were solved by the Direct Method of SHELXS-97 and refined by full-matrix least-squares techniques using the SHELXL-97 program. Non-hydrogen atoms were refined with anisotropic temperature parameters, and the hydrogen atoms of the ligands were refined as rigid groups. Basic information pertaining to crystal parameters and structure refinement is summarized in Table S1.

Table S1. Crystal structure and refinement data for compound **3bn** (thermal ellipsoids at 30% probability).

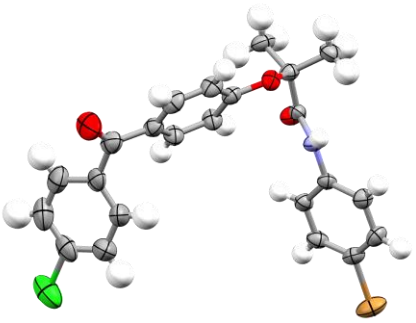

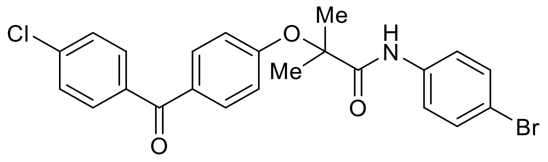

Empirical formula	$C_{23}H_{19}BrClNO_3$
Temperature	296(2) K
Wavelength	0.71073 Å
Space group	P2(1)/n
Unit cell dimensions	a = 5.3594(7) Å<br>b = 17.034(2) Å<br>c = 24.510(3) Å<br>alpha = 70.358(10) deg.<br>beta = 89.889(7) deg.<br>gamma = 89.913(6) deg.
Volume	2107.4(5) $Å^3$
Z	4
Calculated density	1.490 Mg/m^3
Absorption coefficient	2.101 mm^{-1}
F(000)	960
Crystal size	0.124 x 0.112 x 0.089 mm
Theta range for data collection	0.882 to 25.009 deg.
Reflections collected / unique	35921 / 7419 [R(int) = 0.0329]
Data / restraints / parameters	7419 / 2 / 531
Goodness-of-fit on F^2	1.028
Final R indices [I>2sigma(I)]	R1 = 0.0409, wR2 = 0.0969
R indices (all data)	R1 = 0.0634, wR2 = 0.1074

III. Synthesis and analytical data of compounds 1 and 3

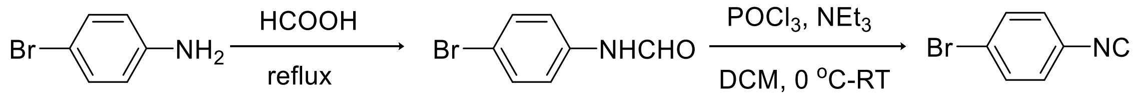

General procedure for synthesis of isocyanides 1: A solution of the amine (20.0 mmol, 1.0 equiv.) was stirred at 0 °C, formic acid (34.6 mmol, 1.73 equiv.) was dropwise added. Then, the reaction mixture was heated to reflux overnight. Upon completion, the solvents were removed under vacuum and the crude product was directly used for the next step without further purification. To a CH_2Cl_2 solution containing the crude product and Et_3N (100.0 mmol, 5.0 equiv.), $POCl_3$ (0.95 equiv.) was added dropwise over 20 minutes. The resulting mixture was stirred at 0 °C for 1 h and further stirred at ambient temperature for 2 h. A sodium bicarbonate solution was added and the resulting mixture was extracted with CH_2Cl_2. The solvents were removed under vacuum and the crude product was purified by flash chromatography to give the pure isocyanide **1**.

Note: *All isocyanides* ***1*** *have been previously reported and all carboxylic acids* ***2*** *are commercially available.*

Reaction optimization

At the outset, a survey of silver salts (30 mol%) were screened in DMF with K_2CO_3 as the base at 80 °C (Table S1, entries 1–6). The results showed that both Ag_2CO_3 and AgOAc were effective catalysts (Table S1, entries 1 and 4, respectively), while other silver salts did not furnish the desired amide product **3a**. Other types of metal salt catalysts, such as $Pd(OAc)_2$ and CuI, were also evaluated and proved to be ineffective, providing a complex mixture of products (Table S1, entries 7–8). Besides K_2CO_3, other common bases, such as Na_2CO_3, Cs_2CO_3 and ^{t}BuOK, were also assessed and revealed to have a minor effect on the desired transformation (Table S1, entries 9–11). Further screening revealed that the reaction could still proceed efficiently in the absence of the base and at 20 mol% loading of the catalyst (Table S1, entries 12–14). Solvents had a significant effect on the reaction, with the polar solvents DMF, DMSO and acetone favoring the reaction, while non-polar solvents, such as xylene and toluene, dramatically reduced the yield of the desired product (Table S1, entries 15–18). The highest yield was observed when using acetone as the solvent. The effect of the reaction temperature was investigated and 60 °C was found to be optimal for the desired transformation (Table S1, entries 16, 19 and 20). Finally, control experiments confirmed that the reaction did not proceed in the absence of the silver catalyst (Table S1, entry 21).

Table S1. Optimization of the reaction conditions.[a]

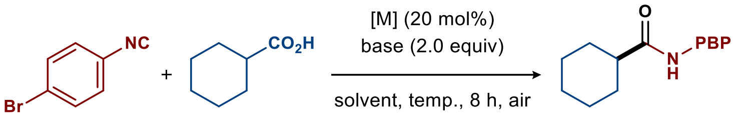

Entry	[M]	Base	Solvent	Temp	Yield[b]
1	Ag_2CO_3	K_2CO_3	DMF	80	63
2	AgF	K_2CO_3	DMF	80	0
3	Ag_2O	K_2CO_3	DMF	80	0
4	AgOAc	K_2CO_3	DMF	80	56
5	AgOTf	K_2CO_3	DMF	80	0
6	$AgNO_3$	K_2CO_3	DMF	80	0
7	$Pd(OAc)_2$	K_2CO_3	DMF	80	0
8	CuI	K_2CO_3	DMF	80	0
9	Ag_2CO_3	Na_2CO_3	DMF	80	61
10	Ag_2CO_3	Cs_2CO_3	DMF	80	64
11	Ag_2CO_3	^{t}BuOK	DMF	80	59
12	Ag_2CO_3	-	DMF	80	62
13[c]	Ag_2CO_3	-	DMF	80	66
14[d]	Ag_2CO_3	-	DMF	80	39
15	Ag_2CO_3	-	DMSO	80	54
16[e]	Ag_2CO_3	-	acetone	80	73
17	Ag_2CO_3	-	xylene	80	31
18	Ag_2CO_3	-	toluene	80	27
19[e]	Ag_2CO_3	-	acetone	60	84
20[e]	Ag_2CO_3	-	acetone	40	76
21[e]	-	K_2CO_3	acetone	60	0

[a] Reaction conditions: **1a** (112 mg, 0.5 mmol), **2a** (198 mg, 1.0 mmol), catalyst (0.10 mmol), H_2O (18 μL, 1.0 mmol), solvent (5.0 mL), room temperature. [b] Isolated yields. [c] 30 mol% of catalyst. [d] 10 mol% of catalyst. [e] The ratio of acetone to water was also investigated and 20:1 was found to be the most suitable. PBP = 4-bromo-C_6H_4.

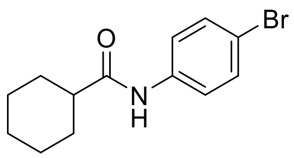

N-(4-Bromophenyl)cyclohexanecarboxamide (3a)[1]

To a 10 mL Schlenk tube equipped with a magnetic stir bar was added carboxylic acid **2a** (128.2 mg, 1 mmol, 2.0 equiv), isocyanide **1a** (91.0 mg, 0.5 mmol, 1.0 equiv), acetone (5.0 mL), H_2O (250 μL), Ag_2CO_3 (27.6 mg, 0.10 mmol, 0.2 equiv). The reaction mixture was stirred under air at 60 °C in an oil bath for about 8 h. The resulting mixture was concentrated and the residue was taken up in ethyl acetate. The organic layer was washed with brine, dried over Na_2SO_4 and concentrated. Purification of the crude product by column chromatography (silica gel; petroleum ether/ethyl acetate = 15:1) afforded **3a** in 84% yield (118 mg).

Faint white solid; mp 188–190 °C; ^{1}H NMR ($CDCl_3$, 400 MHz): δ_H 7.45–7.40 (m, 4H), 7.20 (s, 1H), 2.25–2.19 (m, 1H), 1.97–1.93 (m, 2H), 1.85–1.83 (m, 2H), 1.62–1.48 (m, 3H), 1.35–1.23 (m, 3H); ^{13}C NMR ($CDCl_3$, 101 MHz): δ_C 174.4, 137.1, 131.9, 121.3, 116.6, 46.5, 29.6, 25.6; HRMS (ESI-TOF, *m/z*): calcd for $C_{13}H_{17}BrNO$ $[M + H]^+$, 282.0488; found, 282.0487.

1 mmol scale synthesis of *N*-(4-bromophenyl)cyclohexanecarboxamide (3a)

To a 25 mL Schlenk tube equipped with a magnetic stir bar was added carboxylic acid **2a** (256 mg, 2 mmol, 2.0 equiv), isocyanide **1a** (182.0 mg, 1 mmol, 1.0 equiv), acetone (10.0 mL), H_2O (500 μL), Ag_2CO_3 (55 mg, 0.20 mmol, 0.2 equiv). The reaction mixture was stirred under air at 60 °C in an oil bath for about 8 h. The resulting mixture was concentrated and the residue was taken up in ethyl acetate. The organic layer was washed with brine, dried over Na_2SO_4 and concentrated. Purification of the crude product by column chromatography (silica gel; petroleum ether/ethyl acetate = 15:1) afforded **3a** in 79% yield (221 mg).

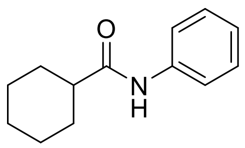

***N*-Phenylcyclohexanecarboxamide (3b)**[2]

To a 10 mL Schlenk tube equipped with a magnetic stir bar was added carboxylic acid **2a** (128.2 mg, 1 mmol, 2.0 equiv), isocyanide **1b** (51.6 mg, 0.5 mmol, 1.0 equiv), acetone (5.0 mL), H_2O (250 µL), Ag_2CO_3 (27.6 mg, 0.10 mmol, 0.2 equiv). The reaction mixture was stirred under air at 60 °C in an oil bath for about 8 h. The resulting mixture was concentrated and the residue was taken up in ethyl acetate. The organic layer was washed with brine, dried over Na_2SO_4 and concentrated. Purification of the crude product by column chromatography (silica gel; petroleum ether/ethyl acetate = 15:1) afforded **3b** in 82% yield (83 mg).

Faint white solid; mp 131–132 °C; ^{1}H NMR ($CDCl_3$, 400 MHz): δ_H 7.53 (d, *J* = 7.6 Hz, 2H), 7.42 (s, 1H), 7.32–7.28 (m, 2H), 7.10–7.06 (m, 1H), 2.26–2.20 (m, 1H), 1.96–1.93 (m, 2H), 1.85–1.81 (m, 2H), 1.71–1.67 (m, 1H), 1.58–1.49 (m, 2H), 1.34–1.19 (m, 3H); ^{13}C NMR ($CDCl_3$, 101 MHz): δ_C 174.4, 138.1, 128.9, 124.0, 119.7, 46.5, 29.6, 25.6; HRMS (ESI-TOF, *m/z*): calcd for $C_{13}H_{18}NO$ $[M + H]^+$, 204.1383; found, 204.1384.

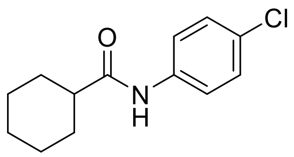

***N*-(4-Chlorophenyl)cyclohexanecarboxamide (3c)**[1]

To a 10 mL Schlenk tube equipped with a magnetic stir bar was added carboxylic acid **2a** (128.2 mg, 1 mmol, 2.0 equiv), isocyanide **1c** (68.8 mg, 0.5 mmol, 1.0 equiv), acetone (5.0 mL), H_2O (250 µL), Ag_2CO_3 (27.6 mg, 0.10 mmol, 0.2 equiv). The reaction mixture was stirred under air at 60 °C in an oil bath for about 8 h. The resulting mixture was concentrated and the residue was taken up in ethyl acetate. The organic layer was washed with brine, dried over Na_2SO_4 and concentrated. Purification of the crude product by column chromatography (silica gel; petroleum ether/ethyl acetate = 15:1) afforded **3c** in 79% yield (94 mg).

Faint white solid; mp 142–143 °C; ^{1}H NMR ($CDCl_3$, 400 MHz): δ_H 7.48 (d, *J* = 8.0 Hz, 2H), 7.26 (d, *J* = 8.0 Hz, 2H), 7.21 (s, 1H), 2.25–2.18 (m, 1H), 1.96–1.93 (m, 2H), 1.86–1.82 (m, 2H), 1.72–1.69 (m, 1H), 1.57–

1.48 (m, 2H), 1.35–1.23 (m, 3H); ^{13}C NMR ($CDCl_3$, 101 MHz): δ_C 174.4, 136.6, 128.9, 121.0, 46.5, 29.6, 25.6; HRMS (ESI-TOF, *m/z*): calcd for $C_{13}H_{17}ClNO$ $[M + H]^+$, 238.0993; found, 238.0976.

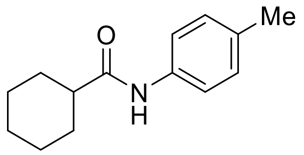

***N*-(*p*-Tolyl)cyclohexanecarboxamide (3d)**[1]

To a 10 mL Schlenk tube equipped with a magnetic stir bar was added carboxylic acid **2a** (128.2 mg, 1 mmol, 2.0 equiv), isocyanide **1d** (58.5 mg, 0.5 mmol, 1.0 equiv), acetone (5.0 mL), H_2O (250 µL), Ag_2CO_3 (27.6 mg, 0.10 mmol, 0.2 equiv). The reaction mixture was stirred under air at 60 °C in an oil bath for about 8 h. The resulting mixture was concentrated and the residue was taken up in ethyl acetate. The organic layer was washed with brine, dried over Na_2SO_4 and concentrated. Purification of the crude product by column chromatography (silica gel; petroleum ether/ethyl acetate = 15:1) afforded **3d** in 86% yield (93 mg).

Faint white solid; mp 154–156 °C; ^{1}H NMR ($CDCl_3$, 400 MHz): δ_H 7.40 (d, *J* = 8.0 Hz, 2H), 7.26 (s, 1H), 7.10 (d, *J* = 8.0 Hz, 2H), 2.30 (s, 3H), 2.24–2.18 (m, 1H), 1.96–1.92 (m, 2H), 1.84–1.80 (m, 2H), 1.71–1.66 (m, 1H), 1.57–1.48 (m, 2H), 1.34–1.22 (m, 3H); ^{13}C NMR ($CDCl_3$, 101 MHz): δ_C 174.3, 135.5, 133.6, 129.4, 119.8, 46.4, 29.6, 25.6, 20.8; HRMS (ESI-TOF, *m/z*): calcd for $C_{14}H_{20}NO$ $[M + H]^+$, 218.1539; found, 218.1539.

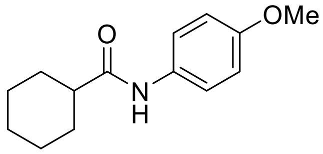

***N*-(4-Methoxyphenyl)cyclohexanecarboxamide (3e)**[3]

To a 10 mL Schlenk tube equipped with a magnetic stir bar was added carboxylic acid **2a** (128.2 mg, 1 mmol, 2.0 equiv), isocyanide **1e** (66.5 mg, 0.5 mmol, 1.0 equiv), acetone (5.0 mL), H_2O (250 µL), Ag_2CO_3 (27.6 mg, 0.10 mmol, 0.2 equiv). The reaction mixture was stirred under air at 60 °C in an oil bath for about 8 h. The resulting mixture was concentrated and the residue was taken up in ethyl acetate. The organic layer was washed with brine, dried over Na_2SO_4 and concentrated. Purification of the crude

product by column chromatography (silica gel; petroleum ether/ethyl acetate = 15:1) afforded **3e** in 89% yield (104 mg).

Faint white solid; mp 147–149 °C; ^{1}H NMR ($CDCl_3$, 400 MHz): δ_H 7.42 (d, J = 8.4 Hz, 2H), 7.17 (s, 1H), 6.84 (d, J = 8.0 Hz, 2H), 3.78 (s, 3H), 2.24–2.18 (m, 1H), 1.96–1.93 (m, 2H), 1.85–1.82 (m, 2H), 1.58–1.49 (m, 2H), 1.42–1.22 (m, 4H); ^{13}C NMR ($CDCl_3$, 101 MHz): δ_C 174.2, 156.2, 131.2, 121.6, 114.0, 55.4, 46.3, 45.8, 29.7, 25.7, 8.6; HRMS (ESI-TOF, *m/z*): calcd for $C_{14}H_{20}NO_2$ [M + H]$^+$, 234.1489; found, 234.1490.

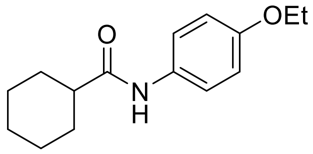

N-(4-Ethoxyphenyl)cyclohexanecarboxamide (3f)

To a 10 mL Schlenk tube equipped with a magnetic stir bar was added carboxylic acid **2a** (128.2 mg, 1 mmol, 2.0 equiv), isocyanide **1f** (73.5 mg, 0.5 mmol, 1.0 equiv), acetone (5.0 mL), H_2O (250 μL), Ag_2CO_3 (27.6 mg, 0.10 mmol, 0.2 equiv). The reaction mixture was stirred under air at 60 °C in an oil bath for about 8 h. The resulting mixture was concentrated and the residue was taken up in ethyl acetate. The organic layer was washed with brine, dried over Na_2SO_4 and concentrated. Purification of the crude product by column chromatography (silica gel; petroleum ether/ethyl acetate = 10:1) afforded **3f** in 88% yield (109 mg).

Faint white solid; mp 156–158 °C; ^{1}H NMR ($CDCl_3$, 400 MHz): δ_H 7.40 (d, J = 8.8 Hz, 2H), 7.15 (s, 1H), 6.83 (d, J = 8.4 Hz, 2H), 4.00 (q, J = 6.8 Hz, 2H), 2.23–2.17 (m, 1H), 1.96–1.93 (m, 2H), 1.85–1.82 (m, 2H), 1.68 (s, 3H), 1.41–1.38 (m, 3H), 1.32–1.23 (m, 3H); ^{13}C NMR ($CDCl_3$, 101 MHz): δ_C 174.2, 155.6, 131.0, 121.6, 114.7, 63.7, 46.4, 29.7, 25.7, 14.8; HRMS (ESI-TOF, *m/z*): calcd for $C_{15}H_{22}NO_2$ [M + H]$^+$, 248.1645; found, 248.1649.

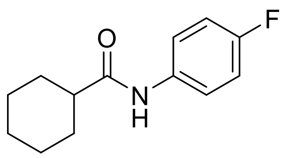

***N*-(4-Fluorophenyl)cyclohexanecarboxamide (3g)**[1]

To a 10 mL Schlenk tube equipped with a magnetic stir bar was added carboxylic acid **2a** (128.2 mg, 1 mmol, 2.0 equiv), isocyanide **1g** (60.5 mg, 0.5 mmol, 1.0 equiv), acetone (5.0 mL), H_2O (250 μL), Ag_2CO_3 (27.6 mg, 0.10 mmol, 0.2 equiv). The reaction mixture was stirred under air at 60 °C in an oil bath for about 8 h. The resulting mixture was concentrated and the residue was taken up in ethyl acetate. The organic layer was washed with brine, dried over Na_2SO_4 and concentrated. Purification of the crude product by column chromatography (silica gel; petroleum ether/ethyl acetate = 15:1) afforded **3g** in 71% yield (79 mg).

Faint white solid; mp 101–103 °C; ^{1}H NMR ($CDCl_3$, 400 MHz): δ_H 7.52 (d, *J* = 11.2 Hz, 1H), 7.43 (s, 1H), 7.27–7.21 (m, 1H), 7.15 (d, *J* = 8.0 Hz, 1H), 6.81–6.77 (m, 1H), 2.26–2.20 (m, 1H), 1.96–1.92 (m, 2H), 1.85–1.82 (m, 2H), 1.72–1.68 (m, 1H), 1.58–1.49 (m, 2H), 1.34–1.26 (m, 3H); ^{13}C NMR ($CDCl_3$, 101 MHz): δ_C 174.6, 162.9 (d, $J_{C\text{–}F}$ = 243.1 Hz), 139.7 (d, $J_{C\text{–}F}$ = 10.9 Hz), 130.0 (d, $J_{C\text{–}F}$ = 9.3 Hz), 114.9, 110.7 (d, $J_{C\text{–}F}$ = 21.2 Hz), 107.2 (d, $J_{C\text{–}F}$ = 26.2 Hz), 46.5, 29.6, 25.6; HRMS (ESI-TOF, *m/z*): calcd for $C_{13}H_{17}FNO$ [M + H]$^+$, 222.1289; found, 222.1289.

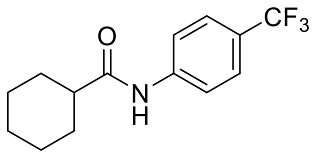

***N*-(4-(Trifluoromethyl)phenyl)cyclohexanecarboxamide (3h)**[3]

To a 10 mL Schlenk tube equipped with a magnetic stir bar was added carboxylic acid **2a** (128.2 mg, 1 mmol, 2.0 equiv), isocyanide **1h** (85.5 mg, 0.5 mmol, 1.0 equiv), acetone (5.0 mL), H_2O (250 μL), Ag_2CO_3 (27.6 mg, 0.10 mmol, 0.2 equiv). The reaction mixture was stirred under air at 60 °C in an oil bath for about 8 h. The resulting mixture was concentrated and the residue was taken up in ethyl acetate. The organic layer was washed with brine, dried over Na_2SO_4 and concentrated. Purification of the crude product by column chromatography (silica gel; petroleum ether/ethyl acetate = 15:1) afforded **3h** in 64% yield (87 mg).

Faint yellow solid; mp 164–166 °C; ^1H NMR ($CDCl_3$, 400 MHz): δ_H 7.66 (d, J = 8.4 Hz, 2H), 7.55 (d, J = 8.4 Hz, 2H), 7.51 (s, 1H), 2.29–2.23 (m, 1H), 1.97–1.94 (m, 2H), 1.86–1.82 (m, 2H), 1.72–1.70 (m, 2H), 1.53–1.50 (m, 1H), 1.35–1.23 (m, 3H); ^{13}C NMR ($CDCl_3$, 101 MHz): δ_C 174.8, 141.1, 126.23 (d, $J_{C–F}$ = 11.2 Hz), 126.20 (d, $J_{C–F}$ = 3.8 Hz), 119.3, 46.5, 25.6; HRMS (ESI-TOF, *m/z*): calcd for $C_{14}H_{17}F_3NO$ [M + H]$^+$, 272.1257; found, 272.1255.

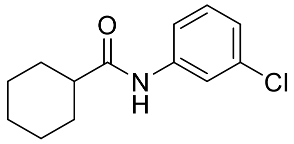

***N*-(3-Chlorophenyl)cyclohexanecarboxamide (3i)**[2]

To a 10 mL Schlenk tube equipped with a magnetic stir bar was added carboxylic acid **2a** (128.2 mg, 1 mmol, 2.0 equiv), isocyanide **1i** (68.5 mg, 0.5 mmol, 1.0 equiv), acetone (5.0 mL), H_2O (250 µL), Ag_2CO_3 (27.6 mg, 0.10 mmol, 0.2 equiv). The reaction mixture was stirred under air at 60 °C in an oil bath for about 8 h. The resulting mixture was concentrated and the residue was taken up in ethyl acetate. The organic layer was washed with brine, dried over Na_2SO_4 and concentrated. Purification of the crude product by column chromatography (silica gel; petroleum ether/ethyl acetate = 15:1) afforded **3i** in 77% yield (91 mg).

Faint white solid; mp 111–115 °C; ^1H NMR ($CDCl_3$, 400 MHz): δ_H 8.40 (d, J = 8.4 Hz, 1H), 7.71 (s, 1H), 7.36 (d, J = 8.0 Hz, 1H), 7.28–7.24 (m, 1H), 7.04–7.00 (m, 1H), 2.35–2.28 (m, 1H), 2.04–2.00 (m, 2H), 1.88–1.83 (m, 2H), 1.74–1.70 (m, 1H), 1.60–1.50 (m, 2H), 1.40–1.25 (m, 3H); ^{13}C NMR ($CDCl_3$, 101 MHz): δ_C 174.2, 134.7, 128.9, 127.7, 124.3, 122.6, 121.5, 46.6, 29.6, 25.6; HRMS (ESI-TOF, *m/z*): calcd for $C_{13}H_{17}ClNO$ [M + H]$^+$, 238.0993; found, 238.0976.

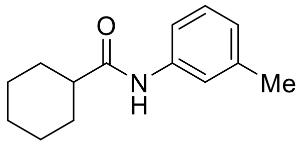

***N*-(*m*-Tolyl)cyclohexanecarboxamide (3j)**[2]

To a 10 mL Schlenk tube equipped with a magnetic stir bar was added carboxylic acid **2a** (128.2 mg, 1 mmol, 2.0 equiv), isocyanide **1j** (58.5 mg, 0.5 mmol, 1.0 equiv), acetone (5.0 mL), H_2O (250 µL), Ag_2CO_3 (27.6 mg, 0.10 mmol, 0.2 equiv). The reaction mixture was stirred under air at 60 °C in an oil bath for about 8 h. The resulting mixture was concentrated and the residue was taken up in ethyl acetate. The organic layer was washed with brine, dried over Na_2SO_4 and concentrated. Purification of the crude product by column chromatography (silica gel; petroleum ether/ethyl acetate = 15:1) afforded **3j** in 81% yield (88 mg).

Faint white solid; mp 102–104 °C; ^{1}H NMR ($CDCl_3$, 400 MHz): δ_H 7.42 (s, 1H), 7.29–7.26 (m, 2H), 7.20–7.16 (m, 1H), 6.91 (d, *J* = 7.2 Hz, 1H), 2.32 (s, 3H), 2.24–2.19 (m, 1H), 1.96–1.93 (m, 2H), 1.84–1.81 (m, 2H), 1.75–1.70 (m, 1H), 1.58–1.49 (m, 2H), 1.35–1.23 (m, 3H); ^{13}C NMR ($CDCl_3$, 101 MHz): δ_C 174.4, 138.9, 138.0, 128.7, 124.8, 120.4, 116.8, 46.5, 29.6, 25.6, 21.4; HRMS (ESI-TOF, *m/z*): calcd for $C_{14}H_{20}NO$ $[M + H]^+$, 218.1539; found, 218.1539.

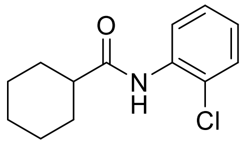

***N*-(2-Chlorophenyl)cyclohexanecarboxamide (3k)**[3]

To a 10 mL Schlenk tube equipped with a magnetic stir bar was added carboxylic acid **2a** (128.2 mg, 1 mmol, 2.0 equiv), isocyanide **1k** (68.5 mg, 0.5 mmol, 1.0 equiv), acetone (5.0 mL), H_2O (250 µL), Ag_2CO_3 (27.6 mg, 0.10 mmol, 0.2 equiv). The reaction mixture was stirred under air at 60 °C in an oil bath for about 8 h. The resulting mixture was concentrated and the residue was taken up in ethyl acetate. The organic layer was washed with brine, dried over Na_2SO_4 and concentrated. Purification of the crude product by column chromatography (silica gel; petroleum ether/ethyl acetate = 15:1) afforded **3k** in 68% yield (81 mg).

Faint white solid; mp 126–128 °C; ^{1}H NMR ($CDCl_3$, 400 MHz): δ_H 7.80 (s, 1H), 7.61 (s, 1H), 7.38–7.32 (m, 2H), 3.13–3.06 (m, 1H), 2.29–2.21 (m, 1H), 1.95–1.94 (m, 2H), 1.85–1.80 (m, 2H), 1.57–1.48 (m, 2H),

1.42–1.38 (m, 1H), 1.33–1.22 (m, 3H); ^{13}C NMR ($CDCl_3$, 101 MHz): δ_C 174.7, 137.6, 132.6, 130.3, 127.1, 121.5, 119.0, 46.3, 45.9, 29.5, 25.5, 8.6; HRMS (ESI-TOF, *m/z*): calcd for $C_{13}H_{17}ClNO$ [M + H]$^+$, 238.0993; found, 238.0976.

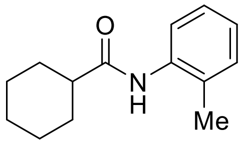

***N*-(*o*-Tolyl)cyclohexanecarboxamide (3l)**[2]

To a 10 mL Schlenk tube equipped with a magnetic stir bar was added carboxylic acid **2a** (128.2 mg, 1 mmol, 2.0 equiv), isocyanide **1l** (58.5 mg, 0.5 mmol, 1.0 equiv), acetone (5.0 mL), H_2O (250 μL), Ag_2CO_3 (27.6 mg, 0.10 mmol, 0.2 equiv). The reaction mixture was stirred under air at 60 °C in an oil bath for about 8 h. The resulting mixture was concentrated and the residue was taken up in ethyl acetate. The organic layer was washed with brine, dried over Na_2SO_4 and concentrated. Purification of the crude product by column chromatography (silica gel; petroleum ether/ethyl acetate = 15:1) afforded **3l** in 73% yield (79 mg).

Faint white solid; mp 147–149 °C; ^1H NMR ($CDCl_3$, 400 MHz): δ_H 7.83–7.79 (m, 1H), 7.21–7.16 (m, 2H), 7.08–7.04 (m, 2H), 2.31–2.28 (m, 1H), 2.25 (m, 3H), 2.01–1.97 (m, 2H), 1.86–1.83 (m, 2H), 1.74–1.70 (m, 1H), 1.59–1.50 (m, 2H), 1.38–1.24 (m, 3H); ^{13}C NMR ($CDCl_3$, 101 MHz): δ_C 174.2, 135.7, 130.3, 128.9, 126.7, 124.9, 123.1, 46.3, 29.8, 25.7, 17.7; HRMS (ESI-TOF, *m/z*): calcd for $C_{14}H_{20}NO$ [M + H]$^+$, 218.1539; found, 218.1539.

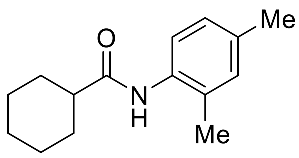

***N*-(2,4-Dimethylphenyl)cyclohexanecarboxamide (3m)**

To a 10 mL Schlenk tube equipped with a magnetic stir bar was added carboxylic acid **2a** (128.2 mg, 1 mmol, 2.0 equiv), isocyanide **1m** (65.5 mg, 0.5 mmol, 1.0 equiv), acetone (5.0 mL), H_2O (250 µL), Ag_2CO_3 (27.6 mg, 0.10 mmol, 0.2 equiv). The reaction mixture was stirred under air at 60 °C in an oil bath for about 8 h. The resulting mixture was concentrated and the residue was taken up in ethyl acetate. The organic layer was washed with brine, dried over Na_2SO_4 and concentrated. Purification of the crude product by column chromatography (silica gel; petroleum ether/ethyl acetate = 15:1) afforded **3m** in 70% yield (81 mg).

Faint white solid; mp 173–175 °C; ^1H NMR ($CDCl_3$, 400 MHz): δ_H 7.60 (d, *J* = 8.0 Hz, 1H), 7.00–6.96 (m, 3H), 2.28 (s, 3H), 2.20 (s, 3H), 2.20–1.97 (m, 2H), 1.86–1.82 (m, 2H), 1.72–1.69 (m, 2H), 1.58–1.49 (m, 2H), 1.38–1.24 (m, 3H); ^{13}C NMR ($CDCl_3$, 101 MHz): δ_C 174.2, 133.9, 131.3, 130.8, 123.3, 122.5, 115.9, 46.6, 29.6, 25.6; HRMS (ESI-TOF, *m/z*): calcd for $C_{15}H_{22}NO$ $[M + H]^+$, 232.1696; found, 232.1698.

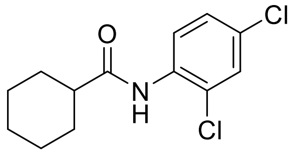

***N*-(2,4-Dichlorophenyl)cyclohexanecarboxamide (3n)**[4]

To a 10 mL Schlenk tube equipped with a magnetic stir bar was added carboxylic acid **2a** (128.2 mg, 1 mmol, 2.0 equiv), isocyanide **1n** (85.5 mg, 0.5 mmol, 1.0 equiv), acetone (5.0 mL), H_2O (250 µL), Ag_2CO_3 (27.6 mg, 0.10 mmol, 0.2 equiv). The reaction mixture was stirred under air at 60 °C in an oil bath for about 8 h. The resulting mixture was concentrated and the residue was taken up in ethyl acetate. The organic layer was washed with brine, dried over Na_2SO_4 and concentrated. Purification of the crude product by column chromatography (silica gel; petroleum ether/ethyl acetate = 15:1) afforded **3n** in 66% yield (89 mg).

Faint white solid; mp 164–166 °C; ^1H NMR ($CDCl_3$, 400 MHz): δ_H 8.38 (d, *J* = 9.2 Hz, 1H), 7.65 (s, 1H), 7.37 (d, *J* = 2.0 Hz, 1H), 7.25–7.23 (m, 1H), 2.35–2.27 (m, 1H), 2.03–1.98 (m, 2H), 1.88–1.83 (m, 2H), 1.74–1.71 (m, 1H), 1.55–1.49 (m, 2H), 1.36–1.25 (m, 3H); ^{13}C NMR ($CDCl_3$, 101 MHz): δ_C 174.2, 133.5,

128.7, 128.6, 127.9, 123.1, 122.2, 46.6, 29.6, 25.6; HRMS (ESI-TOF, *m/z*): calcd for $C_{13}H_{16}Cl_2NO$ [M + H]$^+$, 272.0603; found, 272.0584.

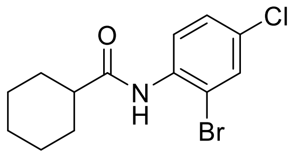

N-(2-Bromo-4-chlorophenyl)cyclohexanecarboxamide (3o)[4]

To a 10 mL Schlenk tube equipped with a magnetic stir bar was added carboxylic acid **2a** (128.2 mg, 1 mmol, 2.0 equiv), isocyanide **1o** (107.5 mg, 0.5 mmol, 1.0 equiv), acetone (5.0 mL), H_2O (250 µL), Ag_2CO_3 (27.6 mg, 0.10 mmol, 0.2 equiv). The reaction mixture was stirred under air at 60 °C in an oil bath for about 8 h. The resulting mixture was concentrated and the residue was taken up in ethyl acetate. The organic layer was washed with brine, dried over Na_2SO_4 and concentrated. Purification of the crude product by column chromatography (silica gel; petroleum ether/ethyl acetate = 15:1) afforded **3o** in 67% yield (106 mg).

Faint white solid; mp 134–136 °C; ^{1}H NMR ($CDCl_3$, 400 MHz): δ_H 8.33 (d, *J* = 8.8 Hz, 1H), 7.66 (s, 1H), 7.51 (s, 1H), 7.38 (d, *J* = 8.8 Hz, 1H), 2.35–2.27 (m, 1H), 2.02–1.98 (m, 2H), 1.88–1.84 (m, 2H), 1.74–1.70 (m, 1H), 1.58–1.49 (m, 2H), 1.37–1.28 (m, 3H); ^{13}C NMR ($CDCl_3$, 101 MHz): δ_C 174.2, 134.7, 133.0, 131.0, 129.3, 127.2, 123.5, 46.3, 29.8, 25.7, 20.8, 17.7; HRMS (ESI-TOF, *m/z*): calcd for $C_{13}H_{16}BrClNO$ [M + H]$^+$, 316.0098; found, 316.0117.

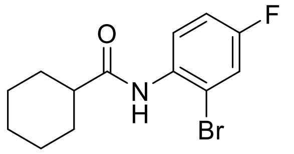

N-(2-Bromo-4-fluorophenyl)cyclohexanecarboxamide (3p)[5]

To a 10 mL Schlenk tube equipped with a magnetic stir bar was added carboxylic acid **2a** (128.2 mg, 1 mmol, 2.0 equiv), isocyanide **1p** (99.5 mg, 0.5 mmol, 1.0 equiv), acetone (5.0 mL), H_2O (250 µL), Ag_2CO_3 (27.6 mg, 0.10 mmol, 0.2 equiv). The reaction mixture was stirred under air at 60 °C in an oil bath for about 8 h. The resulting mixture was concentrated and the residue was taken up in ethyl acetate. The organic layer was washed with brine, dried over Na_2SO_4 and concentrated. Purification of the crude

product by column chromatography (silica gel; petroleum ether/ethyl acetate = 15:1) afforded **3p** in 71% yield (106 mg).

Faint white solid; mp 141–142 °C; ^{1}H NMR ($CDCl_3$, 400 MHz): δ_H 8.34–8.30 (m, 1H), 7.58 (s, 1H), 7.30–7.27 (m, 1H), 7.07–7.02 (m, 1H), 2.34–2.27 (m, 1H), 2.04–2.00 (m, 2H), 1.89–1.83 (m, 2H), 1.74–1.70 (m, 1H), 1.58–1.49 (m, 2H), 1.40–1.25 (m, 3H); ^{13}C NMR ($CDCl_3$, 101 MHz): δ_C 174.2, 158.3 (d, $J_{F\text{-}C}$ = 246.4 Hz), 132.2 (d, $J_{C\text{–}F}$ = 2.9 Hz), 123.0 (d, $J_{C\text{–}F}$ = 7.8 Hz), 119.1 (d, $J_{C\text{–}F}$ = 25.4 Hz), 115.1 (d, $J_{C\text{–}F}$ = 21.4 Hz), 113.5 (d, $J_{C\text{–}F}$ = 9.6 Hz), 46.5, 29.6, 25.6; HRMS (ESI-TOF, *m/z*): calcd for $C_{13}H_{16}BrFNO$ [M + H]$^+$, 300.0394; found, 300.0390.

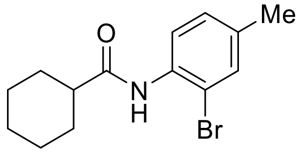

***N*-(2-Bromo-4-methylphenyl)cyclohexanecarboxamide (3q)**

To a 10 mL Schlenk tube equipped with a magnetic stir bar was added carboxylic acid **2a** (128.2 mg, 1 mmol, 2.0 equiv), isocyanide **1q** (97.5 mg, 0.5 mmol, 1.0 equiv), acetone (5.0 mL), H_2O (250 µL), Ag_2CO_3 (27.6 mg, 0.10 mmol, 0.2 equiv). The reaction mixture was stirred under air at 60 °C in an oil bath for about 8 h. The resulting mixture was concentrated and the residue was taken up in ethyl acetate. The organic layer was washed with brine, dried over Na_2SO_4 and concentrated. Purification of the crude product by column chromatography (silica gel; petroleum ether/ethyl acetate = 15:1) afforded **3q** in 74% yield (109 mg).

Faint white solid; mp 138–140 °C; ^{1}H NMR ($CDCl_3$, 400 MHz): δ_H 8.23 (s, 1H), 7.66 (s, 1H), 7.38 (d, *J* = 8.4 Hz, 1H), 6.78 (d, *J* = 8.0 Hz, 1H), 2.31 (s, 3H), 2.04–2.01 (m, 2H), 1.88–1.83 (m, 2H), 1.74–1.70 (m, 1H), 1.56–1.50 (m, 2H), 1.43–1.25 (m, 4H); ^{13}C NMR ($CDCl_3$, 101 MHz): δ_C 174.2, 138.6, 135.3, 131.6, 125.8, 122.3, 110.0, 46.6, 45.7, 29.7, 25.6, 21.3, 8.6; HRMS (ESI-TOF, *m/z*): calcd for $C_{14}H_{19}BrNO$ [M + H]$^+$, 296.0645; found, 296.0641.

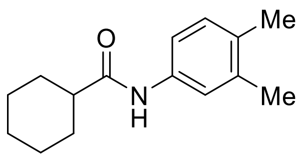

***N*-(3,4-Dimethylphenyl)cyclohexanecarboxamide (3r)**

To a 10 mL Schlenk tube equipped with a magnetic stir bar was added carboxylic acid **2a** (128.2 mg, 1 mmol, 2.0 equiv), isocyanide **1r** (65.5 mg, 0.5 mmol, 1.0 equiv), acetone (5.0 mL), H_2O (250 µL), Ag_2CO_3 (27.6 mg, 0.10 mmol, 0.2 equiv). The reaction mixture was stirred under air at 60 °C in an oil bath for about 8 h. The resulting mixture was concentrated and the residue was taken up in ethyl acetate. The organic layer was washed with brine, dried over Na_2SO_4 and concentrated. Purification of the crude product by column chromatography (silica gel; petroleum ether/ethyl acetate = 15:1) afforded **3r** in 83% yield (96 mg).

Faint white solid; mp 134–135 °C; ^{1}H NMR ($CDCl_3$, 400 MHz): δ_H 7.34 (s, 1H), 7.24–7.20 (m, 2H), 7.04 (d, *J* = 8.0 Hz, 1H), 2.22–2.20 (m, 6H), 1.95–1.92 (m, 2H), 1.83–1.81 (m, 2H), 1.75–1.67 (m, 2H), 1.55–1.51 (m, 2H), 1.34–1.22 (m, 3H); ^{13}C NMR ($CDCl_3$, 101 MHz): δ_C 174.3, 137.1, 135.8, 132.3, 129.8, 121.2, 117.3, 46.5, 29.7, 25.7, 19.8, 19.1; HRMS (ESI-TOF, *m/z*): calcd for $C_{15}H_{22}NO$ [M + H]$^+$, 232.1696; found, 232.1698.

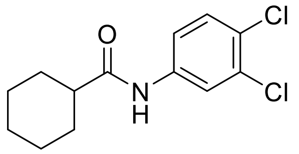

***N*-(3,4-Dichlorophenyl)cyclohexanecarboxamide (3s)**

To a 10 mL Schlenk tube equipped with a magnetic stir bar was added carboxylic acid **2a** (128.2 mg, 1 mmol, 2.0 equiv), isocyanide **1s** (85.5 mg, 0.5 mmol, 1.0 equiv), acetone (5.0 mL), H_2O (250 µL), Ag_2CO_3 (27.6 mg, 0.10 mmol, 0.2 equiv). The reaction mixture was stirred under air at 60 °C in an oil bath for about 8 h. The resulting mixture was concentrated and the residue was taken up in ethyl acetate. The organic layer was washed with brine, dried over Na_2SO_4 and concentrated. Purification of the crude product by column chromatography (silica gel; petroleum ether/ethyl acetate = 15:1) afforded **3s** in 76% yield (103 mg).

Faint white solid; mp 102–103 °C; ^{1}H NMR ($CDCl_3$, 400 MHz): δ_H 7.68 (s, 1H), 7.37 (d, *J* = 8.0 Hz, 1H), 7.21–7.17 (m, 1H), 7.05 (d, *J* = 7.6 Hz, 1H), 2.28–2.21 (m, 1H), 1.94–1.91 (m, 2H), 1.83–1.79 (m, 2H),

1.69–1.67 (m, 1H), 1.56–1.48 (m, 2H), 1.29–1.22 (m, 3H); ^{13}C NMR ($CDCl_3$, 101 MHz): δ_C 174.9, 139.3, 134.5, 129.8, 124.0, 120.0, 117.8, 46.3, 29.5, 25.5; HRMS (ESI-TOF, *m/z*): calcd for $C_{13}H_{16}Cl_2NO$ [M + H]$^+$, 272.0603; found, 272.0584.

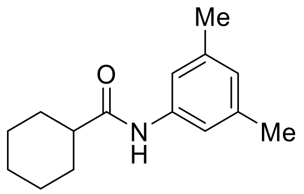

***N*-(3,5-Dimethylphenyl)cyclohexanecarboxamide (3t)**[6]

To a 10 mL Schlenk tube equipped with a magnetic stir bar was added carboxylic acid **2a** (128.2 mg, 1 mmol, 2.0 equiv), isocyanide **1t** (65.5 mg, 0.5 mmol, 1.0 equiv), acetone (5.0 mL), H_2O (250 μL), Ag_2CO_3 (27.6 mg, 0.10 mmol, 0.2 equiv). The reaction mixture was stirred under air at 60 °C in an oil bath for about 8 h. The resulting mixture was concentrated and the residue was taken up in ethyl acetate. The organic layer was washed with brine, dried over Na_2SO_4 and concentrated. Purification of the crude product by column chromatography (silica gel; petroleum ether/ethyl acetate = 15:1) afforded **3t** in 85% yield (98 mg).

Faint white solid; mp 151–152 °C; ^{1}H NMR ($CDCl_3$, 400 MHz): δ_H 7.20–7.17 (m, 3H), 6.73 (s, 1H), 2.28 (s, 6H), 1.95–1.92 (m, 2H), 1.84–1.81 (m, 2H), 1.71–1.67 (m, 2H), 1.57–1.48 (m, 2H), 1.34–1.22 (m, 3H); ^{13}C NMR ($CDCl_3$, 101 MHz): δ_C 174.3, 138.6, 137.9, 125.7, 117.5, 46.5, 29.6, 25.7, 21.3; HRMS (ESI-TOF, *m/z*): calcd for $C_{15}H_{22}NO$ [M + H]$^+$, 232.1696; found, 232.1698.

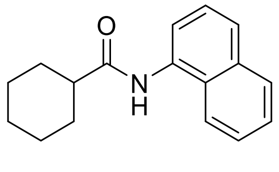

***N*-(Naphthalen-1-yl)cyclohexanecarboxamide (3u)**[7]

To a 10 mL Schlenk tube equipped with a magnetic stir bar was added carboxylic acid **2a** (128.2 mg, 1 mmol, 2.0 equiv), isocyanide **1u** (76.5 mg, 0.5 mmol, 1.0 equiv), acetone (5.0 mL), H_2O (250 μL), Ag_2CO_3 (27.6 mg, 0.10 mmol, 0.2 equiv). The reaction mixture was stirred under air at 60 °C in an oil bath for about 8 h. The resulting mixture was concentrated and the residue was taken up in ethyl acetate. The organic layer was washed with brine, dried over Na_2SO_4 and concentrated. Purification of the crude

product by column chromatography (silica gel; petroleum ether/ethyl acetate = 13:1) afforded **3u** in 83% yield (105 mg).

Faint white solid; mp 183–184 °C; ^{1}H NMR (DMSO-d_6, 400 MHz): δ_H 9.80 (s, 1H), 8.03 (d, J = 8.0 Hz, 1H), 7.93 (d, J = 7.6 Hz, 1H), 7.75 (d, J = 8.4 Hz, 1H), 7.64 (d, J = 7.2 Hz, 1H), 7.57–7.45 (m, 3H), 2.62–2.55 (m, 1H), 1.93 (d, J = 12.8 Hz, 2H), 1.80 (d, J = 12.8 Hz, 2H), 1.69 (d, J = 12.4 Hz, 1H), 1.54–1.44 (m, 2H), 1.38–1.20 (m, 3H); ^{13}C NMR (DMSO-d_6, 101 MHz): δ_C 174.9, 133.7, 128.1, 128.0, 125.9, 125.7, 125.5, 125.1, 122.7, 121.9, 44.3, 29.4, 25.3; HRMS (ESI-TOF, *m/z*): calcd for $C_{17}H_{20}NO$ [M + H]$^+$, 254.1539; found, 254.1547.

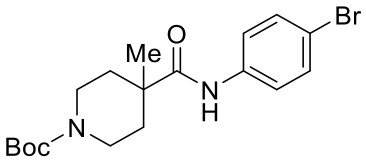

***tert*-Butyl 4-((4-bromophenyl)carbamoyl)-4-methylpiperidine-1-carboxylate (3v)**

To a 10 mL Schlenk tube equipped with a magnetic stir bar was added carboxylic acid **2b** (243.1 mg, 1.0 mmol, 2.0 equiv), isocyanide **1a** (90.5 mg, 0.5 mmol, 1.0 equiv), acetone (5.0 mL), H_2O (250 µL), Ag_2CO_3 (27.6 mg, 0.10 mmol, 0.2 equiv). The reaction mixture was stirred under air at 60 °C in an oil bath for about 8 h. The resulting mixture was concentrated and the residue was taken up in ethyl acetate. The organic layer was washed with brine, dried over Na_2SO_4 and concentrated. Purification of the crude product by column chromatography (silica gel; petroleum ether/ethyl acetate = 10:1) afforded **3v** in 94% yield (186 mg).

Faint white solid; mp 165–166 °C; ^{1}H NMR ($CDCl_3$, 400 MHz): δ_H 7.44–7.40 (m, 4H), 4.15–4.10 (m, 1H), 3.63–3.58 (m, 2H), 3.36–3.30 (m, 2H), 2.08–2.02 (m, 3H), 1.45 (s, 10H), 1.31 (s, 3H); ^{13}C NMR ($CDCl_3$, 101 MHz): δ_C 180.3, 174.6, 154.8, 136.8, 131.9, 121.9, 117.0, 79.7, 60.4, 42.0, 34.6, 28.4, 25.1, 21.0, 14.1; HRMS (ESI-TOF, *m/z*): calcd for $C_{18}H_{25}BrNaN_2O_3$ [M + Na]$^+$, 419.0941; found, 419.0934.

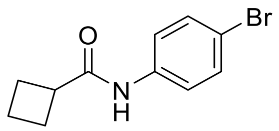

***N*-(4-Bromophenyl)cyclobutanecarboxamide (3w)**

To a 10 mL Schlenk tube equipped with a magnetic stir bar was added carboxylic acid **2c** (100.1 mg, 1.0 mmol, 2.0 equiv), isocyanide **1a** (90.5 mg, 0.5 mmol, 1.0 equiv), acetone (5.0 mL), H_2O (250 µL), Ag_2CO_3 (27.6 mg, 0.10 mmol, 0.2 equiv). The reaction mixture was stirred under air at 60 °C in an oil bath for about 8 h. The resulting mixture was concentrated and the residue was taken up in ethyl acetate. The organic layer was washed with brine, dried over Na_2SO_4 and concentrated. Purification of the crude product by column chromatography (silica gel; petroleum ether/ethyl acetate = 10:1) afforded **3w** in 61% yield (77 mg).

Faint white solid; mp 150–151 °C; ^{1}H NMR ($CDCl_3$, 400 MHz): δ_H 7.45–7.40 (m, 4H), 7.19 (s, 1H), 3.19–3.10 (m, 1H), 2.43–2.33 (m, 2H), 2.25–2.18 (m, 2H), 2.06–1.88 (m, 2H); ^{13}C NMR ($CDCl_3$, 101 MHz): δ_C 173.3, 137.1, 131.9, 121.2, 116.6, 40.8, 25.2, 18.0; HRMS (ESI-TOF, *m/z*): calcd for $C_{11}H_{13}BrNO$ [M + H]$^+$, 254.0175; found, 254.0172.

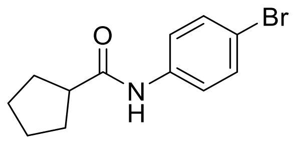

***N*-(4-Bromophenyl)cyclopentanecarboxamide (3x)**[6]

To a 10 mL Schlenk tube equipped with a magnetic stir bar was added carboxylic acid **2d** (114.1 mg, 1.0 mmol, 2.0 equiv), isocyanide **1a** (90.5 mg, 0.5 mmol, 1.0 equiv), acetone (5.0 mL), H_2O (250 µL), Ag_2CO_3 (27.6 mg, 0.10 mmol, 0.2 equiv). The reaction mixture was stirred under air at 60 °C in an oil bath for about 8 h. The resulting mixture was concentrated and the residue was taken up in ethyl acetate. The organic layer was washed with brine, dried over Na_2SO_4 and concentrated. Purification of the crude product by column chromatography (silica gel; petroleum ether/ethyl acetate = 10:1) afforded **3x** in 69% yield (92 mg).

Faint white solid; mp 159–160 °C; ^{1}H NMR ($CDCl_3$, 400 MHz): δ_H 7.45–7.40 (m, 4H), 7.25 (s, 1H), 2.70–2.62 (m, 1H), 1.95–1.83 (m, 4H), 1.82–1.74 (m, 2H), 1.67–1.56 (m, 2H); ^{13}C NMR ($CDCl_3$, 101 MHz): δ_C

174.6, 137.2, 131.9, 121.2, 116.5, 46.8, 30.5, 26.0; HRMS (ESI-TOF, *m/z*): calcd for $C_{12}H_{15}BrNO$ [M + H]$^+$, 268.0332; found, 268.0329.

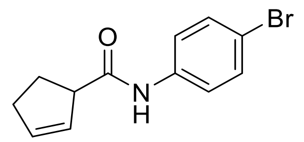

***N*-(4-Bromophenyl)cyclopent-1-ene-1-carboxamide (3y)**

To a 10 mL Schlenk tube equipped with a magnetic stir bar was added carboxylic acid **2e** (112.1 mg, 1.0 mmol, 2.0 equiv), isocyanide **1a** (90.5 mg, 0.5 mmol, 1.0 equiv), acetone (5.0 mL), H_2O (250 µL), Ag_2CO_3 (27.6 mg, 0.10 mmol, 0.2 equiv). The reaction mixture was stirred under air at 60 °C in an oil bath for about 8 h. The resulting mixture was concentrated and the residue was taken up in ethyl acetate. The organic layer was washed with brine, dried over Na_2SO_4 and concentrated. Purification of the crude product by column chromatography (silica gel; petroleum ether/ethyl acetate = 15:1) afforded **3y** in 67% yield (89 mg).

Faint white solid; mp 161–162 °C; ^{1}H NMR ($CDCl_3$, 400 MHz): δ_H 7.49–7.42 (m, 4H), 7.38 (s, 1H), 6.66 (s, 1H), 2.68–2.63 (m, 2H), 2.57–2.53 (m, 2H), 2.08–2.00 (m, 2H); ^{13}C NMR ($CDCl_3$, 101 MHz): δ_C 163.4, 139.6, 139.5, 136.9, 131.9, 121.4, 116.7, 33.4, 31.6, 23.3; HRMS (ESI-TOF, *m/z*): calcd for $C_{12}H_{13}BrNO$ [M + H]$^+$, 266.0175; found, 266.0172.

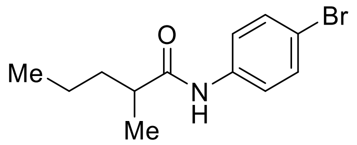

***N*-(4-Bromophenyl)-2-methylpentanamide (3z)**[8]

To a 10 mL Schlenk tube equipped with a magnetic stir bar was added carboxylic acid **2f** (116.1 mg, 1.0 mmol, 2.0 equiv), isocyanide **1a** (90.5 mg, 0.5 mmol, 1.0 equiv), acetone (5.0 mL), H_2O (250 µL), Ag_2CO_3 (27.6 mg, 0.10 mmol, 0.2 equiv). The reaction mixture was stirred under air at 60 °C in an oil bath for about 8 h. The resulting mixture was concentrated and the residue was taken up in ethyl acetate. The organic layer was washed with brine, dried over Na_2SO_4 and concentrated. Purification of the crude product by column chromatography (silica gel; petroleum ether/ethyl acetate = 15:1) afforded **3z** in 64% yield (86 mg).

Faint white solid; mp 117–119 °C; ^{1}H NMR ($CDCl_3$, 400 MHz): δ_H 7.49 (s, 1H), 7.45–7.40 (m, 4H), 2.37–2.31 (m, 1H), 1.72–1.66 (m, 1H), 1.45–1.32 (m, 3H), 1.22–1.20 (m, 3H), 0.94–0.89 (m, 3H); ^{13}C NMR ($CDCl_3$, 101 MHz): δ_C 175.2, 137.0, 131.8, 121.5, 116.7, 42.4, 36.5, 20.6, 17.8, 14.0; HRMS (ESI-TOF, *m/z*): calcd for $C_{12}H_{15}BrNO$ $[M - H]^-$, 268.0343; found, 268.0343.

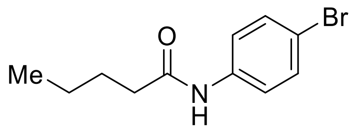

***N*-(4-Bromophenyl)pentanamide (3aa)**

To a 10 mL Schlenk tube equipped with a magnetic stir bar was added carboxylic acid **2g** (102.1 mg, 1.0 mmol, 2.0 equiv), isocyanide **1a** (90.5 mg, 0.5 mmol, 1.0 equiv), acetone (5.0 mL), H_2O (250 µL), Ag_2CO_3 (27.6 mg, 0.10 mmol, 0.2 equiv). The reaction mixture was stirred under air at 60 °C in an oil bath for about 8 h. The resulting mixture was concentrated and the residue was taken up in ethyl acetate. The organic layer was washed with brine, dried over Na_2SO_4 and concentrated. Purification of the crude product by column chromatography (silica gel; petroleum ether/ethyl acetate = 15:1) afforded **3aa** in 58% yield (74 mg).

Faint white solid; mp 93–95 °C; ^{1}H NMR ($CDCl_3$, 400 MHz): δ_H 7.44–7.39 (m, 4H), 7.30 (s, 1H), 2.35 (t, *J* = 7.6 Hz, 2H), 1.70–1.66 (m, 2H), 1.44–1.35 (m, 2H), 0.94 (t, *J* = 7.6 Hz, 3H); ^{13}C NMR ($CDCl_3$, 101 MHz): δ_C 171.5, 137.0, 131.9, 121.3, 116.7, 37.5, 27.6, 22.3, 13.8; HRMS (ESI-TOF, *m/z*): calcd for $C_{11}H_{15}BrNO$ $[M + H]^+$, 256.0332; found, 256.0330.

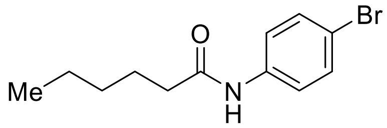

***N*-(4-Bromophenyl)hexanamide (3ab)**[9]

To a 10 mL Schlenk tube equipped with a magnetic stir bar was added carboxylic acid **2h** (116.1 mg, 1.0 mmol, 2.0 equiv), isocyanide **1a** (90.5 mg, 0.5 mmol, 1.0 equiv), acetone (5.0 mL), H_2O (250 µL), Ag_2CO_3 (27.6 mg, 0.10 mmol, 0.2 equiv). The reaction mixture was stirred under air at 60 °C in an oil bath for about 8 h. The resulting mixture was concentrated and the residue was taken up in ethyl acetate. The organic layer was washed with brine, dried over Na_2SO_4 and concentrated. Purification of the crude product by column chromatography (silica gel; petroleum ether/ethyl acetate = 12:1) afforded **3ab** in 65% yield (87 mg).

Faint white solid; mp 102–104 °C; ^{1}H NMR ($CDCl_3$, 400 MHz): δ_H 7.44–7.40 (m, 4H), 7.32 (s, 1H), 2.34 (t, *J* = 7.6 Hz, 2H), 1.75–1.66 (m, 2H), 1.36–1.33 (m, 4H), 0.92–0.89 (m, 3H); ^{13}C NMR ($CDCl_3$, 101 MHz): δ_C 171.5, 137.0, 131.9, 121.3, 116.7, 37.7, 31.4, 25.2, 22.4, 13.9; HRMS (ESI-TOF, *m/z*): calcd for $C_{12}H_{17}BrNO$ [M + H]$^+$, 270.0488; found, 270.0485.

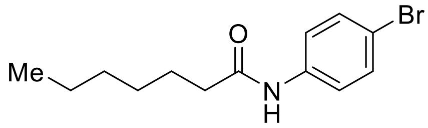

***N*-(4-Bromophenyl)heptanamide (3ac)**

To a 10 mL Schlenk tube equipped with a magnetic stir bar was added carboxylic acid **2i** (130.1 mg, 1.0 mmol, 2.0 equiv), isocyanide **1a** (90.5 mg, 0.5 mmol, 1.0 equiv), acetone (5.0 mL), H_2O (250 µL), Ag_2CO_3 (27.6 mg, 0.10 mmol, 0.2 equiv). The reaction mixture was stirred under air at 60 °C in an oil bath for about 8 h. The resulting mixture was concentrated and the residue was taken up in ethyl acetate. The organic layer was washed with brine, dried over Na_2SO_4 and concentrated. Purification of the crude product by column chromatography (silica gel; petroleum ether/ethyl acetate = 12:1) afforded **3ac** in 69% yield (98 mg).

Faint white solid; mp 91–93 °C; ^{1}H NMR ($CDCl_3$, 400 MHz): δ_H 7.44–7.40 (m, 4H), 7.31 (s, 1H), 2.34 (t, *J* = 7.2 Hz, 2H), 1.74–1.65 (m, 2H), 1.38–1.31 (m, 6H), 0.90–0.87 (m, 3H); ^{13}C NMR ($CDCl_3$, 101 MHz): δ_C

171.5, 137.0, 131.9, 121.3, 116.7, 37.8, 31.5, 28.9, 25.5, 22.5, 14.0; HRMS (ESI-TOF, *m/z*): calcd for $C_{13}H_{19}BrNO$ [M + H]$^+$, 284.0645; found, 284.0644.

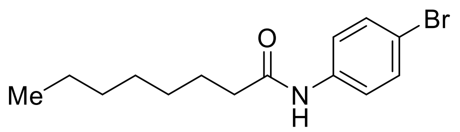

N-(4-Bromophenyl)octanamide (3ad)[10]

To a 10 mL Schlenk tube equipped with a magnetic stir bar was added carboxylic acid **2j** (144.1 mg, 1.0 mmol, 2.0 equiv), isocyanide **1a** (90.5 mg, 0.5 mmol, 1.0 equiv), acetone (5.0 mL), H_2O (250 µL), Ag_2CO_3 (27.6 mg, 0.10 mmol, 0.2 equiv). The reaction mixture was stirred under air at 60 °C in an oil bath for about 8 h. The resulting mixture was concentrated and the residue was taken up in ethyl acetate. The organic layer was washed with brine, dried over Na_2SO_4 and concentrated. Purification of the crude product by column chromatography (silica gel; petroleum ether/ethyl acetate = 12:1) afforded **3ad** in 62% yield (92 mg).

Faint white solid; mp 96–98 °C; ^{1}H NMR ($CDCl_3$, 400 MHz): δ_H 7.44–7.40 (m, 4H), 7.28 (s, 1H), 2.34 (t, *J* = 7.6 Hz, 2H), 1.73–1.67 (m, 2H), 1.36–1.27 (m, 8H), 0.90–0.87 (m, 3H); ^{13}C NMR ($CDCl_3$, 101 MHz): δ_C 171.5, 137.0, 131.9, 121.3, 116.7, 37.8, 31.6, 29.2, 29.0, 25.5, 22.6, 14.1; HRMS (ESI-TOF, *m/z*): calcd for $C_{14}H_{21}BrNO$ [M + H]$^+$, 298.0801; found, 298.0800.

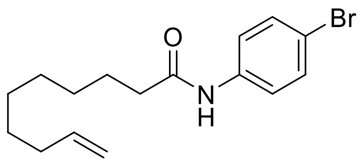

N-(4-Bromophenyl)dec-9-enamide (3ae)

To a 10 mL Schlenk tube equipped with a magnetic stir bar was added carboxylic acid **2k** (170.1 mg, 1.0 mmol, 2.0 equiv), isocyanide **1a** (90.5 mg, 0.5 mmol, 1.0 equiv), acetone (5.0 mL), H_2O (250 µL), Ag_2CO_3 (27.6 mg, 0.10 mmol, 0.2 equiv). The reaction mixture was stirred under air at 60 °C in an oil bath for about 8 h. The resulting mixture was concentrated and the residue was taken up in ethyl acetate. The organic layer was washed with brine, dried over Na_2SO_4 and concentrated. Purification of the crude product by column chromatography (silica gel; petroleum ether/ethyl acetate = 15:1) afforded **3ae** in 73% yield (118 mg).

Faint white solid; mp 79–81 °C; ^{1}H NMR ($CDCl_3$, 400 MHz): δ_H 7.42 (s, 4H), 7.26 (s, 1H), 5.85–5.75 (m, 1H), 5.01–4.92 (m, 2H), 2.37–2.32 (m, 2H), 2.06–2.01 (m, 2H), 1.73–1.67 (m, 2H), 1.39–1.31 (m, 8H); ^{13}C NMR ($CDCl_3$, 101 MHz): δ_C 171.4, 139.1, 137.0, 131.9, 121.3, 116.7, 114.2, 37.7, 33.7, 29.2, 28.9, 28.8, 25.5; HRMS (ESI-TOF, *m/z*): calcd for $C_{16}H_{23}BrNO$ $[M + H]^+$, 324.0958; found, 324.0975.

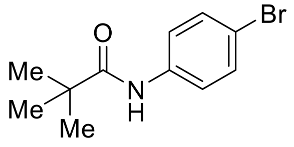

***N*-(4-Bromophenyl)pivalamide (3af)**[11]

To a 10 mL Schlenk tube equipped with a magnetic stir bar was added carboxylic acid **2l** (102.1 mg, 1.0 mmol, 2.0 equiv), isocyanide **1a** (90.5 mg, 0.5 mmol, 1.0 equiv), acetone (5.0 mL), H_2O (250 µL), Ag_2CO_3 (27.6 mg, 0.10 mmol, 0.2 equiv). The reaction mixture was stirred under air at 60 °C in an oil bath for about 8 h. The resulting mixture was concentrated and the residue was taken up in ethyl acetate. The organic layer was washed with brine, dried over Na_2SO_4 and concentrated. Purification of the crude product by column chromatography (silica gel; petroleum ether/ethyl acetate = 12:1) afforded **3af** in 78% yield (100 mg).

Faint white solid; mp 95–97 °C; ^{1}H NMR ($CDCl_3$, 400 MHz): δ_H 7.45–7.40 (m, 4H), 7.35 (s, 1H), 1.31 (s, 9H); ^{13}C NMR ($CDCl_3$, 101 MHz): δ_C 176.6, 137.1, 131.8, 121.6, 116.7, 39.6, 27.5; HRMS (ESI-TOF, *m/z*): calcd for $C_{11}H_{15}BrNO$ $[M + H]^+$, 256.0332; found, 256.0330.

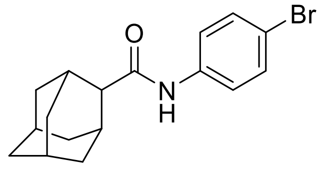

(1*r*,3*r*,5*r*,7*r*)-*N*-(4-Bromophenyl)adamantane-2-carboxamide (3ag)[6]

To a 10 mL Schlenk tube equipped with a magnetic stir bar was added carboxylic acid **2m** (180.1 mg, 1.0 mmol, 2.0 equiv), isocyanide **1a** (90.5 mg, 0.5 mmol, 1.0 equiv), acetone (5.0 mL), H_2O (250 µL), Ag_2CO_3 (27.6 mg, 0.10 mmol, 0.2 equiv). The reaction mixture was stirred under air at 60 °C in an oil bath for about 8 h. The resulting mixture was concentrated and the residue was taken up in ethyl acetate. The organic layer was washed with brine, dried over Na_2SO_4 and concentrated. Purification

of the crude product by column chromatography (silica gel; petroleum ether/ethyl acetate = 10:1) afforded **3ag** in 82% yield (137 mg).

Faint white solid; mp 311–313 °C; ^{1}H NMR ($CDCl_3$, 400 MHz): δ_H 7.46–7.40 (m, 4H), 7.29 (s, 1H), 2.10 (s, 3H), 1.96 (s, 6H), 1.80–1.72 (m, 6H); ^{13}C NMR ($CDCl_3$, 101 MHz): δ_C 176.1, 137.1, 131.8, 121.5, 116.6, 41.5, 39.2, 36.4, 28.1; HRMS (ESI-TOF, *m/z*): calcd for $C_{17}H_{21}BrNO$ $[M + H]^+$, 334.0801; found, 334.0807.

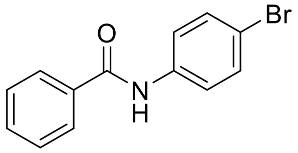

***N*-(4-Bromophenyl)benzamide (3ah)**[10]

To a 10 mL Schlenk tube equipped with a magnetic stir bar was added carboxylic acid **2n** (122.0 mg, 1.0 mmol, 2.0 equiv), isocyanide **1a** (90.5 mg, 0.5 mmol, 1.0 equiv), acetone (5.0 mL), H_2O (250 µL), Ag_2CO_3 (27.6 mg, 0.10 mmol, 0.2 equiv). The reaction mixture was stirred under air at 60 °C in an oil bath for about 8 h. The resulting mixture was concentrated and the residue was taken up in ethyl acetate. The organic layer was washed with brine, dried over Na_2SO_4 and concentrated. Purification of the crude product by column chromatography (silica gel; petroleum ether/ethyl acetate = 10:1) afforded **3ah** in 81% yield (111 mg).

Faint white solid; mp 196–198 °C; ^{1}H NMR (DMSO-d_6, 400 MHz): δ_H 10.35 (s, 1H), 7.95 (d, J = 7.6 Hz, 2H), 7.78 (d, J = 8.8 Hz, 2H), 7.62–7.58 (m, 1H), 7.55–7.52 (m, 4H); ^{13}C NMR (DMSO-d_6, 101 MHz): δ_C 165.6, 138.6, 134.7, 131.7, 131.4, 128.4, 127.7, 122.2, 115.3; HRMS (ESI-TOF, *m/z*): calcd for $C_{13}H_{11}BrNO$ $[M + H]^+$, 276.0019; found, 276.0033.

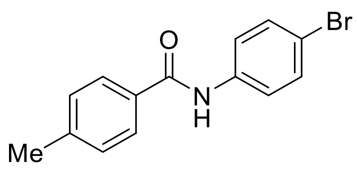

N-(4-Bromophenyl)-4-methylbenzamide (3ai)[12]

To a 10 mL Schlenk tube equipped with a magnetic stir bar was added carboxylic acid **2o** (136.1 mg, 1.0 mmol, 2.0 equiv), isocyanide **1a** (90.5 mg, 0.5 mmol, 1.0 equiv), acetone (5.0 mL), H_2O (250 μL), Ag_2CO_3 (27.6 mg, 0.10 mmol, 0.2 equiv). The reaction mixture was stirred under air at 60 °C in an oil bath for about 8 h. The resulting mixture was concentrated and the residue was taken up in ethyl acetate. The organic layer was washed with brine, dried over Na_2SO_4 and concentrated. Purification of the crude product by column chromatography (silica gel; petroleum ether/ethyl acetate = 12:1) afforded **3ai** in 84% yield (121 mg).

Faint white solid; mp 185–186 °C; ^{1}H NMR (DMSO-d_6, 400 MHz): δ_H 10.28 (s, 1H), 7.87 (d, *J* = 7.6 Hz, 2H), 7.77 (d, *J* = 8.4 Hz, 2H), 7.53 (d, *J* = 8.4 Hz, 2H), 7.37 (d, *J* = 7.6 Hz, 2H), 2.38 (s, 3H); ^{13}C NMR (DMSO-d_6, 101 MHz): δ_C 165.4, 141.7, 138.6, 131.8, 131.4, 128.9, 127.7, 122.2, 115.2, 21.0; HRMS (ESI-TOF, *m/z*): calcd for $C_{14}H_{13}BrNO$ [M + H]$^+$, 290.0175; found, 290.0176.

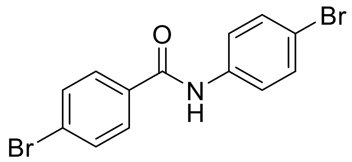

4-Bromo-N-(4-bromophenyl)benzamide (3aj)[12]

To a 10 mL Schlenk tube equipped with a magnetic stir bar was added carboxylic acid **2p** (199.9 mg, 1.0 mmol, 2.0 equiv), isocyanide **1a** (90.5 mg, 0.5 mmol, 1.0 equiv), acetone (5.0 mL), H_2O (250 μL), Ag_2CO_3 (27.6 mg, 0.10 mmol, 0.2 equiv). The reaction mixture was stirred under air at 60 °C in an oil bath for about 8 h. The resulting mixture was concentrated and the residue was taken up in ethyl acetate. The organic layer was washed with brine, dried over Na_2SO_4 and concentrated. Purification of the crude product by column chromatography (silica gel; petroleum ether/ethyl acetate = 10:1) afforded **3aj** in 75% yield (132 mg).

Faint white solid; mp 234–235 °C; ^{1}H NMR ($CDCl_3$, 400 MHz): δ_H 7.79 (s, 1H), 7.73 (d, *J* = 8.0 Hz, 2H), 7.64 (d, *J* = 8.4 Hz, 2H), 7.55–7.48 (m, 4H); ^{13}C NMR ($CDCl_3$, 101 MHz): δ_C 164.7, 136.7, 133.4, 132.1,

128.6, 126.9, 122.8, 121.8, 117.5; HRMS (ESI-TOF, *m/z*): calcd for $C_{13}H_{10}Br_2NO$ $[M + H]^+$, 353.9124; found, 353.9137.

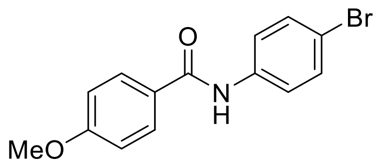

_N_-(4-Bromophenyl)-4-methoxybenzamide (3ak)[10]

To a 10 mL Schlenk tube equipped with a magnetic stir bar was added carboxylic acid **2q** (152.0 mg, 1.0 mmol, 2.0 equiv), isocyanide **1a** (90.5 mg, 0.5 mmol, 1.0 equiv), acetone (5.0 mL), H_2O (250 µL), Ag_2CO_3 (27.6 mg, 0.10 mmol, 0.2 equiv). The reaction mixture was stirred under air at 60 °C in an oil bath for about 8 h. The resulting mixture was concentrated and the residue was taken up in ethyl acetate. The organic layer was washed with brine, dried over Na_2SO_4 and concentrated. Purification of the crude product by column chromatography (silica gel; petroleum ether/ethyl acetate = 9:1) afforded **3ak** in 87% yield (137 mg).

Faint white solid; mp 182–184 °C; ^{1}H NMR (DMSO-*d*$_6$, 400 MHz): δ_H 10.20 (s, 1H), 8.07 (d, *J* = 8.0 Hz, 2H), 7.95 (d, *J* = 8.4 Hz, 1H), 7.76 (d, *J* = 8.4 Hz, 1H), 7.52 (d, *J* = 8.0 Hz, 1H), 7.14 (d, *J* = 8.0 Hz, 2H), 7.06 (d, *J* = 8.0 Hz, 1H), 3.88 (s, 3H); ^{13}C NMR (DMSO-*d*$_6$, 101 MHz): δ_C 165.5, 162.0, 138.8, 131.4, 129.6, 126.7, 122.2, 115.0, 113.6, 55.4; HRMS (ESI-TOF, *m/z*): calcd for $C_{14}H_{13}BrNO_2$ $[M + H]^+$, 306.0124; found, 306.0142.

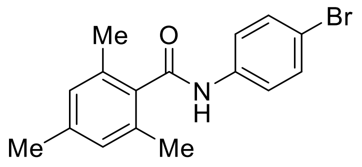

_N_-(4-Bromophenyl)-2,4,6-trimethylbenzamide (3al)

To a 10 mL Schlenk tube equipped with a magnetic stir bar was added carboxylic acid **2r** (164.1 mg, 1.0 mmol, 2.0 equiv), isocyanide **1a** (90.5 mg, 0.5 mmol, 1.0 equiv), acetone (5.0 mL), H_2O (250 µL), Ag_2CO_3 (27.6 mg, 0.10 mmol, 0.2 equiv). The reaction mixture was stirred under air at 60 °C in an oil bath for about 8 h. The resulting mixture was concentrated and the residue was taken up in ethyl acetate. The organic layer was washed with brine, dried over Na_2SO_4 and concentrated. Purification

of the crude product by column chromatography (silica gel; petroleum ether/ethyl acetate = 10:1) afforded **3al** in 68% yield (108 mg).

Faint white solid; mp 217–218 °C; ^{1}H NMR ($CDCl_3$, 400 MHz): δ_H 7.50 (d, *J* = 8.4 Hz, 2H), 7.46–7.43 (m, 3H), 6.87–6.86 (m, 2H), 2.32–2.27 (m, 9H); ^{13}C NMR ($CDCl_3$, 101 MHz): δ_C 168.9, 139.1, 136.9, 134.6, 134.2, 132.0, 128.6, 128.4, 121.3, 117.1, 21.1, 20.1, 19.1; HRMS (ESI-TOF, *m/z*): calcd for $C_{16}H_{17}BrNO$ $[M + H]^+$, 318.0488; found, 318.0495.

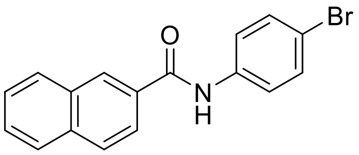

***N*-(4-Bromophenyl)-2-naphthamide (3am)**[12]

To a 10 mL Schlenk tube equipped with a magnetic stir bar was added carboxylic acid **2s** (172.1 mg, 1.0 mmol, 2.0 equiv), isocyanide **1a** (90.5 mg, 0.5 mmol, 1.0 equiv), acetone (5.0 mL), H_2O (250 µL), Ag_2CO_3 (27.6 mg, 0.10 mmol, 0.2 equiv). The reaction mixture was stirred under air at 60 °C in an oil bath for about 8 h. The resulting mixture was concentrated and the residue was taken up in ethyl acetate. The organic layer was washed with brine, dried over Na_2SO_4 and concentrated. Purification of the crude product by column chromatography (silica gel; petroleum ether/ethyl acetate = 13:1) afforded **3am** in 83% yield (135 mg).

Faint white solid; mp 221–223 °C; ^{1}H NMR (DMSO-*d*$_6$, 400 MHz): δ_H 9.71 (s, 1H), 7.73 (s, 1H), 7.25–7.15 (m, 4H), 6.97 (d, *J* = 8.0 Hz, 2H), 6.82–6.75 (m, 2H), 6.71 (d, *J* = 8.0 Hz, 2H); ^{13}C NMR (DMSO-*d*$_6$, 101 MHz): δ_C 165.7, 138.6, 134.3, 132.03, 131.99, 131.5, 129.0, 128.1, 127.9, 127.7, 126.9, 124.4, 122.2, 115.3; HRMS (ESI-TOF, *m/z*): calcd for $C_{17}H_{13}BrNO$ $[M + H]^+$, 326.0175; found, 326.0174.

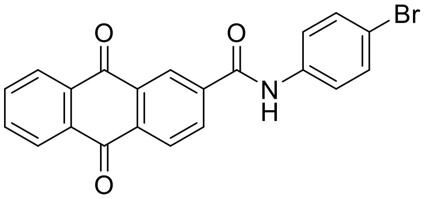

***N*-(4-Bromophenyl)-9,10-dioxo-8a,9,10,10a-tetrahydroanthracene-2-carboxamide (3an)**

To a 10 mL Schlenk tube equipped with a magnetic stir bar was added carboxylic acid **2t** (252.0 mg, 1.0 mmol, 2.0 equiv), isocyanide **1a** (90.5 mg, 0.5 mmol, 1.0 equiv), acetone (5.0 mL), H_2O (250 µL), Ag_2CO_3 (27.6 mg, 0.10 mmol, 0.2 equiv). The reaction mixture was stirred under air at 60 °C in an oil

bath for about 8 h. The resulting mixture was concentrated and the residue was taken up in ethyl acetate. The organic layer was washed with brine, dried over Na_2SO_4 and concentrated. Purification of the crude product by column chromatography (silica gel; petroleum ether/ethyl acetate = 10:1) afforded **3an** in 88% yield (179 mg).

Faint white solid; mp 265–267 °C; ^{1}H NMR (DMSO-d_6, 400 MHz): δ_H 10.84 (s, 1H), 8.73 (s, 1H), 8.43 (d, *J* = 8.0 Hz, 1H), 8.32 (d, *J* = 8.0 Hz, 1H), 8.26–8.22 (m, 2H), 7.97–7.92 (m, 2H), 7.81 (d, *J* = 8.4 Hz, 2H), 7.57 (d, *J* = 8.4 Hz, 2H); ^{13}C NMR (DMSO-d_6, 101 MHz): δ_C 182.1, 164.0, 139.4, 138.2, 134.8, 134.7, 134.5, 133.3, 133.1, 133.0, 131.5, 127.1, 126.9, 126.8, 126.0, 122.4, 115.9; HRMS (ESI-TOF, *m/z*): calcd for $C_{21}H_{13}BrNO_3$ [M + H]$^+$, 406.0073; found, 406.0089.

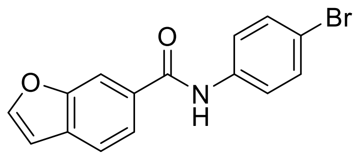

***N*-(4-Bromophenyl)benzofuran-6-carboxamide (3ao)**

To a 10 mL Schlenk tube equipped with a magnetic stir bar was added carboxylic acid **2u** (162.0 mg, 1.0 mmol, 2.0 equiv), isocyanide **1a** (90.5 mg, 0.5 mmol, 1.0 equiv), acetone (5.0 mL), H_2O (250 µL), Ag_2CO_3 (27.6 mg, 0.10 mmol, 0.2 equiv). The reaction mixture was stirred under air at 60 °C in an oil bath for about 8 h. The resulting mixture was concentrated and the residue was taken up in ethyl acetate. The organic layer was washed with brine, dried over Na_2SO_4 and concentrated. Purification of the crude product by column chromatography (silica gel; petroleum ether/ethyl acetate = 12:1) afforded **3ao** in 77% yield (121 mg).

Faint white solid; mp 177–179 °C; ^{1}H NMR (DMSO-d_6, 400 MHz): δ_H 10.39 (s, 1H), 8.21 (d, *J* = 22.4 Hz, 2H), 7.89 (d, *J* = 8.4 Hz, 1H), 7.80 (d, *J* = 7.6 Hz, 3H), 7.55 (d, *J* = 8.0 Hz, 2H), 7.08 (s, 1H); ^{13}C NMR (DMSO-d_6, 101 MHz): δ_C 165.4, 153.8, 148.7, 138.6, 131.4, 130.8, 130.3, 122.7, 122.2, 121.1, 115.3, 110.8, 106.9; HRMS (ESI-TOF, *m/z*): calcd for $C_{15}H_{11}BrNO_2$ [M + H]$^+$, 315.9968; found, 315.9975.

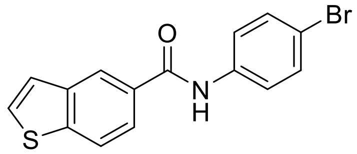

***N*-(4-Bromophenyl)benzo[b]thiophene-5-carboxamide (3ap)**

To a 10 mL Schlenk tube equipped with a magnetic stir bar was added carboxylic acid **2v** (178.0 mg, 1.0 mmol, 2.0 equiv), isocyanide **1a** (90.5 mg, 0.5 mmol, 1.0 equiv), acetone (5.0 mL), H_2O (250 µL), Ag_2CO_3 (27.6 mg, 0.10 mmol, 0.2 equiv). The reaction mixture was stirred under air at 60 °C in an oil bath for about 8 h. The resulting mixture was concentrated and the residue was taken up in ethyl acetate. The organic layer was washed with brine, dried over Na_2SO_4 and concentrated. Purification of the crude product by column chromatography (silica gel; petroleum ether/ethyl acetate = 12:1) afforded **3ap** in 64% yield (106 mg).

Faint white solid; mp 206–207 °C; ^1H NMR (DMSO-d_6, 400 MHz): δ_H 10.40 (s, 1H), 8.21 (d, J = 18.8 Hz, 2H), 7.89 (d, J = 8.0 Hz, 1H), 7.80 (d, J = 8.4 Hz, 3H), 7.55 (d, J = 8.0 Hz, 2H), 7.08 (s, 1H); ^{13}C NMR (DMSO-d_6, 101 MHz): δ_C 165.4, 153.8, 148.7, 138.6, 131.4, 130.8, 130.3, 122.6, 122.2, 121.1, 115.3, 110.8, 106.9; HRMS (ESI-TOF, *m/z*): calcd for $C_{15}H_{11}BrNOS$ [M + H]$^+$, 331.9739; found, 331.9743.

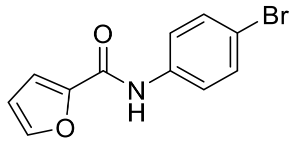

***N*-(4-Bromophenyl)furan-2-carboxamide (3aq)**[10]

To a 10 mL Schlenk tube equipped with a magnetic stir bar was added carboxylic acid **2w** (112.0 mg, 1.0 mmol, 2.0 equiv), isocyanide **1a** (90.5 mg, 0.5 mmol, 1.0 equiv), acetone (5.0 mL), H_2O (250 µL), Ag_2CO_3 (27.6 mg, 0.10 mmol, 0.2 equiv). The reaction mixture was stirred under air at 60 °C in an oil bath for about 8 h. The resulting mixture was concentrated and the residue was taken up in ethyl acetate. The organic layer was washed with brine, dried over Na_2SO_4 and concentrated. Purification of the crude product by column chromatography (silica gel; petroleum ether/ethyl acetate = 15:1) afforded **3aq** in 82% yield (109 mg).

Faint white solid; mp 142–144 °C; ^1H NMR ($CDCl_3$, 400 MHz): δ_H 8.09 (s, 1H), 7.56 (d, J = 8.4 Hz, 2H), 7.51–7.46 (m, 3H), 7.24 (d, J = 2..8 Hz, 1H), 6.57 (s, 1H); ^{13}C NMR $CDCl_3$, 101 MHz): δ_C 156.0, 147.5,

144.3, 136.4, 132.0, 121.4, 117.1, 115.6, 112.7; HRMS (ESI-TOF, *m/z*): calcd for $C_{11}H_7BrNO_2$ $[M - H]^-$, 263.9666; found, 263.9679.

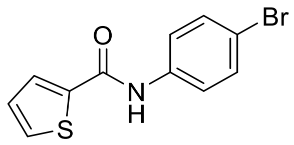

***N*-(4-Bromophenyl)thiophene-2-carboxamide (3ar)**[12]

To a 10 mL Schlenk tube equipped with a magnetic stir bar was added carboxylic acid **2x** (128.0 mg, 1.0 mmol, 2.0 equiv), isocyanide **1a** (90.5 mg, 0.5 mmol, 1.0 equiv), acetone (5.0 mL), H_2O (250 µL), Ag_2CO_3 (27.6 mg, 0.10 mmol, 0.2 equiv). The reaction mixture was stirred under air at 60 °C in an oil bath for about 8 h. The resulting mixture was concentrated and the residue was taken up in ethyl acetate. The organic layer was washed with brine, dried over Na_2SO_4 and concentrated. Purification of the crude product by column chromatography (silica gel; petroleum ether/ethyl acetate = 15:1) afforded **3ar** in 69% yield (97 mg).

Faint white solid; mp 218–219 °C; ^{1}H NMR (DMSO-d_6, 400 MHz): δ_H 10.34 (s, 1H), 8.02 (d, *J* = 1.8 Hz, 1H), 7.88 (d, *J* = 5.2 Hz, 1H), 7.71 (d, *J* = 8.4 Hz, 2H), 7.54 (d, *J* = 8.8 Hz, 2H), 7.24–7.22 (m, 1H); ^{13}C NMR (DMSO-d_6, 101 MHz): δ_C 159.9, 139.7, 138.1, 132.2, 131.5, 129.4, 128.1, 122.2, 115.4; HRMS (ESI-TOF, *m/z*): calcd for $C_{11}H_7BrNOS$ $[M - H]^-$, 279.9437; found, 279.9438.

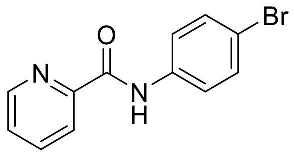

***N*-(4-Bromophenyl)picolinamide (3as)**[13]

To a 10 mL Schlenk tube equipped with a magnetic stir bar was added carboxylic acid **2y** (123.0 mg, 1.0 mmol, 2.0 equiv), isocyanide **1a** (90.5 mg, 0.5 mmol, 1.0 equiv), acetone (5.0 mL), H_2O (250 µL), Ag_2CO_3 (27.6 mg, 0.10 mmol, 0.2 equiv). The reaction mixture was stirred under air at 60 °C in an oil bath for about 8 h. The resulting mixture was concentrated and the residue was taken up in ethyl acetate. The organic layer was washed with brine, dried over Na_2SO_4 and concentrated. Purification of the crude product by column chromatography (silica gel; petroleum ether/ethyl acetate = 15:1) afforded **3as** in 63% yield (87 mg).

Faint white solid; mp 148–149 °C; ^{1}H NMR ($CDCl_3$, 400 MHz): δ_H 10.04 (s, 1H), 8.61 (d, J = 4.4 Hz, 1H), 8.28 (d, J = 7.6 Hz, 1H), 7.93–7.89 (m, 1H), 7.69 (d, J = 8.4 Hz, 2H), 7.49 (d, J = 8.8 Hz, 3H); ^{13}C NMR ($CDCl_3$, 101 MHz): δ_C 162.0, 149.4, 147.9, 137.7, 136.8, 132.0, 126.6, 122.4, 121.1, 116.8; HRMS (ESI-TOF, *m/z*): calcd for $C_{12}H_{10}BrN_2O$ [M + H]$^+$, 276.9971; found, 276.9968.

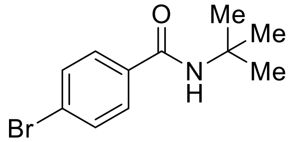

4-Bromo-*N*-(*tert*-butyl)benzamide (3at)

To a 10 mL Schlenk tube equipped with a magnetic stir bar was added carboxylic acid **2p** (119.9 mg, 1.0 mmol, 2.0 equiv), isocyanide **1v** (41.5 mg, 0.5 mmol, 1.0 equiv), acetone (5.0 mL), H_2O (250 µL), Ag_2CO_3 (27.6 mg, 0.10 mmol, 0.2 equiv). The reaction mixture was stirred under air at 60 °C in an oil bath for about 8 h. The resulting mixture was concentrated and the residue was taken up in ethyl acetate. The organic layer was washed with brine, dried over Na_2SO_4 and concentrated. Purification of the crude product by column chromatography (silica gel; petroleum ether/ethyl acetate = 12:1) afforded **3at** in 74% yield (94 mg).

Faint white solid; mp 173–175 °C; ^{1}H NMR ($CDCl_3$, 400 MHz): δ_H 7.59 (d, J = 8.0 Hz, 2H), 7.54 (d, J = 8.0 Hz, 2H), 5.91 (s, 1H), 1.47 (s, 9H); ^{13}C NMR ($CDCl_3$, 101 MHz): δ_C 165.9, 134.7, 132.4, 131.9, 131.6, 128.3, 125.6, 51.8, 28.8; HRMS (ESI-TOF, *m/z*): calcd for $C_{11}H_{15}BrNO$ [M + H]$^+$, 256.0332; found, 256.0330.

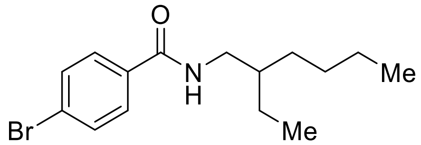

4-Bromo-*N*-(2-ethylhexyl)benzamide (3au)

To a 10 mL Schlenk tube equipped with a magnetic stir bar was added carboxylic acid **2p** (120 mg, 1.0 mmol, 2.0 equiv), isocyanide **1w** (70 mg, 0.5 mmol, 1.0 equiv), acetone (5.0 mL), H_2O (250 µL), Ag_2CO_3 (27.6 mg, 0.10 mmol, 0.2 equiv). The reaction mixture was stirred under air at 60 °C in an oil bath for about 8 h. The resulting mixture was concentrated and the residue was taken up in ethyl acetate. The organic layer was washed with brine, dried over Na_2SO_4 and concentrated. Purification of the crude

product by column chromatography (silica gel; petroleum ether/ethyl acetate = 15:1) afforded **3au** in 74% yield (115 mg).

Faint white solid; mp 81-82 °C; ^{1}H NMR ($CDCl_3$, 400 MHz): δ_H 7.62 (d, *J* = 8.0 Hz, 2H), 7.56–7.54 (m, 2H), 6.14 (s, 1H), 3.38 (s, 2H), 1.42–1.30 (m, 9H), 0.95–0.88 (m, 6H); ^{13}C NMR ($CDCl_3$, 101 MHz): δ_C 166.6, 133.7, 131.7, 128.4, 125.9, 43.0, 39.4, 31.1, 28.9, 24.3, 23.0, 14.1, 10.9; HRMS (ESI-TOF, *m/z*): $C_{15}H_{23}BrNO$ [M + H]$^+$, 312.0958; found, 312.0958.

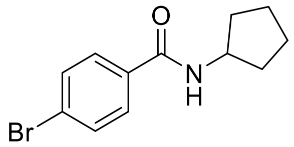

4-Bromo-*N*-cyclopentylbenzamide (3av)[14]

To a 10 mL Schlenk tube equipped with a magnetic stir bar was added **2p** (119.9 mg, 1.0 mmol, 2.0 equiv), isocyanide **1x** (47.5 mg, 0.5 mmol, 1.0 equiv), acetone (5.0 mL), H_2O (250 µL), Ag_2CO_3 (27.6 mg, 0.10 mmol, 0.2 equiv). The reaction mixture was stirred under air at 60 °C in an oil bath for about 8 h. The resulting mixture was concentrated and the residue was taken up in ethyl acetate. The organic layer was washed with brine, dried over Na_2SO_4 and concentrated. Purification of the crude product by column chromatography (silica gel; petroleum ether/ethyl acetate = 15:1) afforded **3av** in 69% yield (92 mg).

Faint white solid; mp 156–157 °C; ^{1}H NMR ($CDCl_3$, 400 MHz): δ_H 7.62 (d, *J* = 7.6 Hz, 2H), 7.54 (d, *J* = 7.2 Hz, 2H), 6.11 (s, 1H), 4.42–4.33 (m, 1H), 2.13–2.07 (m, 2H), 1.75–1.63 (m, 3H), 1.51–1.46 (m, 2H), 1.42–1.38 (m, 1H); ^{13}C NMR ($CDCl_3$, 101 MHz): δ_C 166.1, 133.7, 131.7, 128.5, 125.8, 51.8, 45.7, 33.1, 23.8, 8.6; HRMS (ESI-TOF, *m/z*): calcd for $C_{12}H_{15}BrNO$ [M + H]$^+$, 268.0332; found, 268.0346.

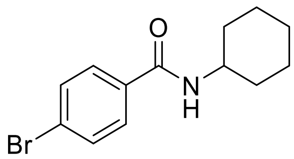

4-Bromo-*N*-cyclohexylbenzamide (3aw)[15]

To a 10 mL Schlenk tube equipped with a magnetic stir bar was added **2p** (119.9 mg, 1.0 mmol, 2.0 equiv), isocyanide **1y** (54.5 mg, 0.5 mmol, 1.0 equiv), acetone (5.0 mL), H_2O (250 µL), Ag_2CO_3 (27.6 mg, 0.10 mmol, 0.2 equiv). The reaction mixture was stirred under air at 60 °C in an oil bath for about 8 h. The resulting mixture was concentrated and the residue was taken up in ethyl acetate. The organic layer was washed with brine, dried over Na_2SO_4 and concentrated. Purification of the crude product by column chromatography (silica gel; petroleum ether/ethyl acetate = 15:1) afforded **3aw** in 81% yield (114 mg).

Faint white solid; mp 169–170 °C; ^{1}H NMR ($CDCl_3$, 400 MHz): δ_H 7.62 (d, *J* = 7.6 Hz, 2H), 7.54 (d, *J* = 7.6 Hz, 2H), 6.09 (s, 1H), 3.99–3.90 (m, 1H), 2.03–1.99 (m, 2H), 1.77–1.73 (m, 2H), 1.67–1.64 (m, 1H), 1.45–1.36 (m, 2H), 1.27–1.14 (m, 3H); ^{13}C NMR ($CDCl_3$, 101 MHz): δ_C 165.6, 133.8, 132.3, 131.9, 131.6, 128.5, 125.8, 48.8, 33.1, 25.5, 24.9; HRMS (ESI-TOF, *m/z*): calcd for $C_{13}H_{17}BrNO$ $[M + H]^+$, 282.0488; found, 282.0497.

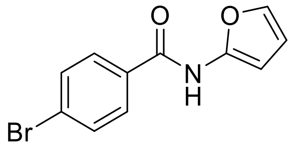

4-Bromo-*N*-(furan-2-yl)benzamide (3ax)

To a 10 mL Schlenk tube equipped with a magnetic stir bar was added carboxylic acid **2p** (119.9 mg, 1.0 mmol, 2.0 equiv), isocyanide **1z** (46.5 mg, 0.5 mmol, 1.0 equiv), acetone (5.0 mL), H_2O (250 µL), Ag_2CO_3 (27.6 mg, 0.10 mmol, 0.2 equiv). The reaction mixture was stirred under air at 60 °C in an oil bath for about 8 h. The resulting mixture was concentrated and the residue was taken up in ethyl acetate. The organic layer was washed with brine, dried over Na_2SO_4 and concentrated. Purification of the crude product by column chromatography (silica gel; petroleum ether/ethyl acetate = 13:1) afforded **3ax** in 66% yield (87 mg).

Faint white solid; mp 138–140 °C; ^{1}H NMR (DMSO-d_6, 400 MHz): δ_C 10.32 (s, 1H), 7.95 (s, 1H), 7.74 (d, *J* = 8.4 Hz, 2H), 7.52 (d, *J* = 8.0 Hz, 2H), 7.35 (s, 1H), 6.71 (s, 1H); ^{13}C NMR (DMSO-d_6, 101 MHz): δ_C 156.3,

147.3, 146.0, 138.0, 131.5, 122.3, 115.5, 115.1, 112.3; HRMS (ESI-TOF, *m/z*): calcd for $C_{11}H_9BrNO_2$ [M + H]$^+$, 265.9811; found, 265.9814.

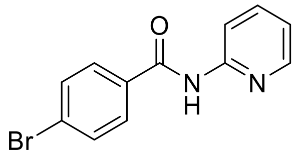

4-Bromo-*N*-(pyridin-2-yl)benzamide (3ay)[16]

To a 10 mL Schlenk tube equipped with a magnetic stir bar was added carboxylic acid **2p** (119.9 mg, 1.0 mmol, 2.0 equiv), isocyanide **1aa** (52.0 mg, 0.5 mmol, 1.0 equiv), acetone (5.0 mL), H_2O (250 μL), Ag_2CO_3 (27.6 mg, 0.10 mmol, 0.2 equiv). The reaction mixture was stirred under air at 60 °C in an oil bath for about 8 h. The resulting mixture was concentrated and the residue was taken up in ethyl acetate. The organic layer was washed with brine, dried over Na_2SO_4 and concentrated. Purification of the crude product by column chromatography (silica gel; petroleum ether/ethyl acetate = 14:1) afforded **3ay** in 73% yield (101 mg).

Faint white solid; mp 155–156 °C; ^{1}H NMR ($CDCl_3$, 400 MHz): δ_H 8.39 (d, *J* = 4.8 Hz, 1H), 7.77–7.73 (m, 1H), 7.62 (d, *J* = 8.0 Hz, 3H), 7.50 (d, *J* = 8.0 Hz, 3H), 7.21–7.18 (m, 1H); ^{13}C NMR ($CDCl_3$, 101 MHz): δ_C 172.0, 153.2, 149.5, 138.5, 133.1, 132.4, 132.0, 131.9, 130.7, 127.7, 122.5, 121.9; HRMS (ESI-TOF, *m/z*): calcd for $C_{12}H_{10}BrN_2O$ [M + H]$^+$, 276.9971; found, 276.9979.

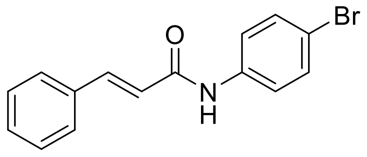

***N*-(4-Bromophenyl)cinnamamide (3az)**[12]

To a 10 mL Schlenk tube equipped with a magnetic stir bar was added carboxylic acid **2z** (148.1 mg, 1.0 mmol, 2.0 equiv), isocyanide **1a** (90.5 mg, 0.5 mmol, 1.0 equiv), acetone (5.0 mL), H_2O (250 μL), Ag_2CO_3 (27.6 mg, 0.10 mmol, 0.2 equiv). The reaction mixture was stirred under air at 60 °C in an oil bath for about 8 h. The resulting mixture was concentrated and the residue was taken up in ethyl acetate. The organic layer was washed with brine, dried over Na_2SO_4 and concentrated. Purification of the crude product by column chromatography (silica gel; petroleum ether/ethyl acetate = 10:1) afforded **3az** in 77% yield (116 mg).

Faint white solid; mp 192–194 °C; ^{1}H NMR (DMSO-d_6, 400 MHz): δ_H 10.35 (s, 1H), 7.68 (d, J = 8.4 Hz, 2H), 7.64–7.59 (m, 3H), 7.52 (d, J = 8.4 Hz, 2H), 7.47–7.41 (m, 3H), 6.81 (d, J = 16 Hz, 1H); ^{13}C NMR (DMSO-d_6, 101 MHz): δ_C 163.6, 140.6, 138.6, 134.6, 131.6, 129.9, 129.0, 127.8, 121.9, 121.1, 115.0; HRMS (ESI-TOF, *m/z*): calcd for $C_{15}H_{11}BrNO$ $[M - H]^-$, 300.0030; found, 300.0036.

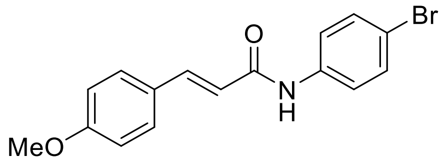

(*E*)-*N*-(4-Bromophenyl)-3-(4-methoxyphenyl)acrylamide (3ba)

To a 10 mL Schlenk tube equipped with a magnetic stir bar was added carboxylic acid **2aa** (178.1 mg, 1.0 mmol, 2.0 equiv), isocyanide **1a** (90.5 mg, 0.5 mmol, 1.0 equiv), acetone (5.0 mL), H_2O (250 µL), Ag_2CO_3 (27.6 mg, 0.10 mmol, 0.2 equiv). The reaction mixture was stirred under air at 60 °C in an oil bath for about 8 h. The resulting mixture was concentrated and the residue was taken up in ethyl acetate. The organic layer was washed with brine, dried over Na_2SO_4 and concentrated. Purification of the crude product by column chromatography (silica gel; petroleum ether/ethyl acetate = 8:1) afforded **3ba** in 83% yield (137 mg).

Faint white solid; mp 180–182 °C; ^{1}H NMR (CDCl$_3$, 400 MHz): δ_H 7.70 (d, J = 35.2 Hz, 1H), 7.53–7.43 (m, 7H), 6.90 (d, J = 8.0 Hz, 2H), 6.40 (d, J = 15.2 Hz, 1H), 3.84 (s, 3H); ^{13}C NMR (CDCl$_3$, 101 MHz): δ_C 164.3, 161.3, 142.6, 137.2, 132.0, 129.6, 127.1, 121.4, 117.8, 116.8, 114.4, 55.4; HRMS (ESI-TOF, *m/z*): calcd for $C_{16}H_{13}BrNO_2$ $[M - H]^-$, 330.0135; found, 330.0141.

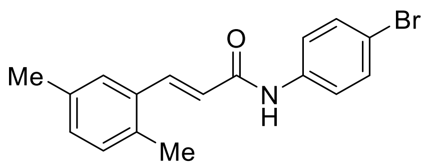

(*E*)-*N*-(4-Bromophenyl)-3-(2,5-dimethylphenyl)acrylamide (3bb)

To a 10 mL Schlenk tube equipped with a magnetic stir bar was added carboxylic acid **2ab** (176.1 mg, 1.0 mmol, 2.0 equiv), isocyanide **1a** (90.5 mg, 0.5 mmol, 1.0 equiv), acetone (5.0 mL), H_2O (250 µL), Ag_2CO_3 (27.6 mg, 0.10 mmol, 0.2 equiv). The reaction mixture was stirred under air at 60 °C in an oil bath for about 8 h. The resulting mixture was concentrated and the residue was taken up in ethyl acetate. The organic layer was washed with brine, dried over Na_2SO_4 and concentrated. Purification

of the crude product by column chromatography (silica gel; petroleum ether/ethyl acetate = 12:1) afforded **3bb** in 84% yield (138 mg).

Faint white solid; mp 178–180 °C; ^{1}H NMR ($CDCl_3$, 400 MHz): δ_H 8.03 (d, *J* = 15.2 Hz, 1H), 7.54 (d, *J* = 8.4 Hz, 2H), 7.46–7.42 (m, 3H), 7.36 (s, 1H), 7.12–7.08 (m, 2H), 6.44 (d, *J* = 15.2 Hz, 1H), 2.40 (s, 3H), 2.32 (s, 3H); ^{13}C NMR ($CDCl_3$, 101 MHz): δ_C 164.1, 140.9, 137.2, 135.6, 134.8, 133.2, 132.0, 130.8, 126.7, 121.4, 121.1, 116.9, 20.9, 19.3; HRMS (ESI-TOF, *m/z*): calcd for $C_{17}H_{17}BrNO$ [M + H]$^+$, 330.0488; found, 330.0487.

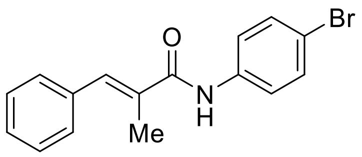

(*E*)-*N*-(4-Bromophenyl)-2-methyl-3-phenylacrylamide (3bc)

To a 10 mL Schlenk tube equipped with a magnetic stir bar was added carboxylic acid **2ac** (162.1 mg, 1.0 mmol, 2.0 equiv), isocyanide **1a** (90.5 mg, 0.5 mmol, 1.0 equiv), acetone (5.0 mL), H_2O (250 µL), Ag_2CO_3 (27.6 mg, 0.10 mmol, 0.2 equiv). The reaction mixture was stirred under air at 60 °C in an oil bath for about 8 h. The resulting mixture was concentrated and the residue was taken up in ethyl acetate. The organic layer was washed with brine, dried over Na_2SO_4 and concentrated. Purification of the crude product by column chromatography (silica gel; petroleum ether/ethyl acetate = 15:1) afforded **3bc** in 81% yield (128 mg).

Faint white solid; mp 151–153 °C; ^{1}H NMR ($CDCl_3$, 400 MHz): δ_H 7.65 (s, 1H), 7.52 (d, *J* = 8.4 Hz, 2H), 7.45 (d, *J* = 8.4 Hz, 2H), 7.40 (d, *J* = 6.4 Hz, 3H), 7.37–7.31 (m, 3H), 2.19 (s, 3H); ^{13}C NMR ($CDCl_3$, 101 MHz): δ_C 167.8, 137.1, 135.6, 134.7, 132.5, 132.0, 129.4, 128.4, 128.2, 121.7, 117.0, 14.4; HRMS (ESI-TOF, *m/z*): calcd for $C_{16}H_{13}BrNO$ [M – H]$^-$, 314.0186; found, 314.0199.

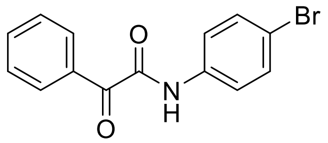

N-(4-Bromophenyl)-2-oxo-2-phenylacetamide (3bd)

To a 10 mL Schlenk tube equipped with a magnetic stir bar was added carboxylic acid **2ad** (150.0 mg, 1.0 mmol, 2.0 equiv), isocyanide **1a** (90.5 mg, 0.5 mmol, 1.0 equiv), acetone (5.0 mL), H_2O (250 µL), Ag_2CO_3 (27.6 mg, 0.10 mmol, 0.2 equiv). The reaction mixture was stirred under air at 60 °C in an oil bath for about 8 h. The resulting mixture was concentrated and the residue was taken up in ethyl acetate. The organic layer was washed with brine, dried over Na_2SO_4 and concentrated. Purification of the crude product by column chromatography (silica gel; petroleum ether/ethyl acetate = 15:1) afforded **3bd** in 68% yield (103 mg).

Faint white solid; mp 158–159 °C; ^{1}H NMR ($CDCl_3$, 400 MHz): δ_H 8.98 (s, 1H), 8.40 (d, *J* = 8.0 Hz, 2H), 7.67 (m, 1H), 7.61 (d, *J* = 8.4 Hz, 2H), 7.53–7.50 (m, 4H); ^{13}C NMR ($CDCl_3$, 101 MHz): δ_C 187.0, 158.8, 135.7, 134.8, 132.9, 132.2, 131.5, 128.6, 121.4, 118.1; HRMS (ESI-TOF, *m/z*): calcd for $C_{14}H_9BrNO_2$ [M – H]$^-$, 301.9822; found, 301.9828.

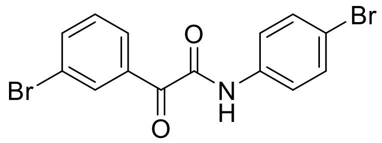

2-(3-Bromophenyl)-N-(4-bromophenyl)-2-oxoacetamide (3be)

To a 10 mL Schlenk tube equipped with a magnetic stir bar was added carboxylic acid **2ae** (227.9 mg, 1.0 mmol, 2.0 equiv), isocyanide **1a** (90.5 mg, 0.5 mmol, 1.0 equiv), acetone (5.0 mL), H_2O (250 µL), Ag_2CO_3 (27.6 mg, 0.10 mmol, 0.2 equiv). The reaction mixture was stirred under air at 60 °C in an oil bath for about 8 h. The resulting mixture was concentrated and the residue was taken up in ethyl acetate. The organic layer was washed with brine, dried over Na_2SO_4 and concentrated. Purification of the crude product by column chromatography (silica gel; petroleum ether/ethyl acetate = 15:1) afforded **3be** in 62% yield (118 mg).

Faint white solid; mp 155–157 °C; ^{1}H NMR ($CDCl_3$, 400 MHz): δ_H 8.94 (s, 1H), 8.56 (s, 1H), 8.39 (d, *J* = 7.6 Hz, 1H), 7.80 (d, *J* = 8.0 Hz, 1H), 7.61 (d, *J* = 8.0 Hz, 2H), 7.52 (d, *J* = 8.0 Hz, 2H), 7.41 (m, 1H); ^{13}C

NMR ($CDCl_3$, 101 MHz): δ_C 185.6, 158.1, 137.6, 135.5, 134.5, 134.2, 132.3, 130.2, 130.1, 122.8, 121.5, 118.3; HRMS (ESI-TOF, *m/z*): calcd for $C_{14}H_8Br_2NO_2$ $[M - H]^-$, 379.8927; found, 379.8929.

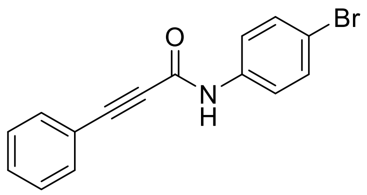

***N*-(4-Bromophenyl)-3-phenylpropiolamide (3bf)**[17]

To a 10 mL Schlenk tube equipped with a magnetic stir bar was added carboxylic acid **2af** (146.0 mg, 1.0 mmol, 2.0 equiv), isocyanide **1a** (90.5 mg, 0.5 mmol, 1.0 equiv), acetone (5.0 mL), H_2O (250 µL), Ag_2CO_3 (27.6 mg, 0.10 mmol, 0.2 equiv). The reaction mixture was stirred under air at 60 °C in an oil bath for about 8 h. The resulting mixture was concentrated and the residue was taken up in ethyl acetate. The organic layer was washed with brine, dried over Na_2SO_4 and concentrated. Purification of the crude product by column chromatography (silica gel; petroleum ether/ethyl acetate = 13:1) afforded **3bf** in 47% yield (70 mg).

Faint white solid; mp 113–115 °C; ^1H NMR ($CDCl_3$, 400 MHz): δ_H 7.65 (s, 1H), 7.57 (d, *J* = 6.8 Hz, 2H), 7.47–7.44 (m, 5H), 7.40–7.37 (m, 2H); ^{13}C NMR ($CDCl_3$, 101 MHz): δ_C 150.9, 136.4, 132.6, 132.1, 130.5, 128.6, 121.4, 119.7, 117.6, 86.2, 83.2; HRMS (ESI-TOF, *m/z*): calcd for $C_{15}H_{11}BrNO$ $[M + H]^+$, 300.0019; found, 300.0015.

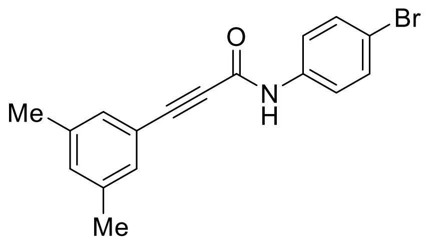

***N*-(4-Bromophenyl)-3-(3,5-dimethylphenyl)propiolamide (3bg)**

To a 10 mL Schlenk tube equipped with a magnetic stir bar was added carboxylic acid **2ag** (174.1 mg, 1.0 mmol, 2.0 equiv), isocyanide **1a** (90.5 mg, 0.5 mmol, 1.0 equiv), acetone (5.0 mL), H_2O (250 µL), Ag_2CO_3 (27.6 mg, 0.10 mmol, 0.2 equiv). The reaction mixture was stirred under air at 60 °C in an oil bath for about 8 h. The resulting mixture was concentrated and the residue was taken up in ethyl acetate. The organic layer was washed with brine, dried over Na_2SO_4 and concentrated. Purification

of the crude product by column chromatography (silica gel; petroleum ether/ethyl acetate = 15:1) afforded **3bg** in 43% yield (70 mg).

Faint white solid; mp 168–170 °C; ^{1}H NMR (DMSO-d_6, 400 MHz): δ_H 10.90 (s, 1H), 7.62 (d, J = 8.4 Hz, 2H), 7.54 (d, J = 8.0 Hz, 2H), 7.48 (d, J = 7.6 Hz, 1H), 7.20 (s, 1H), 7.11 (d, J = 7.6 Hz, 1H), 2.44 (s, 3H), 2.32 (s, 3H); ^{13}C NMR (DMSO-d_6, 101 MHz): δ_C 150.6, 141.0, 140.6, 132.6, 131.7, 130.6, 126.9, 121.6, 116.2, 115.8, 87.2, 84.2, 21.1, 20.1; HRMS (ESI-TOF, m/z): calcd for $C_{17}H_{15}BrNO$ [M + H]$^+$, 328.0332; found, 328.0339.

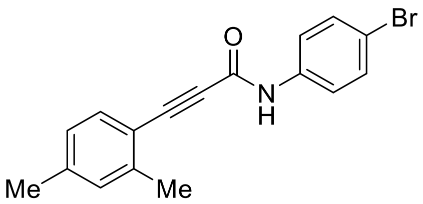

***N*-(4-Bromophenyl)-3-(2,4-dimethylphenyl)propiolamide (3bh)**

To a 10 mL Schlenk tube equipped with a magnetic stir bar was added carboxylic acid **2ah** (174.1 mg, 1.0 mmol, 2.0 equiv), isocyanide **1a** (90.5 mg, 0.5 mmol, 1.0 equiv), acetone (5.0 mL), H_2O (250 µL), Ag_2CO_3 (27.6 mg, 0.10 mmol, 0.2 equiv). The reaction mixture was stirred under air at 60 °C in an oil bath for about 8 h. The resulting mixture was concentrated and the residue was taken up in ethyl acetate. The organic layer was washed with brine, dried over Na_2SO_4 and concentrated. Purification of the crude product by column chromatography (silica gel; petroleum ether/ethyl acetate = 15:1) afforded **3bh** in 46% yield (75 mg).

Faint white solid; mp 165–167 °C; ^{1}H NMR (DMSO-d_6, 400 MHz): δ_H 10.88 (s, 1H), 7.62 (d, J = 8.0 Hz, 2H), 7.53 (d, J = 8.0 Hz, 2H), 7.47 (d, J = 8.0 Hz, 1H), 7.20 (s, 1H), 7.11 (d, J = 8.0 Hz, 1H), 2.44 (s, 3H), 2.32 (s, 3H); ^{13}C NMR (DMSO-d_6, 101 MHz): δ_C 150.6, 141.0, 137.8, 132.6, 131.7, 130.6, 126.9, 121.5, 116.2, 115.7, 87.2, 84.1, 21.1, 20.1; HRMS (ESI-TOF, m/z): calcd for $C_{17}H_{15}BrNO$ [M + H]$^+$, 328.0332; found, 328.0341.

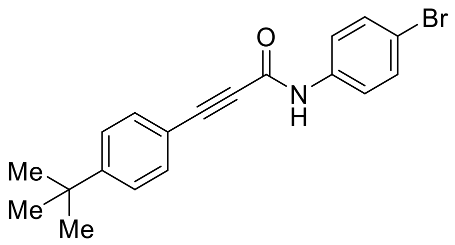

***N*-(4-Bromophenyl)-3-(4-(*tert*-butyl)phenyl)propiolamide (3bi)**

To a 10 mL Schlenk tube equipped with a magnetic stir bar was added carboxylic acid **2ai** (202.1 mg, 1.0 mmol, 2.0 equiv), isocyanide **1a** (90.5 mg, 0.5 mmol, 1.0 equiv), acetone (5.0 mL), H_2O (250 µL), Ag_2CO_3 (27.6 mg, 0.10 mmol, 0.2 equiv). The reaction mixture was stirred under air at 60 °C in an oil bath for about 8 h. The resulting mixture was concentrated and the residue was taken up in ethyl acetate. The organic layer was washed with brine, dried over Na_2SO_4 and concentrated. Purification of the crude product by column chromatography (silica gel; petroleum ether/ethyl acetate = 12:1) afforded **3bi** in 38% yield (68 mg).

Faint white solid; mp 171–172 °C; ^{1}H NMR (DMSO-d_6, 400 MHz): δ_H 10.97 (s, 1H), 7.63–7.58 (m, 4H), 7.55–7.50 (m, 4H), 1.30 (s, 9H); ^{13}C NMR (DMSO-d_6, 101 MHz): δ_C 153.6, 150.4, 137.8, 132.2, 131.7, 125.9, 121.5, 116.4, 115.8, 85.1, 83.6, 34.8, 30.8; HRMS (ESI-TOF, *m/z*): calcd for $C_{19}H_{19}BrNO$ $[M + H]^+$, 356.0645; found, 356.0651.

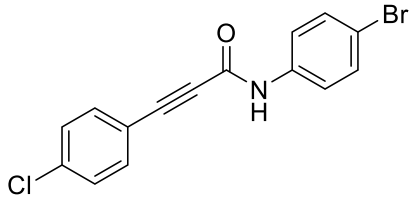

***N*-(4-Bromophenyl)-3-(4-chlorophenyl)propiolamide (3bj)**

To a 10 mL Schlenk tube equipped with a magnetic stir bar was added carboxylic acid **2aj** (180.0 mg, 1.0 mmol, 2.0 equiv), isocyanide **1a** (90.5 mg, 0.5 mmol, 1.0 equiv), acetone (5.0 mL), H_2O (250 µL), Ag_2CO_3 (27.6 mg, 0.10 mmol, 0.2 equiv). The reaction mixture was stirred under air at 60 °C in an oil bath for about 8 h. The resulting mixture was concentrated and the residue was taken up in ethyl acetate. The organic layer was washed with brine, dried over Na_2SO_4 and concentrated. Purification of the crude product by column chromatography (silica gel; petroleum ether/ethyl acetate = 12:1) afforded **3bj** in 41% yield (68 mg).

Faint white solid; mp 183–185 °C; ^{1}H NMR (DMSO-d_6, 400 MHz): δ_H 11.04 (s, 1H), 7.68 (d, J = 8.0 Hz, 2H), 7.62–7.53 (m, 6H); ^{13}C NMR (DMSO-d_6, 101 MHz): δ_C 150.1, 137.7, 135.5, 134.0, 131.7, 129.3, 121.5, 118.3, 115.9, 84.8, 83.5; HRMS (ESI-TOF, *m/z*): calcd for $C_{15}H_{10}BrClNO$ [M + H]$^+$, 333.9629; found, 333.9638.

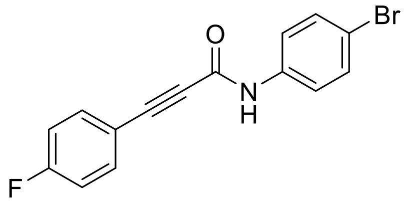

N-(4-Bromophenyl)-3-(4-fluorophenyl)propiolamide (3bk)

To a 10 mL Schlenk tube equipped with a magnetic stir bar was added carboxylic acid **2ak** (164.0 mg, 1.0 mmol, 2.0 equiv), isocyanide **1a** (90.5 mg, 0.5 mmol, 1.0 equiv), acetone (5.0 mL), H_2O (250 µL), Ag_2CO_3 (27.6 mg, 0.10 mmol, 0.2 equiv). The reaction mixture was stirred under air at 60 °C in an oil bath for about 8 h. The resulting mixture was concentrated and the residue was taken up in ethyl acetate. The organic layer was washed with brine, dried over Na_2SO_4 and concentrated. Purification of the crude product by column chromatography (silica gel; petroleum ether/ethyl acetate = 12:1) afforded **3bk** in 40% yield (63 mg).

Faint white solid; mp 169–170 °C; ^{1}H NMR (DMSO-d_6, 400 MHz): δ_H 11.00 (s, 1H), 7.75–7.71 (m, 2H), 7.61 (d, J = 8.4 Hz, 2H), 7.61 (d, J = 8.4 Hz, 2H), 7.38–7.33 (m, 2H); ^{13}C NMR (DMSO-d_6, 101 MHz): δ_C 150.3, 137.7, 135.0 (d, J_{C-F} = 8.9 Hz), 131.7, 121.5, 116.5 (d, J_{C-F} = 23.5 Hz), 115.9, 83.8; HRMS (ESI-TOF, *m/z*): calcd for $C_{15}H_{10}BrFNO$ [M + H]$^+$, 317.9924; found, 317.9929.

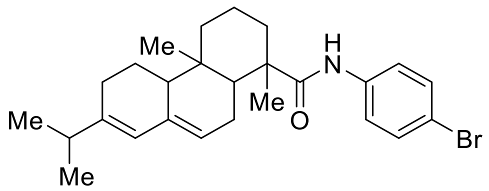

***N*-(4-Bromophenyl)-7-isopropyl-1,4a-dimethyl-1,2,3,4,4a,4b,5,6,10,10a-decahydrophenanthrene-1-carboxamide (3bl)**

To a 10 mL Schlenk tube equipped with a magnetic stir bar was added carboxylic acid **2al** (302.2 mg, 1.0 mmol, 2.0 equiv), isocyanide **1a** (90.5 mg, 0.5 mmol, 1.0 equiv), acetone (5.0 mL), H_2O (250 µL), Ag_2CO_3 (27.6 mg, 0.10 mmol, 0.2 equiv). The reaction mixture was stirred under air at 60 °C in an oil bath for about 8 h. The resulting mixture was concentrated and the residue was taken up in ethyl acetate. The organic layer was washed with brine, dried over Na_2SO_4 and concentrated. Purification of the crude product by column chromatography (silica gel; petroleum ether/ethyl acetate = 13:1) afforded **3bl** in 72% yield (164 mg).

Faint white solid; mp 96–98 °C; ^{1}H NMR ($CDCl_3$, 400 MHz): δ_H 7.42 (s, 4H), 7.40 (s, 1H), 5.75 (s, 1H), 5.33 (s, 1H), 2.24–2.20 (m, 1H), 2.08–1.80 (m, 6H), 1.39 (s, 3H), 1.32–1.10 (m, 5H), 1.02–1.00 (m, 6H), 0.87–0.83 (m, 6H); ^{13}C NMR ($CDCl_3$, 101 MHz): δ_C 176.6, 145.5, 137.0, 135.6, 131.9, 122.3, 121.7, 120.1, 116.7, 51.0, 47.4, 46.1, 38.2, 37.5, 34.9, 34.7, 27.4, 25.4, 22.5, 21.4, 20.8, 18.3, 17.1, 14.1; HRMS (ESI-TOF, *m/z*): calcd for $C_{26}H_{35}BrNO$ $[M + H]^+$, 456.1897; found, 456.1894.

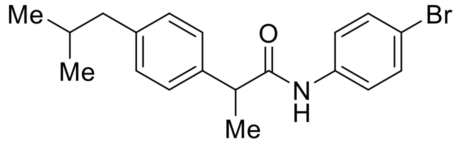

***N*-(4-Bromophenyl)-2-(4-isobutylphenyl)propanamide (3bm)**

To a 10 mL Schlenk tube equipped with a magnetic stir bar was added carboxylic acid **2am** (206.1 mg, 1.0 mmol, 2.0 equiv), isocyanide **1a** (90.5 mg, 0.5 mmol, 1.0 equiv), acetone (5.0 mL), H_2O (250 µL), Ag_2CO_3 (27.6 mg, 0.10 mmol, 0.2 equiv). The reaction mixture was stirred under air at 60 °C in an oil bath for about 8 h. The resulting mixture was concentrated and the residue was taken up in ethyl acetate. The organic layer was washed with brine, dried over Na_2SO_4 and concentrated. Purification of the crude product by column chromatography (silica gel; petroleum ether/ethyl acetate = 10:1) afforded **3bm** in 83% yield (149 mg).

Faint white solid; mp 110–112 °C; ^{1}H NMR (DMSO-d_6, 400 MHz): δ_H 10.16 (s, 1H), 7.57 (d, *J* = 8.8 Hz, 2H), 7.45 (d, *J* = 8.4 Hz, 2H), 7.27 (d, *J* = 7.6 Hz, 2H), 7.10 (d, *J* = 8.0 Hz, 2H), 3.80–3.75 (m, 1H), 2.40 (d, *J* = 7.2 Hz, 2H), 1.82–1.76 (m, 1H), 1.39 (d, *J* = 7.2 Hz, 3H), 0.84 (d, *J* = 6.4 Hz, 6H); ^{13}C NMR (DMSO-d_6, 101 MHz): δ_C 172.5, 139.5, 138.9, 138.6, 131.4, 128.9, 126.9, 121.0, 114.6, 45.6, 44.2, 29.6, 22.1, 18.6; HRMS (ESI-TOF, *m/z*): calcd for $C_{19}H_{23}BrNO$ [M + H]$^+$, 360.0958; found, 360.0979.

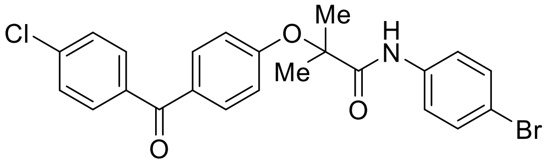

***N*-(4-Bromophenyl)-2-(4-(4-chlorobenzoyl)phenoxy)-2-methylpropanamide (3bn)**

To a 10 mL Schlenk tube equipped with a magnetic stir bar was added carboxylic acid **2an** (318.1 mg, 1.0 mmol, 2.0 equiv), isocyanide **1a** (90.5 mg, 0.5 mmol, 1.0 equiv), acetone (5.0 mL), H_2O (250 µL), Ag_2CO_3 (27.6 mg, 0.10 mmol, 0.2 equiv). The reaction mixture was stirred under air at 60 °C in an oil bath for about 8 h. The resulting mixture was concentrated and the residue was taken up in ethyl acetate. The organic layer was washed with brine, dried over Na_2SO_4 and concentrated. Purification of the crude product by column chromatography (silica gel; petroleum ether/ethyl acetate = 9:1) afforded **3bn** in 59% yield (139 mg).

Faint white solid; mp 112–113 °C; ^{1}H NMR (DMSO-d_6, 400 MHz): δ_H 10.18 (s, 1H), 7.74–7.68 (m, 4H), 7.65–7.58 (m, 4H), 7.48 (d, *J* = 8.4 Hz, 2H), 7.02 (d, *J* = 8.4 Hz, 2H), 1.63 (s, 6H); ^{13}C NMR (DMSO-d_6, 101 MHz): δ_C 193.2, 172.0, 159.0, 137.8, 137.1, 136.1, 131.8, 131.3, 131.2, 130.0, 128.6, 122.4, 118.2, 115.6, 81.0, 24.8; HRMS (ESI-TOF, *m/z*): calcd for $C_{23}H_{20}BrClNO_3$ [M + H]$^+$, 472.0310; found, 472.0317.

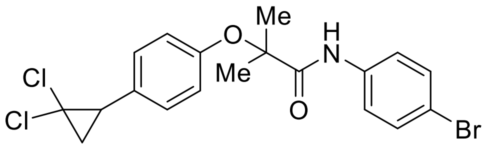

***N*-(4-Bromophenyl)-2-(4-(2,2-dichlorocyclopropyl)phenoxy)-2-methylpropanamide (3bo)**

To a 10 mL Schlenk tube equipped with a magnetic stir bar was added carboxylic acid **2ao** (288.0 mg, 1.0 mmol, 2.0 equiv), isocyanide **1a** (90.5 mg, 0.5 mmol, 1.0 equiv), acetone (5.0 mL), H_2O (250 µL), Ag_2CO_3 (27.6 mg, 0.10 mmol, 0.2 equiv). The reaction mixture was stirred under air at 60 °C in an oil bath for about 8 h. The resulting mixture was concentrated and the residue was taken up in ethyl

acetate. The organic layer was washed with brine, dried over Na_2SO_4 and concentrated. Purification of the crude product by column chromatography (silica gel; petroleum ether/ethyl acetate = 9:1) afforded **3bo** in 64% yield (141 mg).

Faint white solid; mp 108–110 °C; ^1H NMR (DMSO-d_6, 400 MHz): δ_H 10.15 (s, 1H), 7.67 (d, J = 8.0 Hz, 2H), 7.48 (d, J = 8.0 Hz, 2H), 7.22 (d, J = 8.0 Hz, 2H), 6.89 (d, J = 7.6 Hz, 2H), 3.03–2.98 (m, 1H), 2.08–1.99 (m, 2H), 1.53 (s, 6H); ^{13}C NMR (DMSO-d_6, 101 MHz): δ_C 172.7, 154.0, 137.9, 131.3, 129.8, 128.5, 122.3, 119.4, 115.4, 80.6, 61.9, 33.9, 24.7; HRMS (ESI-TOF, m/z): calcd for $C_{19}H_{19}BrCl_2NO_2$ [M + H]$^+$, 441.9971; found, 441.9986.

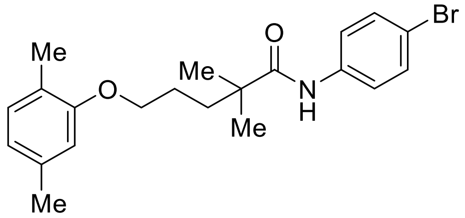

***N*-(4-Bromophenyl)-5-(2,5-dimethylphenoxy)-2,2-dimethylpentanamide (3bp)**

To a 10 mL Schlenk tube equipped with a magnetic stir bar was added carboxylic acid **2ap** (250.2 mg, 1.0 mmol, 2.0 equiv), isocyanide **1a** (90.5 mg, 0.5 mmol, 1.0 equiv), acetone (5.0 mL), H_2O (250 µL), Ag_2CO_3 (27.6 mg, 0.10 mmol, 0.2 equiv). The reaction mixture was stirred under air at 60 °C in an oil bath for about 8 h. The resulting mixture was concentrated and the residue was taken up in ethyl acetate. The organic layer was washed with brine, dried over Na_2SO_4 and concentrated. Purification of the crude product by column chromatography (silica gel; petroleum ether/ethyl acetate = 10:1) afforded **3bp** in 77% yield (155 mg).

Faint white solid; mp 107–108 °C; ^1H NMR (DMSO-d_6, 400 MHz): δ_H 9.32 (s, 1H), 7.62 (d, J = 8.4 Hz, 2H), 7.46 (d, J = 8.4 Hz, 2H), 6.96 (d, J = 7.2 Hz, 1H), 6.65 (s, 1H), 6.60 (d, J = 7.2 Hz, 1H), 3.91–3.88 (m, 2H), 2.21 (s, 3H), 2.06 (s, 3H), 1.76–1.64 (m, 4H), 1.23 (s, 6H); ^{13}C NMR (DMSO-d_6, 101 MHz): δ_C 175.8, 156.4, 138.6, 136.0, 131.1, 130.0, 122.4, 122.2, 120.5, 114.8, 112.0, 42.4, 36.6, 25.0, 24.6, 21.0, 15.5; HRMS (ESI-TOF, m/z): calcd for $C_{21}H_{27}BrNO_2$ [M + H]$^+$, 404.1220; found, 404.1231.

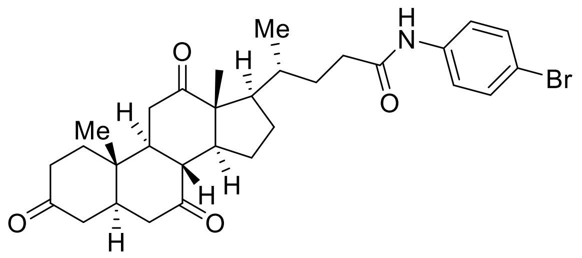

(*R*)-*N*-(4-Bromophenyl)-4-((5*R*,8*R*,9*S*,10*S*,13*R*,14*S*,17*R*)-10,13-dimethyl-3,7,12-trioxohexadecahydro-1*H*-cyclopenta[*a*]phenanthren-17-yl)pentanamide (3bq)

To a 10 mL Schlenk tube equipped with a magnetic stir bar was added carboxylic acid **2aq** (402.2 mg, 1.0 mmol, 2.0 equiv), isocyanide **1a** (90.5 mg, 0.5 mmol, 1.0 equiv), acetone (5.0 mL), H_2O (250 µL), Ag_2CO_3 (27.6 mg, 0.10 mmol, 0.2 equiv). The reaction mixture was stirred under air at 60 °C in an oil bath for about 8 h. The resulting mixture was concentrated and the residue was taken up in ethyl acetate. The organic layer was washed with brine, dried over Na_2SO_4 and concentrated. Purification of the crude product by column chromatography (silica gel; petroleum ether/ethyl acetate = 8:1) afforded **3bq** in 56% yield (156 mg).

Faint white solid; mp 227–229 °C; ^{1}H NMR (DMSO-d_6, 400 MHz): δ_H 10.00 (s, 1H), 7.56 (d, *J* = 8.4 Hz, 2H), 7.46 (d, *J* = 8.4 Hz, 2H), 3.08–2.97 (m, 2H), 2.88–2.81 (m, 3H), 2.37–2.13 (m, 6H), 1.99–1.78 (m, 7H), 1.33 (s, 6H), 1.11–1.08 (m, 3H), 1.01 (s, 3H), 0.81 (d, *J* = 4.8 Hz, 3H); ^{13}C NMR (DMSO-d_6, 101 MHz): δ_C 212.0, 209.6, 174.8, 171.7, 138.7, 131.4, 120.9, 114.4, 56.2, 51.2, 47.9, 46.0, 45.3, 44.5, 44.0, 42.5, 38.3, 36.1, 35.6, 35.1, 34.5, 33.6, 30.8, 27.2, 24.6, 21.1, 18.8, 11.4; HRMS (ESI-TOF, *m/z*): calcd for $C_{30}H_{39}BrNO_4$ $[M + H]^+$, 556.2057; found, 556.2059.

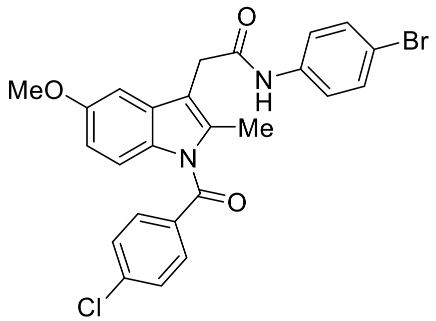

***N*-(4-Bromophenyl)-2-(1-(4-chlorobenzoyl)-5-methoxy-2-methyl-1*H*-indol-3-yl)acetamide (3br)**

To a 10 mL Schlenk tube equipped with a magnetic stir bar was added carboxylic acid **2ar** (357.1 mg, 1.0 mmol, 2.0 equiv), isocyanide **1a** (90.5 mg, 0.5 mmol, 1.0 equiv), acetone (5.0 mL), H_2O (250 µL), Ag_2CO_3 (27.6 mg, 0.10 mmol, 0.2 equiv). The reaction mixture was stirred under air at 60 °C in an oil

bath for about 8 h. The resulting mixture was concentrated and the residue was taken up in ethyl acetate. The organic layer was washed with brine, dried over Na_2SO_4 and concentrated. Purification of the crude product by column chromatography (silica gel; petroleum ether/ethyl acetate = 8:1) afforded **3br** in 73% yield (186 mg).

Faint white solid; mp 175–177 °C; ^{1}H NMR (DMSO-*d*$_6$, 400 MHz): δ_H 10.38 (s, 1H), 7.70–7.65 (m, 4H), 7.59 (d, *J* = 8.4 Hz, 2H), 7.48 (d, *J* = 8.0 Hz, 2H), 7.18 (s, 1H), 6.93 (d, *J* = 8.8 Hz, 1H), 6.71 (d, *J* = 9.2 Hz, 1H), 3.75 (d, *J* = 4.8 Hz, 3H), 2.67–2.64 (m, 1H), 2.28 (s, 3H), 2.21–2.20 (m, 1H); ^{13}C NMR (DMSO-*d*$_6$, 101 MHz): δ_C 168.6, 167.8, 155.6, 138.5, 137.6, 135.4, 134.2, 131.5, 131.1, 130.8, 130.2, 129.0, 121.1, 114.8, 114.6, 113.9, 111.2, 101.9, 55.4, 45.5, 32.0, 13.4; HRMS (ESI-TOF, *m/z*): calcd for $C_{25}H_{21}BrClN_2O_3$ $[M + H]^+$, 511.0419; found, 511.0427.

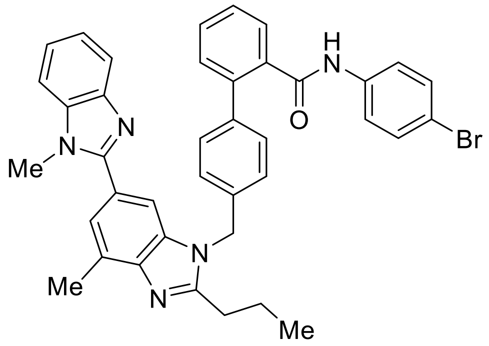

N-(4-Bromophenyl)-4'-((1,7'-dimethyl-2'-propyl-1*H*,3'*H*-[2,5'-bibenzo[*d*]imidazol]-3'-yl)methyl)-[1,1'-biphenyl]-2-carboxamide (3bs)

To a 10 mL Schlenk tube equipped with a magnetic stir bar was added carboxylic acid **2as** (514.2 mg, 1.0 mmol, 2.0 equiv), isocyanide **1a** (90.5 mg, 0.5 mmol, 1.0 equiv), acetone (5.0 mL), H_2O (250 µL), Ag_2CO_3 (27.6 mg, 0.10 mmol, 0.2 equiv). The reaction mixture was stirred under air at 60 °C in an oil bath for about 8 h. The resulting mixture was concentrated and the residue was taken up in ethyl acetate. The organic layer was washed with brine, dried over Na_2SO_4 and concentrated. Purification of the crude product by column chromatography (silica gel; petroleum ether/ethyl acetate = 8:1) afforded **3bs** in 71% yield (237 mg).

Faint white solid; mp 234–236 °C; ^{1}H NMR (DMSO-*d*$_6$, 400 MHz): δ_H 10.19 (s, 1H), 7.65 (s, 1H), 7.52 (d, *J* = 7.6 Hz, 1H), 7.44–7.40 (m, 3H), 7.36–7.25 (m, 9H), 7.17–7.08 (m, 2H), 7.02 (d, *J* = 7.6 Hz, 2H), 5.45 (s, 2H), 3.67 (s, 3H), 2.71–2.67 (m, 2H), 2.38 (s, 3H), 1.64–1.58 (m, 2H), 0.82–0.78 (m, 3H); ^{13}C NMR (DMSO-*d*$_6$, 101 MHz): δ_C 167.8, 156.1, 154.0, 142.6, 142.5, 139.1, 138.7, 138.3, 136.7, 136.6, 136.2,

134.8, 131.3, 129.94, 129.90, 128.7, 128.2, 127.8, 127.3, 126.5, 123.3, 123.2, 122.0, 121.7, 121.4, 118.7, 115.2, 110.3, 109.1, 45.9, 31.7, 28.7, 20.6, 16.5, 13.7; HRMS (ESI-TOF, *m/z*): calcd for $C_{39}H_{35}BrN_5O$ $[M + H]^+$, 668.2019; found, 668.2037.

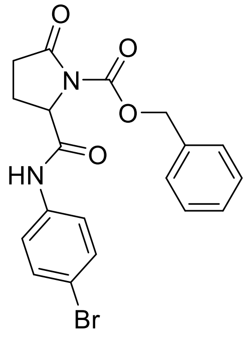

Benzyl 2-((4-bromophenyl)carbamoyl)-5-oxopyrrolidine-1-carboxylate (3bt)

To a 10 mL Schlenk tube equipped with a magnetic stir bar was added carboxylic acid **2at** (263.1 mg, 1.0 mmol, 2.0 equiv), isocyanide **1a** (90.5 mg, 0.5 mmol, 1.0 equiv), acetone (5.0 mL), H_2O (250 µL), Ag_2CO_3 (27.6 mg, 0.10 mmol, 0.2 equiv). The reaction mixture was stirred under air at 60 °C in an oil bath for about 8 h. The resulting mixture was concentrated and the residue was taken up in ethyl acetate. The organic layer was washed with brine, dried over Na_2SO_4 and concentrated. Purification of the crude product by column chromatography (silica gel; petroleum ether/ethyl acetate = 8:1) afforded **3bt** in 80% yield (166 mg).

Faint white solid; mp 179–181 °C; ^{1}H NMR (DMSO-d_6, 400 MHz): δ_H 10.46 (s, 1H), 7.56–7.50 (m, 4H), 7.33–7.26 (m, 5H), 5.22 (d, *J* = 12.8 Hz, 1H), 5.15 (d, *J* = 12.8 Hz, 1H), 4.75 (d, *J* = 8.0 Hz, 1H), 2.56–2.52 (m, 1H), 2.48–2.47 (m, 1H), 2.38–2.33 (m, 1H), 2.00–1.94 (m, 1H); ^{13}C NMR (DMSO-d_6, 101 MHz): δ_C 173.5, 169.7, 150.5, 138.0, 135.4, 131.6, 128.3, 128.0, 127.4, 121.2, 115.2, 67.1, 59.7, 30.9, 21.9; HRMS (ESI-TOF, *m/z*): calcd for $C_{19}H_{18}BrN_2O_4$ $[M + H]^+$, 417.0444; found, 417.0459.

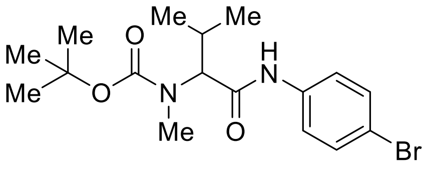

tert-Butyl (1-((4-bromophenyl)amino)-3-methyl-1-oxobutan-2-yl)(methyl)carbamate (3bu)

To a 10 mL Schlenk tube equipped with a magnetic stir bar was added carboxylic acid **2au** (231.1 mg, 1.0 mmol, 2.0 equiv), isocyanide **1a** (90.5 mg, 0.5 mmol, 1.0 equiv), acetone (5.0 mL), H_2O (250 µL), Ag_2CO_3 (27.6 mg, 0.10 mmol, 0.2 equiv). The reaction mixture was stirred under air at 60 °C in an oil bath for about 8 h. The resulting mixture was concentrated and the residue was taken up in ethyl acetate. The organic layer was washed with brine, dried over Na_2SO_4 and concentrated. Purification of the crude product by column chromatography (silica gel; petroleum ether/ethyl acetate = 10:1) afforded **3bu** in 85% yield (163 mg).

Faint white solid; mp 128–130 °C; ^{1}H NMR (DMSO-d_6, 400 MHz): δ_H 10.30 (s, 1H), 7.60 (d, J = 8.0 Hz, 2H), 7.48 (d, J = 8.0 Hz, 2H), 4.35 (d, J = 10.8 Hz, 2H), 2.86 (s, 3H), 1.41 (s, 9H), 0.92–0.82 (m, 7H); ^{13}C NMR (DMSO-d_6, 101 MHz): δ_C 169.5, 138.0, 131.5, 121.5, 115.2, 79.1, 63.4, 29.7, 28.0, 26.9, 19.1, 18.7; HRMS (ESI-TOF, m/z): calcd for $C_{17}H_{26}BrN_2O_3$ $[M + H]^+$, 385.1121; found, 385.1143.

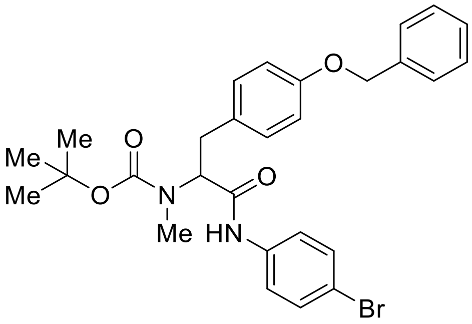

tert-Butyl (3-(4-(benzyloxy)phenyl)-1-((4-bromophenyl)amino)-1-oxopropan-2-yl)(methyl)carbamate (3bv)

To a 10 mL Schlenk tube equipped with a magnetic stir bar was added carboxylic acid **2av** (385.2 mg, 1.0 mmol, 2.0 equiv), isocyanide **1a** (90.5 mg, 0.5 mmol, 1.0 equiv), acetone (5.0 mL), H_2O (250 µL), Ag_2CO_3 (27.6 mg, 0.10 mmol, 0.2 equiv). The reaction mixture was stirred under air at 60 °C in an oil bath for about 8 h. The resulting mixture was concentrated and the residue was taken up in ethyl acetate. The organic layer was washed with brine, dried over Na_2SO_4 and concentrated. Purification

of the crude product by column chromatography (silica gel; petroleum ether/ethyl acetate = 10:1) afforded **3bv** in 71% yield (191 mg).

Faint white solid; mp 135–136 °C; ^{1}H NMR (DMSO-d_6, 400 MHz): δ_H 10.05 (s, 1H), 7.62–7.58 (m, 2H), 7.51–7.48 (m, 2H), 7.43 (d, *J* = 7.6 Hz, 2H), 7.38 (t, *J* = 7.2 Hz, 2H), 7.33 (d, *J* = 6.8 Hz, 1H), 7.18 (d, *J* = 8.0 Hz, 2H), 6.96–6.92 (m, 2H), 5.06 (s, 2H), 3.18–3.14 (m, 1H), 2.95–2.89 (m, 1H), 2.77–2.69 (m, 3H), 1.28 (s, 9H); ^{13}C NMR (DMSO-d_6, 101 MHz): δ_C 169.7, 156.9, 138.2, 137.2, 131.4, 129.9, 129.8, 128.4, 127.7, 127.6, 121.8, 121.6, 115.1, 114.5, 79.0, 69.1, 61.5, 59.2, 45.6, 33.6, 31.9, 30.8, 27.9; HRMS (ESI-TOF, *m/z*): calcd for $C_{28}H_{32}BrN_2O_4$ [M + H]$^+$, 539.1540; found, 539.1552.

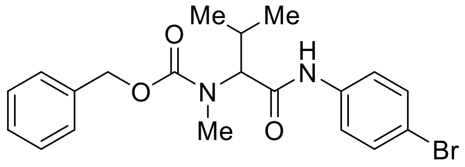

Benzyl (1-((4-bromophenyl)amino)-3-methyl-1-oxobutan-2-yl)(methyl)carbamate (3bw)

To a 10 mL Schlenk tube equipped with a magnetic stir bar was added carboxylic acid **2aw** (265.3 mg, 1.0 mmol, 2.0 equiv), isocyanide **1a** (90.5 mg, 0.5 mmol, 1.0 equiv), acetone (5.0 mL), H_2O (250 µL), Ag_2CO_3 (27.6 mg, 0.10 mmol, 0.2 equiv). The reaction mixture was stirred under air at 60 °C in an oil bath for about 8 h. The resulting mixture was concentrated and the residue was taken up in ethyl acetate. The organic layer was washed with brine, dried over Na_2SO_4 and concentrated. Purification of the crude product by column chromatography (silica gel; petroleum ether/ethyl acetate = 10:1) afforded **3bw** in 58% yield (121 mg).

Colorless oil; ^{1}H NMR (DMSO-d_6, 400 MHz): δ_H 10.35 (s, 1H), 7.61–7.56 (m, 2H), 7.51–7.47 (m, 2H), 7.37–7.28 (m, 5H), 5.16–5.08 (m, 2H), 4.42–4.28 (m, 1H), 2.95 (s, 3H), 2.21–2.15 (m, 1H), 0.85 (dd, *J* = 24.4, 6.4 Hz, 6H); ^{13}C NMR (DMSO-d_6, 101 MHz): δ_C 169.3, 156.2, 137.9, 136.8, 131.5, 128.4, 127.8, 127.4, 121.5, 115.2, 66.5, 64.3, 29.6, 26.9, 19.0; HRMS (ESI-TOF, *m/z*): calcd for $C_{20}H_{24}BrN_2O_3$ [M + H]$^+$, 419.0965; found, 419.0988.

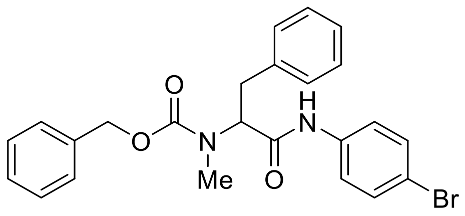

Benzyl (1-((4-bromophenyl)amino)-1-oxo-3-phenylpropan-2-yl)(methyl)carbamate (3bx)

To a 10 mL Schlenk tube equipped with a magnetic stir bar was added carboxylic acid **2ax** (313.1 mg, 1.0 mmol, 2.0 equiv), isocyanide **1a** (90.5 mg, 0.5 mmol, 1.0 equiv), acetone (5.0 mL), H_2O (250 μL), Ag_2CO_3 (27.6 mg, 0.10 mmol, 0.2 equiv). The reaction mixture was stirred under air at 60 °C in an oil bath for about 8 h. The resulting mixture was concentrated and the residue was taken up in ethyl acetate. The organic layer was washed with brine, dried over Na_2SO_4 and concentrated. Purification of the crude product by column chromatography (silica gel; petroleum ether/ethyl acetate = 10:1) afforded **3bx** in 68% yield (152 mg).

Faint white solid; mp 123–125 °C; ^{1}H NMR (DMSO-d_6, 400 MHz): δ_H 10.18 (s, 1H), 7.60–6.57 (m, 2H), 7.50 (d, J = 8.8 Hz, 2H), 7.35–7.19 (m, 10H), 5.10–4.98 (m, 3H), 3.28–3.23 (m, 1H), 3.07–3.01 (m, 1H), 2.86 (s, 3H); ^{13}C NMR (DMSO-d_6, 101 MHz): δ_C 169.4, 156.0, 155.4, 138.0, 137.5, 136.7, 131.5, 128.8, 128.3, 127.6, 127.1, 126.4, 121.7, 115.2, 66.2, 60.3, 34.4, 31.3, 30.8; HRMS (ESI-TOF, m/z): calcd for $C_{24}H_{24}BrN_2O_3$ $[M + H]^+$, 467.0965; found, 467.0974.

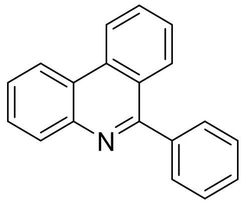

6-Phenylphenanthridine (4a)[18]

To a 10 mL Schlenk tube equipped with a magnetic stir bar was added carboxylic acid **2n** (122.0 mg, 1.0 mmol, 2.0 equiv), isocyanide **1ab** (89.5 mg, 0.5 mmol, 1.0 equiv), acetone (5.0 mL), H_2O (250 μL), Ag_2CO_3 (27.6 mg, 0.10 mmol, 0.2 equiv). The reaction mixture was stirred under air at 60 °C in an oil bath for about 8 h. The resulting mixture was concentrated and the residue was taken up in ethyl acetate. The organic layer was washed with brine, dried over Na_2SO_4 and concentrated. Purification of the crude product by column chromatography (silica gel; petroleum ether/ethyl acetate = 40:1) afforded **4a** in 49% yield (63 mg).

Faint yellow oil; ^{1}H NMR ($CDCl_3$, 400 MHz): δ_H 8.66–8.61 (m, 2H), 8.55 (d, J = 8.0 Hz, 1H), 8.23 (d, J = 8.0 Hz, 1H), 7.88 (t, J = 7.2 Hz, 1H), 7.79–7.74 (m, 4H), 7.68 (t, J = 7.6 Hz, 1H), 7.45–7.43 (m, 3H); ^{13}C NMR ($CDCl_3$, 101 MHz): δ_C 144.9, 144.1, 132.5, 132.3, 131.1, 130.1, 129.4, 129.0, 128.5, 128.1, 127.7, 127.6, 126.7, 123.8, 122.0, 93.7, 87.1; HRMS (ESI-TOF, *m/z*): calcd for $C_{19}H_{14}N$ [M + H]$^+$, 256.1121; found, 256.1128.

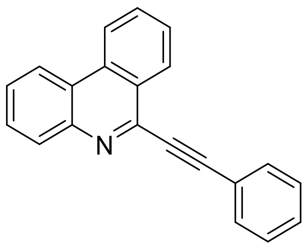

6-(Phenylethynyl)phenanthridine (4b)[19]

To a 10 mL Schlenk tube equipped with a magnetic stir bar was added carboxylic acid **2af** (146.0 mg, 1.0 mmol, 2.0 equiv), isocyanide **1ab** (89.5 mg, 0.5 mmol, 1.0 equiv), acetone (5.0 mL), H_2O (250 µL), Ag_2CO_3 (27.6 mg, 0.10 mmol, 0.2 equiv). The reaction mixture was stirred under air at 60 °C in an oil bath for about 8 h. The resulting mixture was concentrated and the residue was taken up in ethyl acetate. The organic layer was washed with brine, dried over Na_2SO_4 and concentrated. Purification of the crude product by column chromatography (silica gel; petroleum ether/ethyl acetate = 40:1) afforded **4b** in 24% yield (33 mg).

Faint white solid; mp 107–108 °C; ^{1}H NMR ($CDCl_3$, 400 MHz): δ_H 8.71 (d, J = 8.4 Hz, 1H), 8.62 (d, J = 8.4 Hz, 1H), 8.26 (d, J = 8.4 Hz, 1H), 8.11 (d, J = 8.4 Hz, 1H), 7.86 (t, J = 7.2 Hz, 1H), 7.78–7.67 (m, 4H), 7.63–7.53 (m, 4H); ^{13}C NMR ($CDCl_3$, 101 MHz): δ_C 161.2, 143.7, 139.7, 137.0, 136.2, 133.4, 130.5, 130.3, 129.7, 128.9, 128.8, 128.7, 128.4, 127.1, 126.9, 125.2, 123.7, 122.2, 121.9; HRMS (ESI-TOF, *m/z*): calcd for $C_{21}H_{14}N$ [M + H]$^+$, 280.1121; found, 280.1133.

IV. NMR spectra

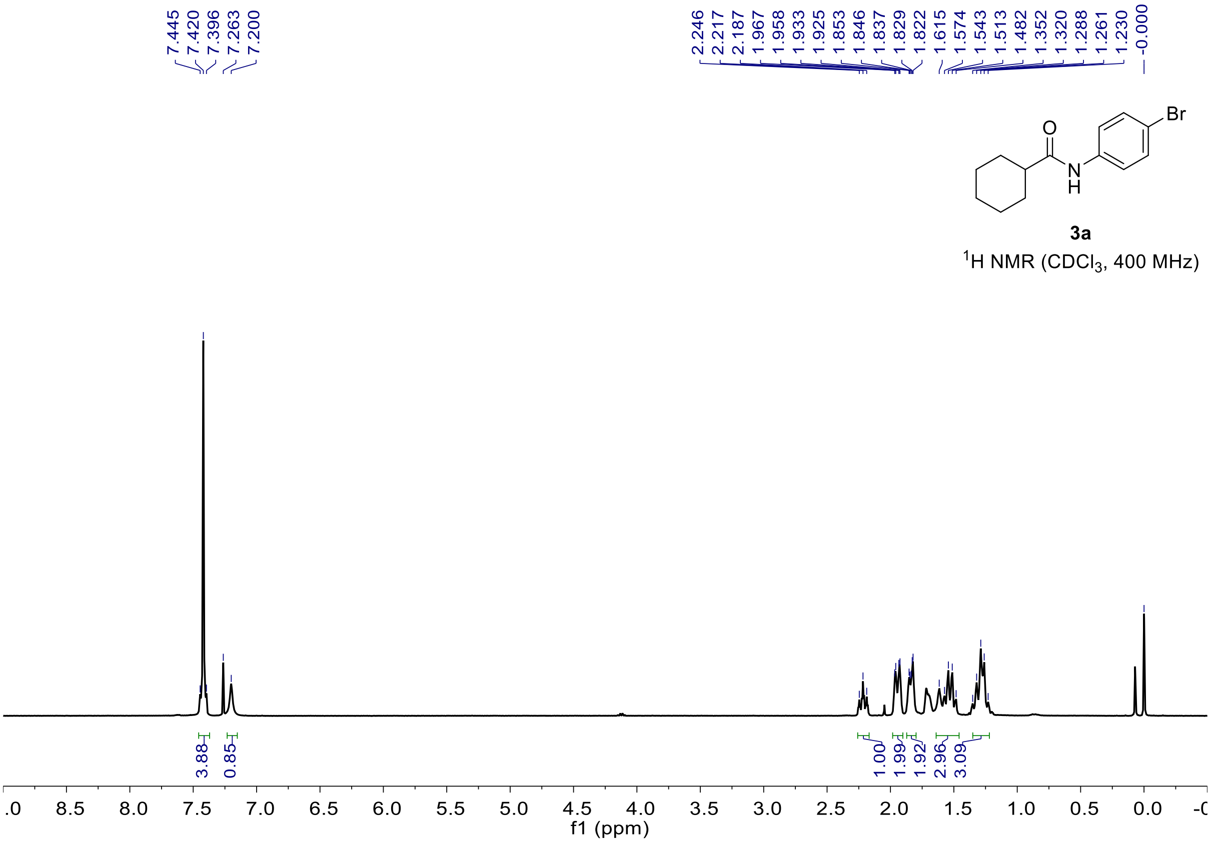

3a

^{1}H NMR ($CDCl_3$, 400 MHz)

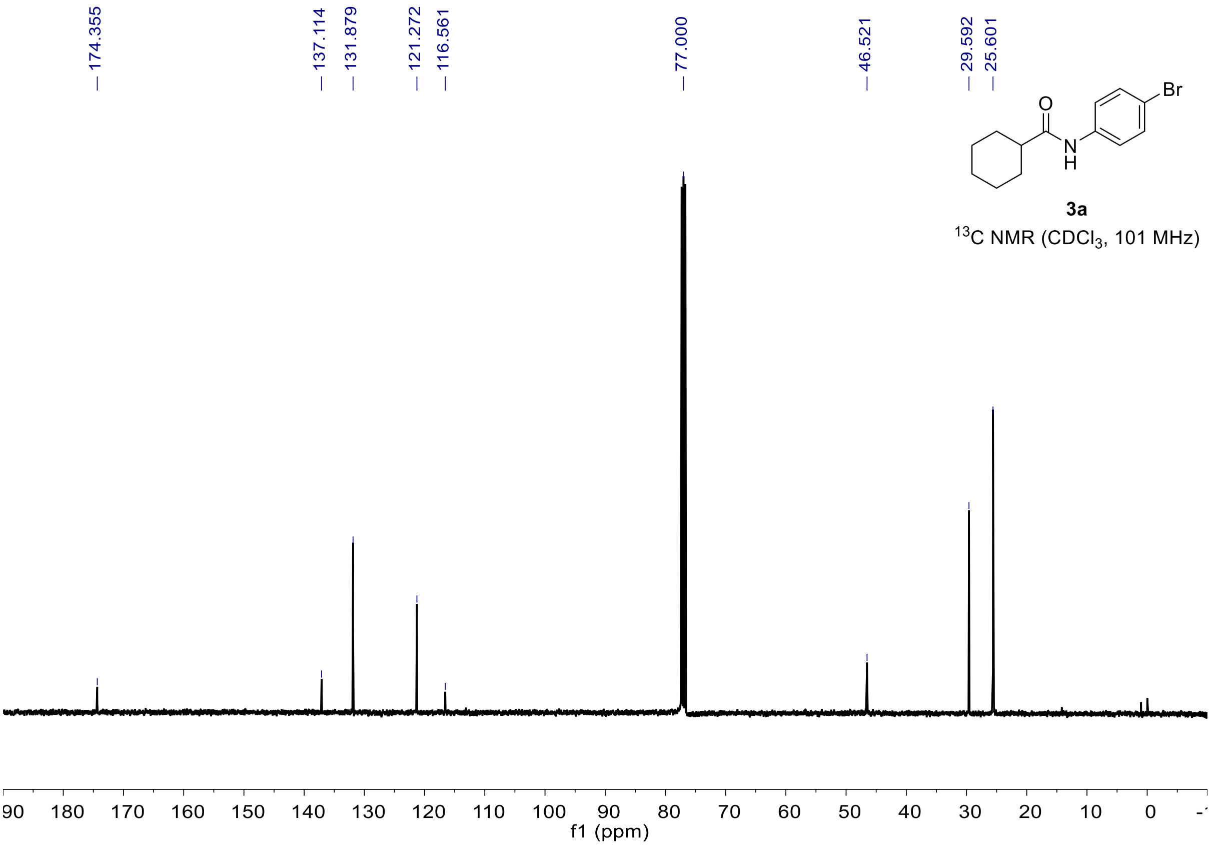

3a

^{13}C NMR ($CDCl_3$, 101 MHz)

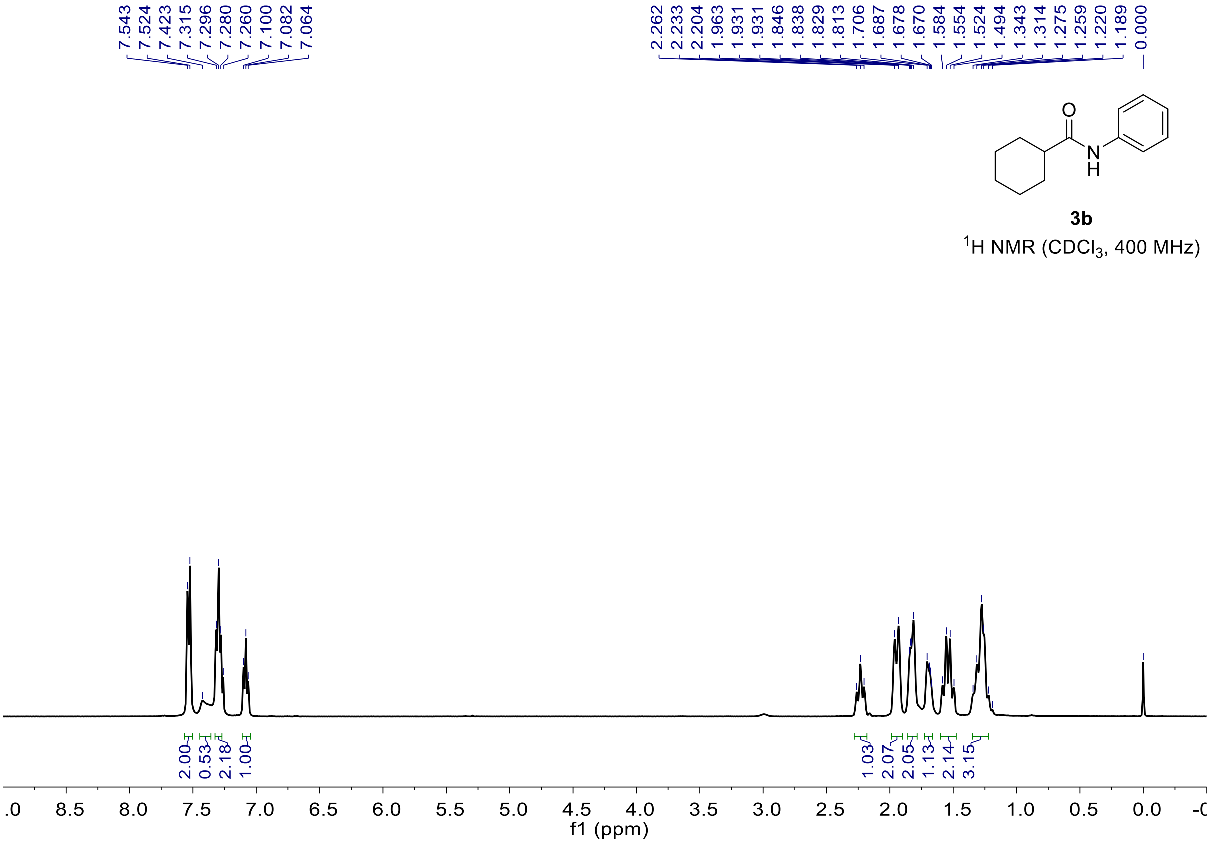

3b

^{1}H NMR ($CDCl_3$, 400 MHz)

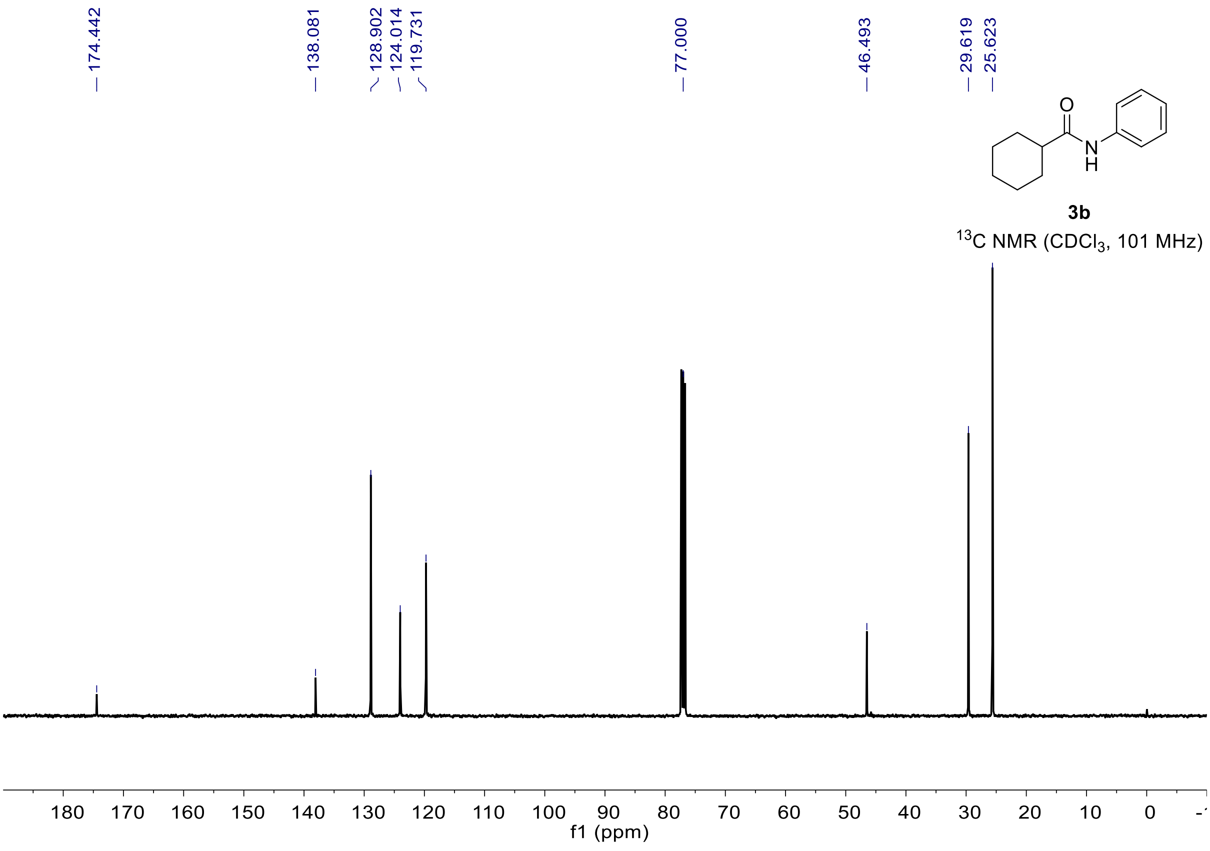

3b

^{13}C NMR ($CDCl_3$, 101 MHz)

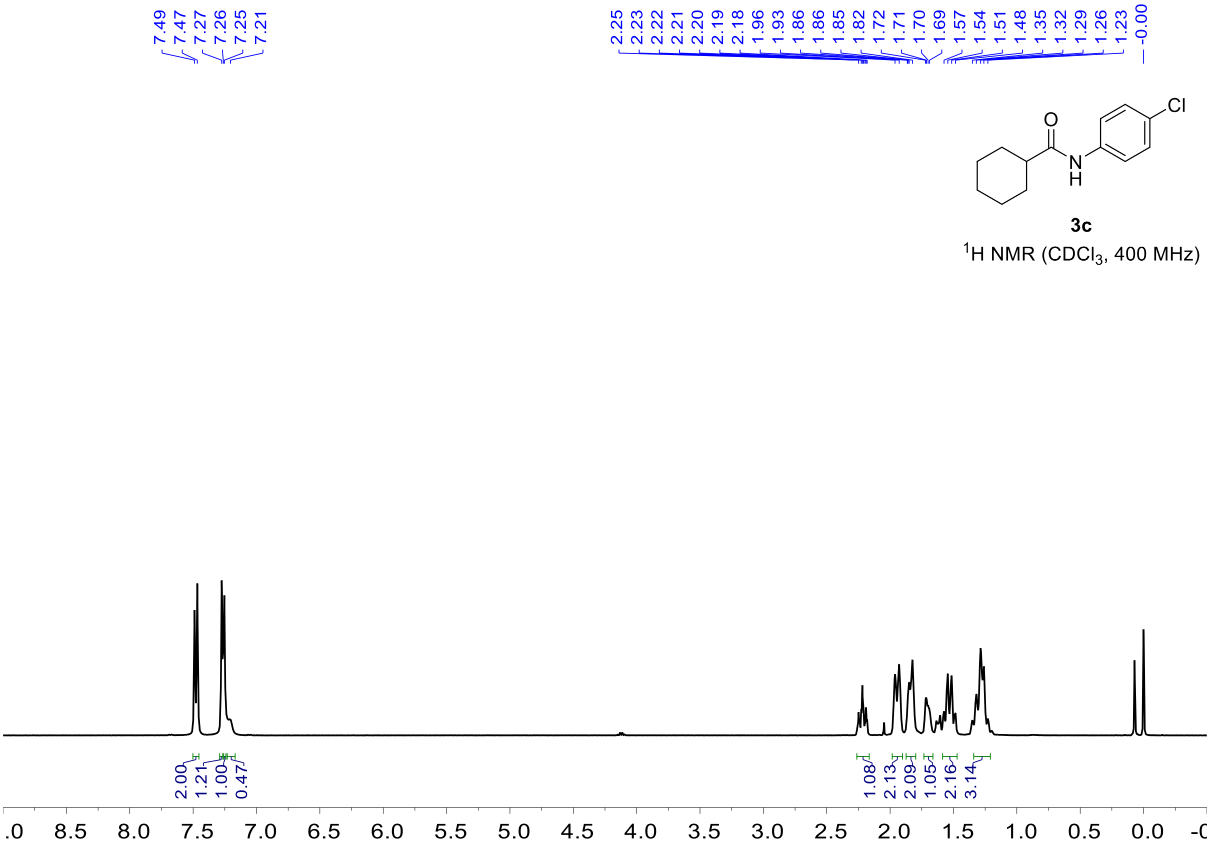

3c

^{1}H NMR ($CDCl_3$, 400 MHz)

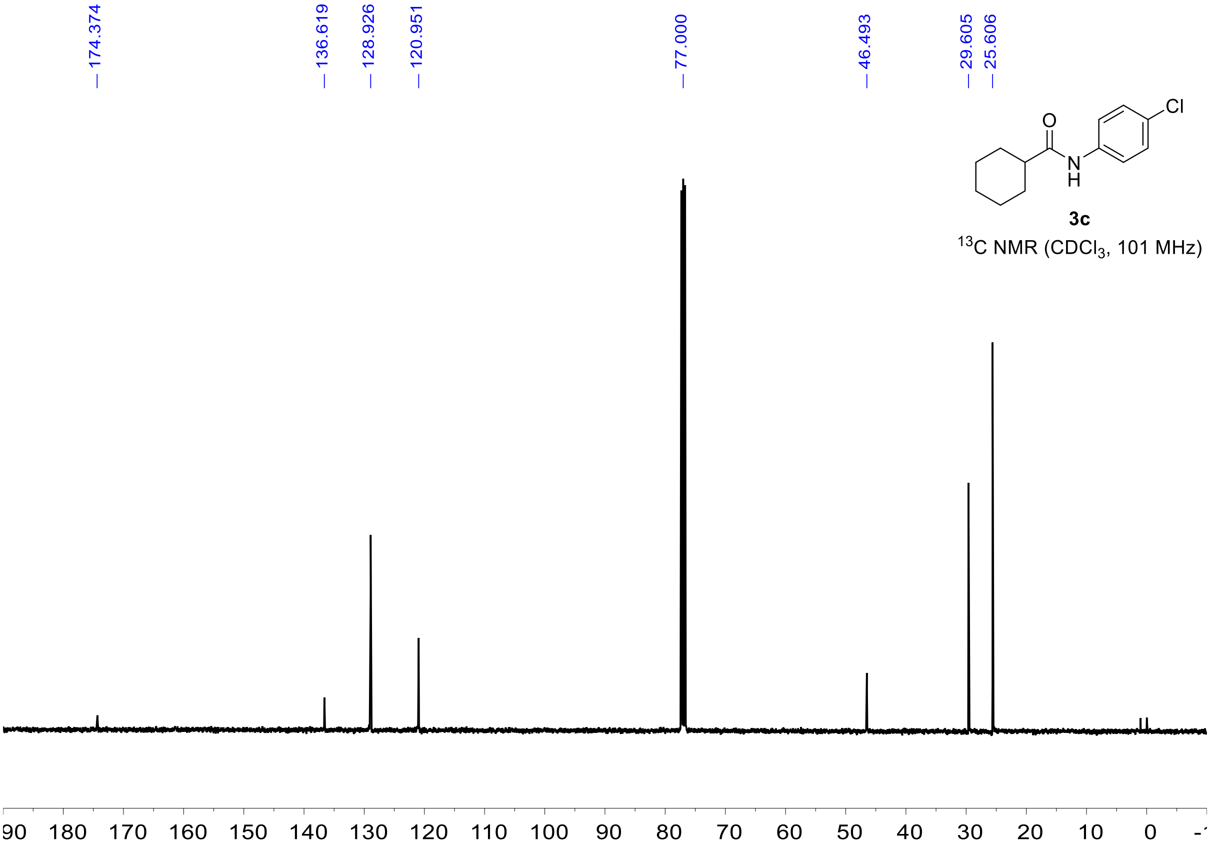

3c

^{13}C NMR ($CDCl_3$, 101 MHz)

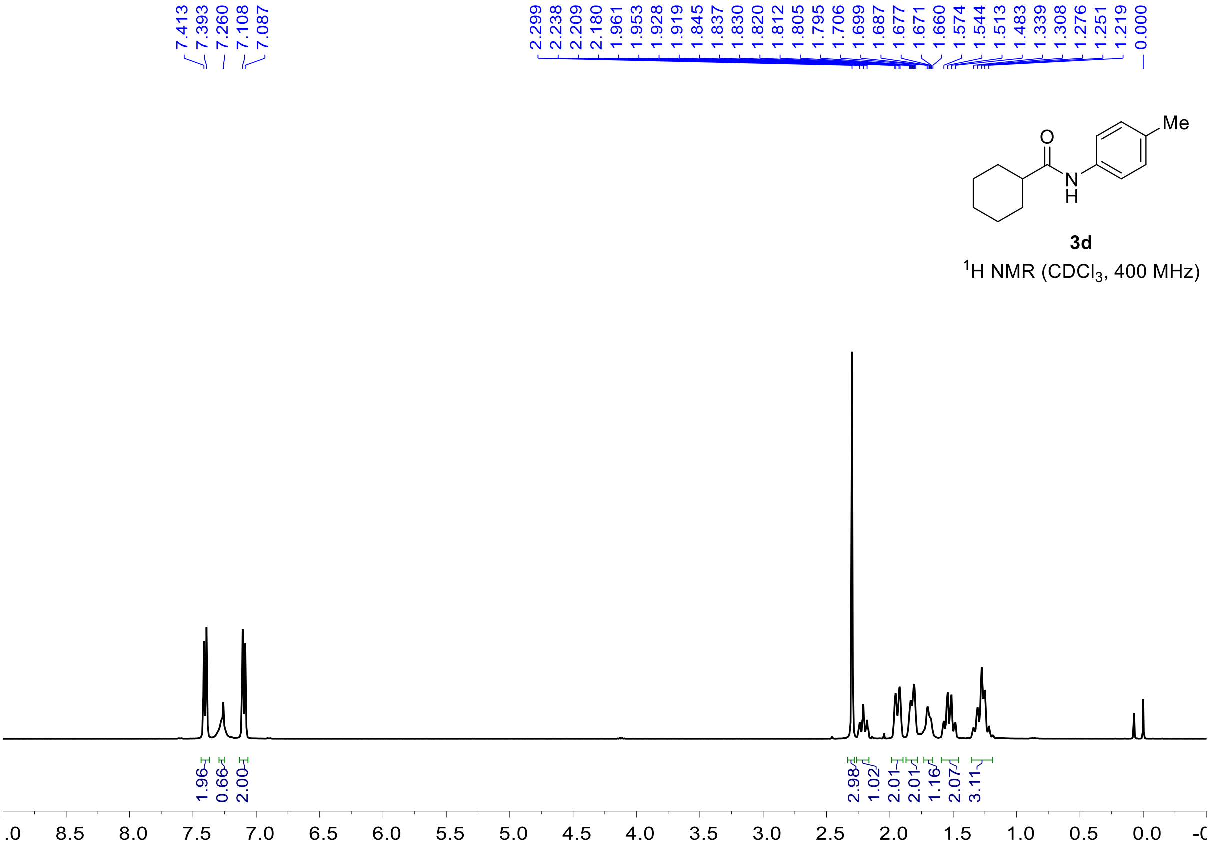

3d

^{1}H NMR ($CDCl_3$, 400 MHz)

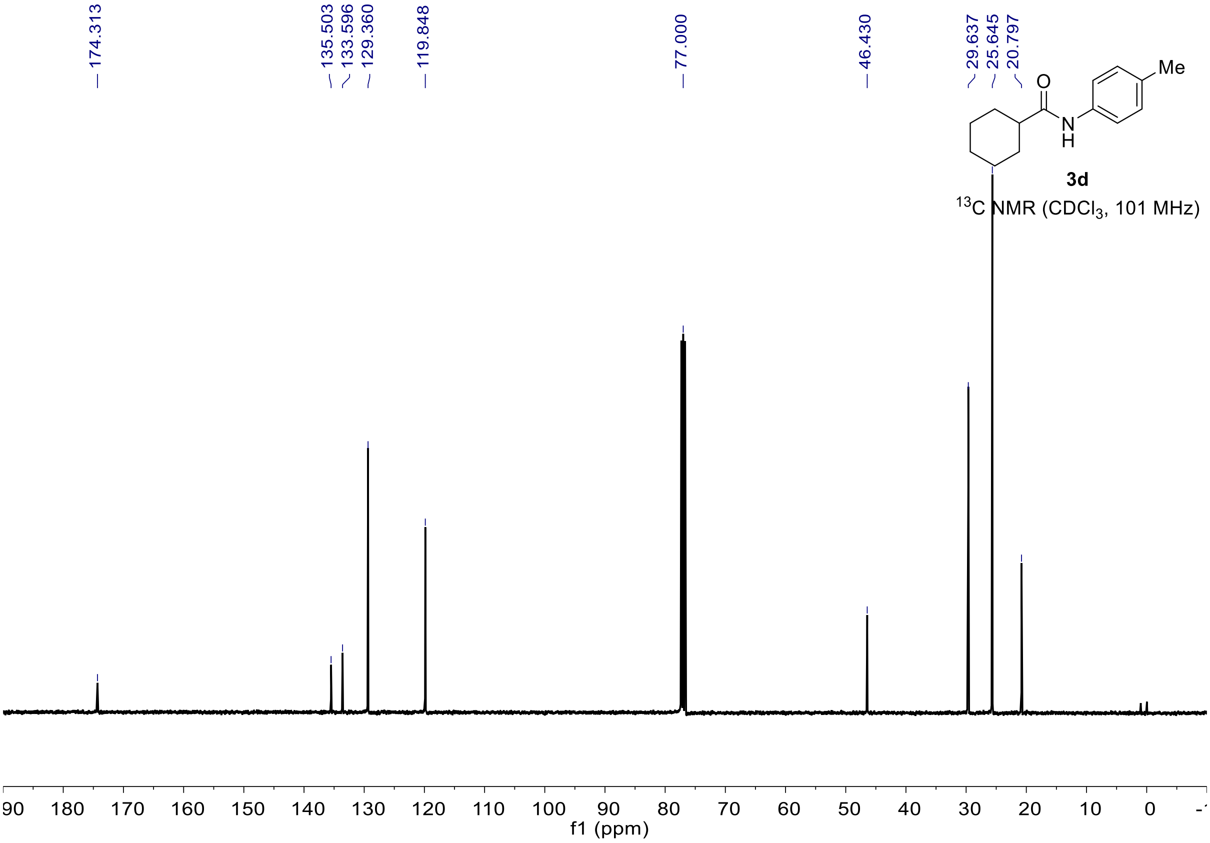

3d

^{13}C NMR ($CDCl_3$, 101 MHz)

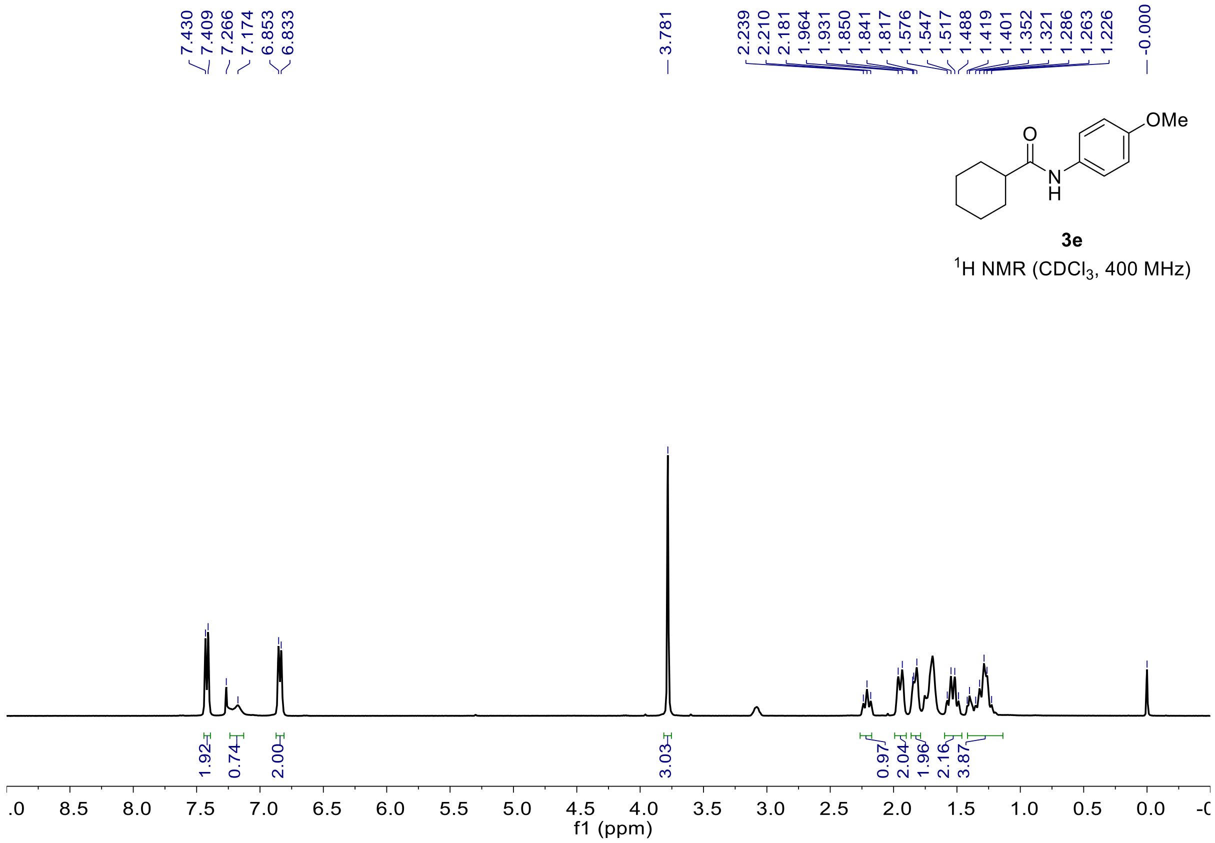
7.430
7.409
7.266
7.174
6.853
6.833
3.781
2.239
2.210
2.181
1.964
1.931
1.850
1.841
1.817
1.576
1.547
1.517
1.488
1.419
1.401
1.352
1.321
1.286
1.263
1.226
-0.000
OMe
O
N
H
3e
1H NMR (CDCl3, 400 MHz)
1.92
0.74
2.00
3.03
0.97
2.04
1.96
2.16
3.87
f1 (ppm)

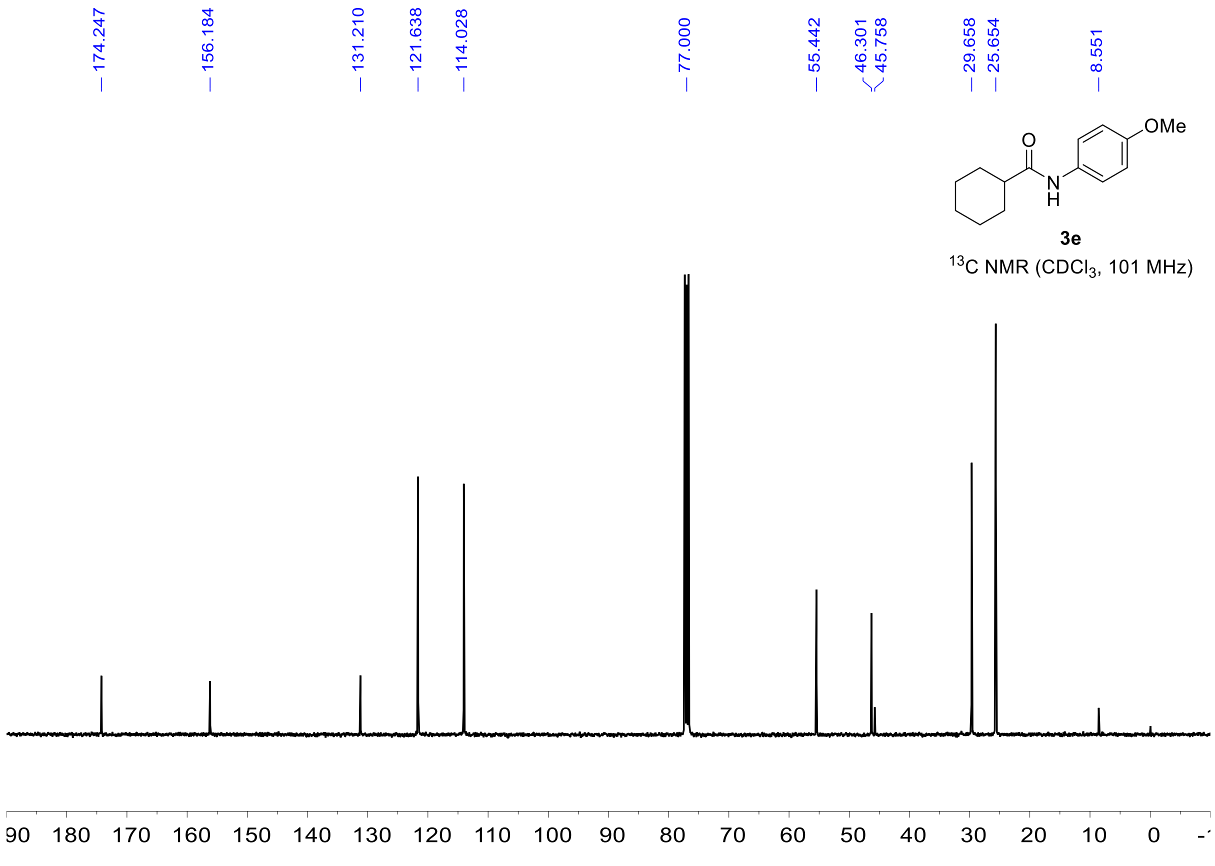
174.247
156.184
131.210
121.638
114.028
77.000
55.442
46.301
45.758
29.658
25.654
8.551
OMe
O
N
H
3e
13C NMR (CDCl3, 101 MHz)

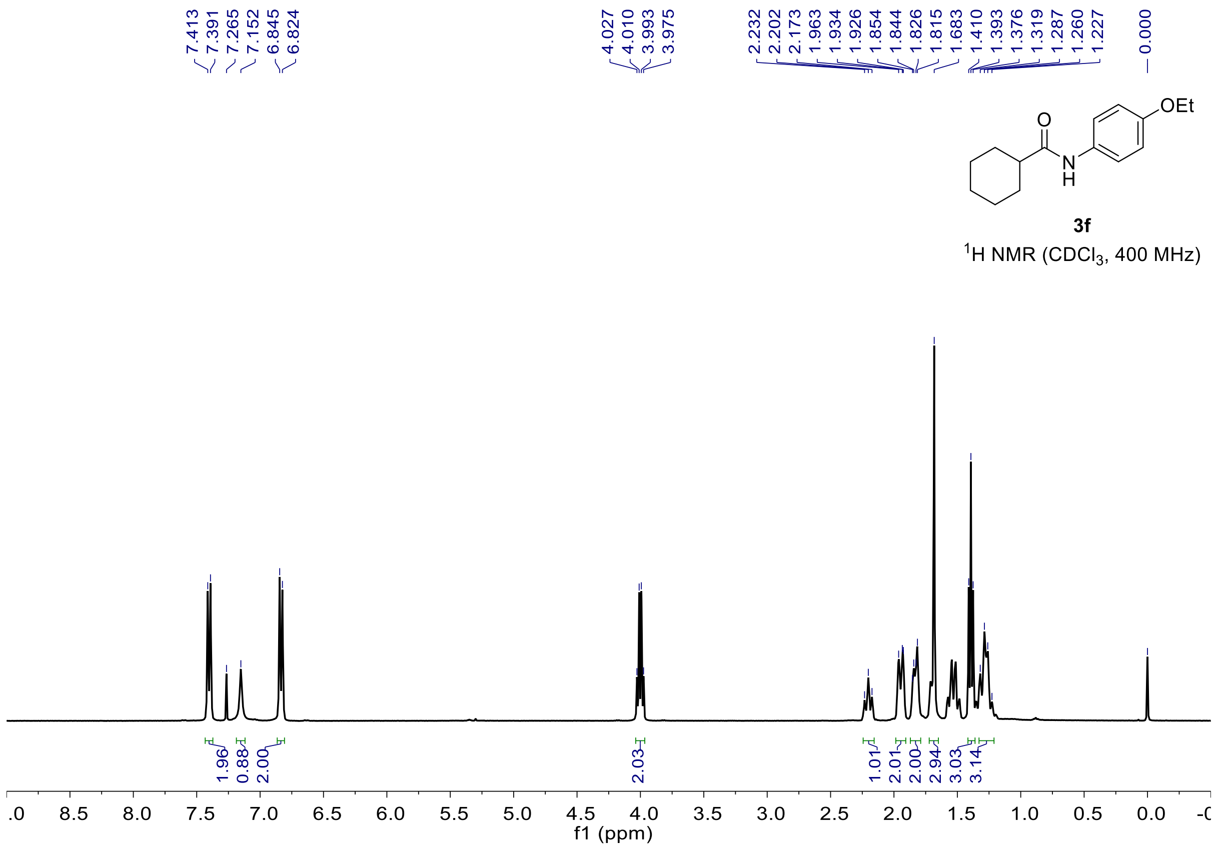

3f

^{1}H NMR (CDCl$_3$, 400 MHz)

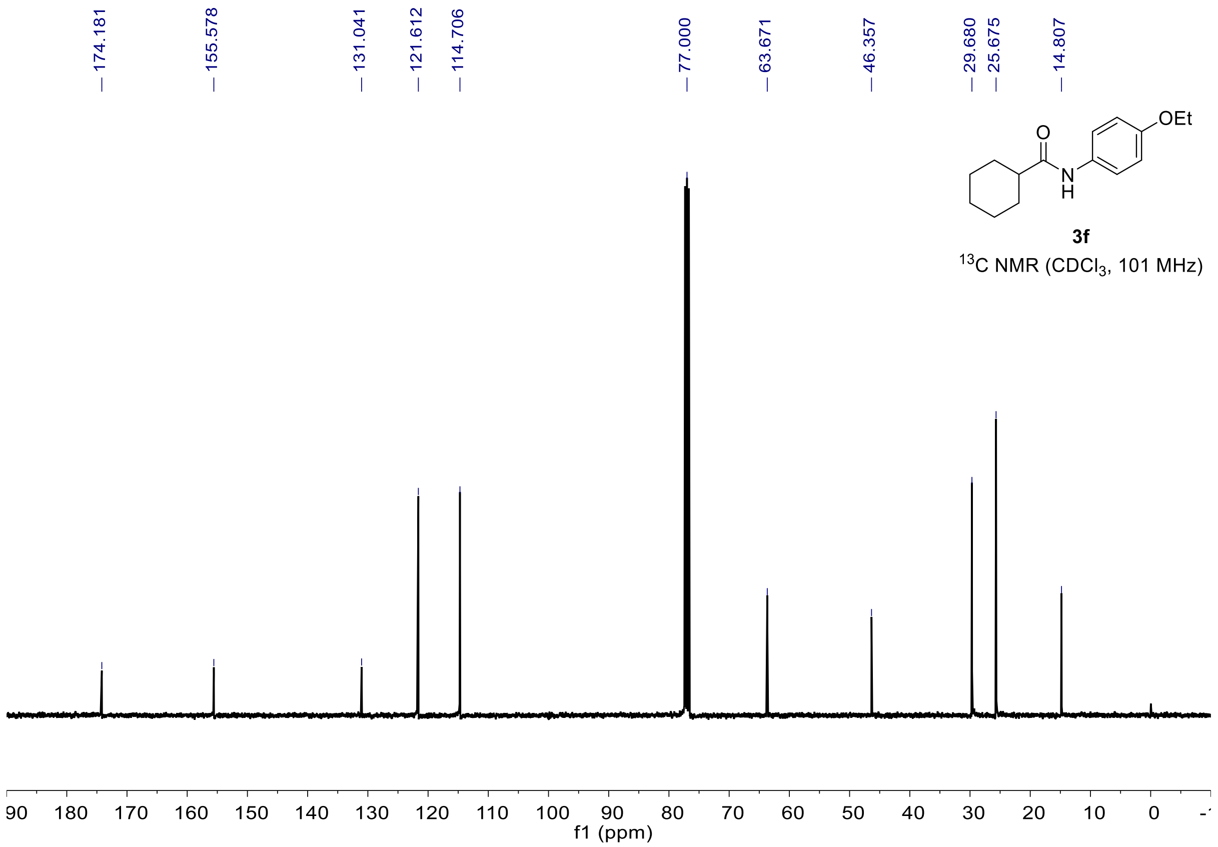

3f

^{13}C NMR (CDCl$_3$, 101 MHz)

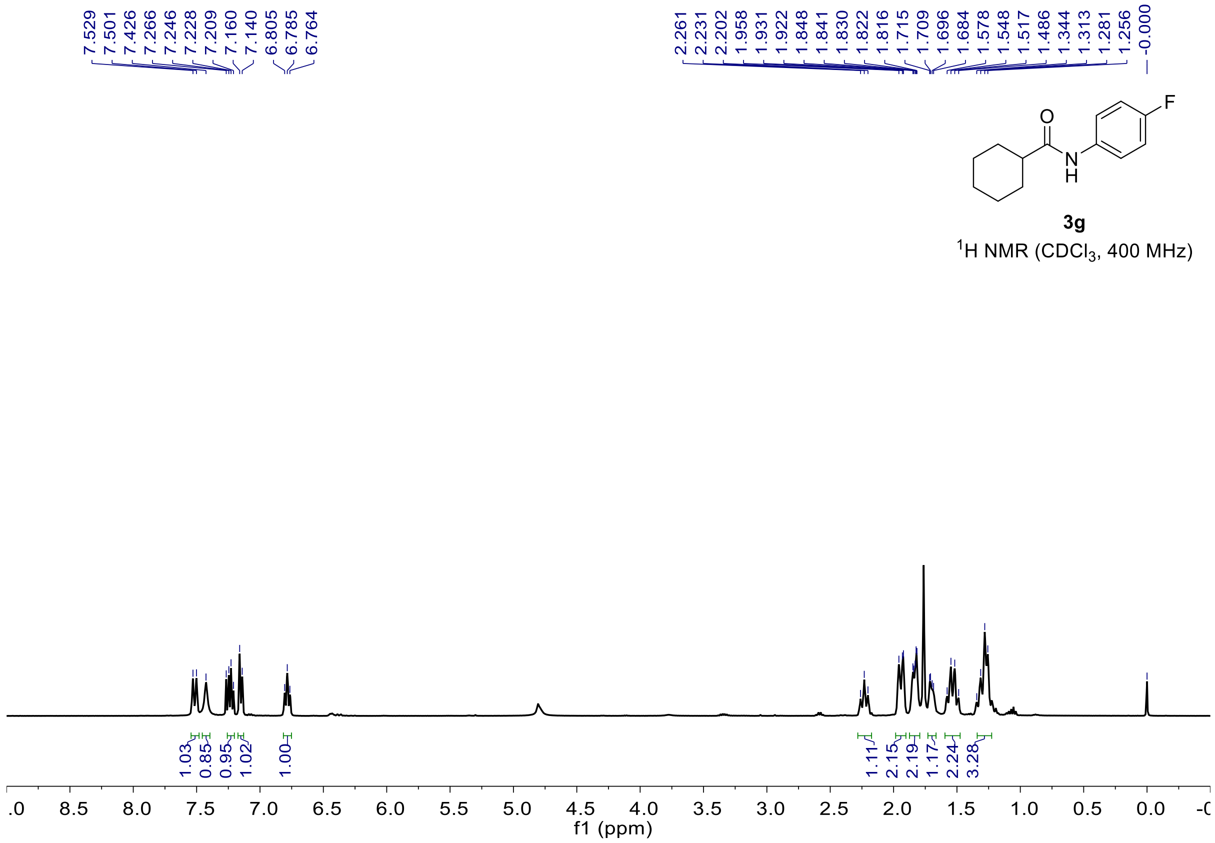
7.529 7.501 7.426 7.266 7.246 7.228 7.209 7.160 7.140 6.805 6.785 6.764
2.261 2.231 2.202 1.958 1.931 1.922 1.848 1.841 1.830 1.822 1.816 1.715 1.709 1.696 1.684 1.578 1.548 1.517 1.486 1.344 1.313 1.281 1.256 -0.000
O
N
H
F
3g
^{1}H NMR (CDCl$_3$, 400 MHz)
1.03 0.85 0.95 1.02 1.00
1.11 2.15 2.19 1.17 2.24 3.28
f1 (ppm)

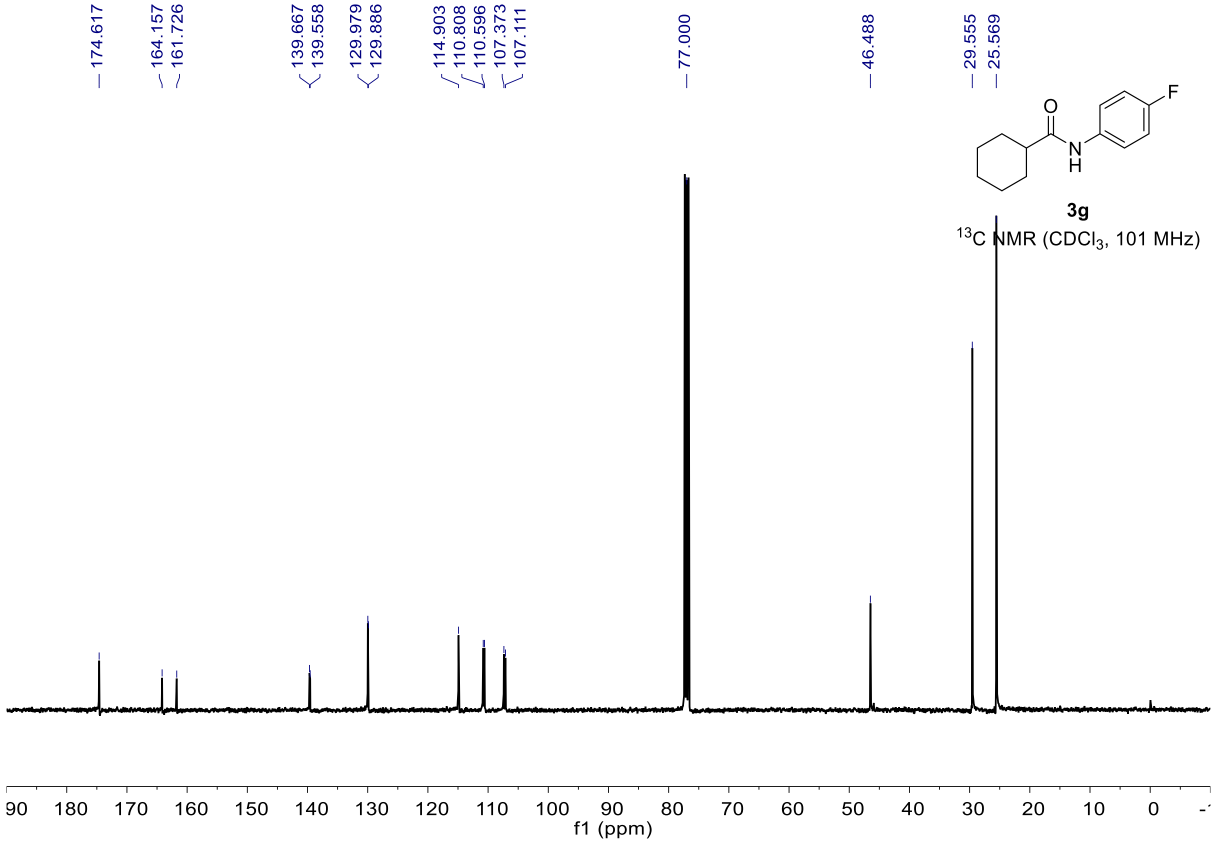
174.617
164.157 161.726
139.667 139.558
129.979 129.886
114.903 110.808 110.596 107.373 107.111
77.000
46.488
29.555 25.569
O
N
H
F
3g
^{13}C NMR (CDCl$_3$, 101 MHz)
f1 (ppm)

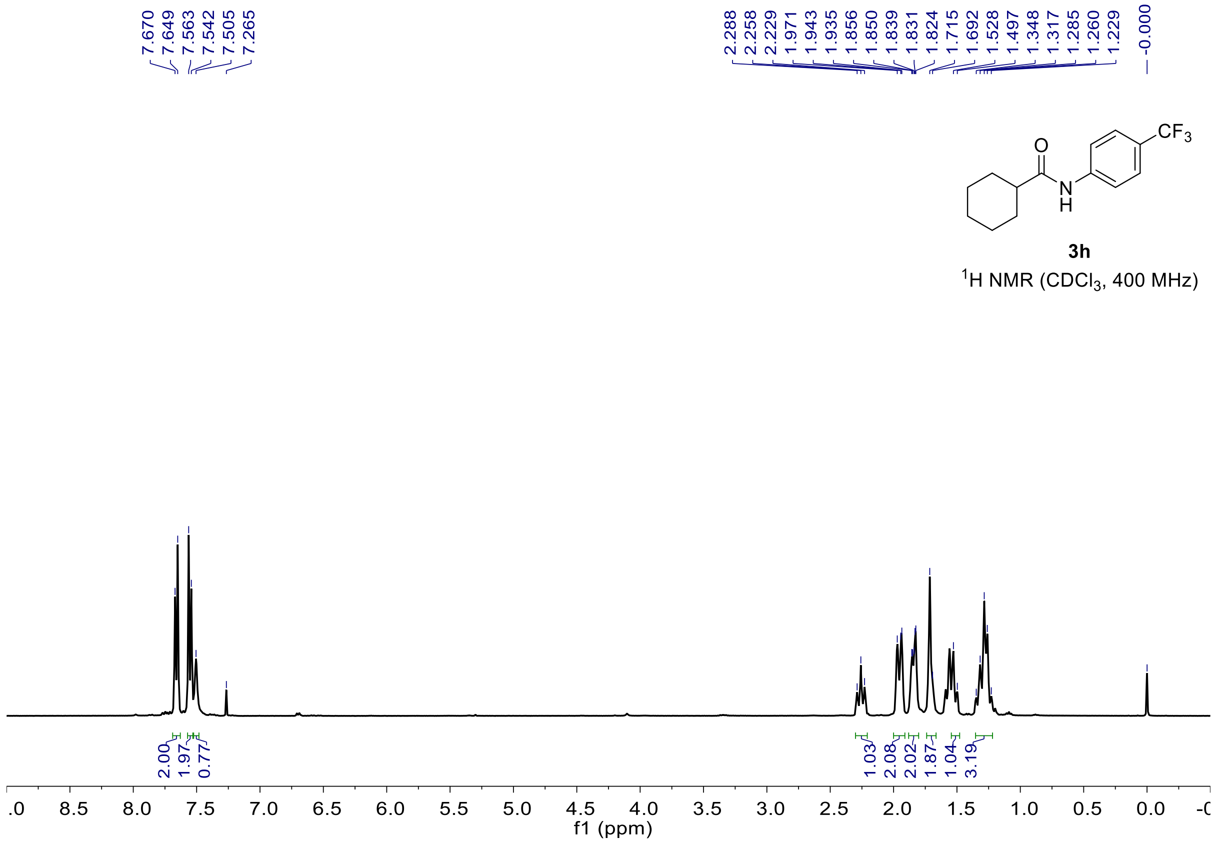

3h

^1H NMR ($CDCl_3$, 400 MHz)

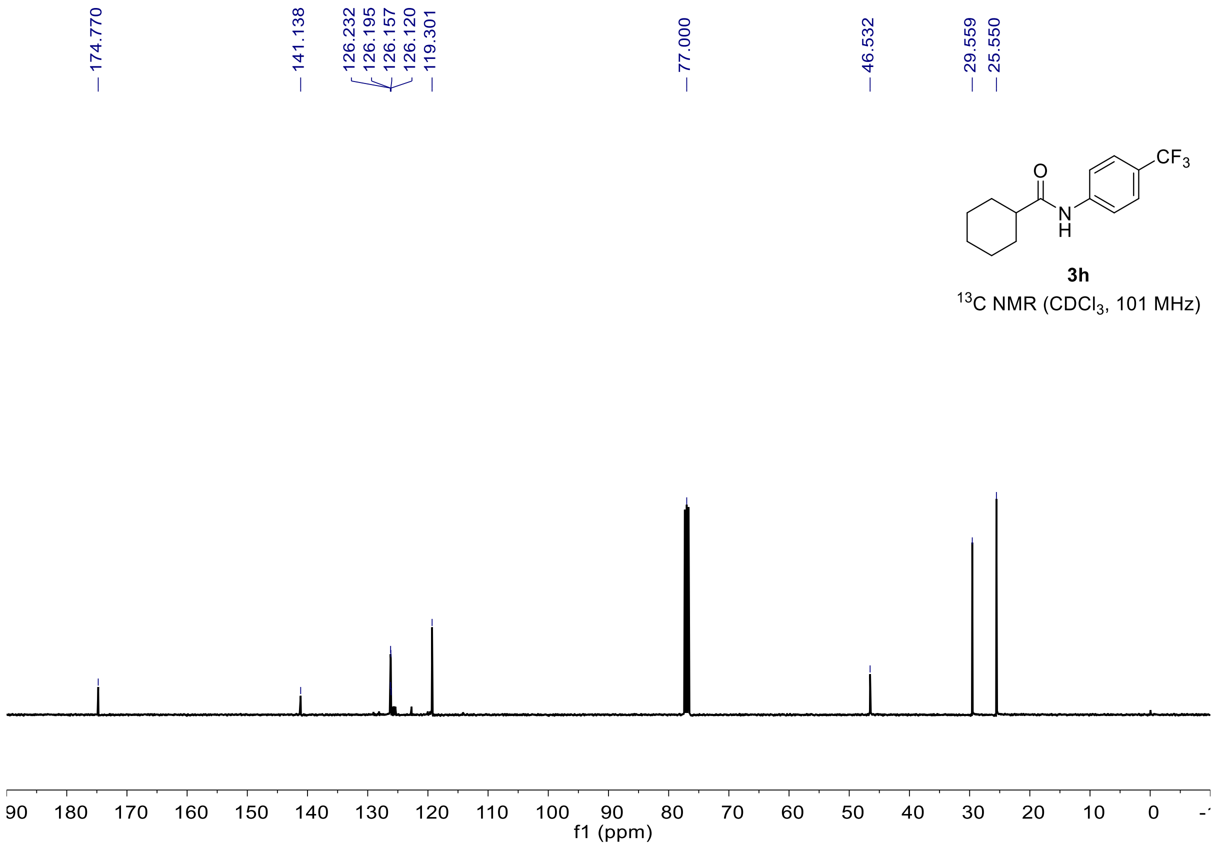

3h

^{13}C NMR ($CDCl_3$, 101 MHz)

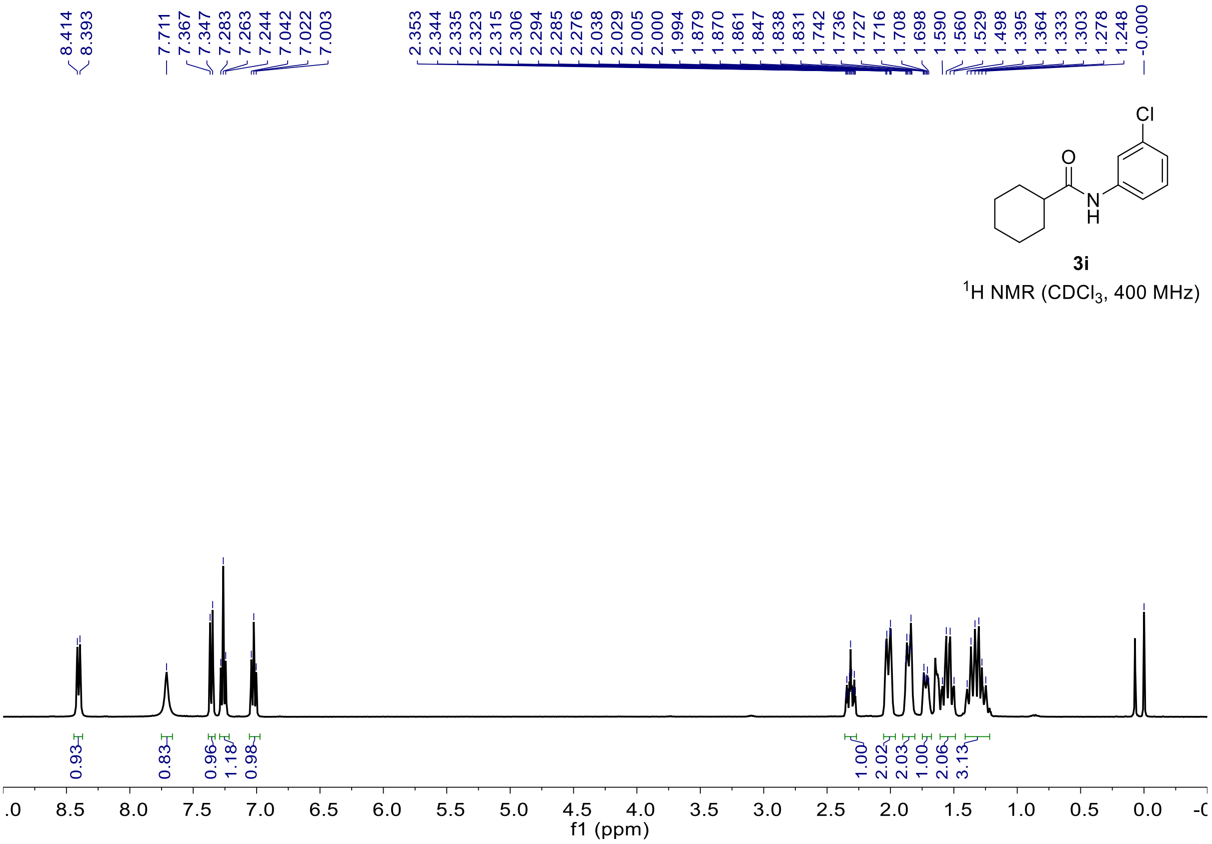
8.414
8.393
7.711
7.367
7.347
7.283
7.263
7.244
7.042
7.022
7.003
2.353
2.344
2.335
2.323
2.315
2.306
2.294
2.285
2.276
2.038
2.029
2.005
2.000
1.994
1.879
1.870
1.861
1.847
1.838
1.831
1.742
1.736
1.727
1.716
1.708
1.698
1.590
1.560
1.529
1.498
1.395
1.364
1.333
1.303
1.278
1.248
-0.000
Cl
O
N
H
3i
1H NMR (CDCl3, 400 MHz)
0.93
0.83
0.96
1.18
0.98
1.00
2.02
2.03
1.00
2.06
3.13
f1 (ppm)

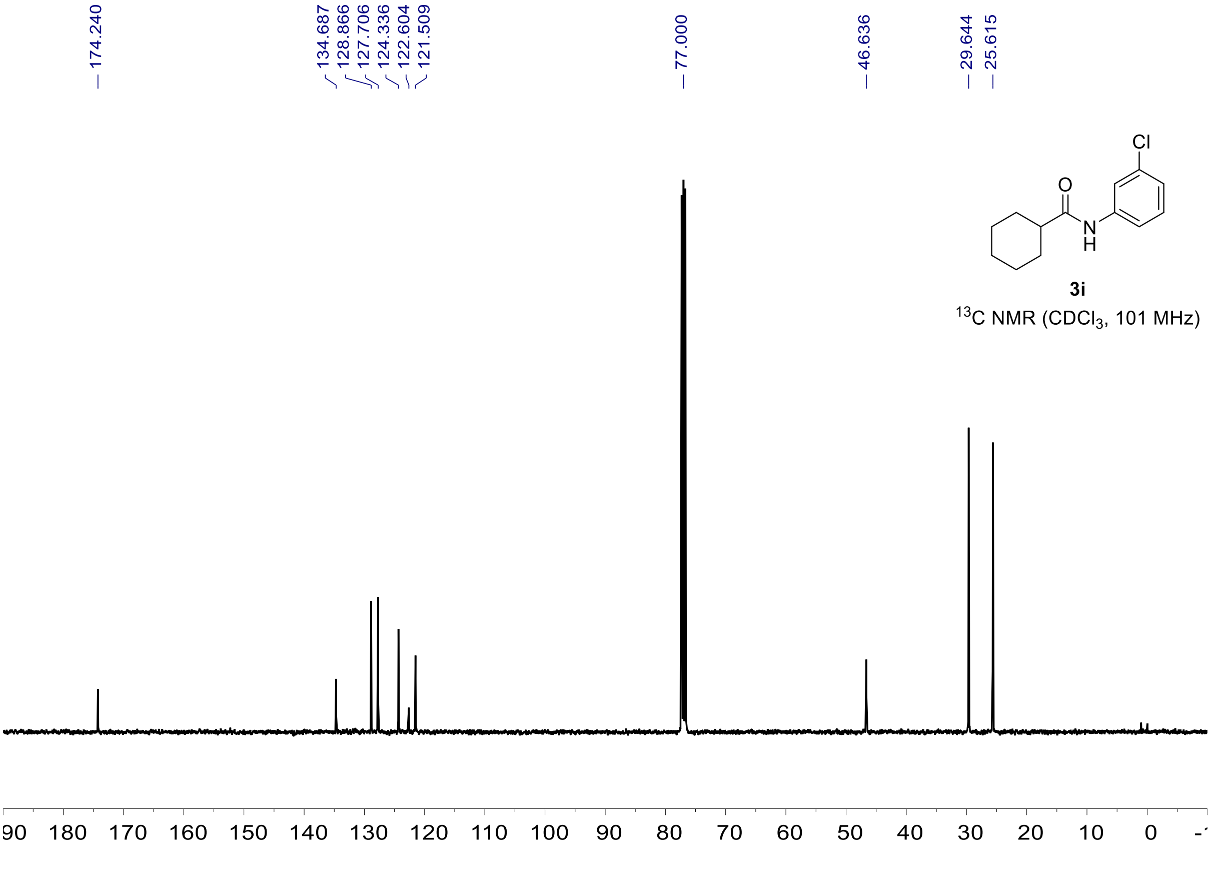
174.240
134.687
128.866
127.706
124.336
122.604
121.509
77.000
46.636
29.644
25.615
Cl
O
N
H
3i
13C NMR (CDCl3, 101 MHz)

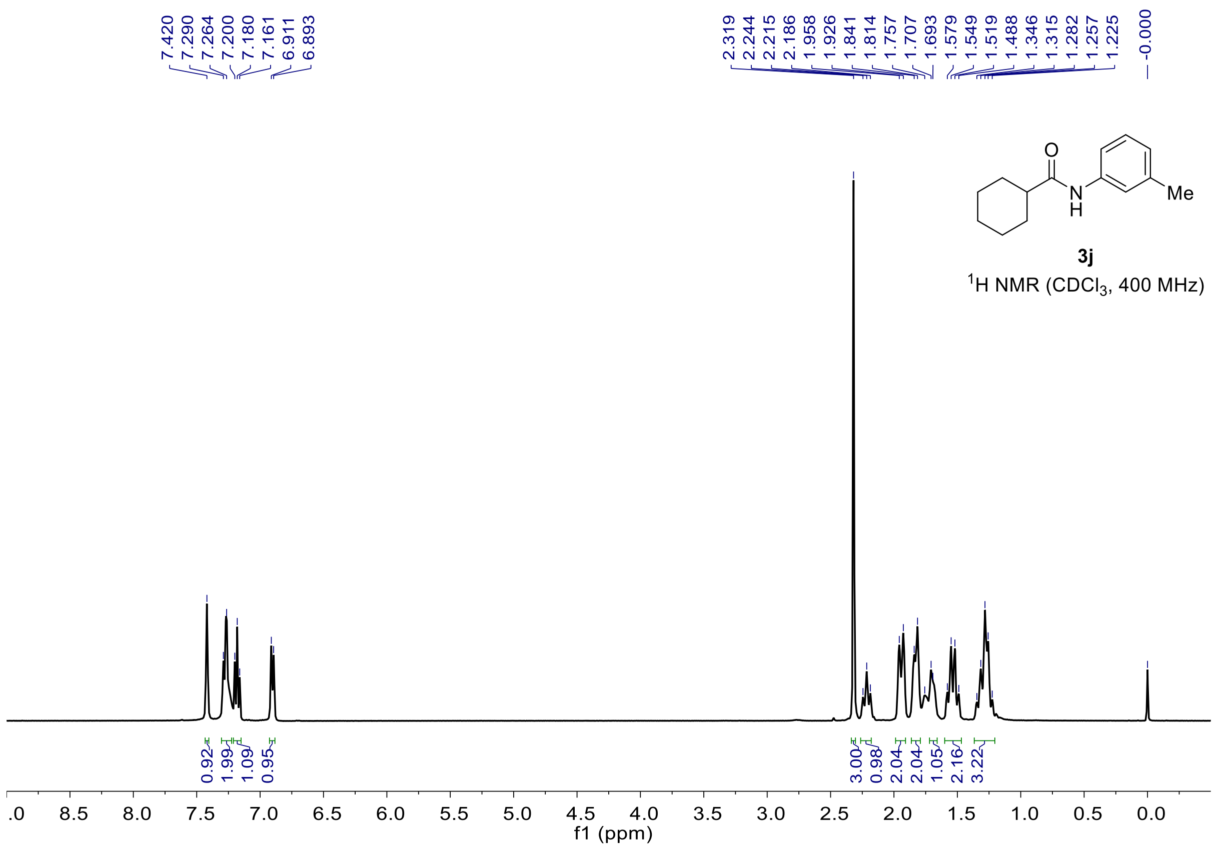

7.420
7.290
7.264
7.200
7.180
7.161
6.911
6.893
2.319
2.244
2.215
2.186
1.958
1.926
1.841
1.814
1.757
1.707
1.693
1.579
1.549
1.519
1.488
1.346
1.315
1.282
1.257
1.225
-0.000
O
N
H
Me
3j
1H NMR (CDCl3, 400 MHz)
0.92
1.99
1.09
0.95
3.00
0.98
2.04
2.04
1.05
2.16
3.22
.0 8.5 8.0 7.5 7.0 6.5 6.0 5.5 5.0 4.5 4.0 3.5 3.0 2.5 2.0 1.5 1.0 0.5 0.0
f1 (ppm)

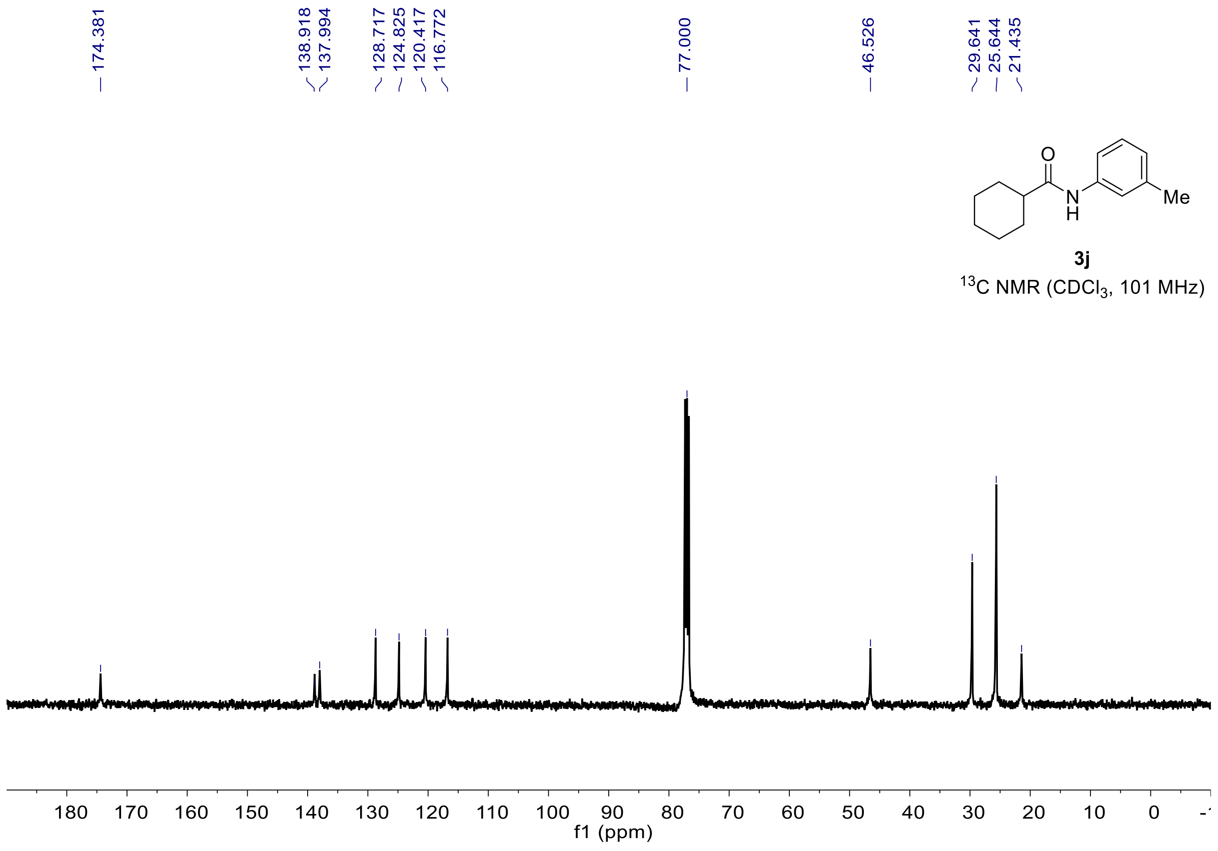

174.381
138.918
137.994
128.717
124.825
120.417
116.772
77.000
46.526
29.641
25.644
21.435
O
N
H
Me
3j
13C NMR (CDCl3, 101 MHz)
180 170 160 150 140 130 120 110 100 90 80 70 60 50 40 30 20 10 0
f1 (ppm)

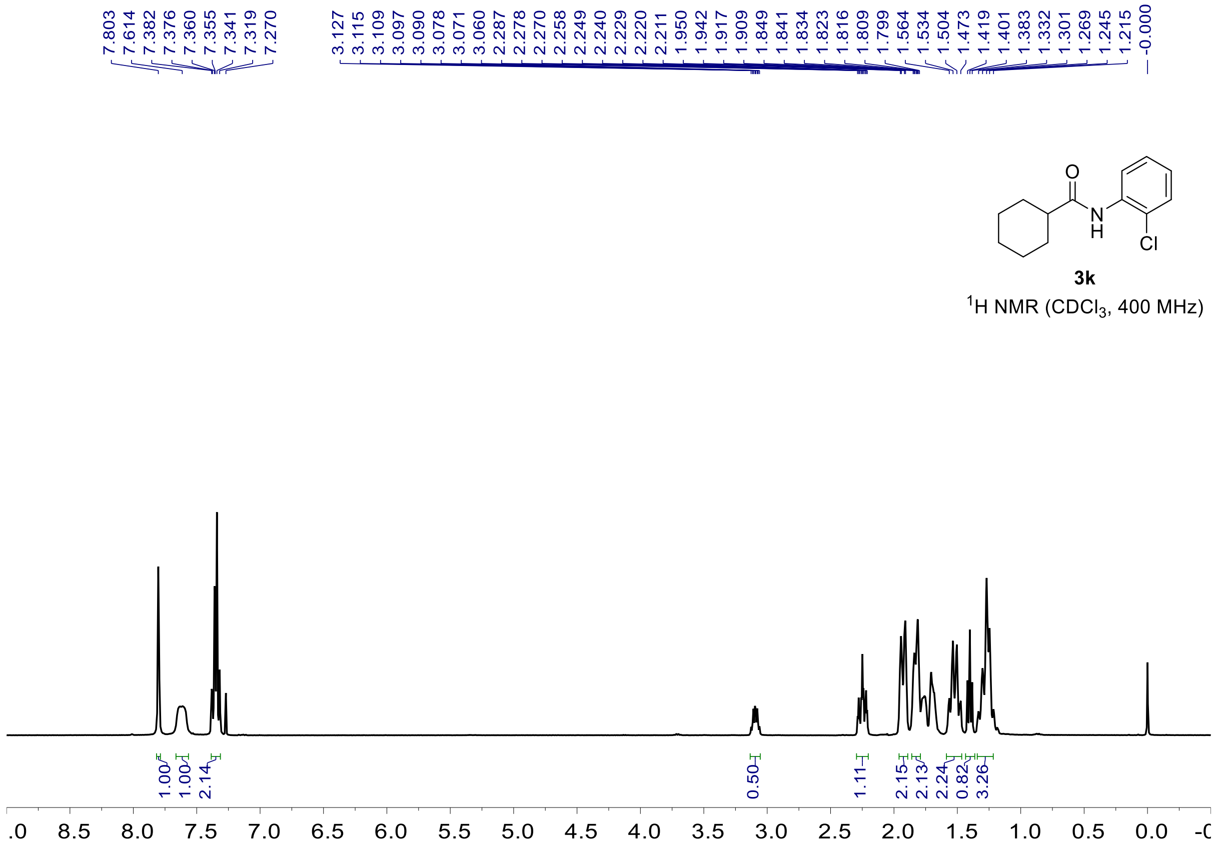

3k

^{1}H NMR ($CDCl_3$, 400 MHz)

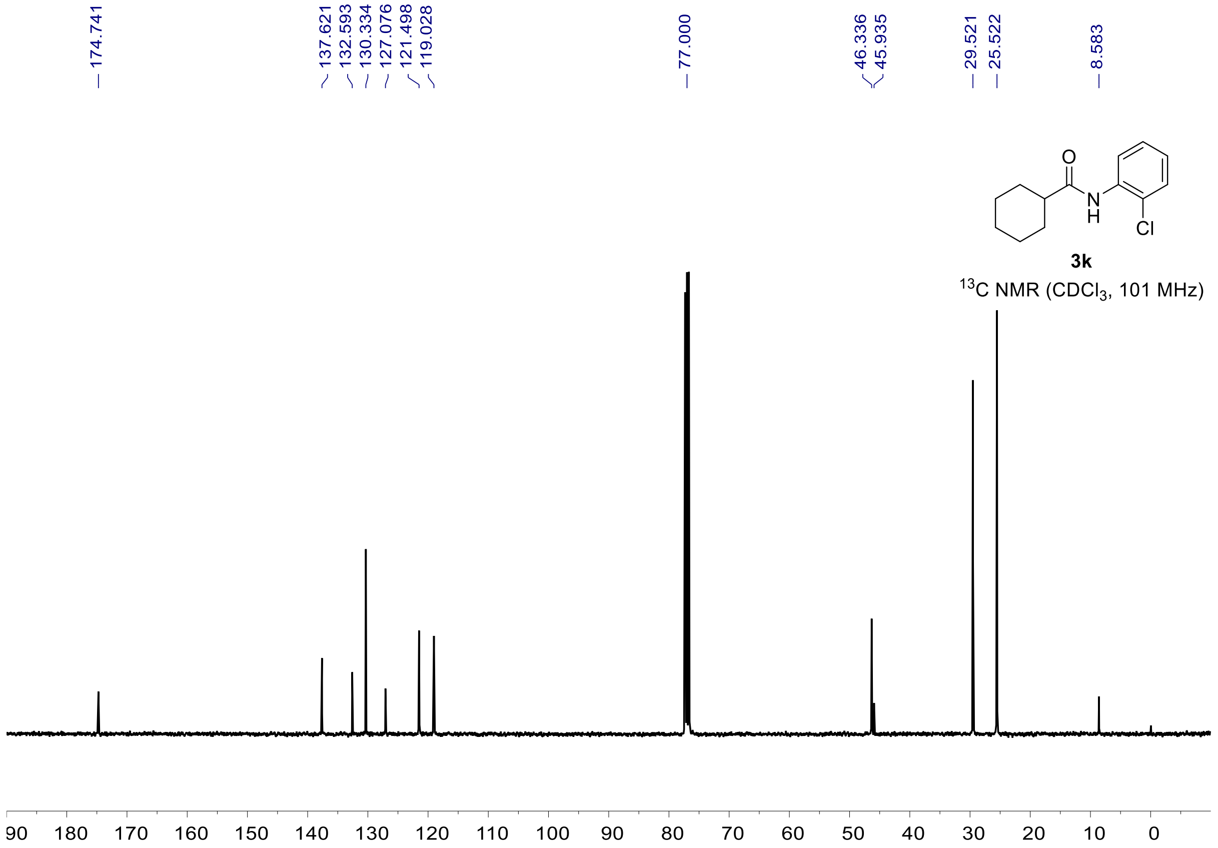

3k

^{13}C NMR ($CDCl_3$, 101 MHz)

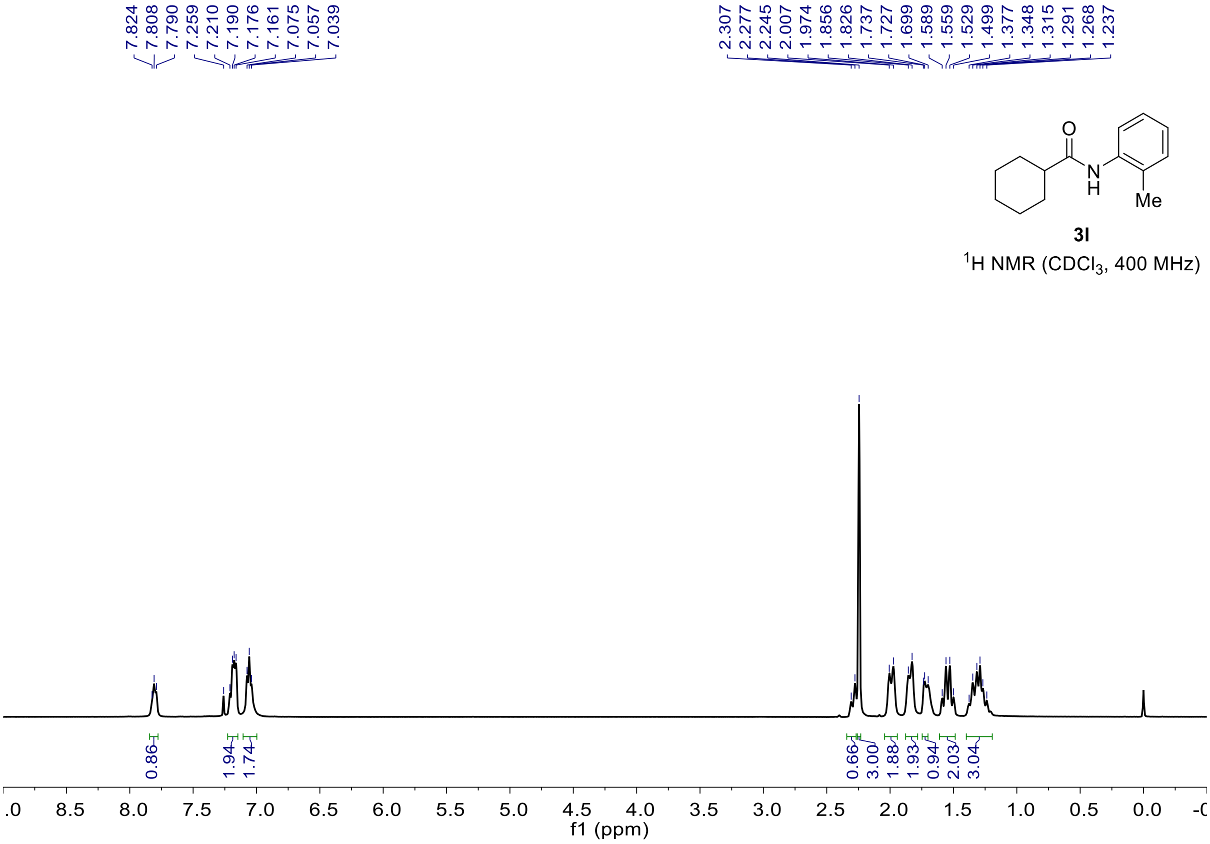
7.824
7.808
7.790
7.259
7.210
7.190
7.176
7.161
7.075
7.057
7.039
2.307
2.277
2.245
2.007
1.974
1.856
1.826
1.737
1.727
1.699
1.589
1.559
1.529
1.499
1.377
1.348
1.315
1.291
1.268
1.237
O
N
H
Me
3l
^{1}H NMR ($CDCl_3$, 400 MHz)
0.86
1.94
1.74
0.66
3.00
1.88
1.93
0.94
2.03
3.04
f1 (ppm)

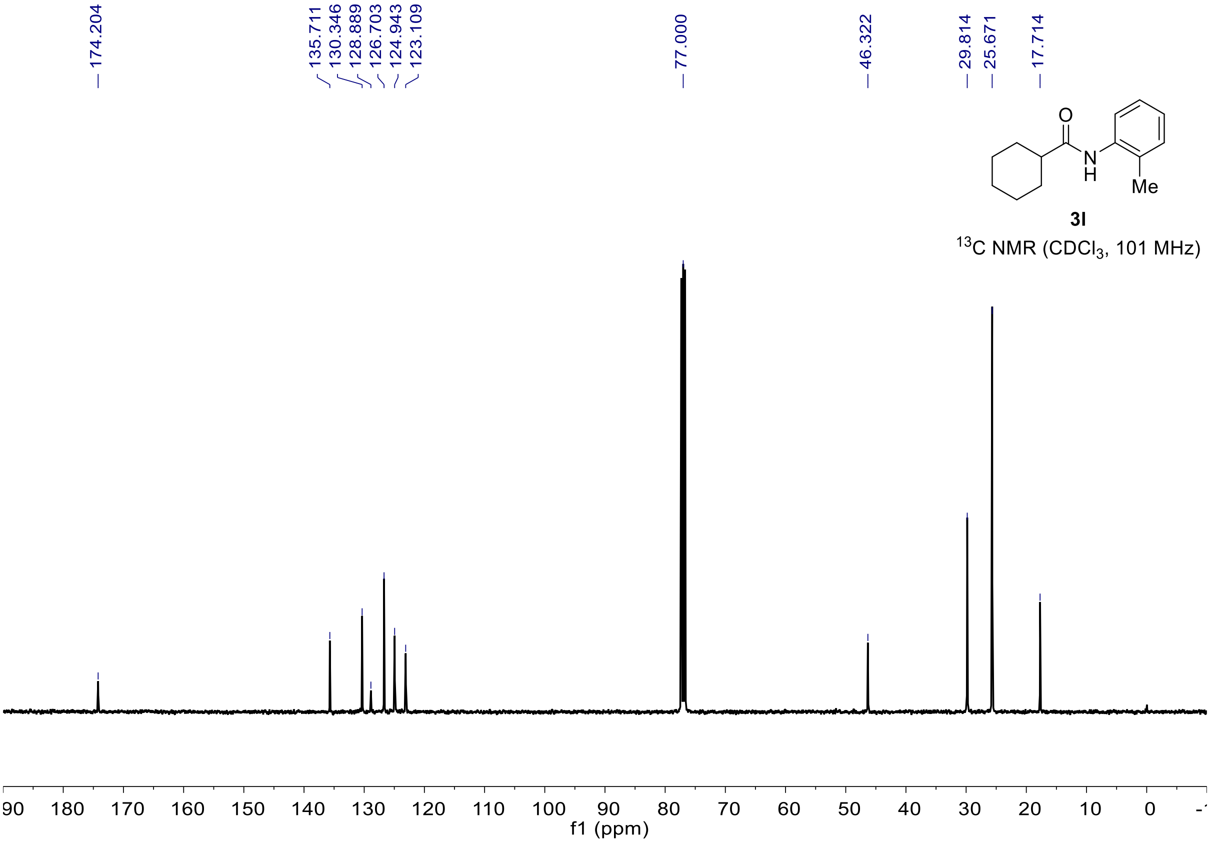
174.204
135.711
130.346
128.889
126.703
124.943
123.109
77.000
46.322
29.814
25.671
17.714
O
N
H
Me
3l
^{13}C NMR ($CDCl_3$, 101 MHz)
f1 (ppm)

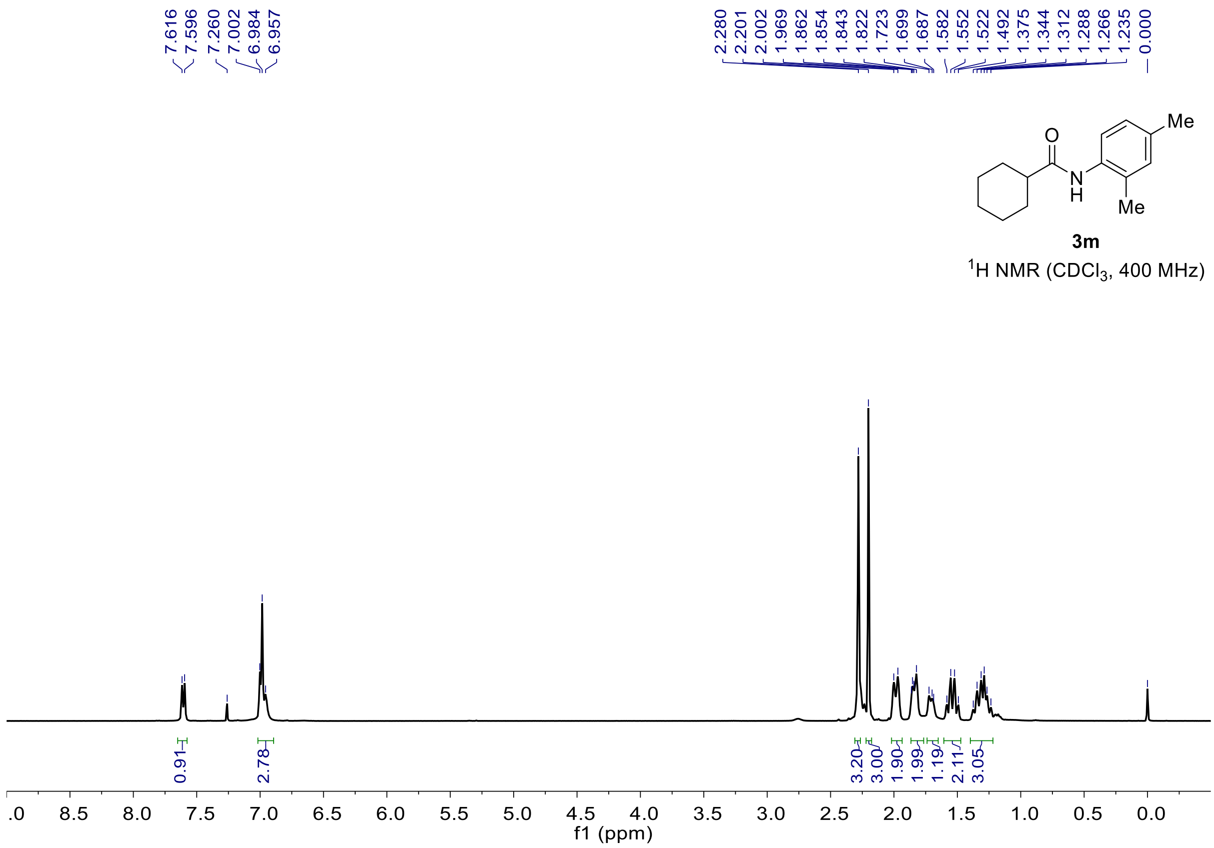
7.616
7.596
7.260
7.002
6.984
6.957
2.280
2.201
2.002
1.969
1.862
1.854
1.843
1.822
1.723
1.699
1.687
1.582
1.552
1.522
1.492
1.375
1.344
1.312
1.288
1.266
1.235
0.000
Me
O
N
H
Me
3m
1H NMR (CDCl3, 400 MHz)
0.91
2.78
3.20
3.00
1.90
1.99
1.19
2.11
3.05
f1 (ppm)

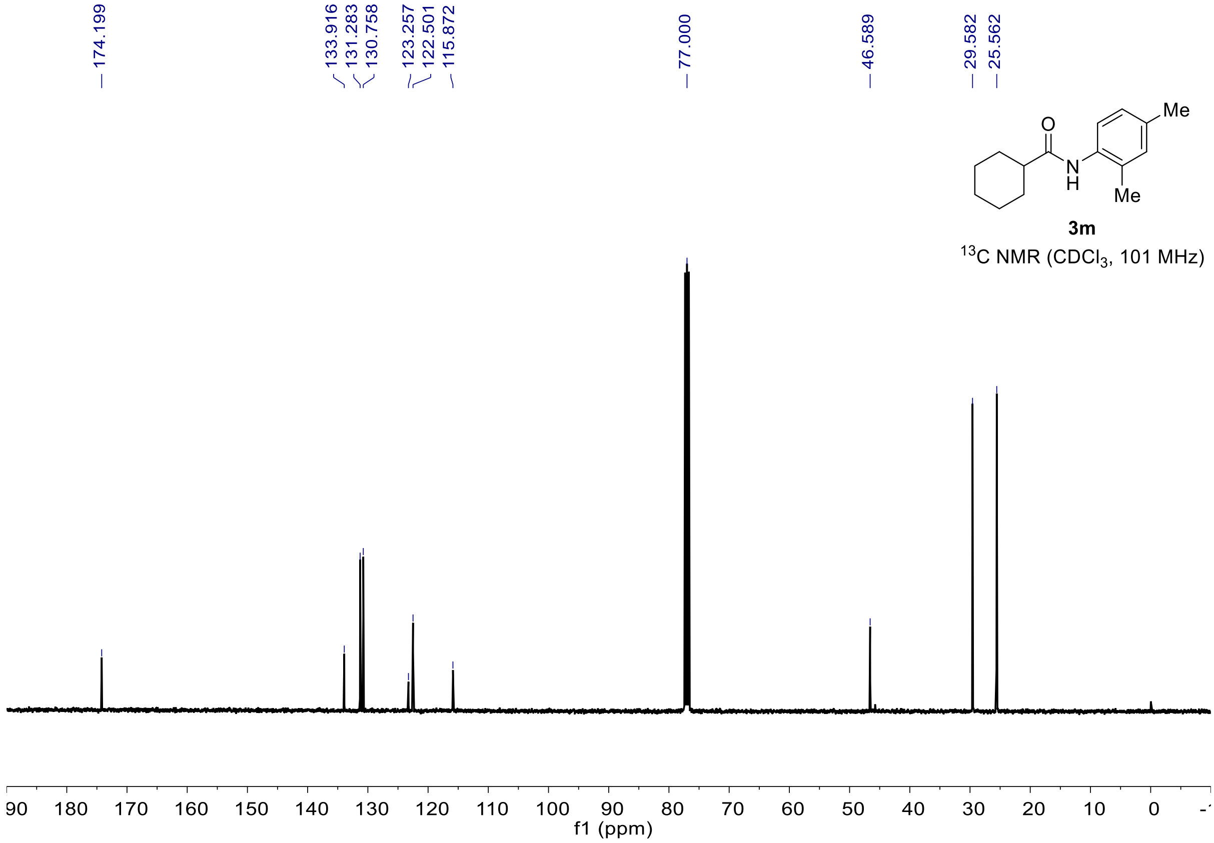
174.199
133.916
131.283
130.758
123.257
122.501
115.872
77.000
46.589
29.582
25.562
Me
O
N
H
Me
3m
13C NMR (CDCl3, 101 MHz)
f1 (ppm)

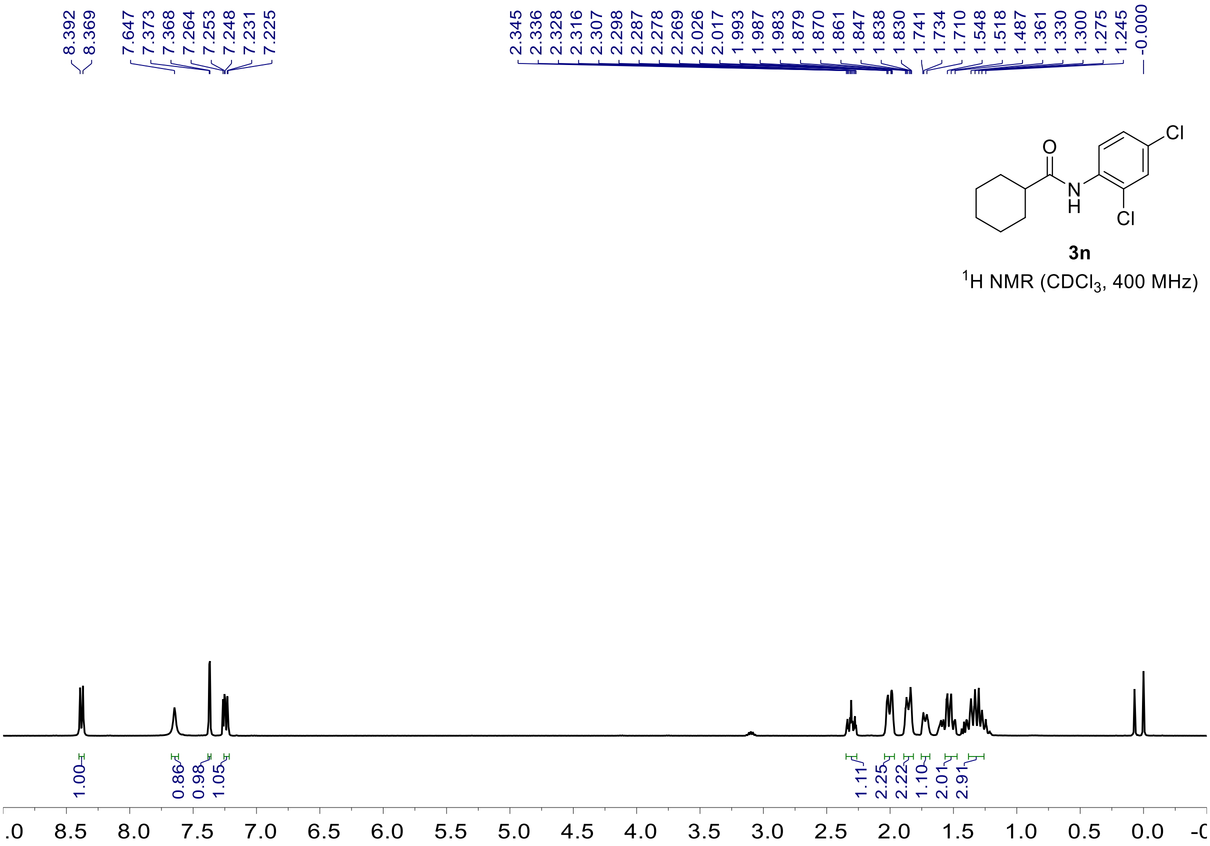

3n

^{1}H NMR ($CDCl_3$, 400 MHz)

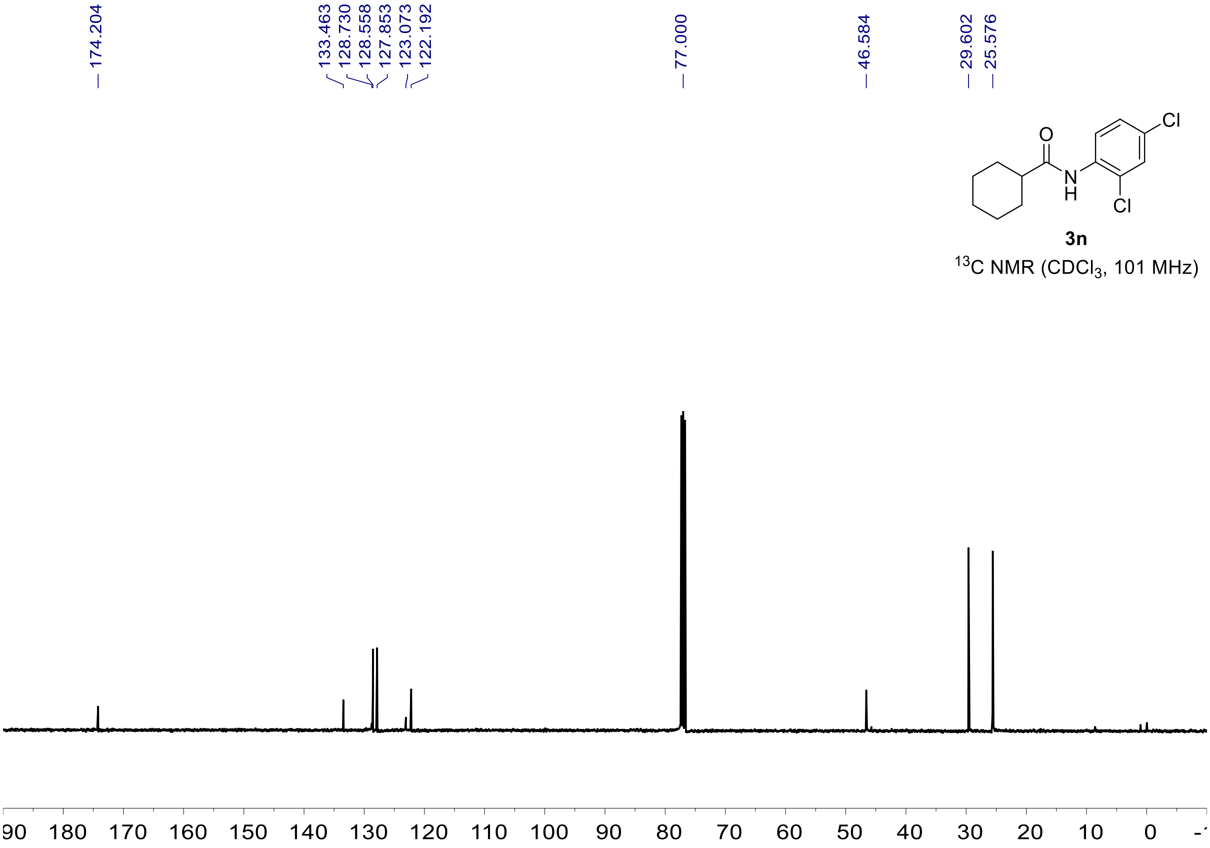

3n

^{13}C NMR ($CDCl_3$, 101 MHz)

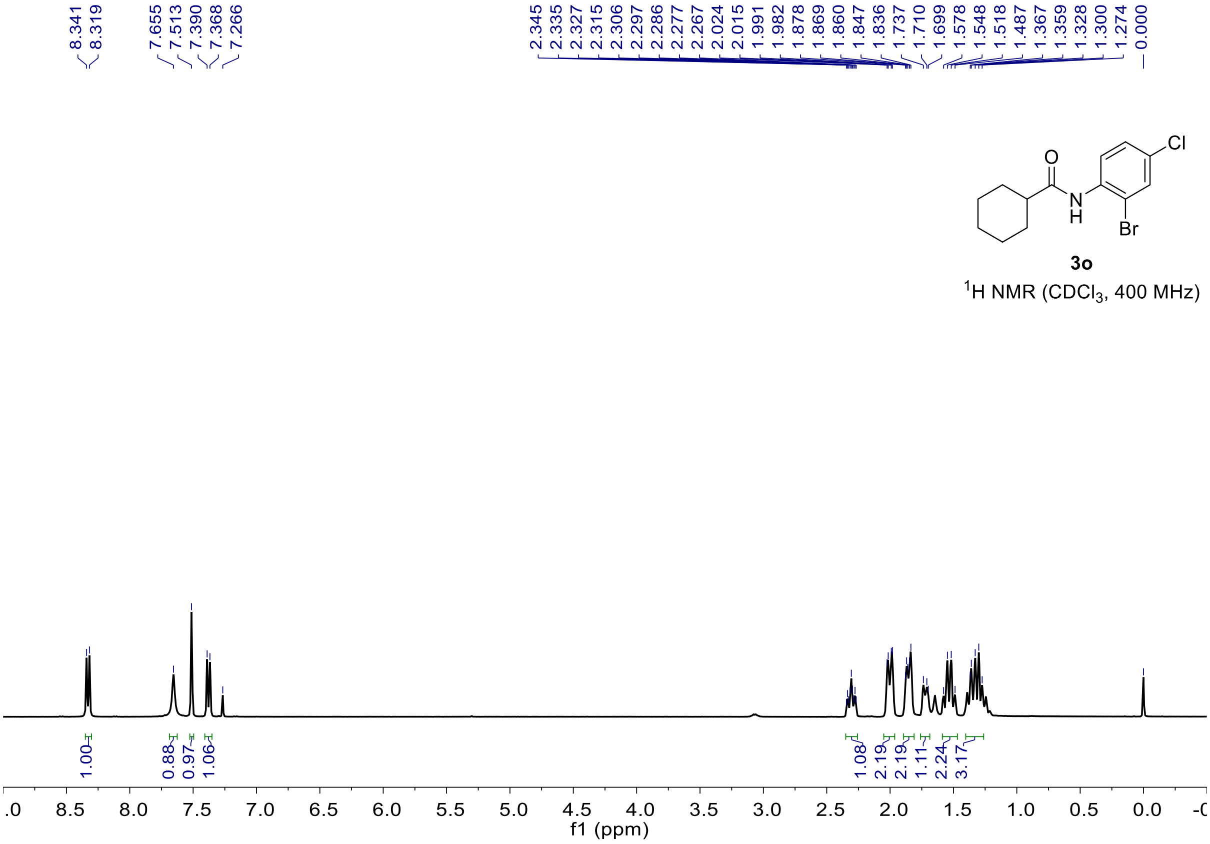

3o

^{1}H NMR ($CDCl_3$, 400 MHz)

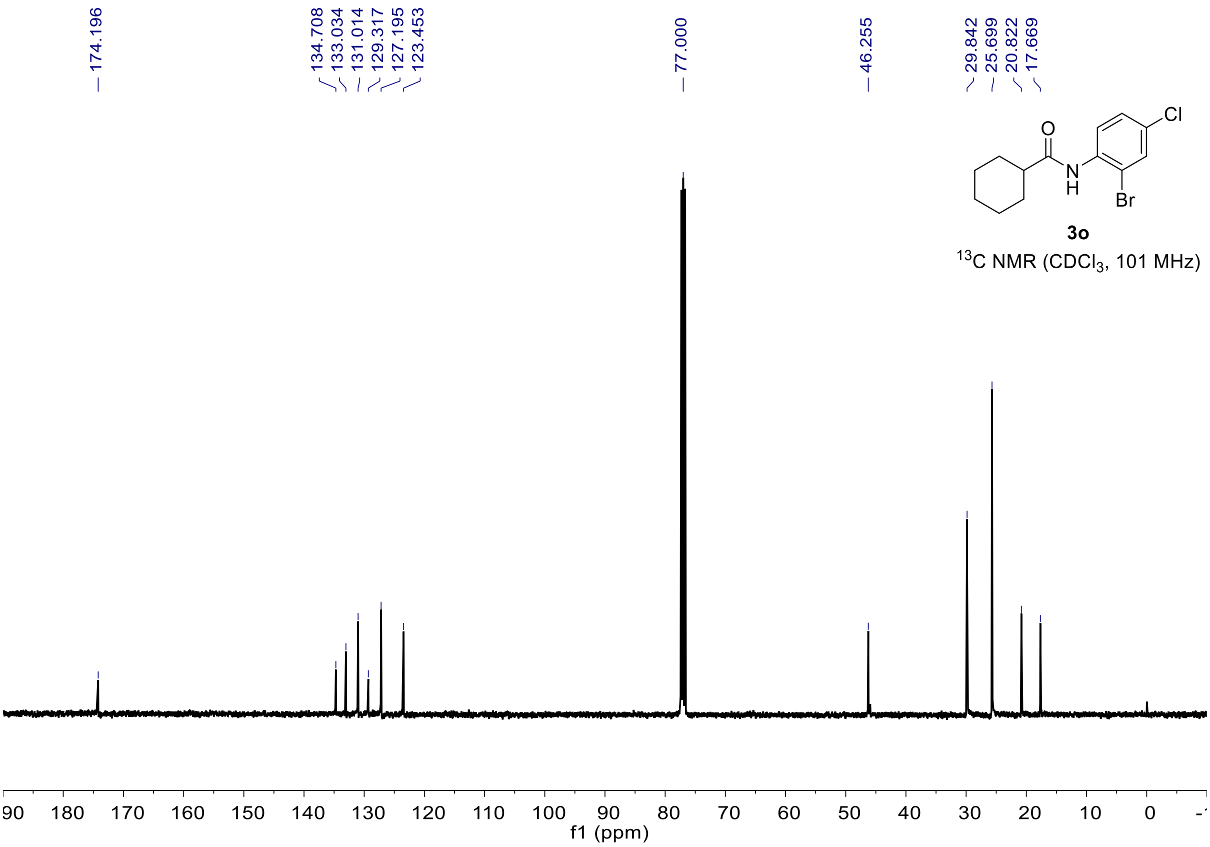

3o

^{13}C NMR ($CDCl_3$, 101 MHz)

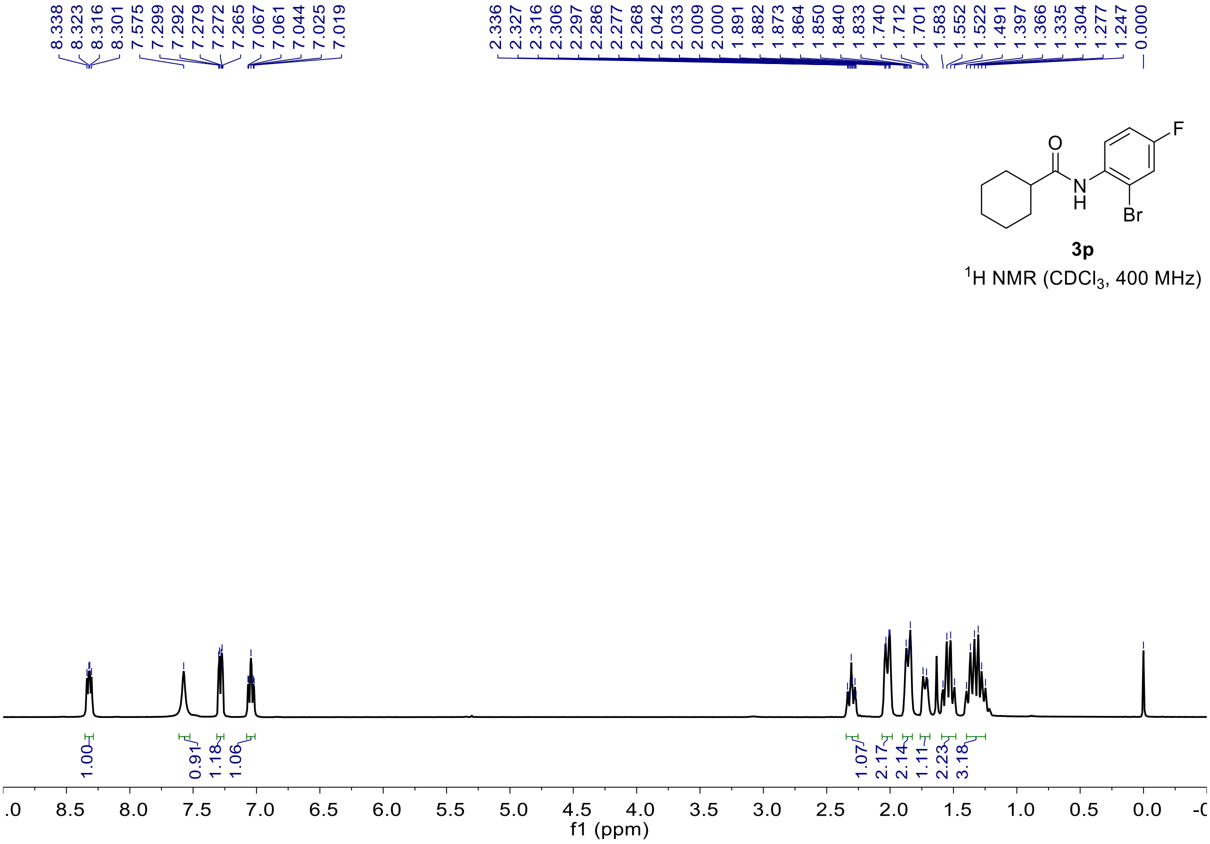
8.338
8.323
8.316
8.301
7.575
7.299
7.292
7.279
7.272
7.265
7.067
7.061
7.044
7.025
7.019
2.336
2.327
2.316
2.306
2.297
2.286
2.277
2.268
2.042
2.033
2.009
2.000
1.891
1.882
1.873
1.864
1.850
1.840
1.833
1.740
1.712
1.701
1.583
1.552
1.522
1.491
1.397
1.366
1.335
1.304
1.277
1.247
0.000
O
N
H
F
Br
3p
1H NMR (CDCl3, 400 MHz)
1.00
0.91
1.18
1.06
1.07
2.17
2.14
1.11
2.23
3.18
f1 (ppm)

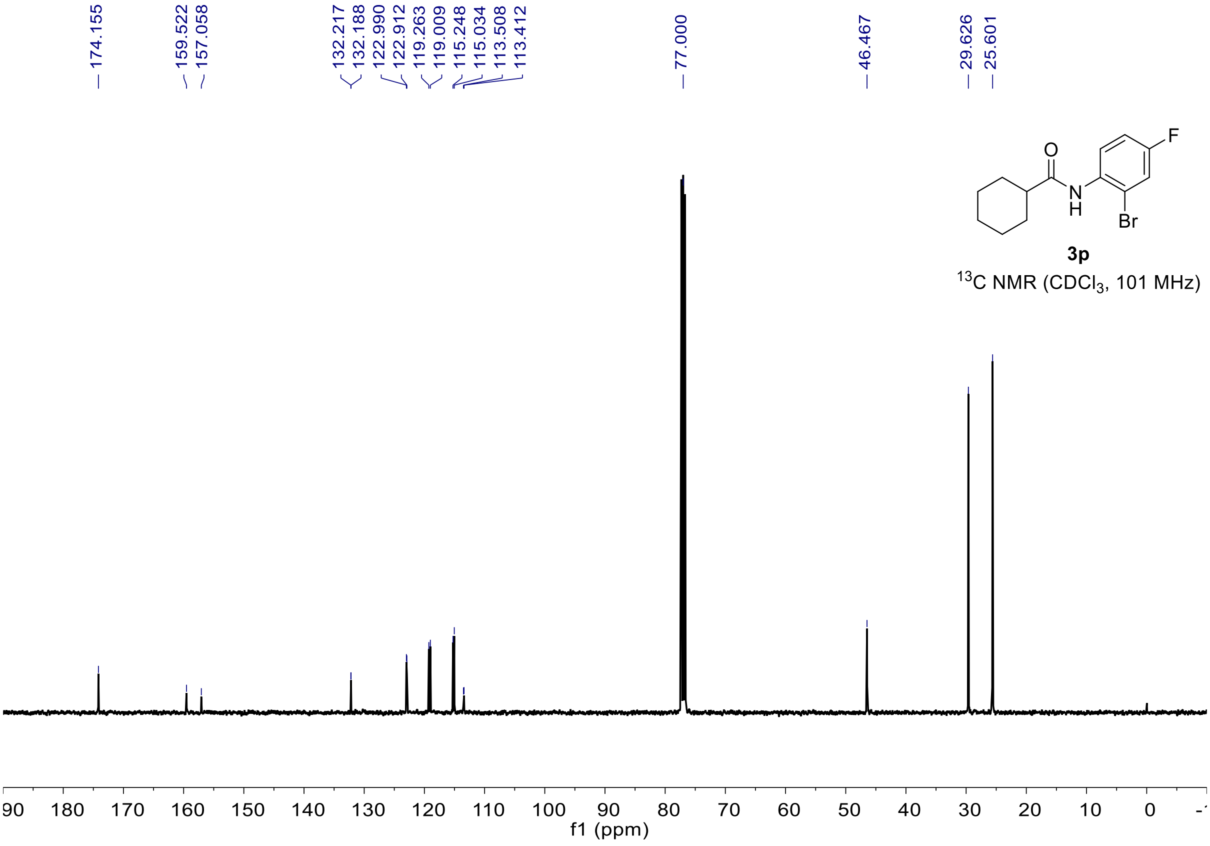
174.155
159.522
157.058
132.217
132.188
122.990
122.912
119.263
119.009
115.248
115.034
113.508
113.412
77.000
46.467
29.626
25.601
O
N
H
F
Br
3p
13C NMR (CDCl3, 101 MHz)
f1 (ppm)

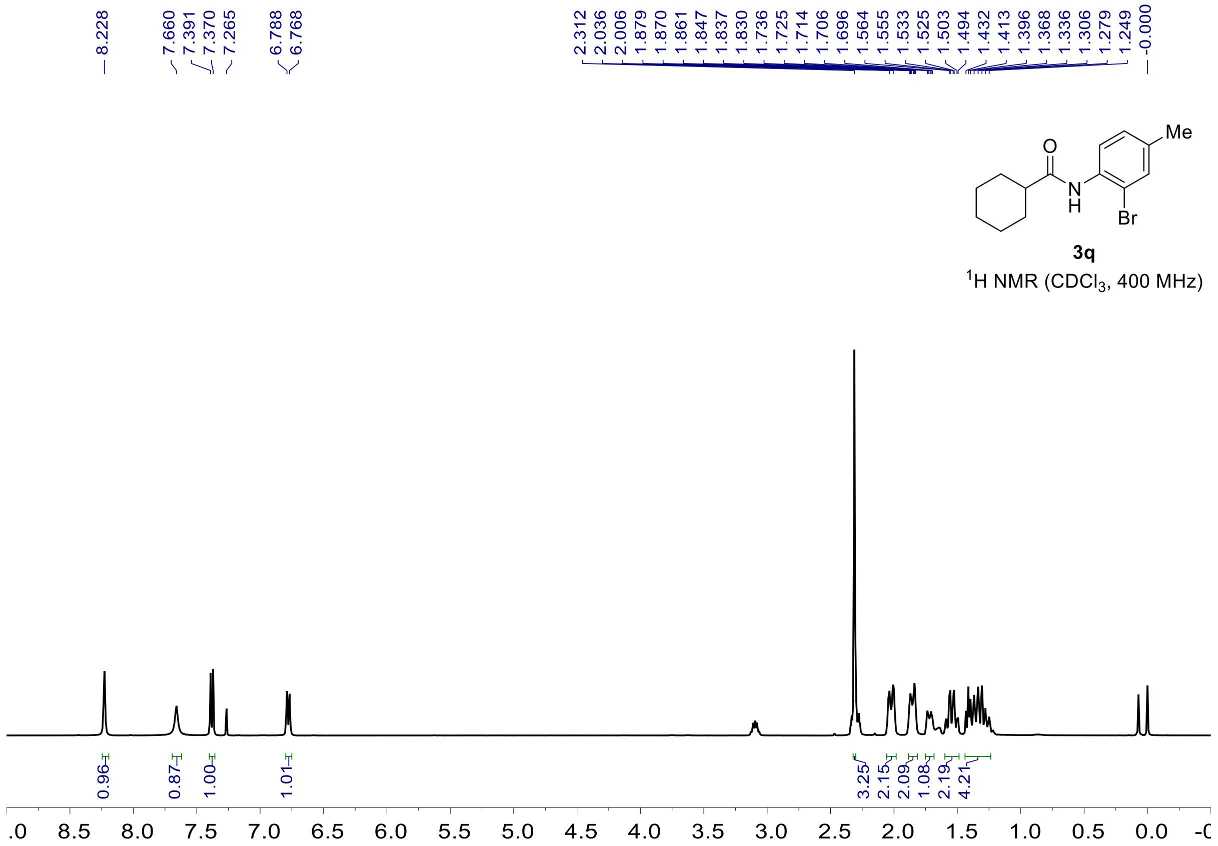
8.228
7.660
7.391
7.370
7.265
6.788
6.768
2.312
2.036
2.006
1.879
1.870
1.861
1.847
1.837
1.830
1.736
1.725
1.714
1.706
1.696
1.564
1.555
1.533
1.525
1.503
1.494
1.432
1.413
1.396
1.368
1.336
1.306
1.279
1.249
-0.000
O
N
H
Me
Br
3q
^{1}H NMR ($CDCl_3$, 400 MHz)
0.96
0.87
1.00
1.01
3.25
2.15
2.09
1.08
2.19
4.21
.0 8.5 8.0 7.5 7.0 6.5 6.0 5.5 5.0 4.5 4.0 3.5 3.0 2.5 2.0 1.5 1.0 0.5 0.0

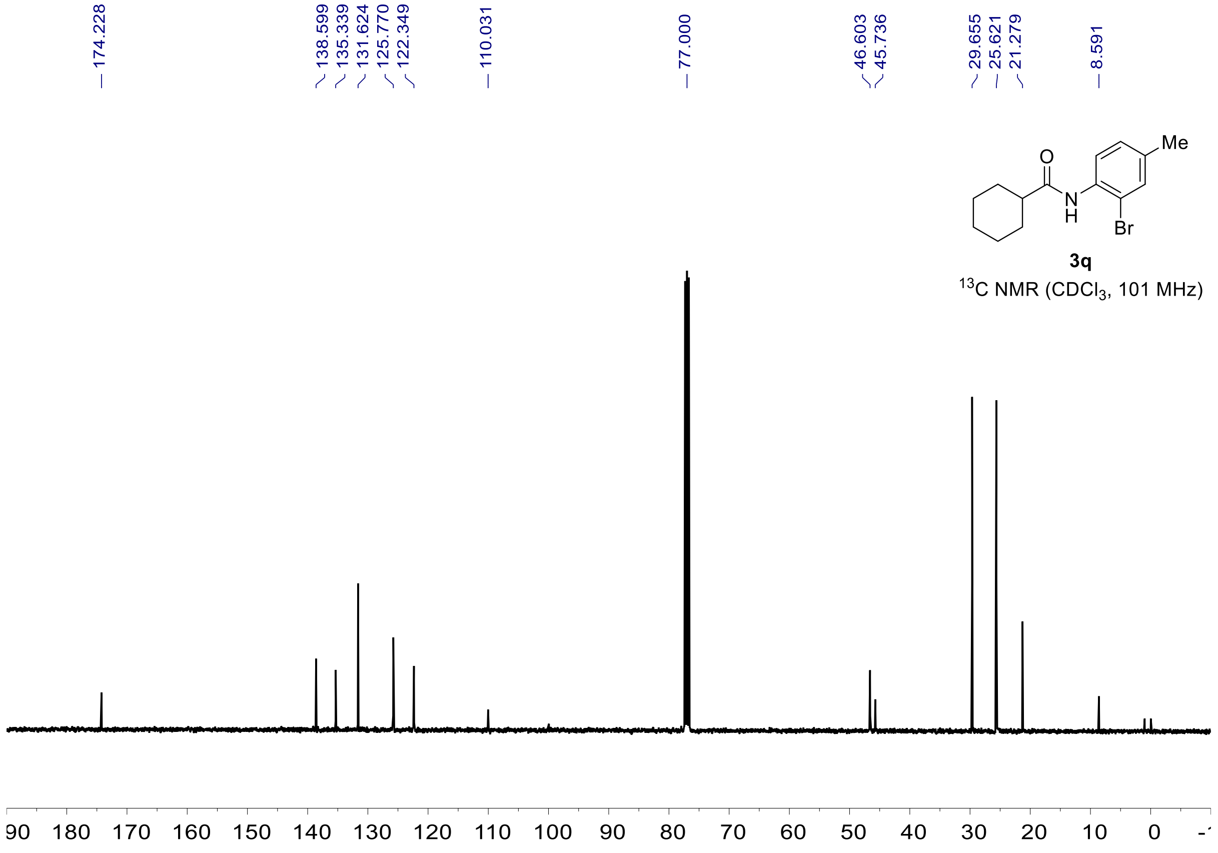
174.228
138.599
135.339
131.624
125.770
122.349
110.031
77.000
46.603
45.736
29.655
25.621
21.279
8.591
O
N
H
Me
Br
3q
^{13}C NMR ($CDCl_3$, 101 MHz)
90 180 170 160 150 140 130 120 110 100 90 80 70 60 50 40 30 20 10 0

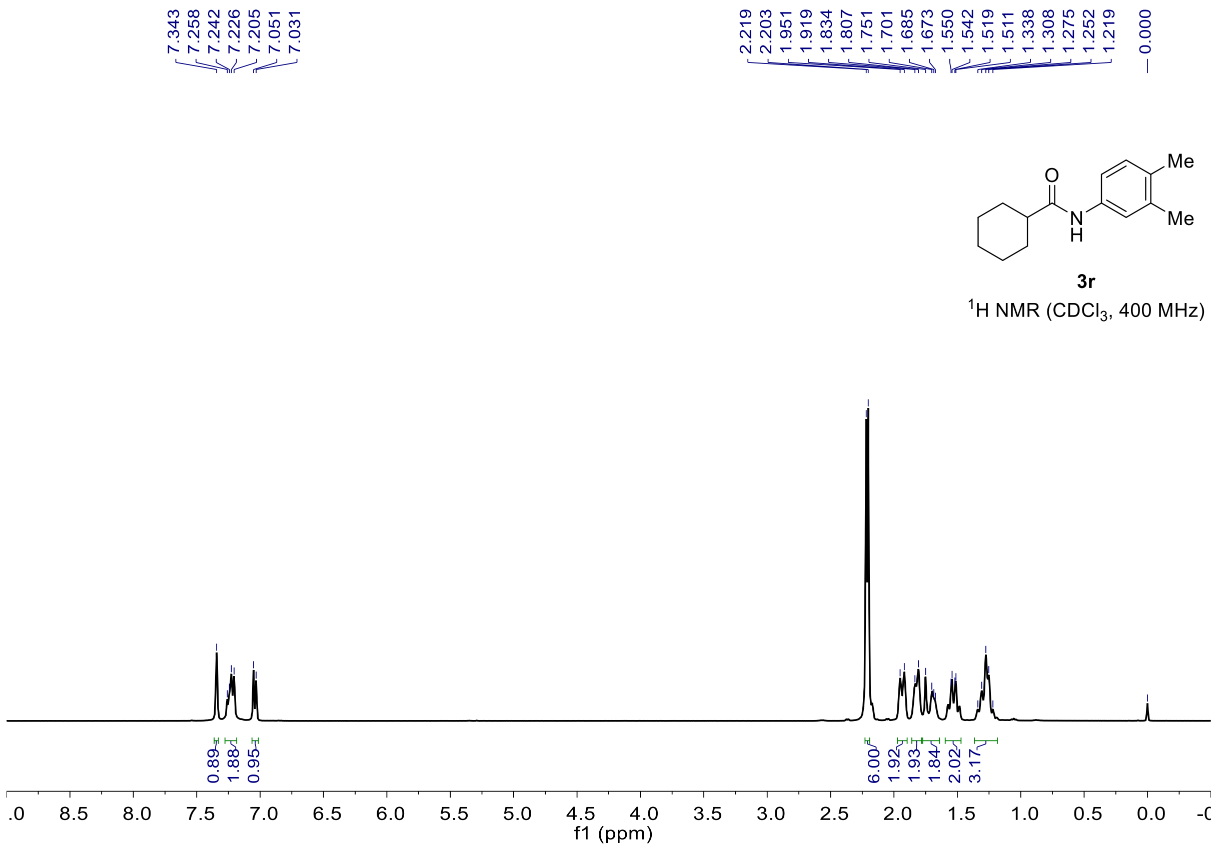
7.343
7.258
7.242
7.226
7.205
7.051
7.031
2.219
2.203
1.951
1.919
1.834
1.807
1.751
1.701
1.685
1.673
1.550
1.542
1.519
1.511
1.338
1.308
1.275
1.252
1.219
0.000
O
N
H
Me
Me
3r
^{1}H NMR ($CDCl_3$, 400 MHz)
0.89
1.88
0.95
6.00
1.92
1.93
1.84
2.02
3.17
f1 (ppm)

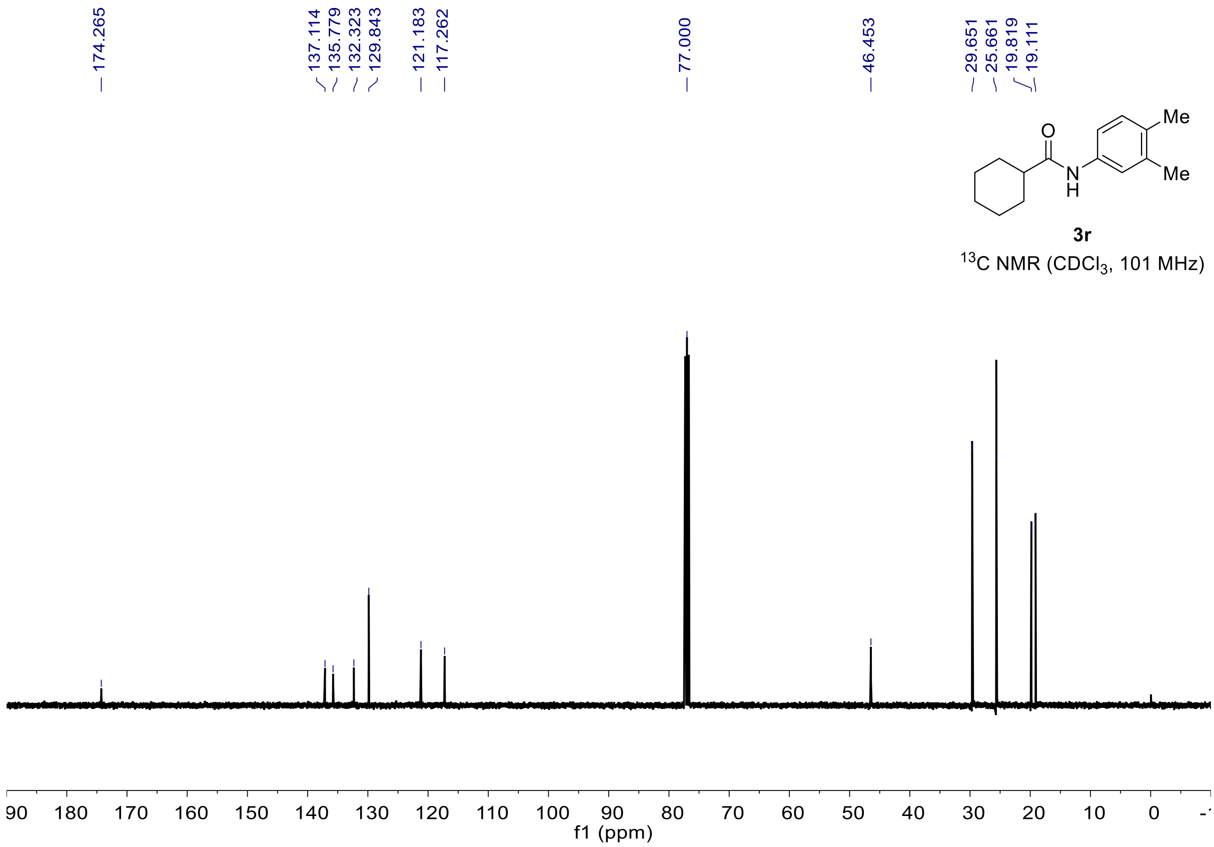
174.265
137.114
135.779
132.323
129.843
121.183
117.262
77.000
46.453
29.651
25.661
19.819
19.111
O
N
H
Me
Me
3r
^{13}C NMR ($CDCl_3$, 101 MHz)
f1 (ppm)

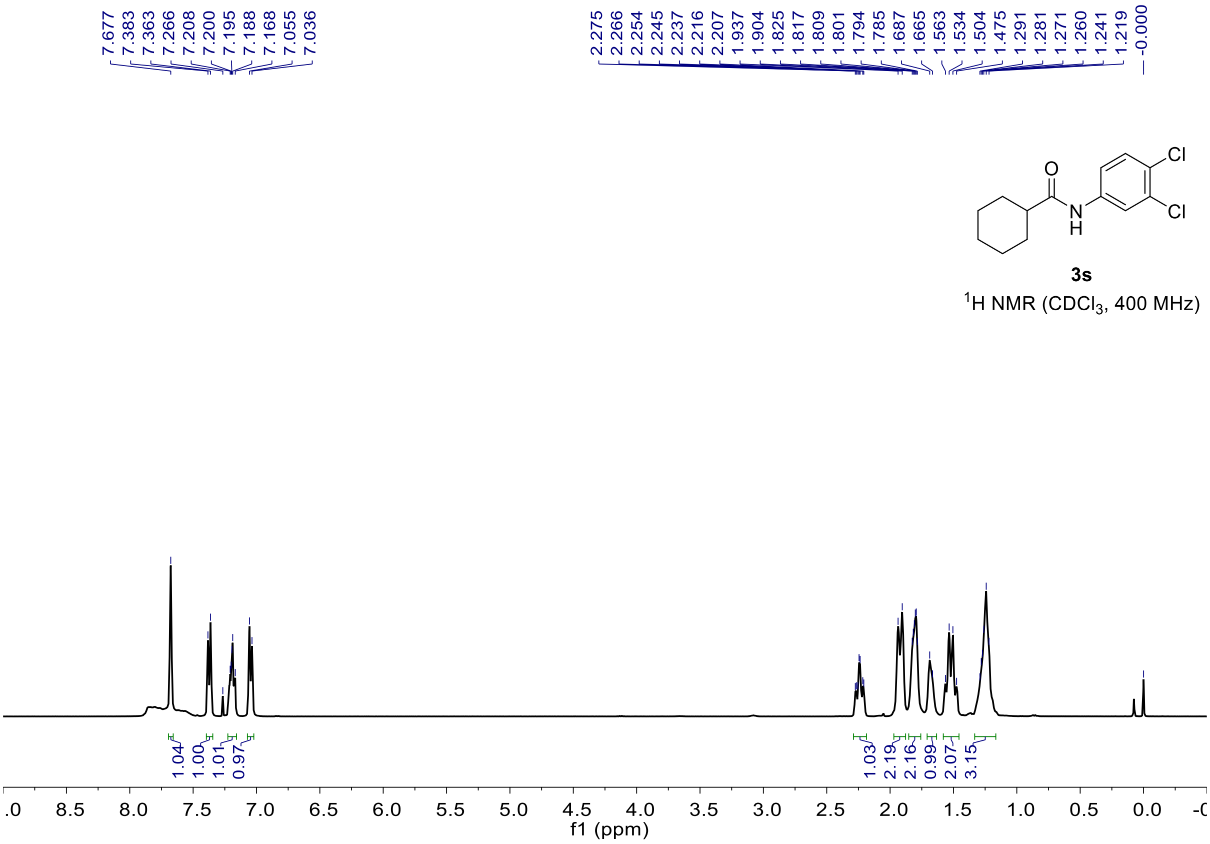
7.677
7.383
7.363
7.266
7.208
7.200
7.195
7.188
7.168
7.055
7.036
2.275
2.266
2.254
2.245
2.237
2.216
2.207
1.937
1.904
1.825
1.817
1.809
1.801
1.794
1.785
1.687
1.665
1.563
1.534
1.504
1.475
1.291
1.281
1.271
1.260
1.241
1.219
-0.000
O
N
H
Cl
Cl
3s
^{1}H NMR ($CDCl_3$, 400 MHz)
1.04
1.00
1.01
0.97
1.03
2.19
2.16
0.99
2.07
3.15
f1 (ppm)

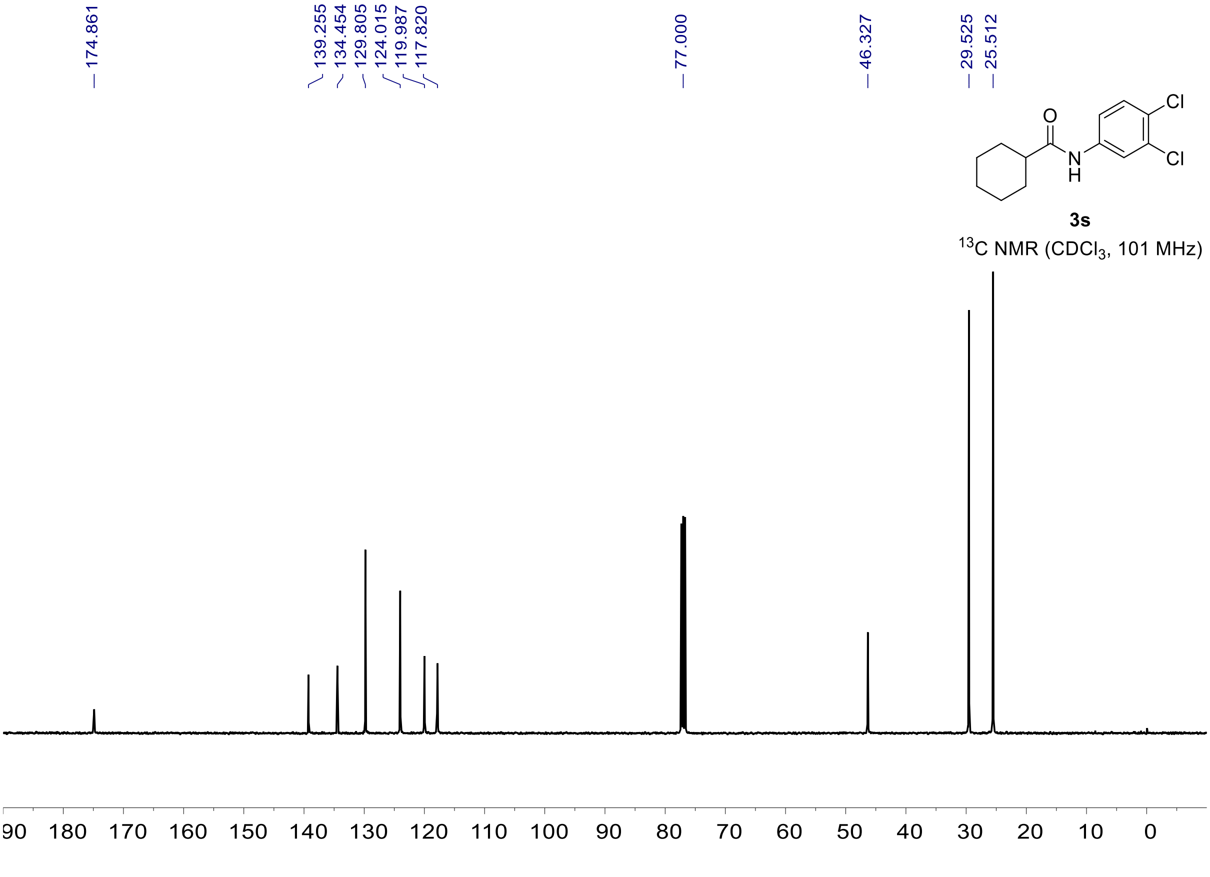
174.861
139.255
134.454
129.805
124.015
119.987
117.820
77.000
46.327
29.525
25.512
O
N
H
Cl
Cl
3s
^{13}C NMR ($CDCl_3$, 101 MHz)

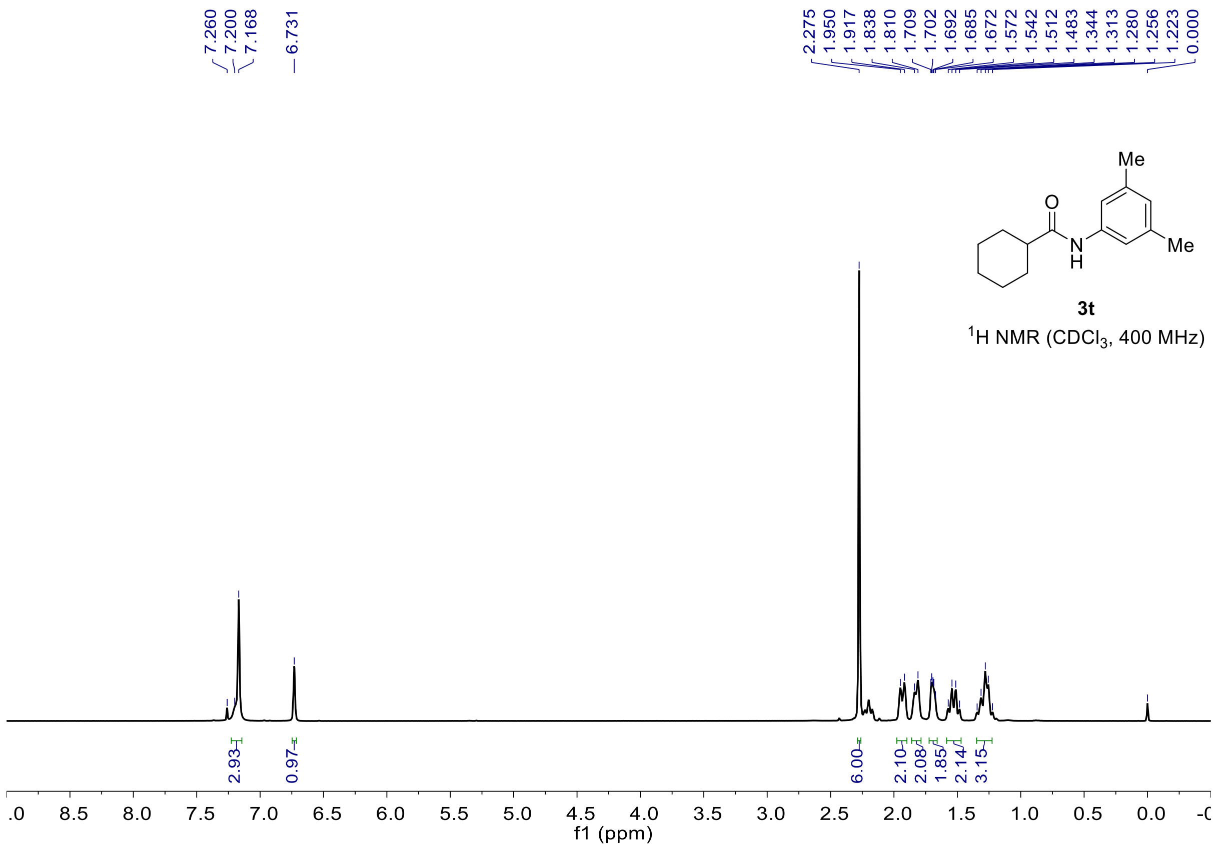
7.260
7.200
7.168
6.731
2.275
1.950
1.917
1.838
1.810
1.709
1.702
1.692
1.685
1.672
1.572
1.542
1.512
1.483
1.344
1.313
1.280
1.256
1.223
0.000
Me
O
N
H
Me
3t
^{1}H NMR ($CDCl_3$, 400 MHz)
2.93
0.97
6.00
2.10
2.08
1.85
2.14
3.15
f1 (ppm)

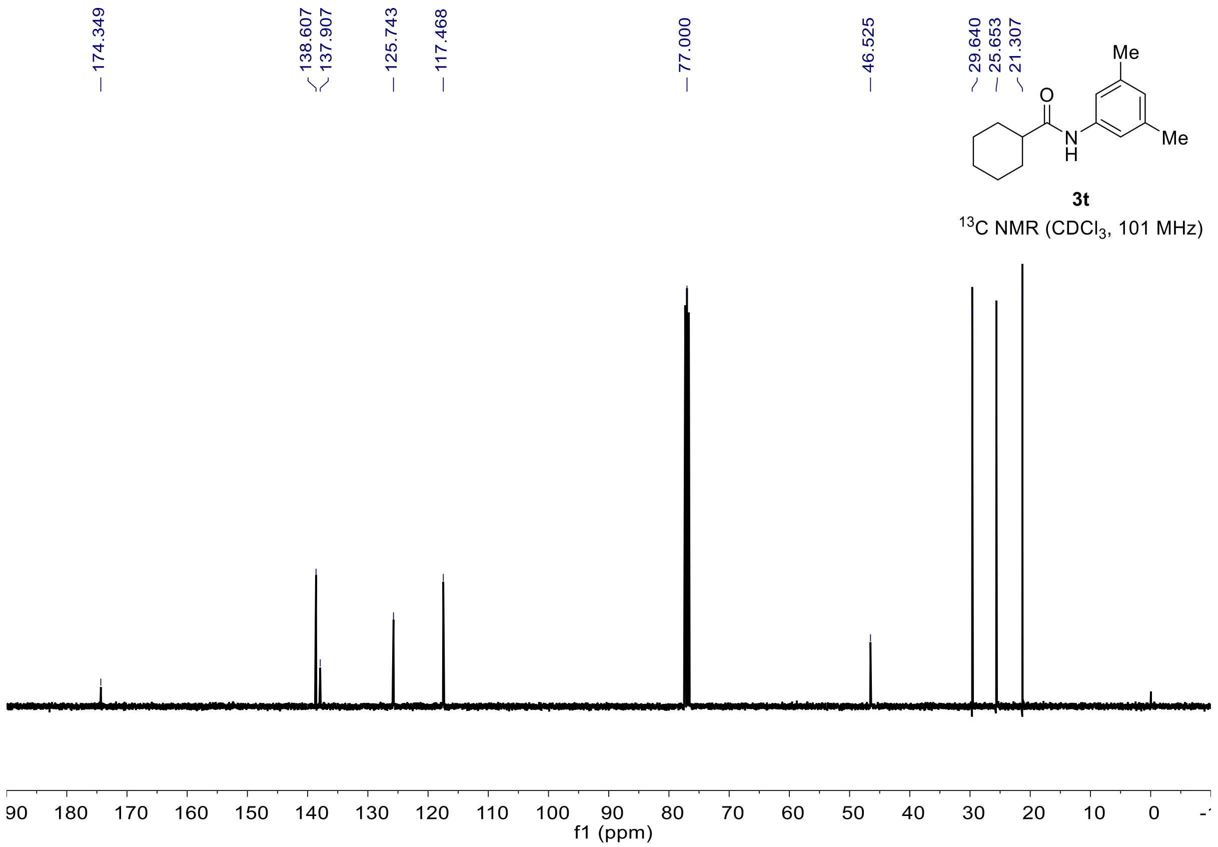
174.349
138.607
137.907
125.743
117.468
77.000
46.525
29.640
25.653
21.307
Me
O
N
H
Me
3t
^{13}C NMR ($CDCl_3$, 101 MHz)
f1 (ppm)

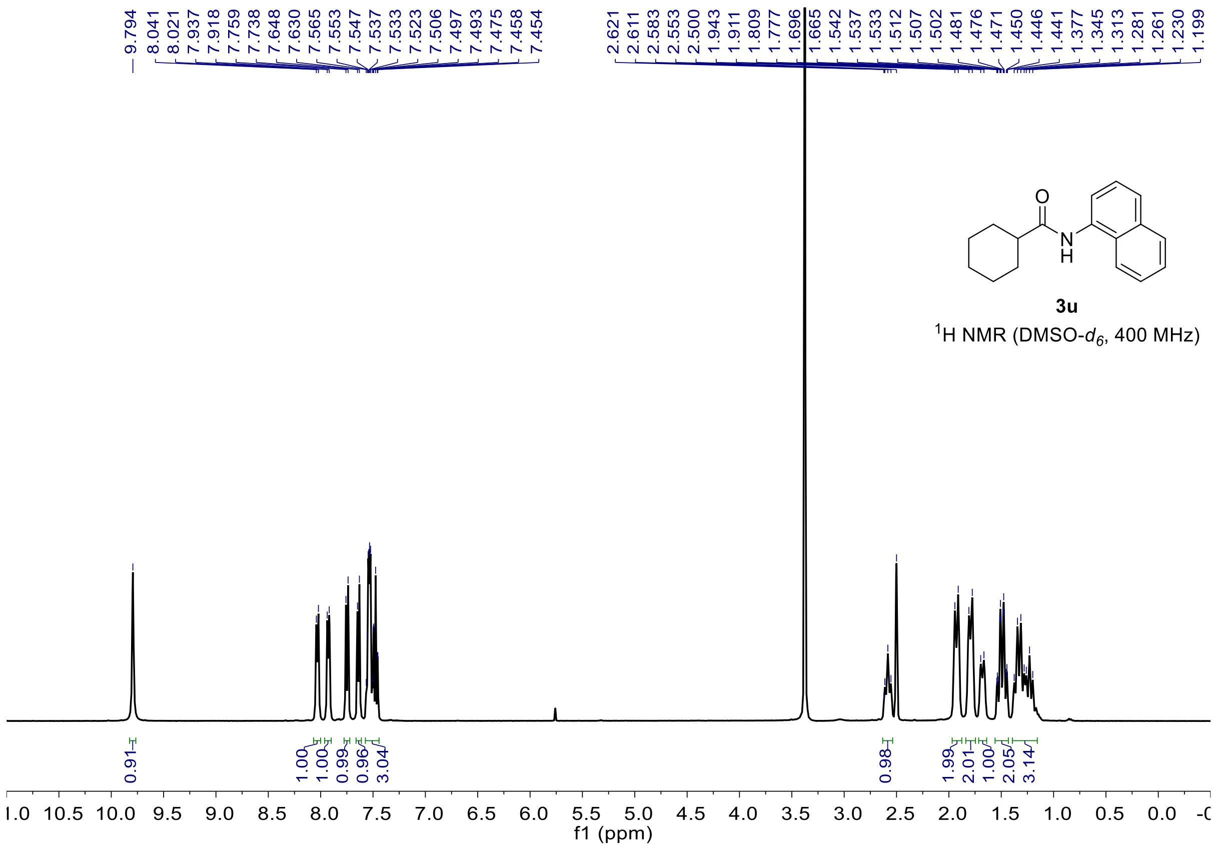
9.794
8.041 8.021 7.937 7.918 7.759 7.738 7.648 7.630 7.565 7.553 7.547 7.537 7.533 7.523 7.506 7.497 7.493 7.475 7.458 7.454
2.621 2.611 2.583 2.553 2.500 1.943 1.911 1.809 1.777 1.696 1.665 1.542 1.537 1.533 1.512 1.507 1.502 1.481 1.476 1.471 1.450 1.446 1.441 1.377 1.345 1.313 1.281 1.261 1.230 1.199
O
N
H
3u
1H NMR (DMSO-d6, 400 MHz)
0.91
1.00 1.00 0.99 0.96 3.04
0.98
1.99 2.01 1.00 2.05 3.14
f1 (ppm)

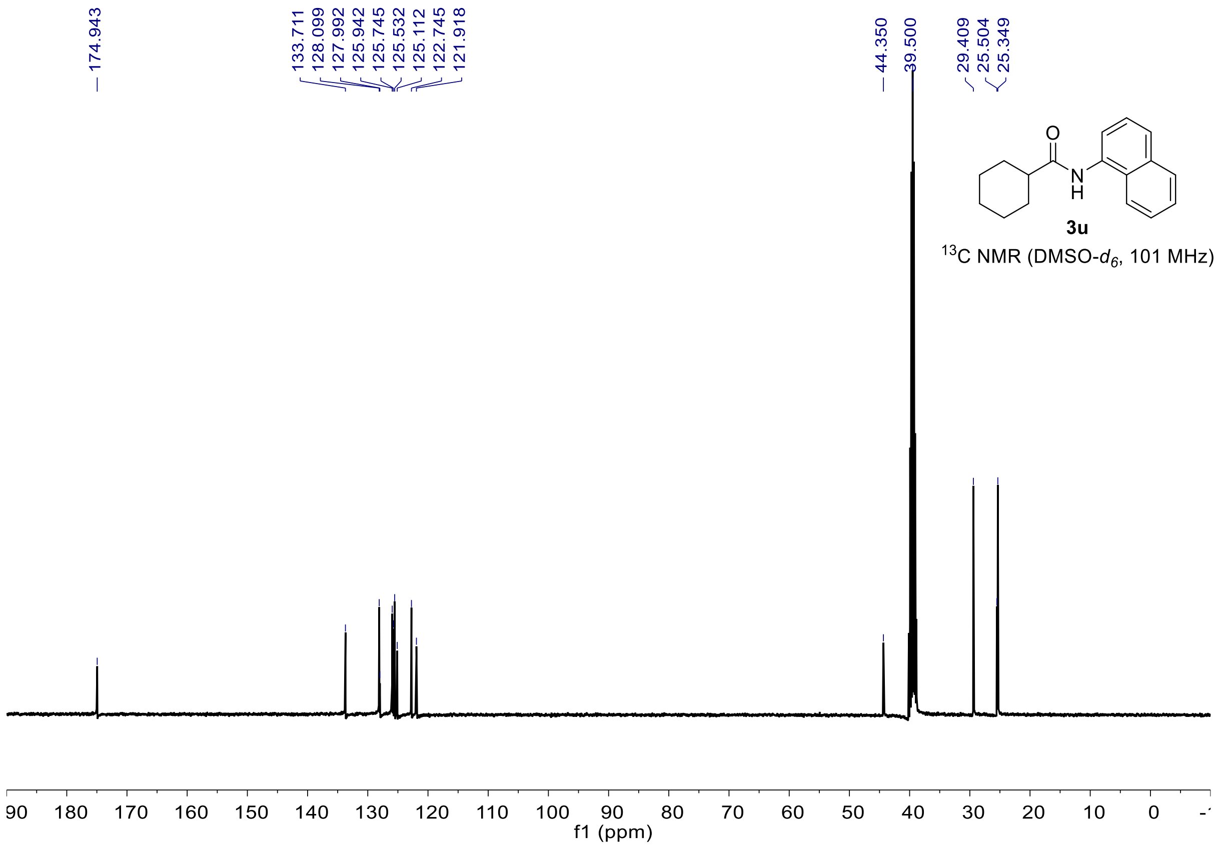
174.943
133.711 128.099 127.992 125.942 125.745 125.532 125.112 122.745 121.918
44.350 39.500
29.409 25.504 25.349
O
N
H
3u
13C NMR (DMSO-d6, 101 MHz)
f1 (ppm)

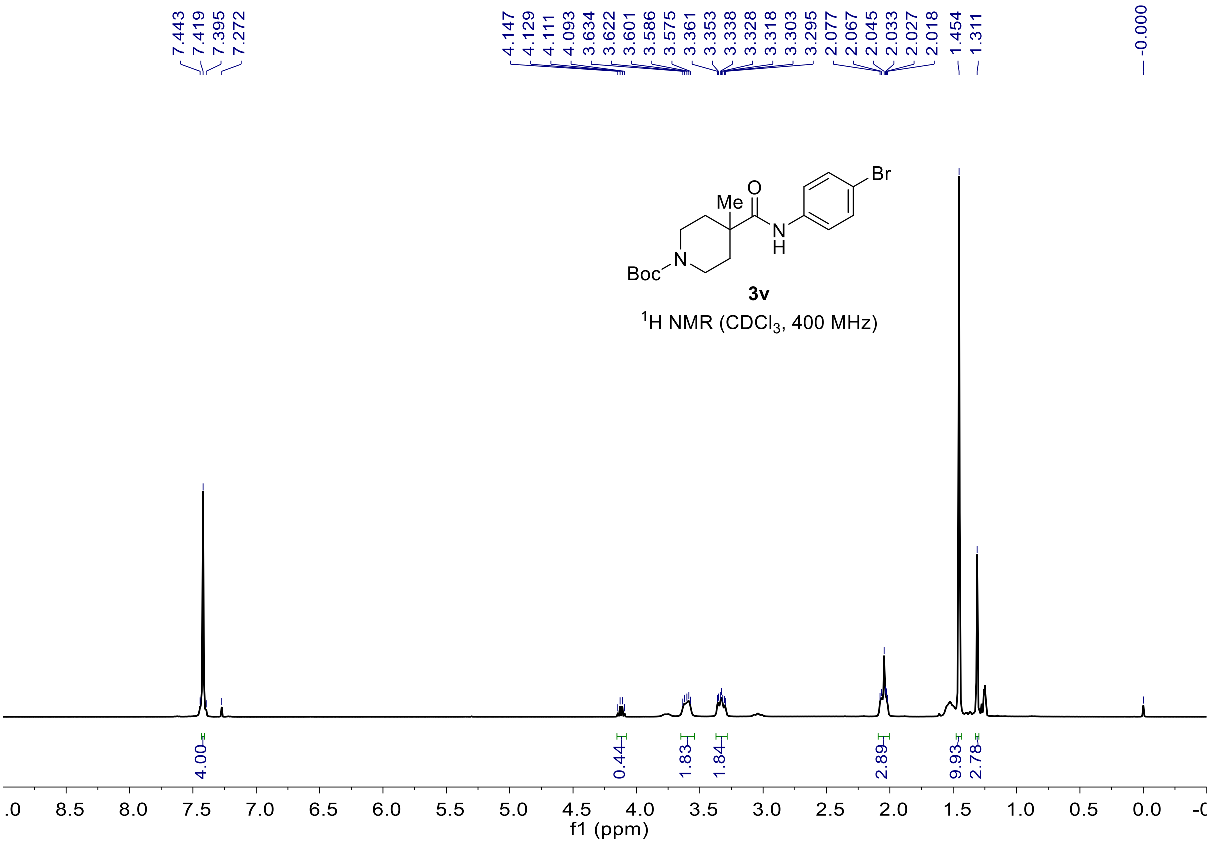

3v

^{1}H NMR ($CDCl_3$, 400 MHz)

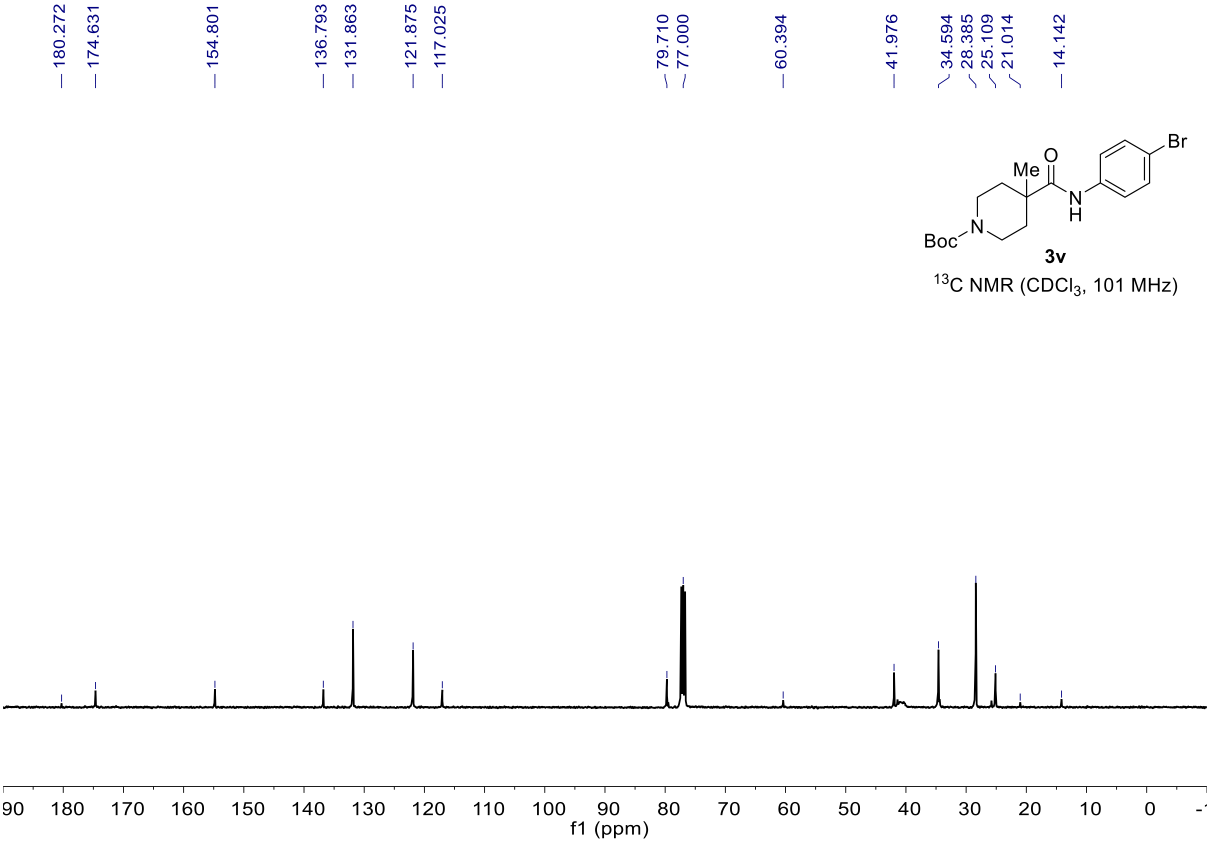

3v

^{13}C NMR ($CDCl_3$, 101 MHz)

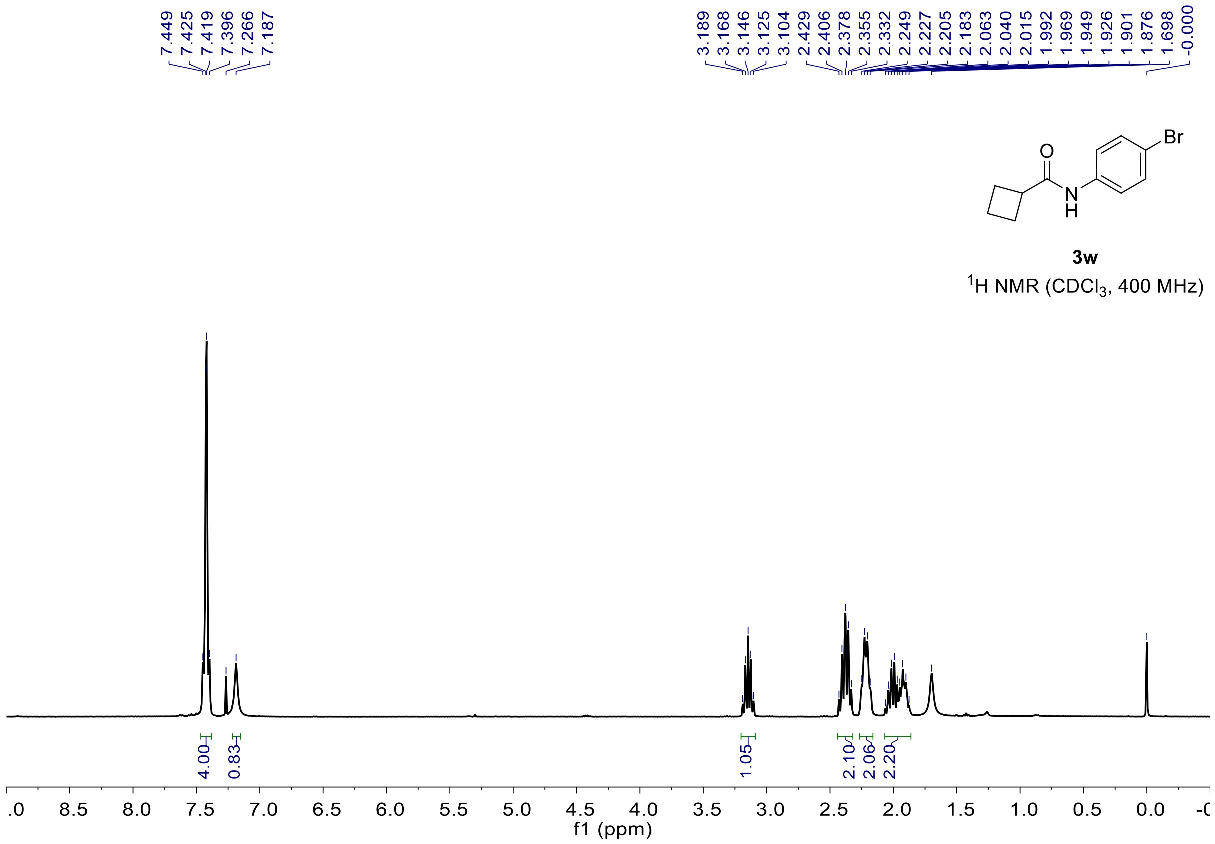

3w

^{1}H NMR ($CDCl_3$, 400 MHz)

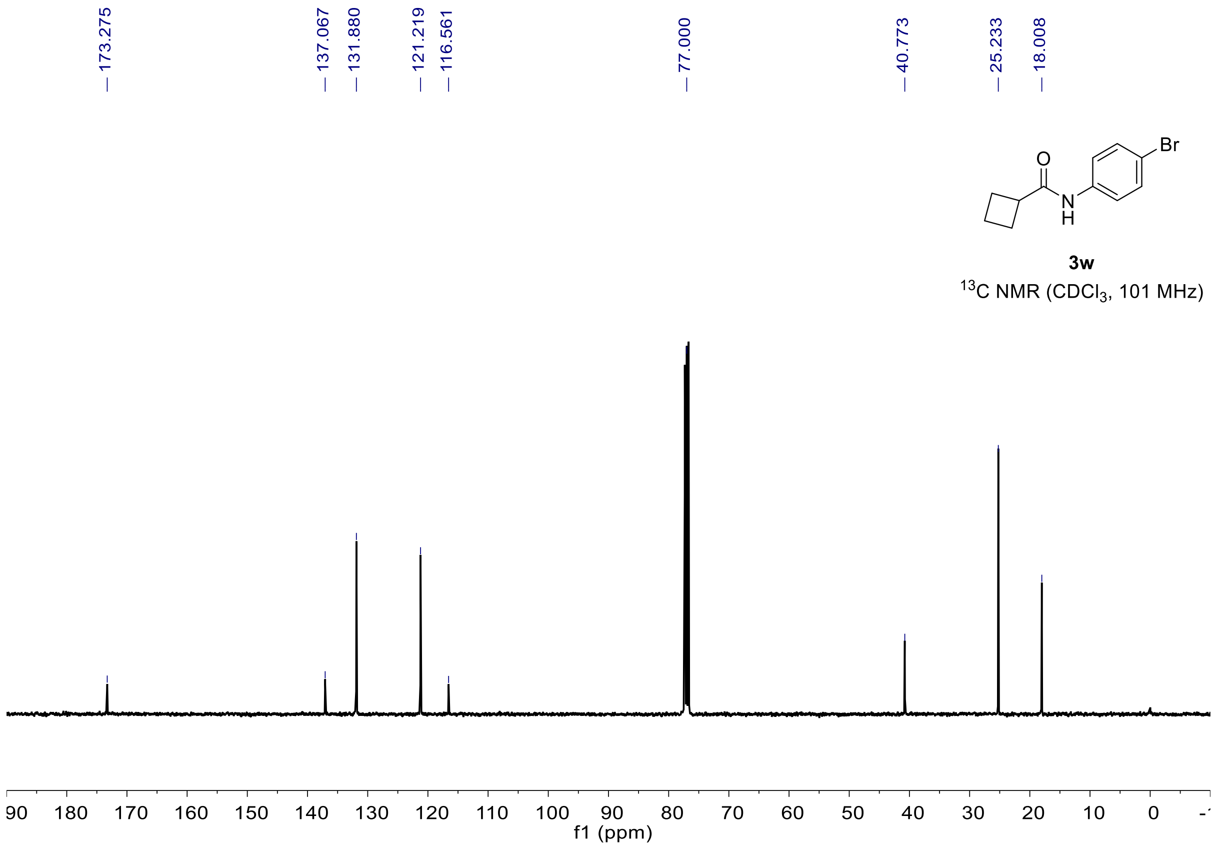

3w

^{13}C NMR ($CDCl_3$, 101 MHz)

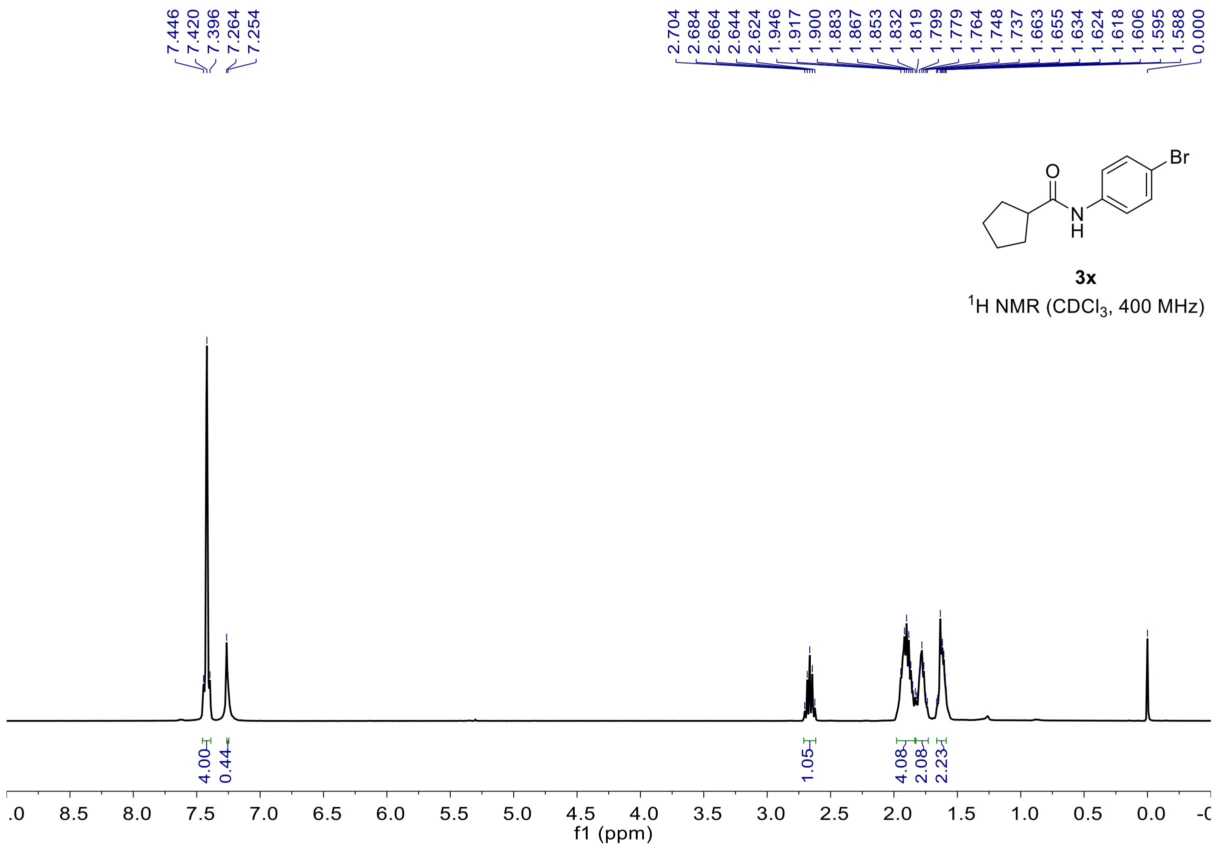
7.446
7.420
7.396
7.264
7.254
2.704
2.684
2.664
2.644
2.624
1.946
1.917
1.900
1.883
1.867
1.853
1.832
1.819
1.799
1.779
1.764
1.748
1.737
1.663
1.655
1.634
1.624
1.618
1.606
1.595
1.588
0.000
Br
O
N
H
3x
1H NMR (CDCl3, 400 MHz)
4.00
0.44
1.05
4.08
2.08
2.23
f1 (ppm)

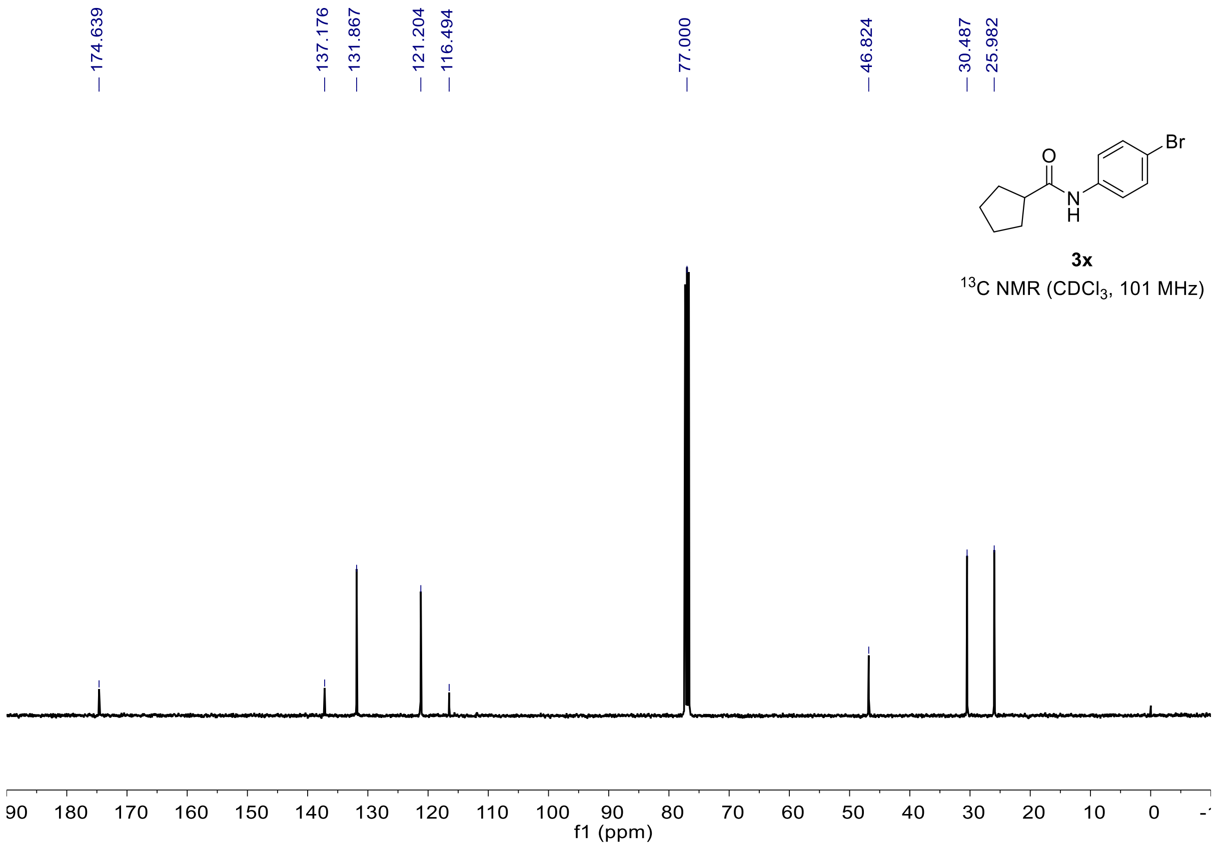
174.639
137.176
131.867
121.204
116.494
77.000
46.824
30.487
25.982
Br
O
N
H
3x
13C NMR (CDCl3, 101 MHz)
f1 (ppm)

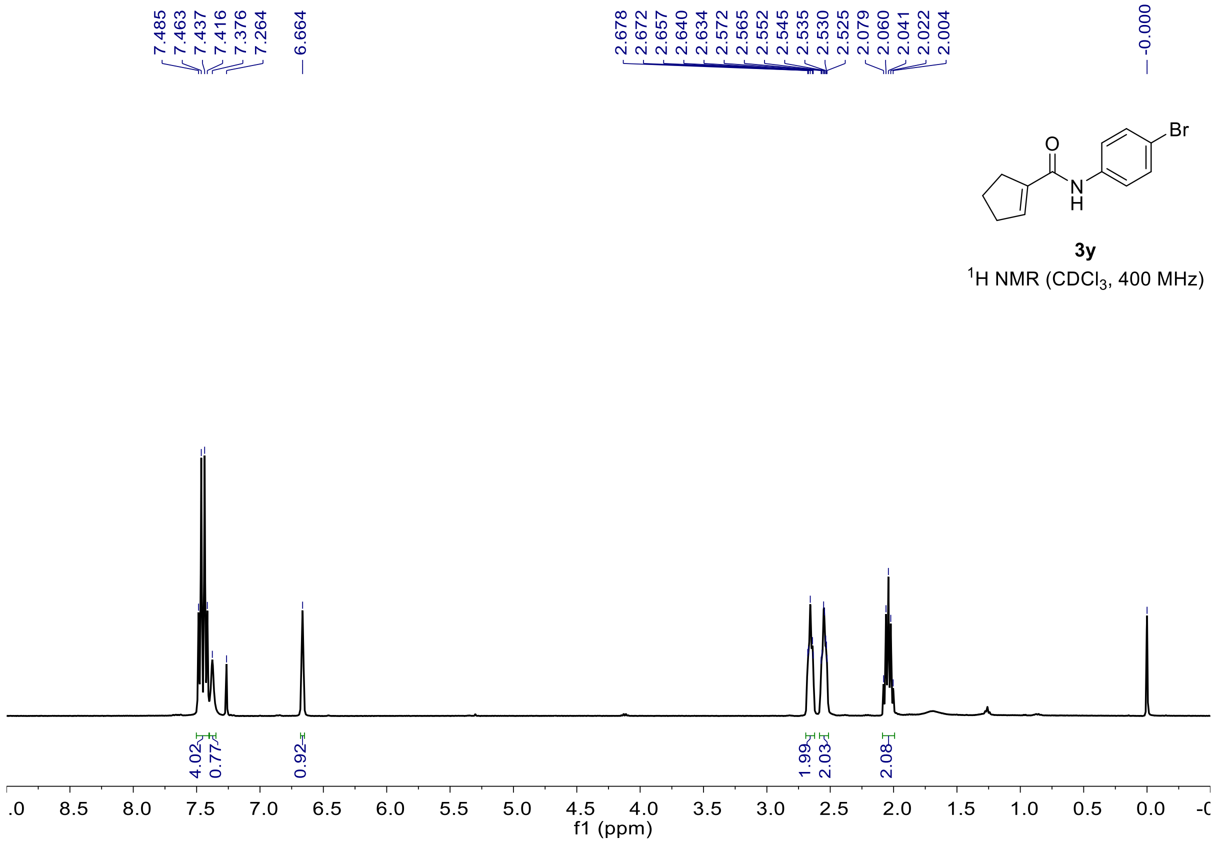
7.485
7.463
7.437
7.416
7.376
7.264
6.664
2.678
2.672
2.657
2.640
2.634
2.572
2.565
2.552
2.545
2.535
2.530
2.525
2.079
2.060
2.041
2.022
2.004
-0.000
O
N
H
Br
3y
^{1}H NMR ($CDCl_3$, 400 MHz)
4.02
0.77
0.92
1.99
2.03
2.08
8.5 8.0 7.5 7.0 6.5 6.0 5.5 5.0 4.5 4.0 3.5 3.0 2.5 2.0 1.5 1.0 0.5 0.0
f1 (ppm)

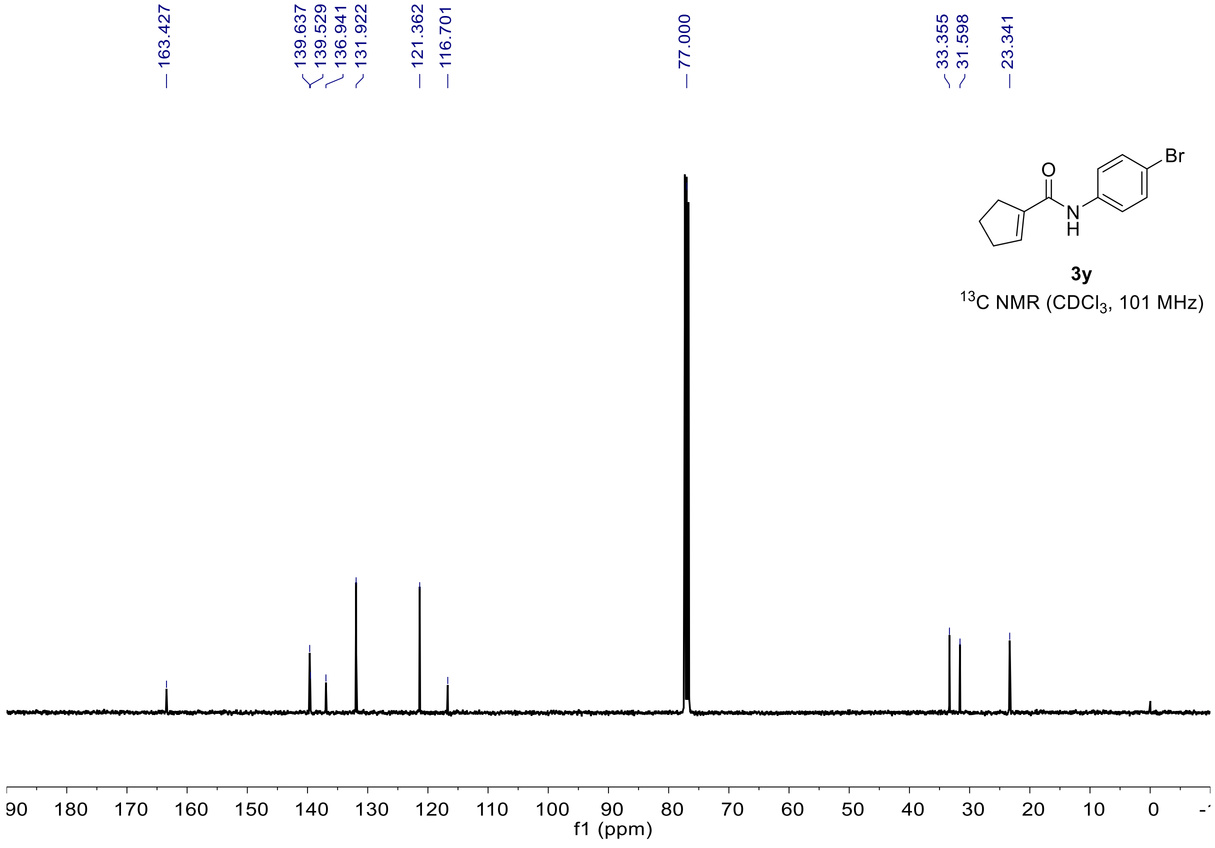
163.427
139.637
139.529
136.941
131.922
121.362
116.701
77.000
33.355
31.598
23.341
O
N
H
Br
3y
^{13}C NMR ($CDCl_3$, 101 MHz)
180 170 160 150 140 130 120 110 100 90 80 70 60 50 40 30 20 10 0
f1 (ppm)

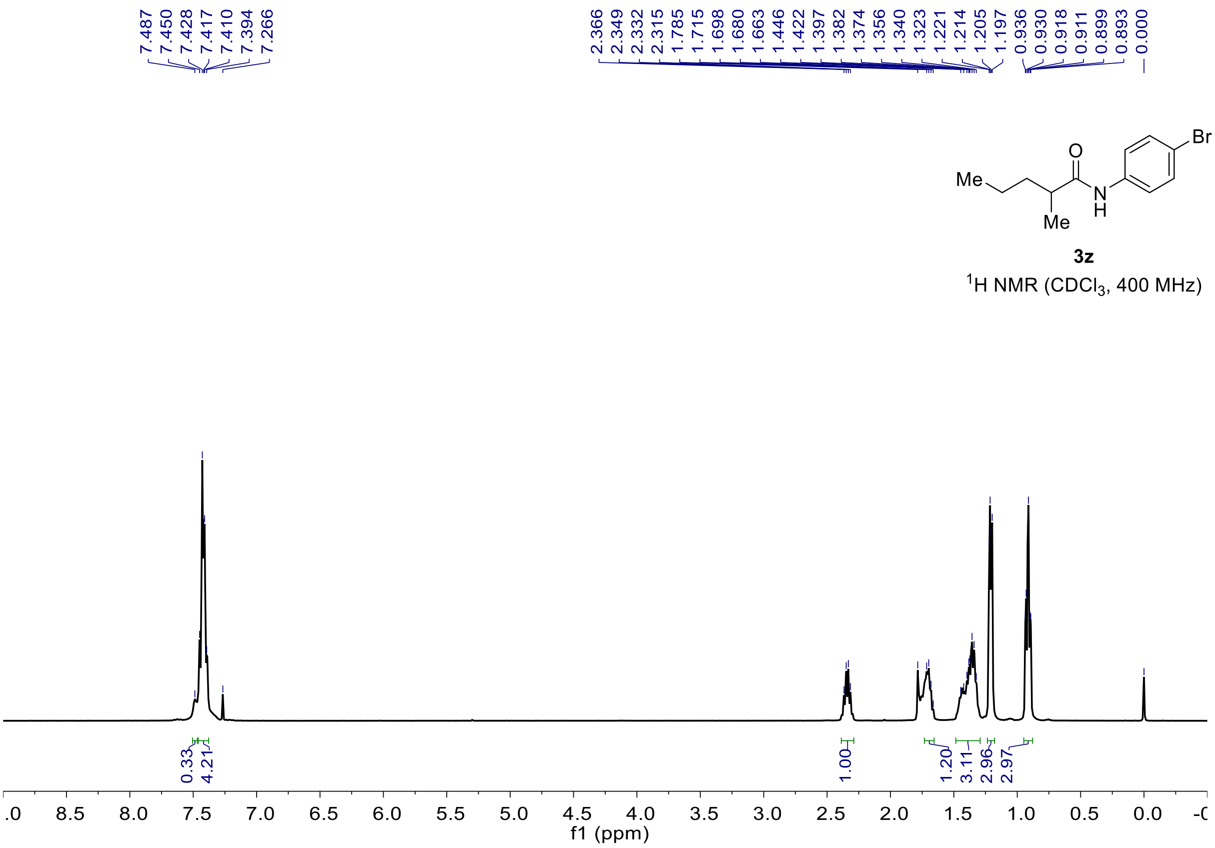

3z

^{1}H NMR ($CDCl_3$, 400 MHz)

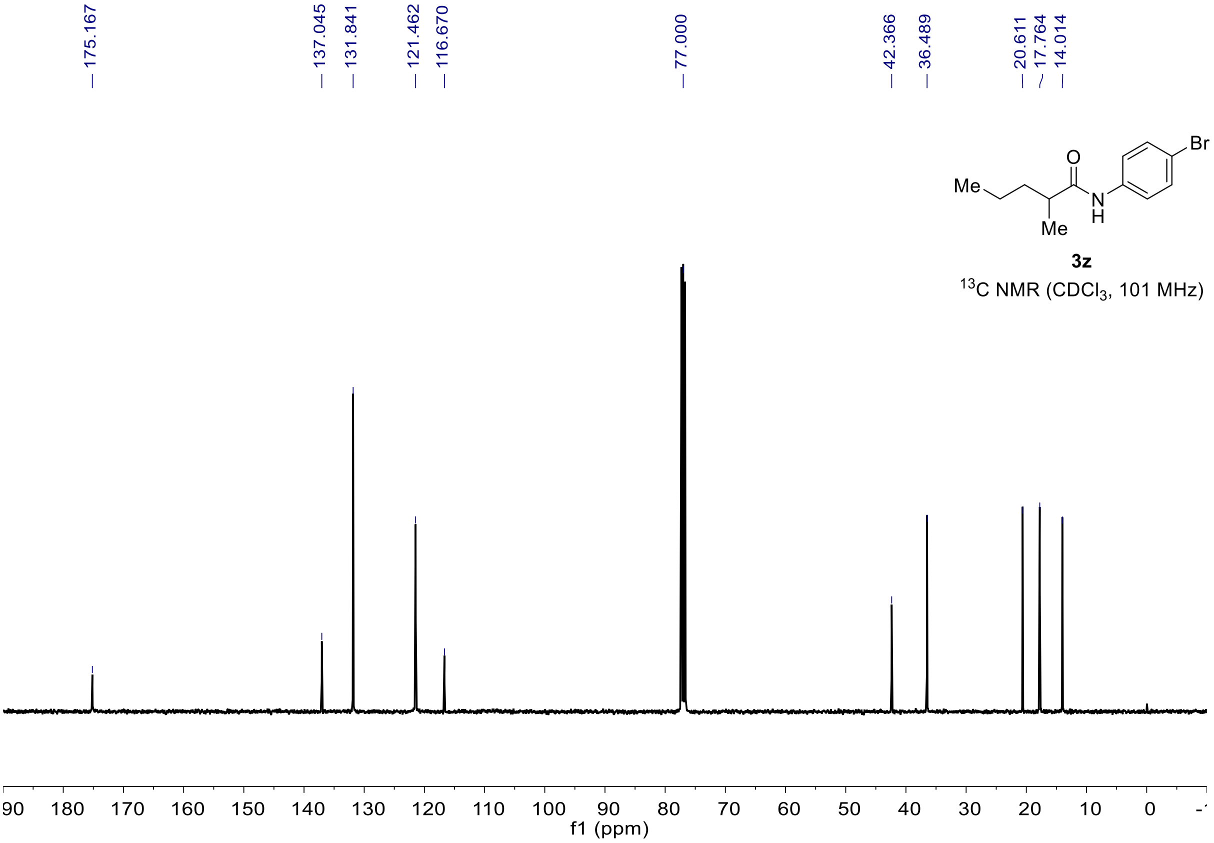

3z

^{13}C NMR ($CDCl_3$, 101 MHz)

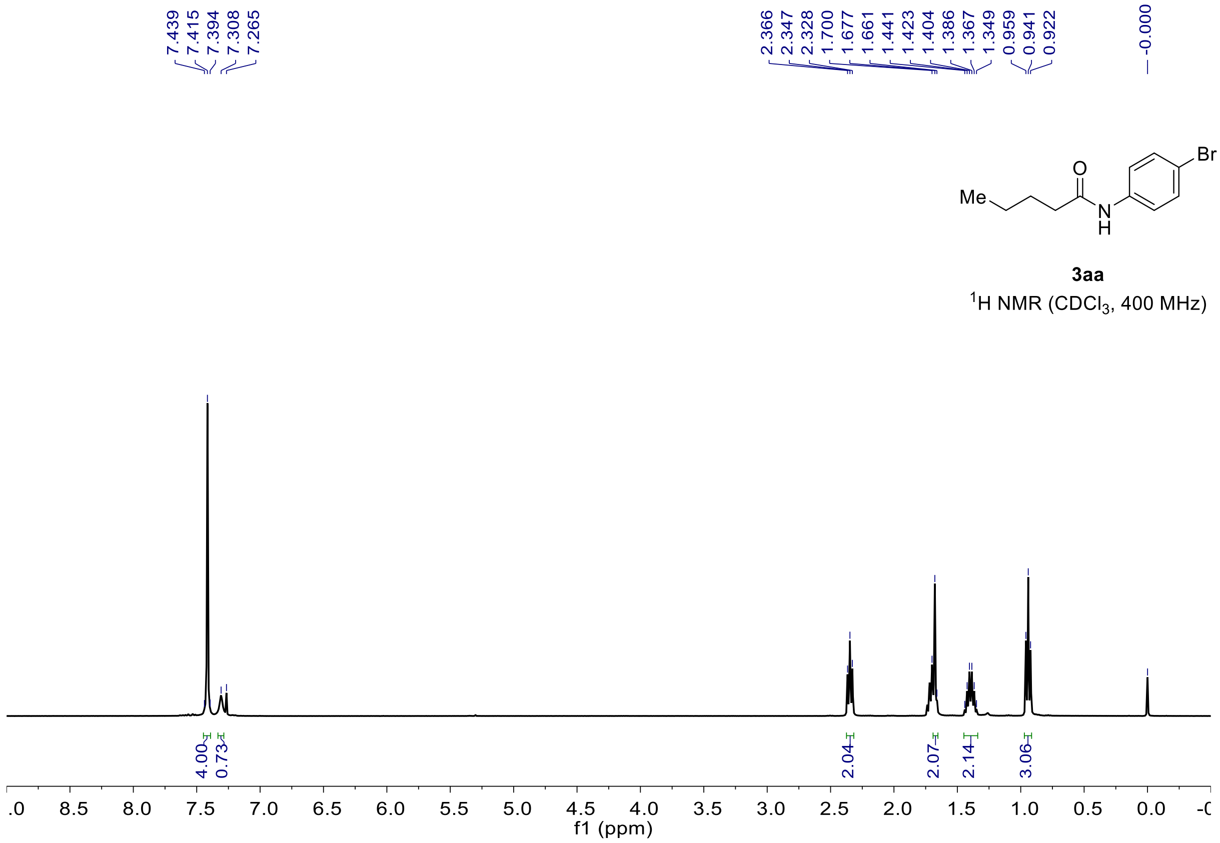
7.439
7.415
7.394
7.308
7.265
2.366
2.347
2.328
1.700
1.677
1.661
1.441
1.423
1.404
1.386
1.367
1.349
0.959
0.941
0.922
-0.000
Me
O
N
H
Br
3aa
1H NMR (CDCl3, 400 MHz)
4.00
0.73
2.04
2.07
2.14
3.06
f1 (ppm)

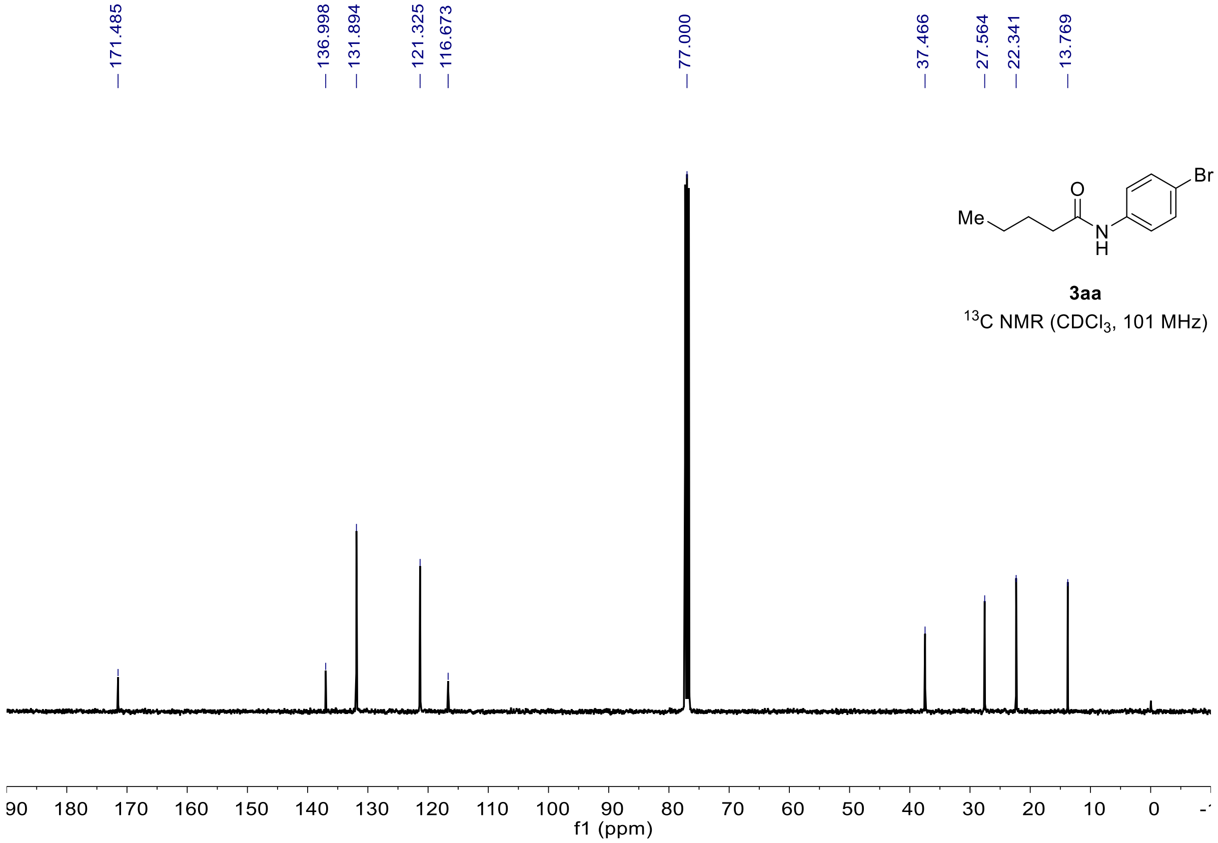
171.485
136.998
131.894
121.325
116.673
77.000
37.466
27.564
22.341
13.769
Me
O
N
H
Br
3aa
13C NMR (CDCl3, 101 MHz)
f1 (ppm)

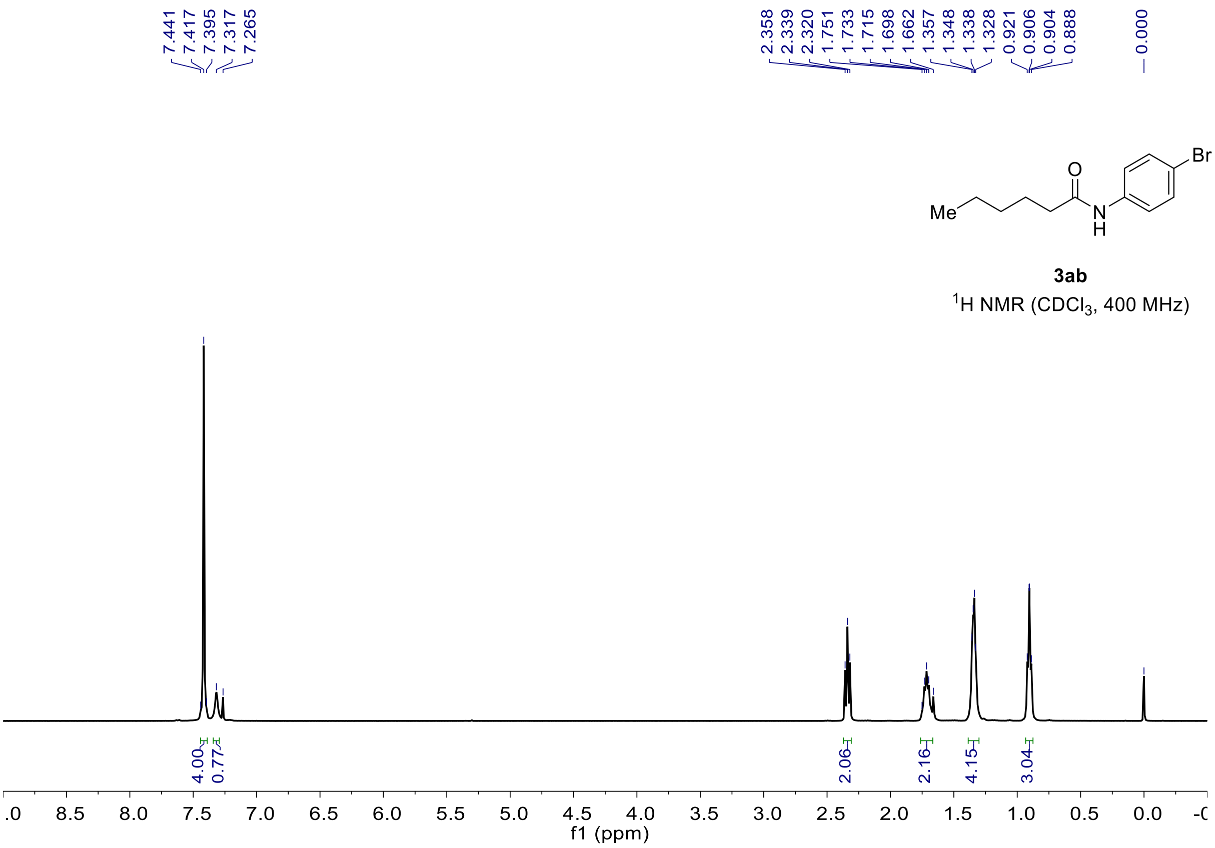
7.441
7.417
7.395
7.317
7.265
2.358
2.339
2.320
1.751
1.733
1.715
1.698
1.662
1.357
1.348
1.338
1.328
0.921
0.906
0.904
0.888
0.000
O
Br
Me
N
H
3ab
1H NMR (CDCl3, 400 MHz)
4.00
0.77
2.06
2.16
4.15
3.04
f1 (ppm)

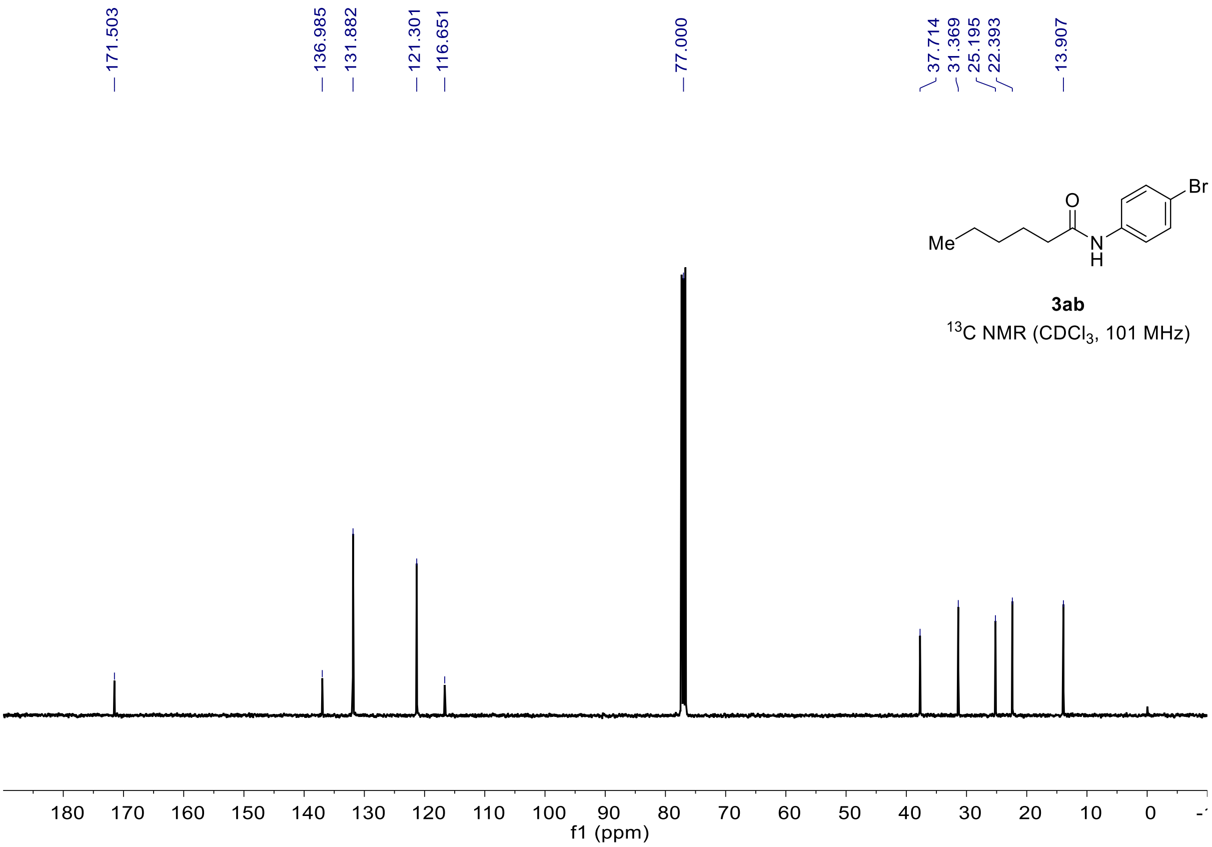
171.503
136.985
131.882
121.301
116.651
77.000
37.714
31.369
25.195
22.393
13.907
O
Br
Me
N
H
3ab
13C NMR (CDCl3, 101 MHz)
f1 (ppm)

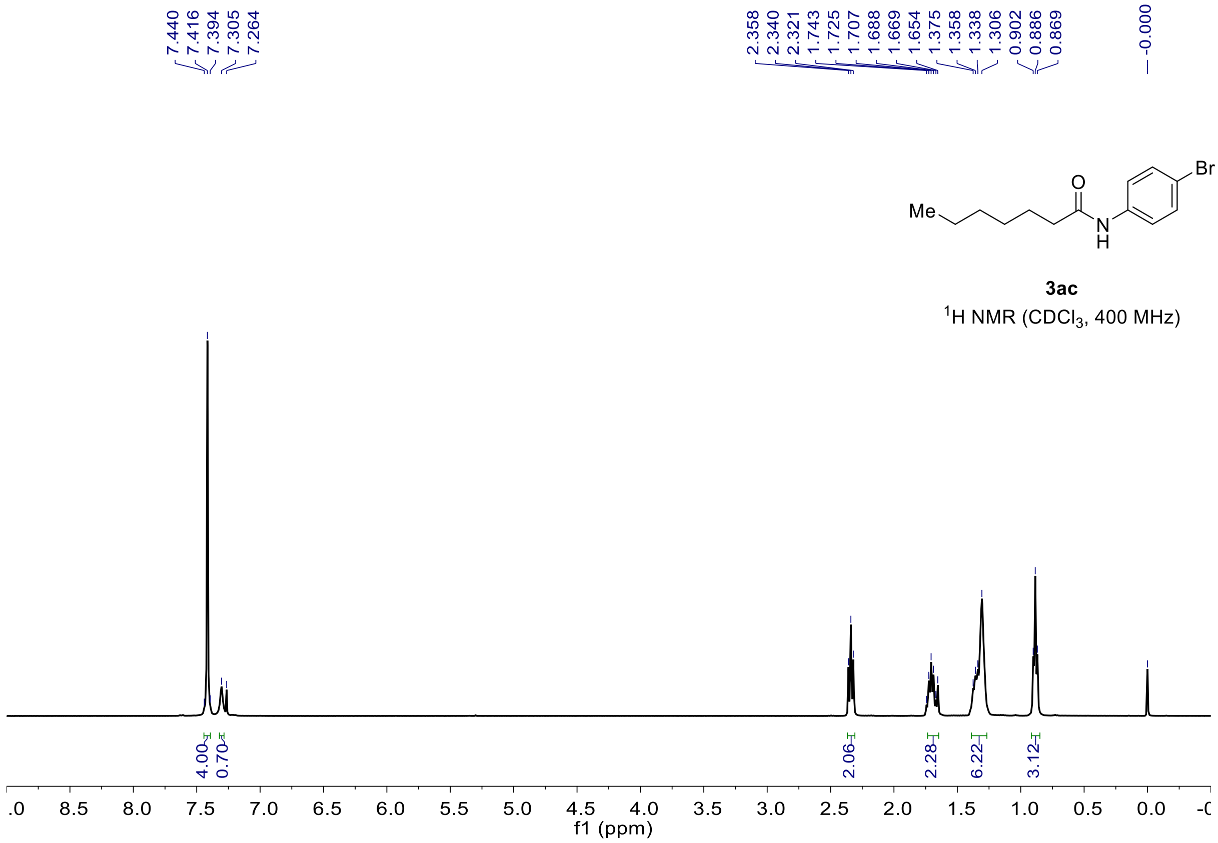
7.440
7.416
7.394
7.305
7.264
2.358
2.340
2.321
1.743
1.725
1.707
1.688
1.669
1.654
1.375
1.358
1.338
1.306
0.902
0.886
0.869
-0.000
Me
O
N
H
Br
3ac
^{1}H NMR ($CDCl_3$, 400 MHz)
4.00
0.70
2.06
2.28
6.22
3.12
f1 (ppm)

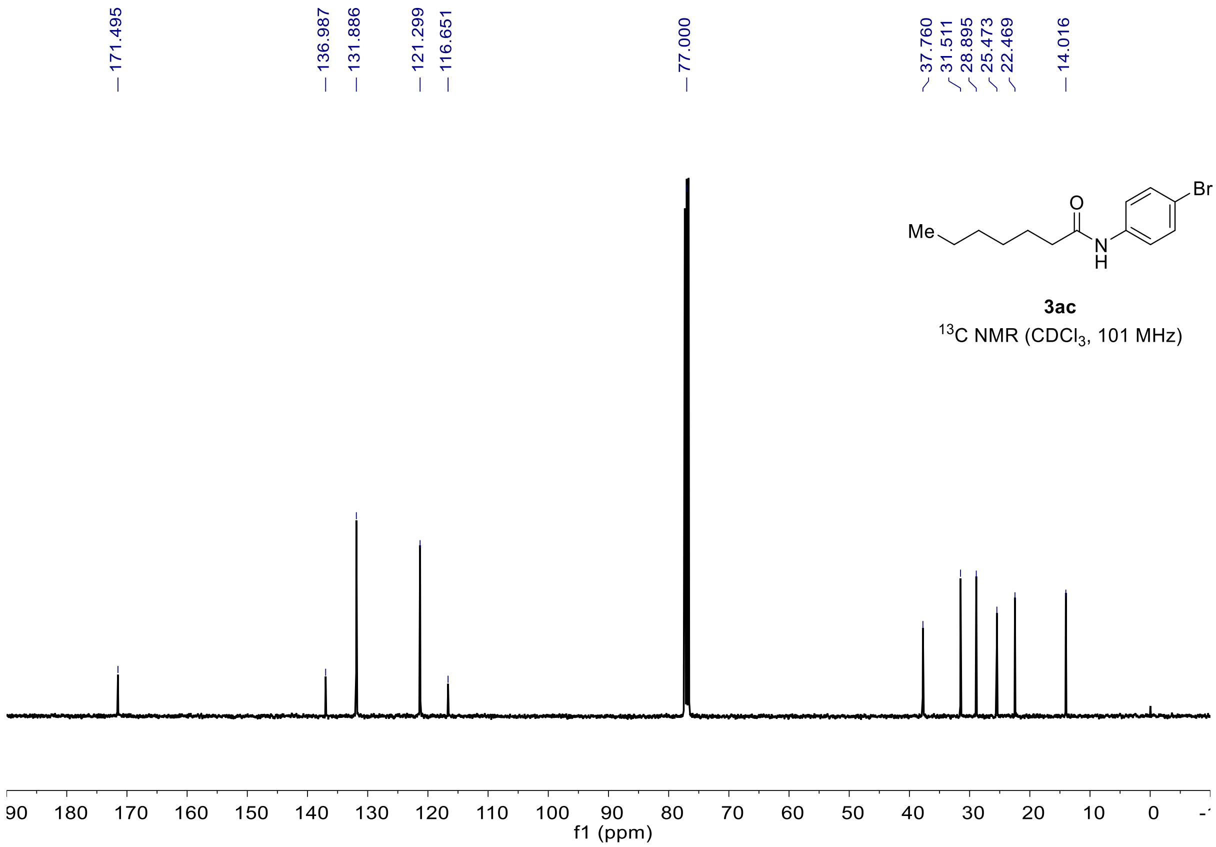
171.495
136.987
131.886
121.299
116.651
77.000
37.760
31.511
28.895
25.473
22.469
14.016
Me
O
N
H
Br
3ac
^{13}C NMR ($CDCl_3$, 101 MHz)
f1 (ppm)

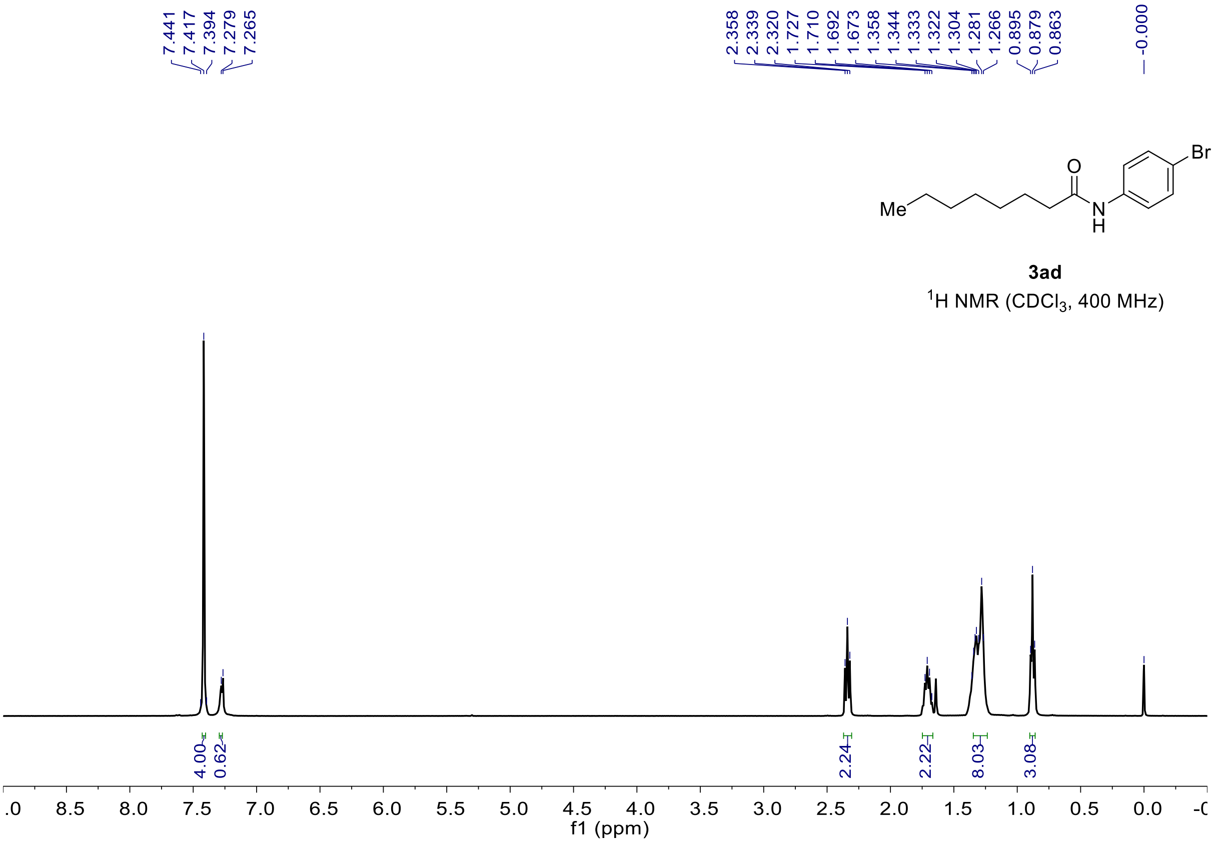

3ad
^{1}H NMR ($CDCl_3$, 400 MHz)

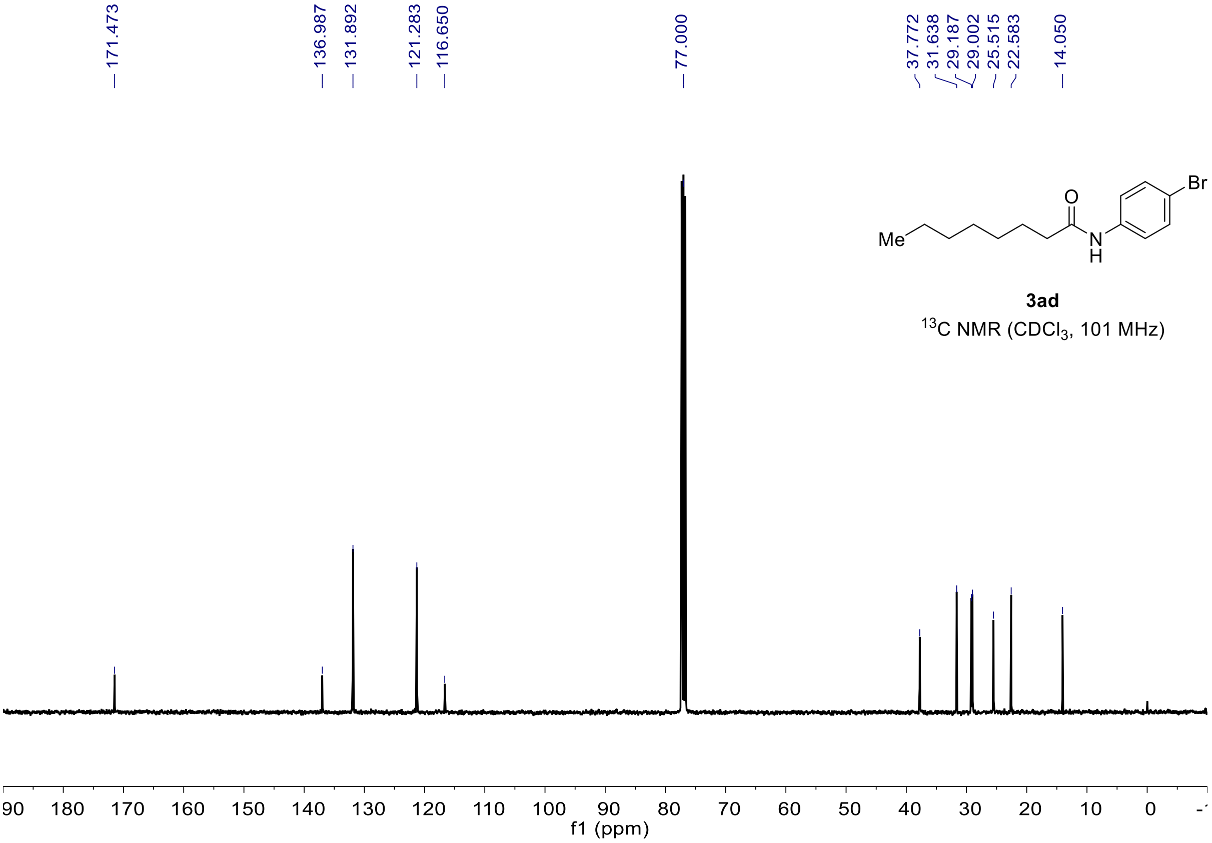

3ad
^{13}C NMR ($CDCl_3$, 101 MHz)

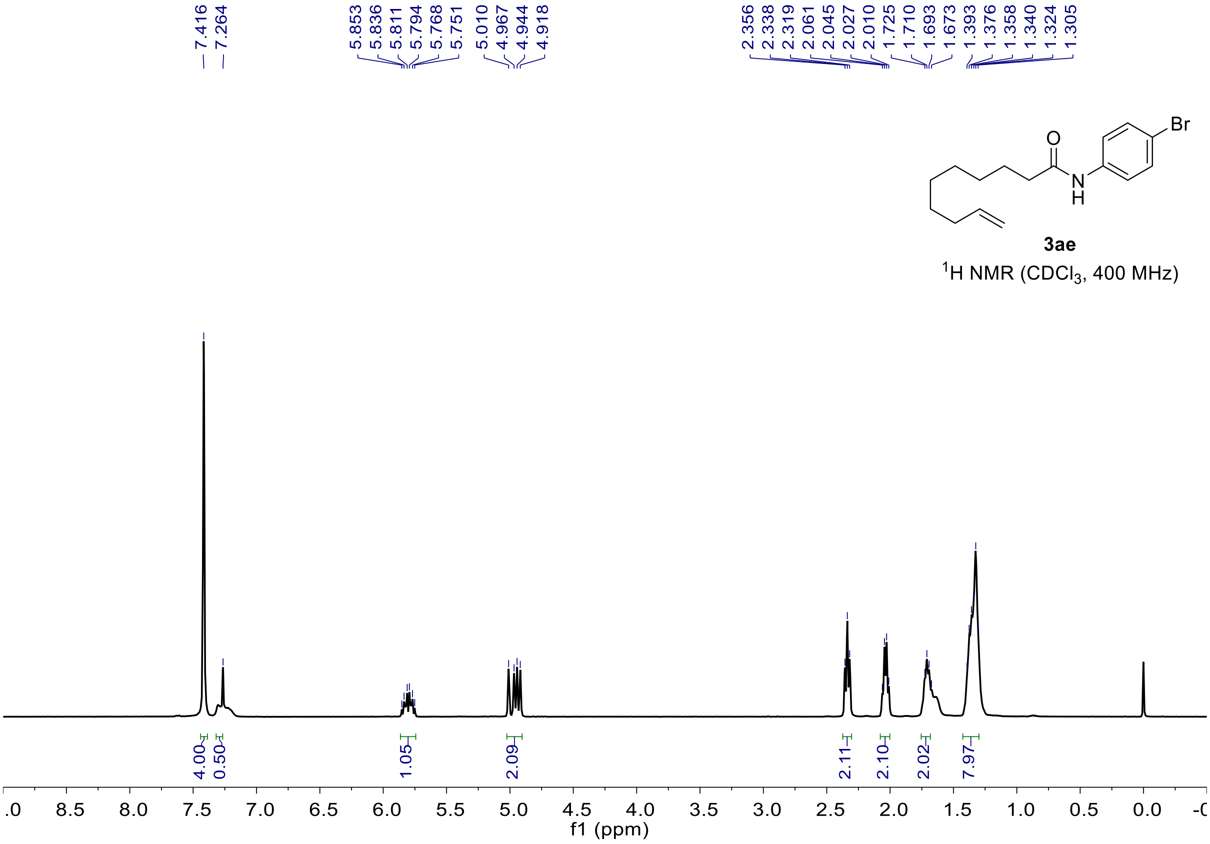

3ae

^{1}H NMR ($CDCl_3$, 400 MHz)

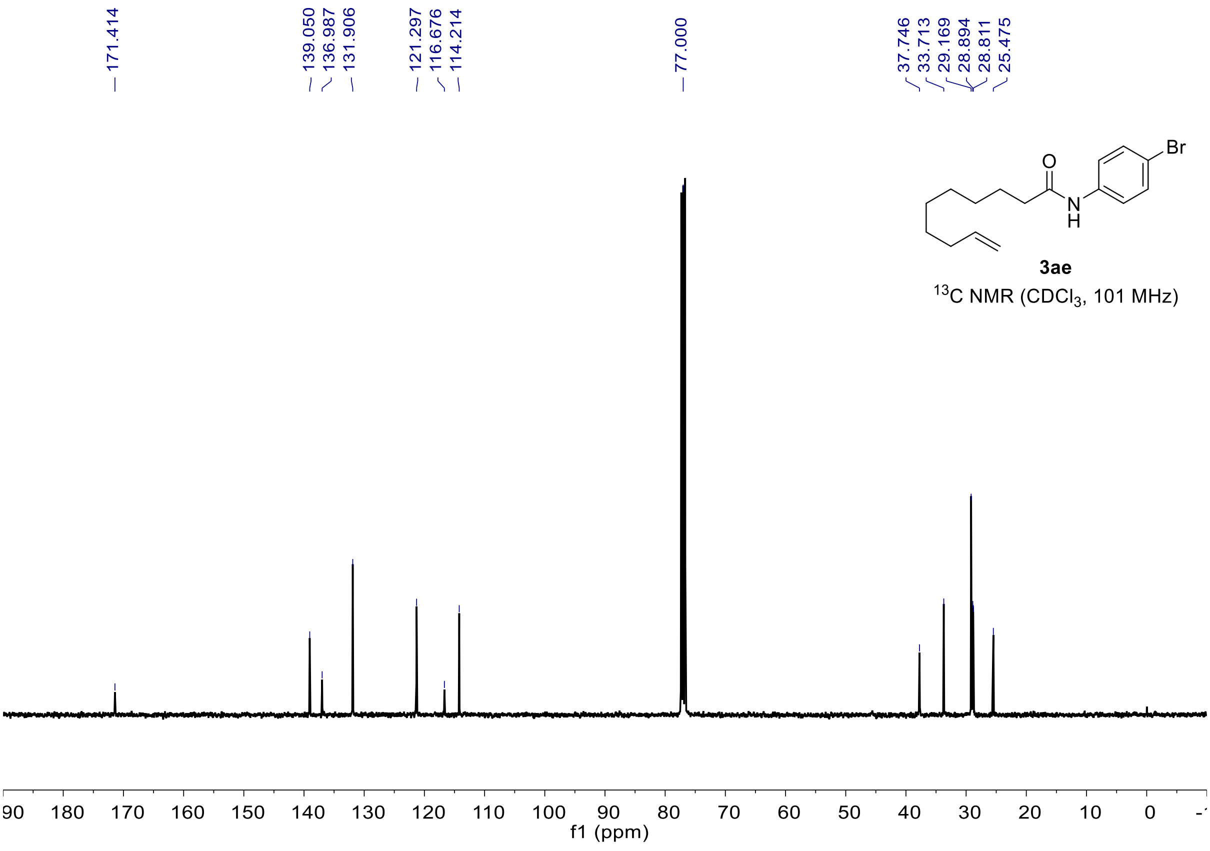

3ae

^{13}C NMR ($CDCl_3$, 101 MHz)

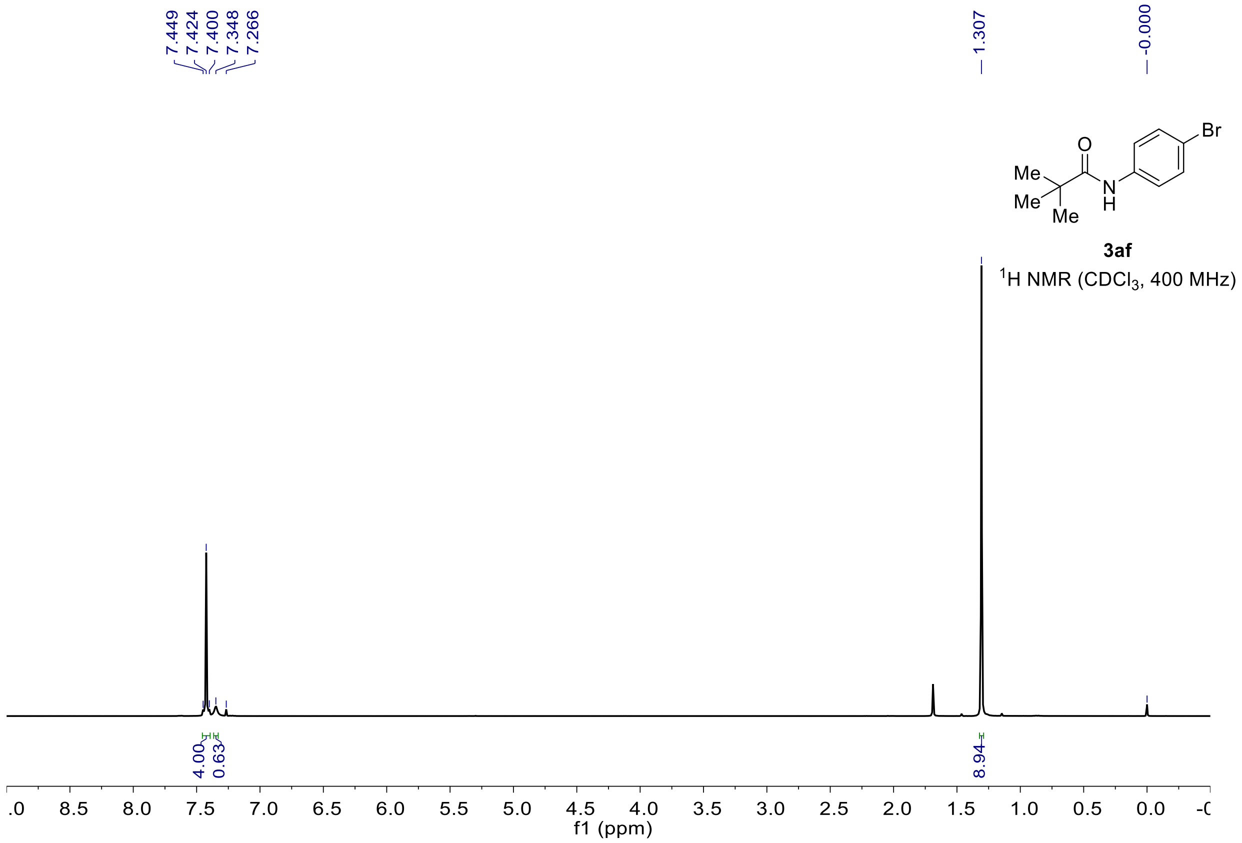
7.449
7.424
7.400
7.348
7.266
1.307
-0.000
Br
O
Me
N
Me
H
Me
3af
^{1}H NMR (CDCl$_3$, 400 MHz)
4.00
0.63
8.94
f1 (ppm)

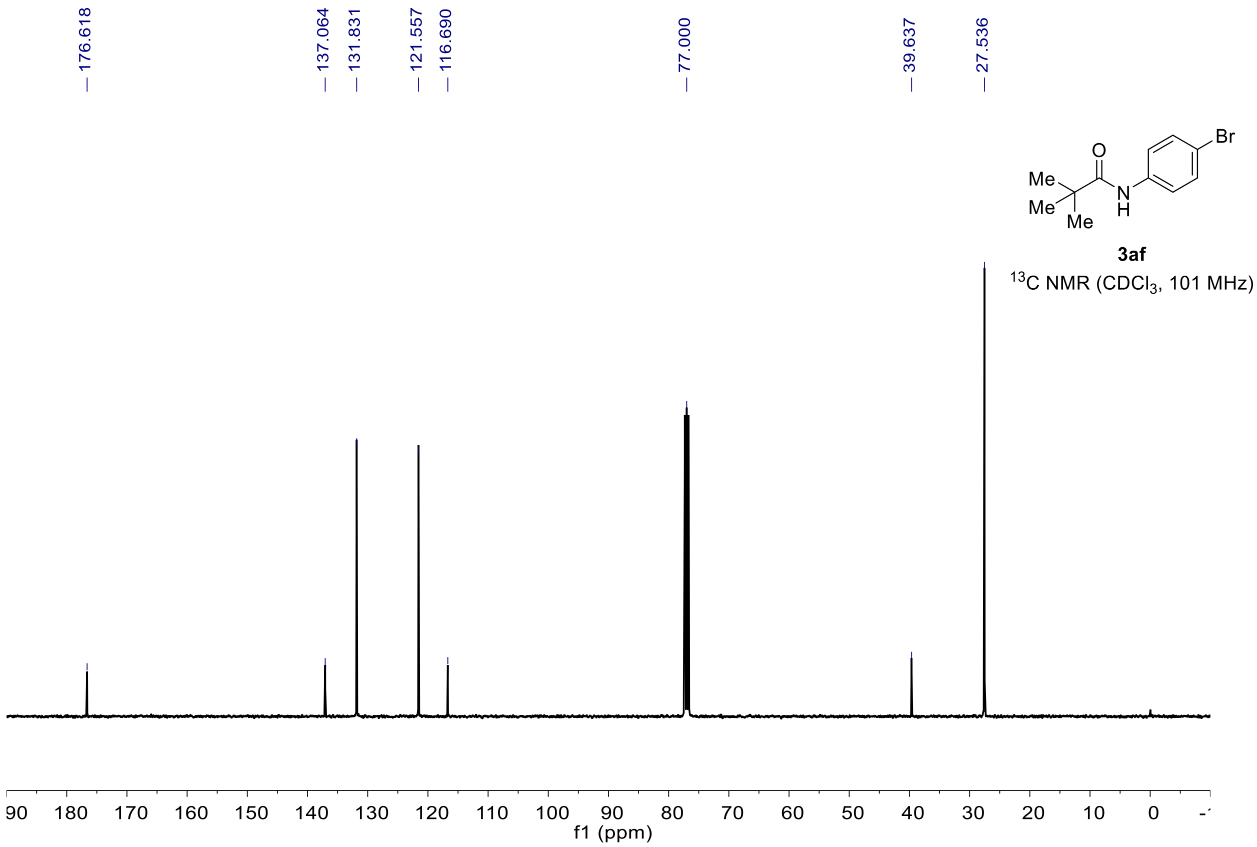
176.618
137.064
131.831
121.557
116.690
77.000
39.637
27.536
Br
O
Me
N
Me
H
Me
3af
^{13}C NMR (CDCl$_3$, 101 MHz)
f1 (ppm)

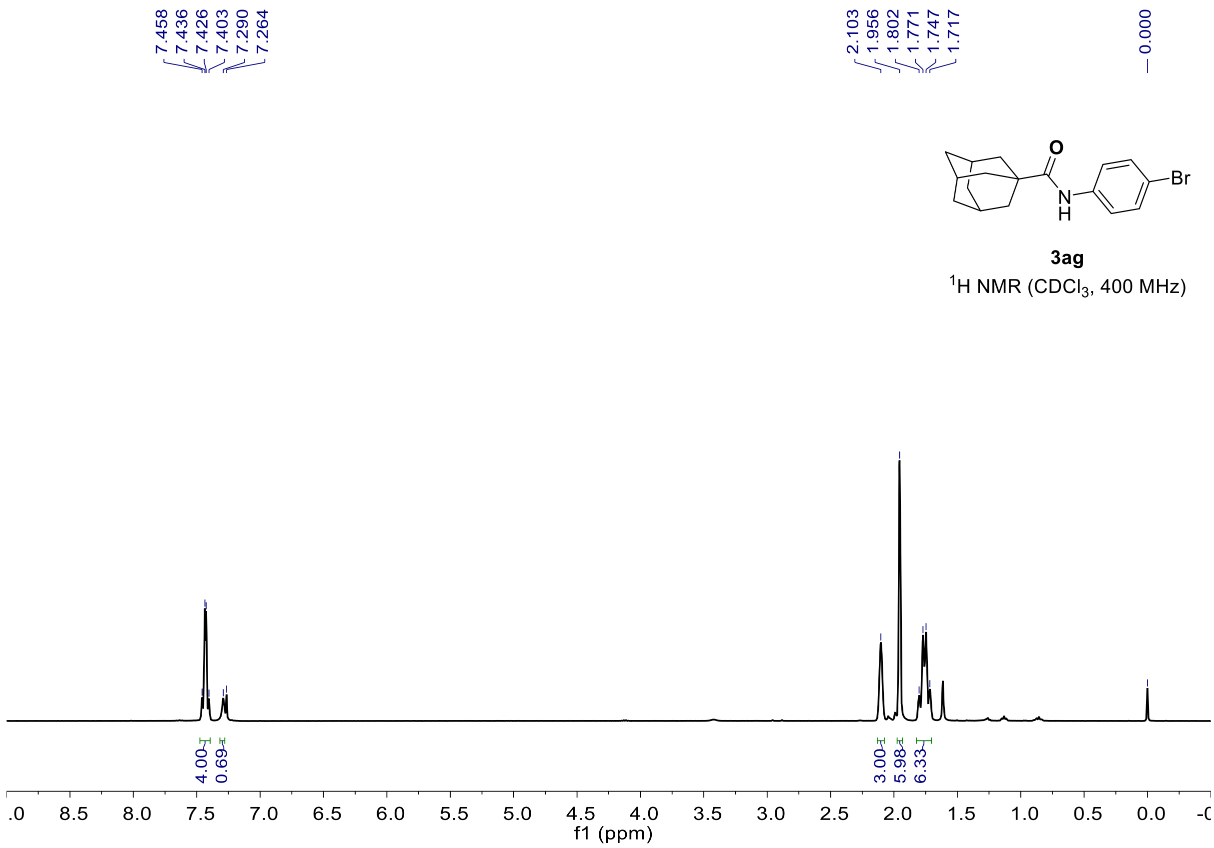

3ag
^{1}H NMR ($CDCl_3$, 400 MHz)

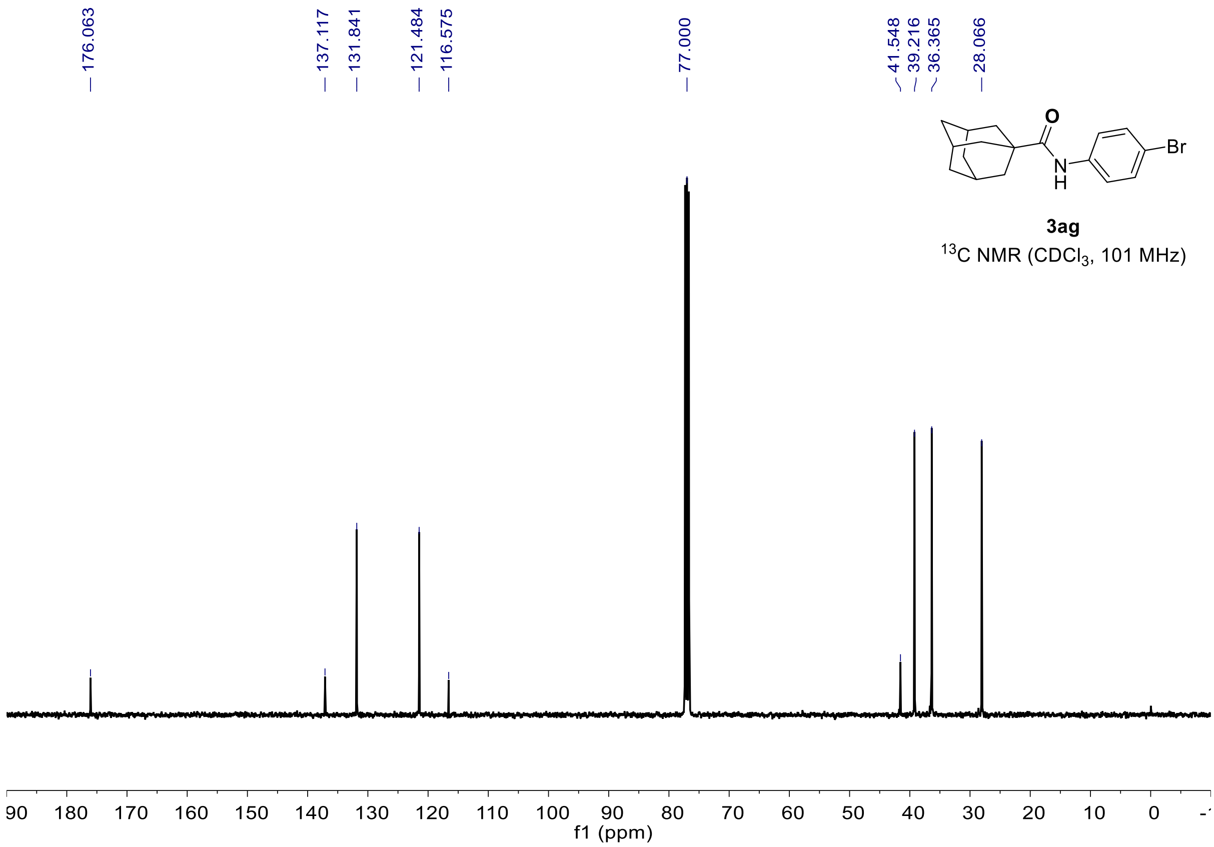

3ag
^{13}C NMR ($CDCl_3$, 101 MHz)

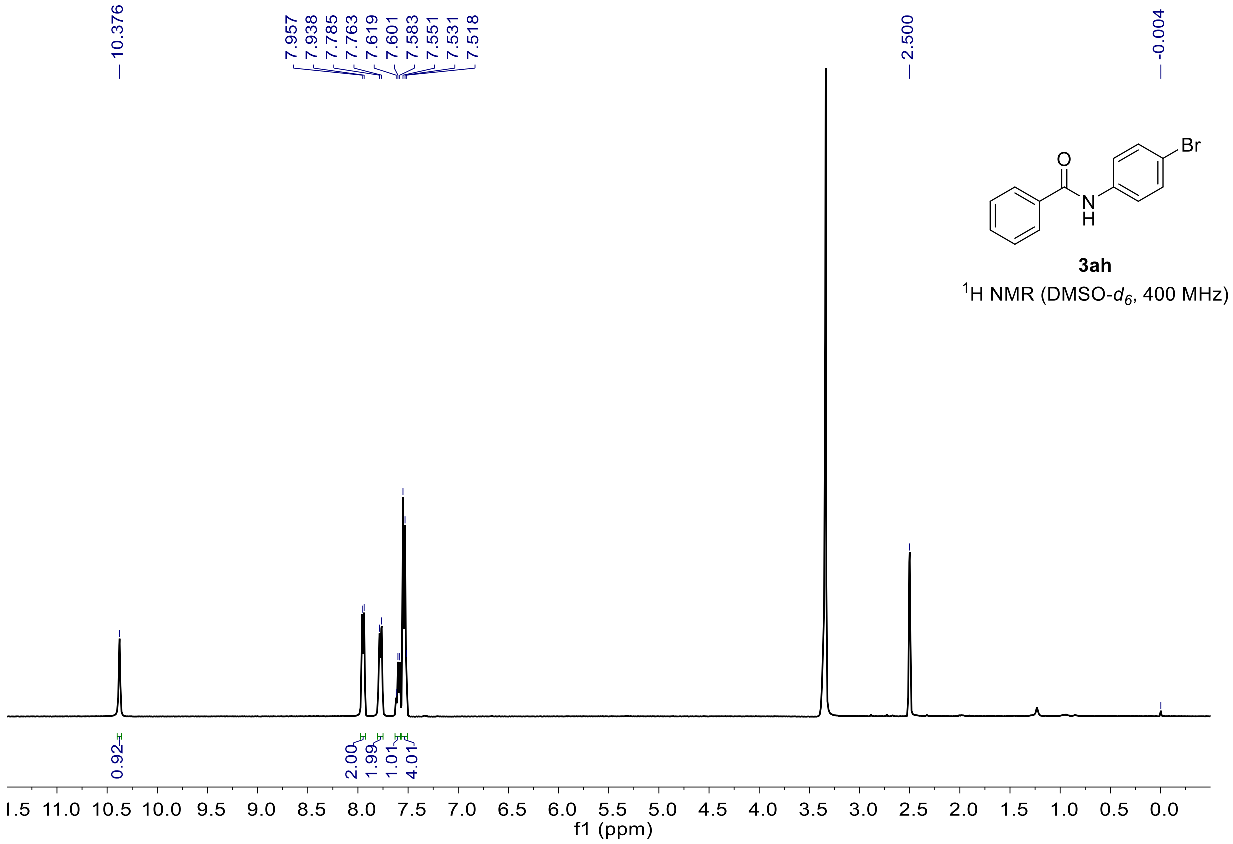
10.376
7.957
7.938
7.785
7.763
7.619
7.601
7.583
7.551
7.531
7.518
2.500
-0.004
O
N
H
Br
3ah
^{1}H NMR (DMSO-d_6, 400 MHz)
0.92
2.00
1.99
1.01
4.01
f1 (ppm)

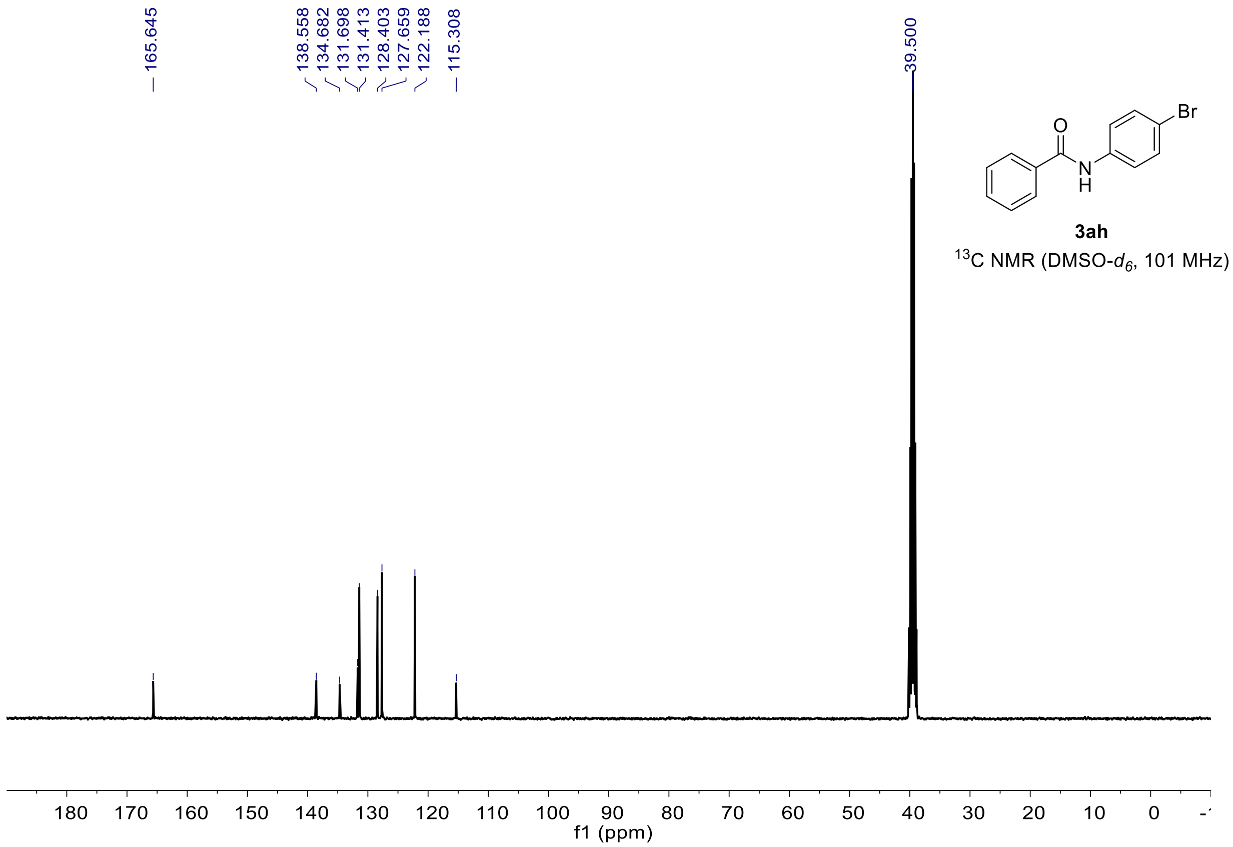
165.645
138.558
134.682
131.698
131.413
128.403
127.659
122.188
115.308
39.500
O
N
H
Br
3ah
^{13}C NMR (DMSO-d_6, 101 MHz)
f1 (ppm)

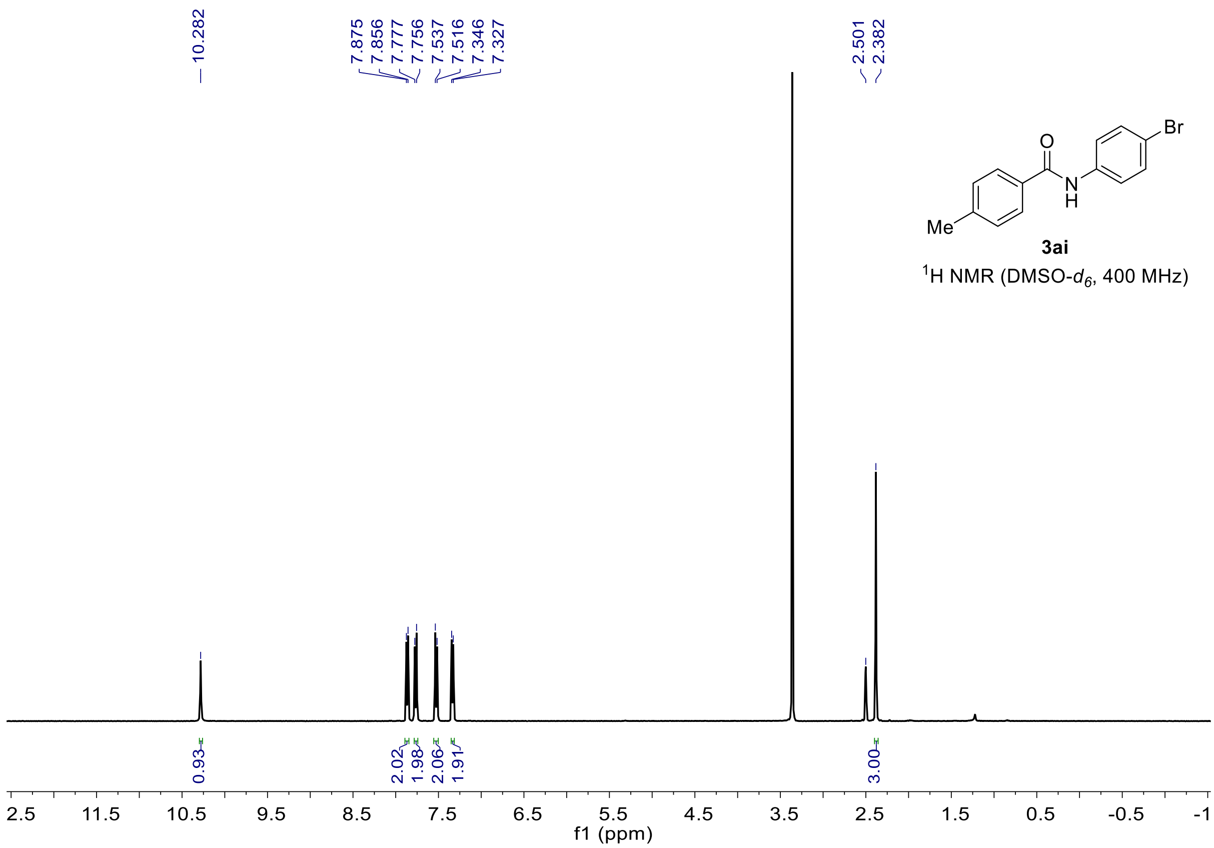

10.282
7.875
7.856
7.777
7.756
7.537
7.516
7.346
7.327
2.501
2.382
Br
O
N
H
Me
3ai
1H NMR (DMSO-d6, 400 MHz)
0.93
2.02
1.98
2.06
1.91
3.00
f1 (ppm)

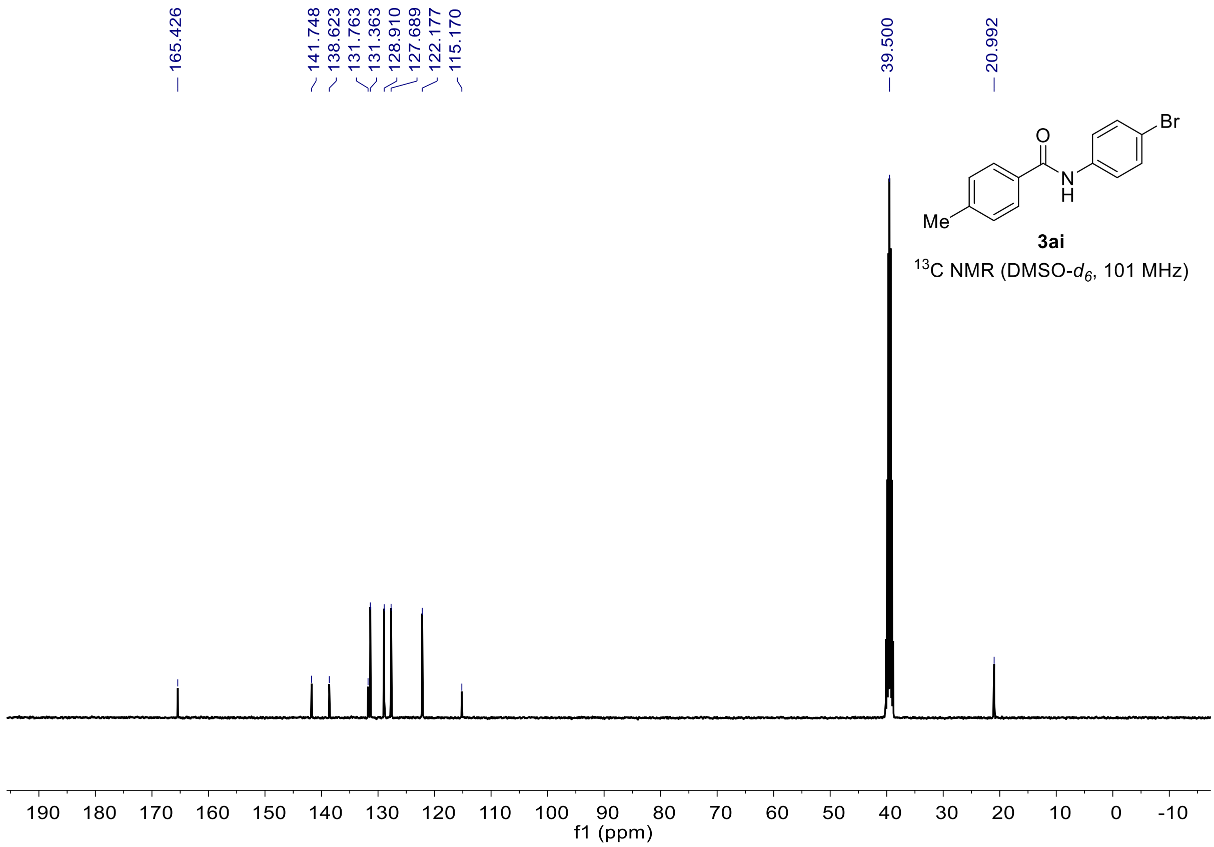

165.426
141.748
138.623
131.763
131.363
128.910
127.689
122.177
115.170
39.500
20.992
Br
O
N
H
Me
3ai
13C NMR (DMSO-d6, 101 MHz)
f1 (ppm)

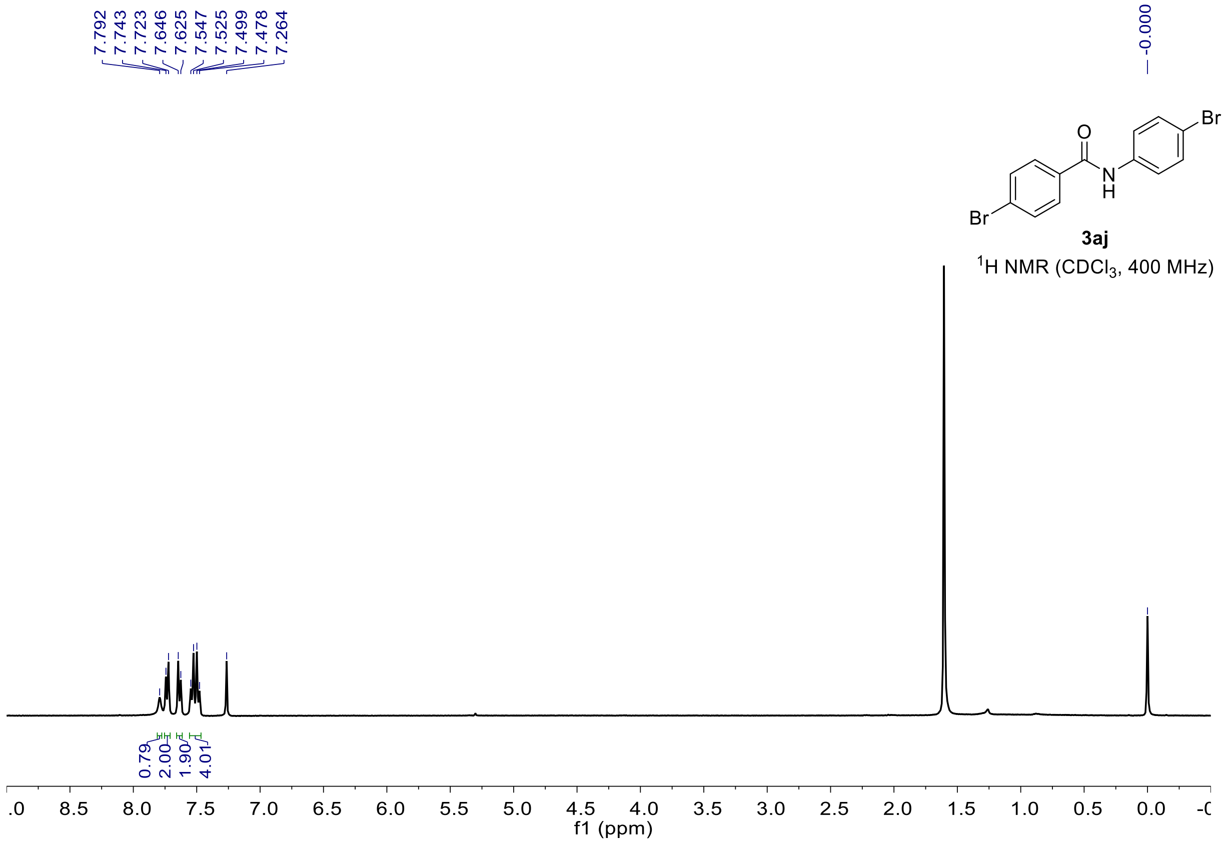
7.792
7.743
7.723
7.646
7.625
7.547
7.525
7.499
7.478
7.264
-0.000
3aj
^{1}H NMR ($CDCl_3$, 400 MHz)
0.79
2.00
1.90
4.01
f1 (ppm)

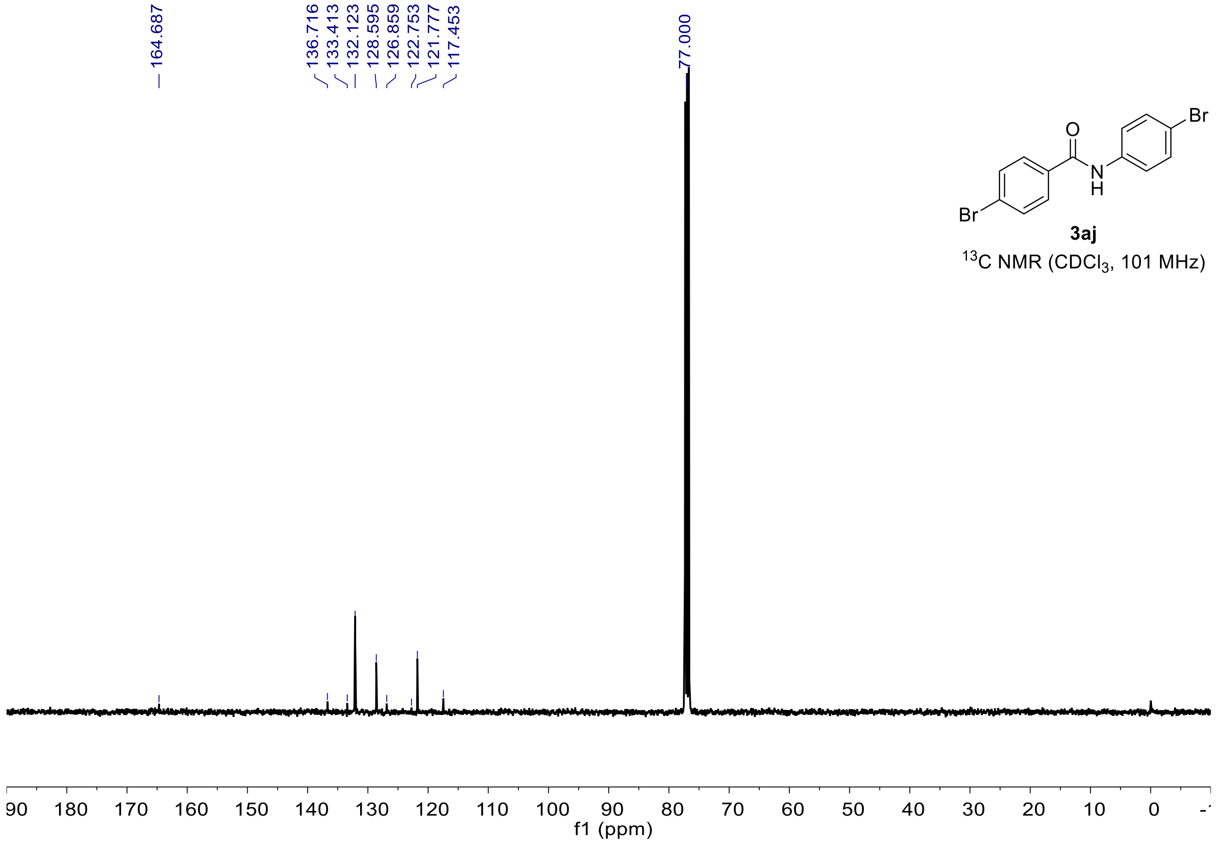
164.687
136.716
133.413
132.123
128.595
126.859
122.753
121.777
117.453
77.000
3aj
^{13}C NMR ($CDCl_3$, 101 MHz)
f1 (ppm)

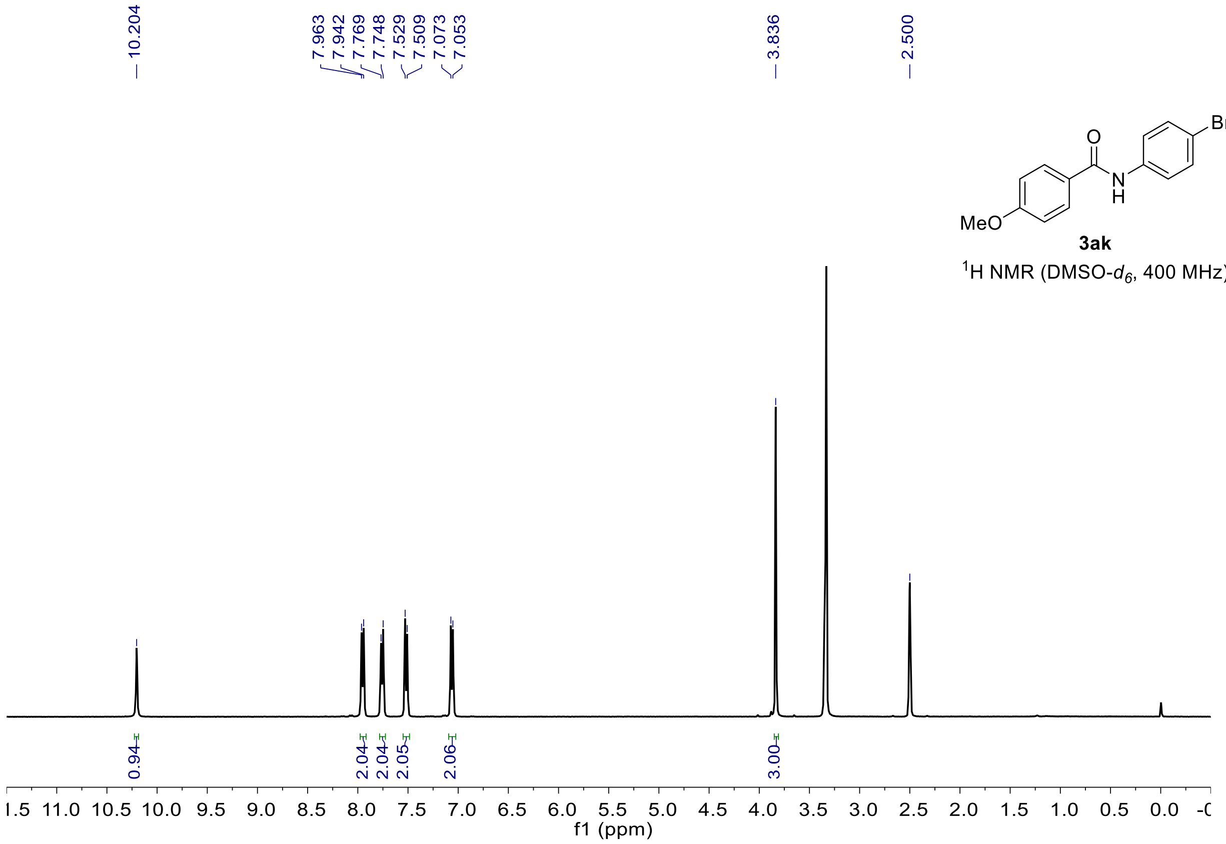

3ak

^{1}H NMR (DMSO-d_6, 400 MHz)

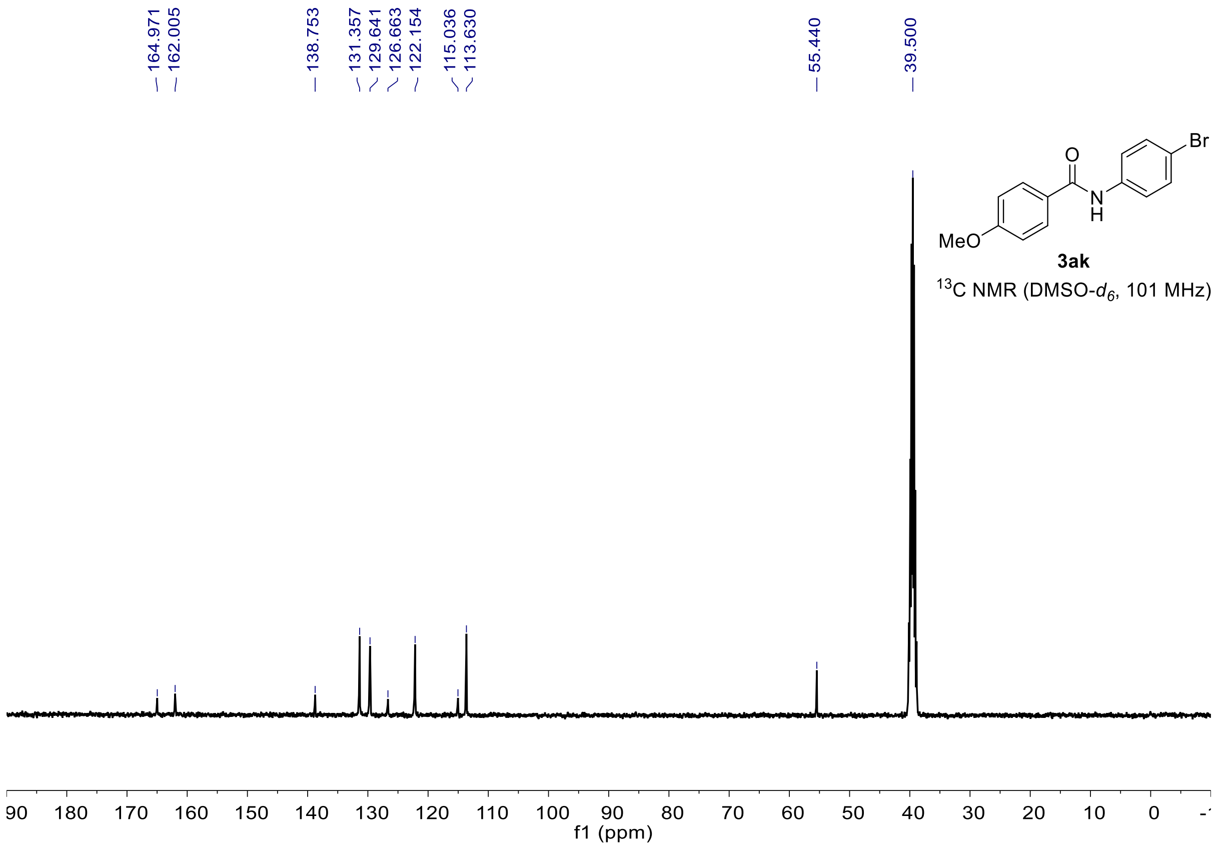

3ak

^{13}C NMR (DMSO-d_6, 101 MHz)

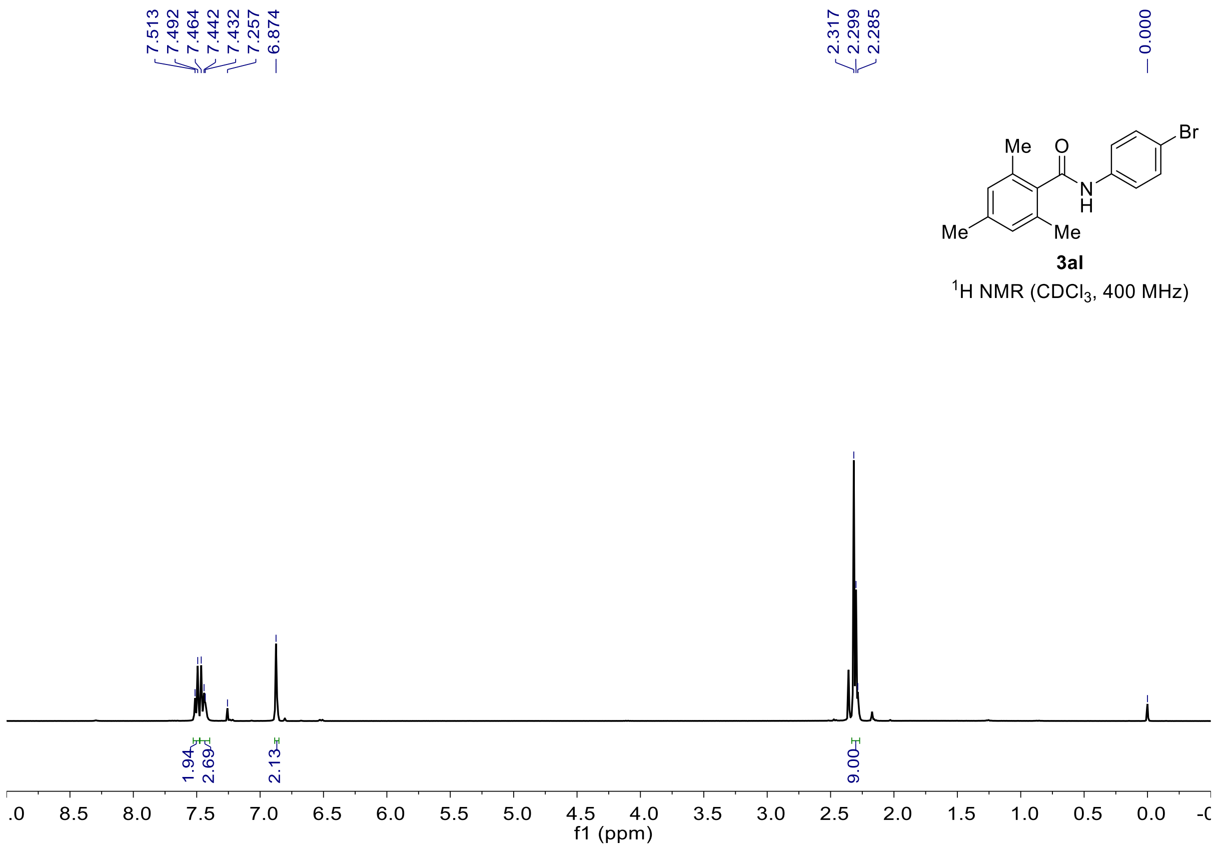
7.513
7.492
7.464
7.442
7.432
7.257
6.874
2.317
2.299
2.285
0.000
Me
O
Br
N
H
Me
Me
3al
^{1}H NMR ($CDCl_3$, 400 MHz)
1.94
2.69
2.13
9.00
f1 (ppm)

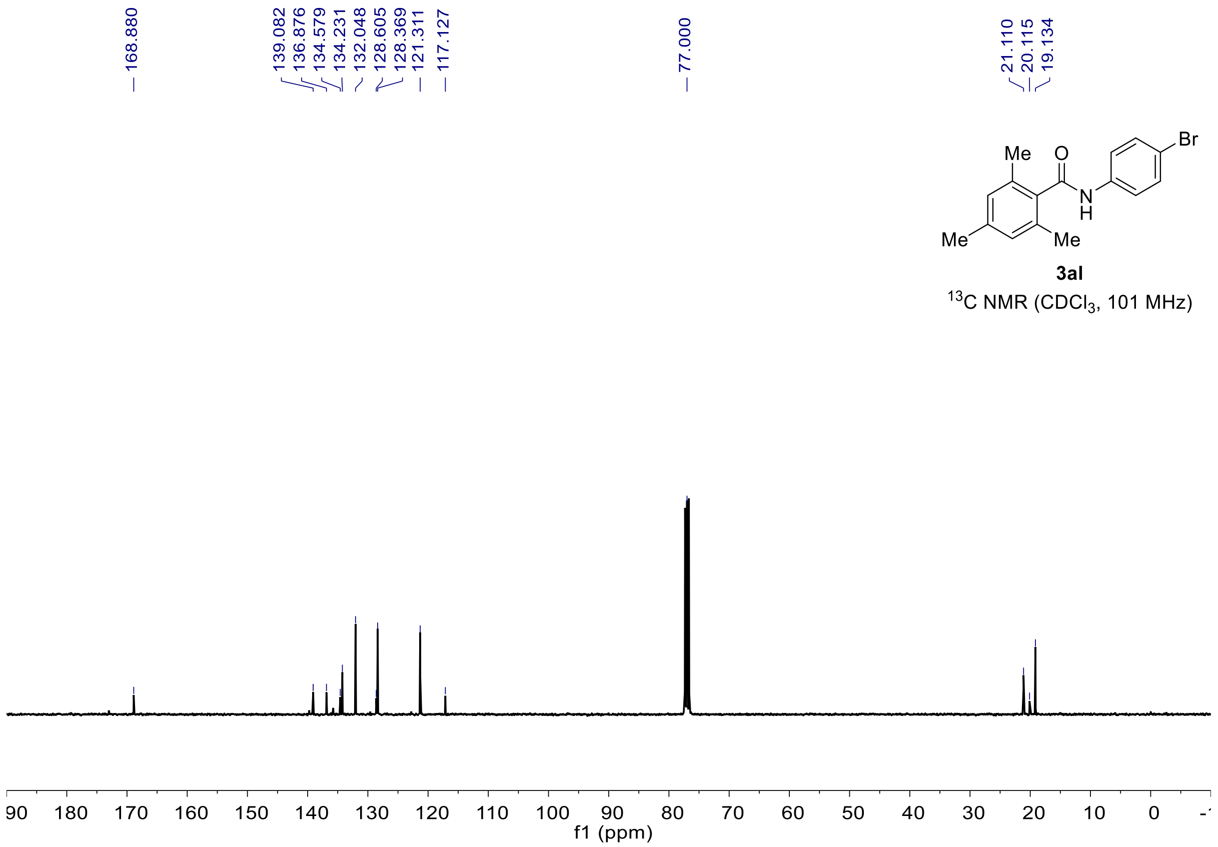
168.880
139.082
136.876
134.579
134.231
132.048
128.605
128.369
121.311
117.127
77.000
21.110
20.115
19.134
Me
O
Br
N
H
Me
Me
3al
^{13}C NMR ($CDCl_3$, 101 MHz)
f1 (ppm)

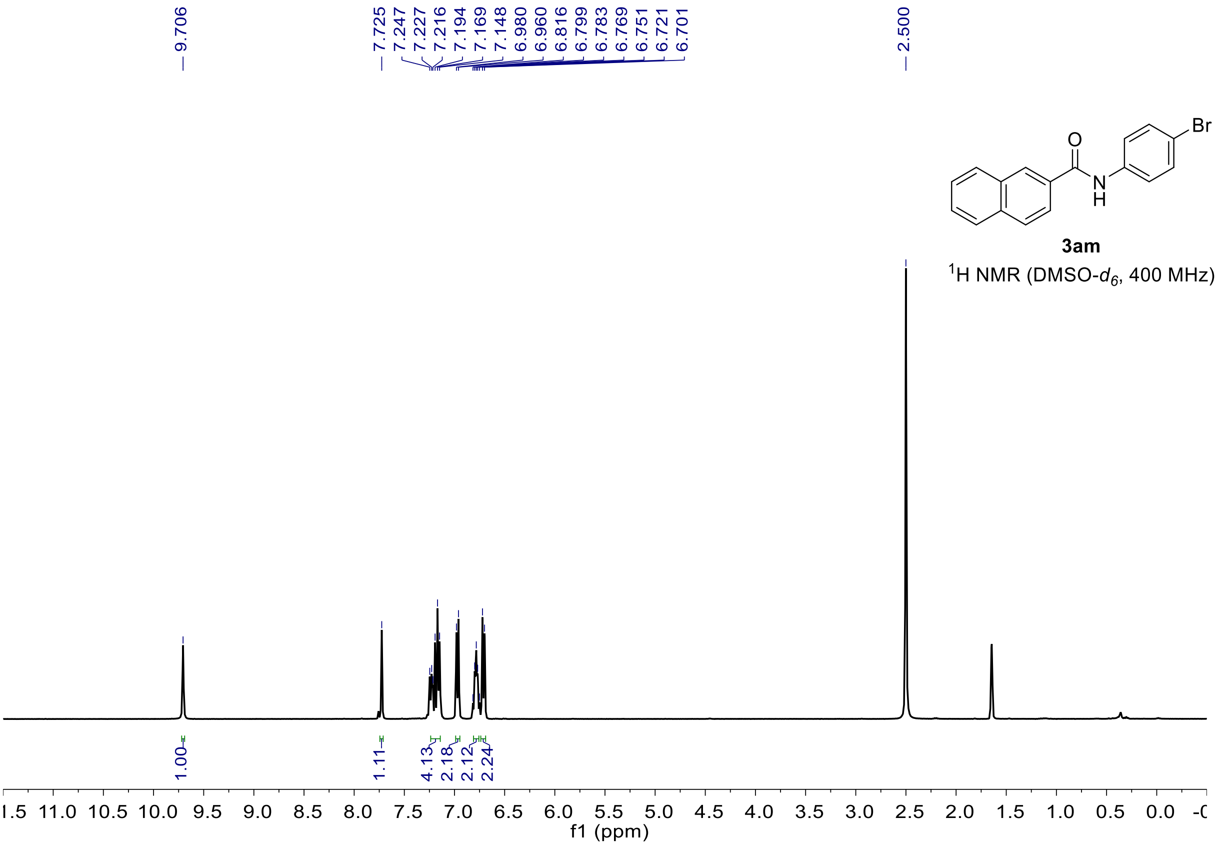
9.706
7.725
7.247
7.227
7.216
7.194
7.169
7.148
6.980
6.960
6.816
6.799
6.783
6.769
6.751
6.721
6.701
2.500
O
N
H
Br
3am
^{1}H NMR (DMSO-d_6, 400 MHz)
1.00
1.11
4.13
2.18
2.12
2.24
f1 (ppm)

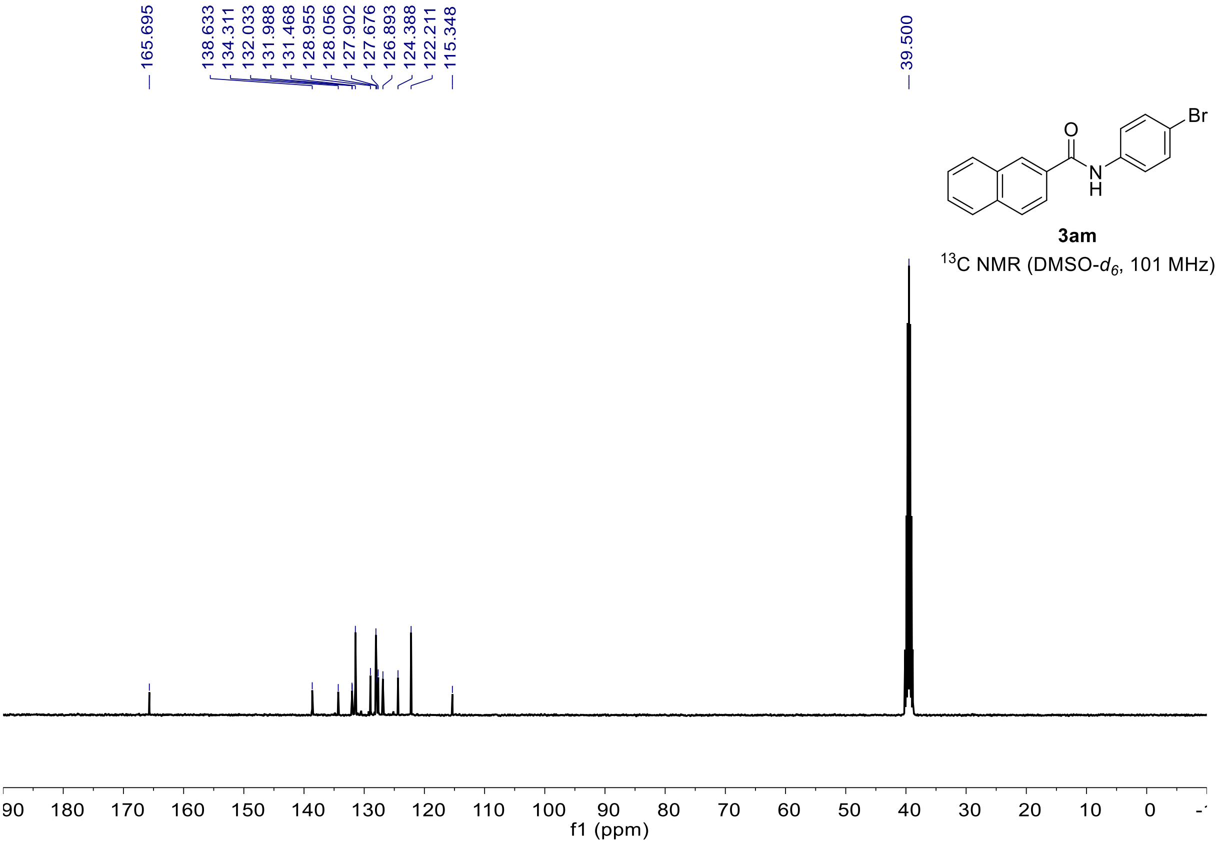
165.695
138.633
134.311
132.033
131.988
131.468
128.955
128.056
127.902
127.676
126.893
124.388
122.211
115.348
39.500
O
N
H
Br
3am
^{13}C NMR (DMSO-d_6, 101 MHz)
f1 (ppm)

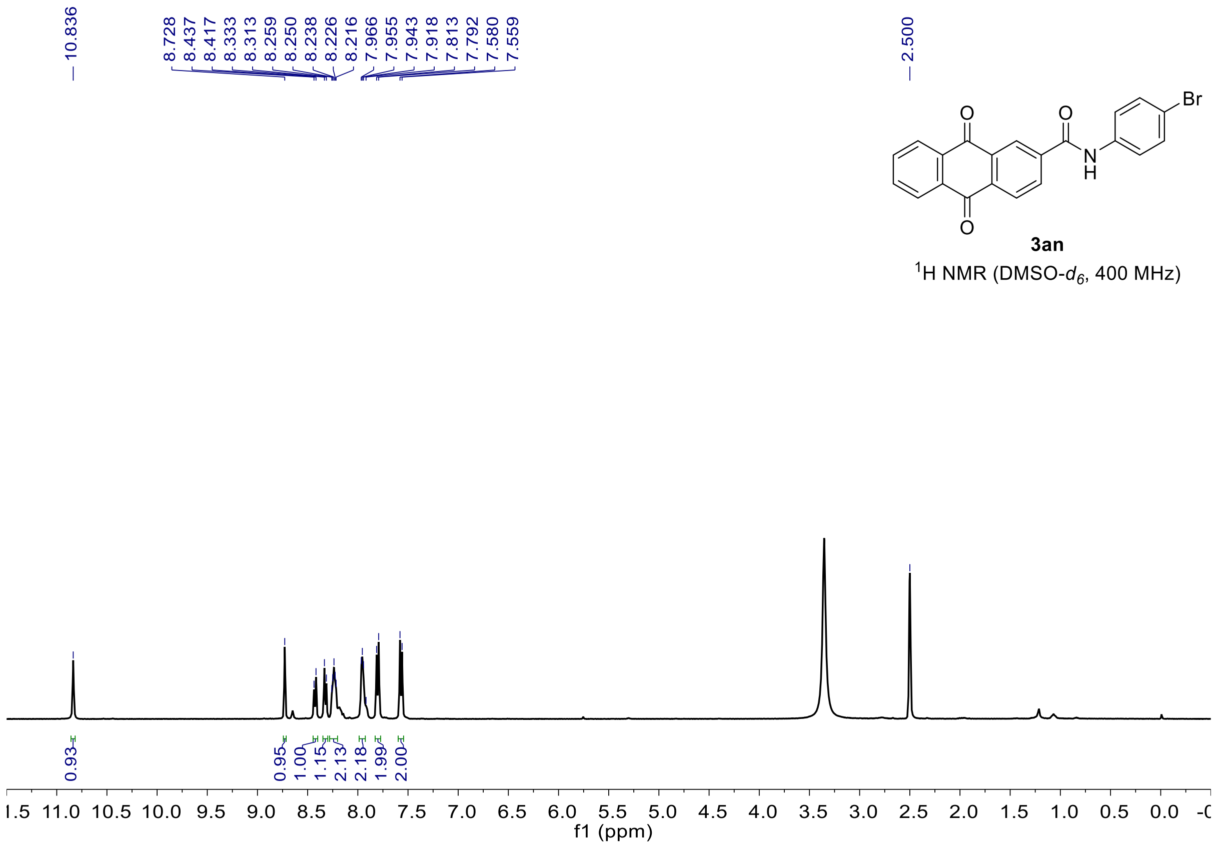

3an

^{1}H NMR (DMSO-d_6, 400 MHz)

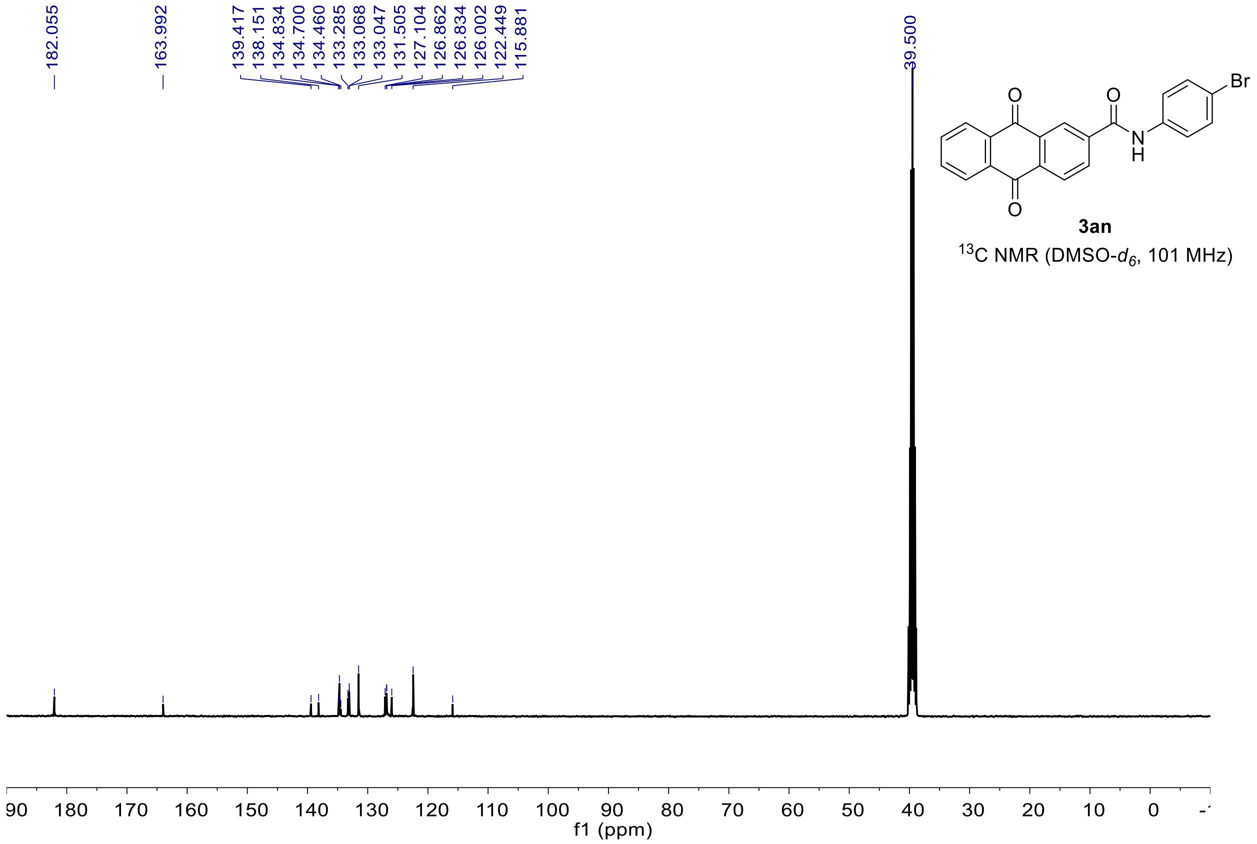

3an

^{13}C NMR (DMSO-d_6, 101 MHz)

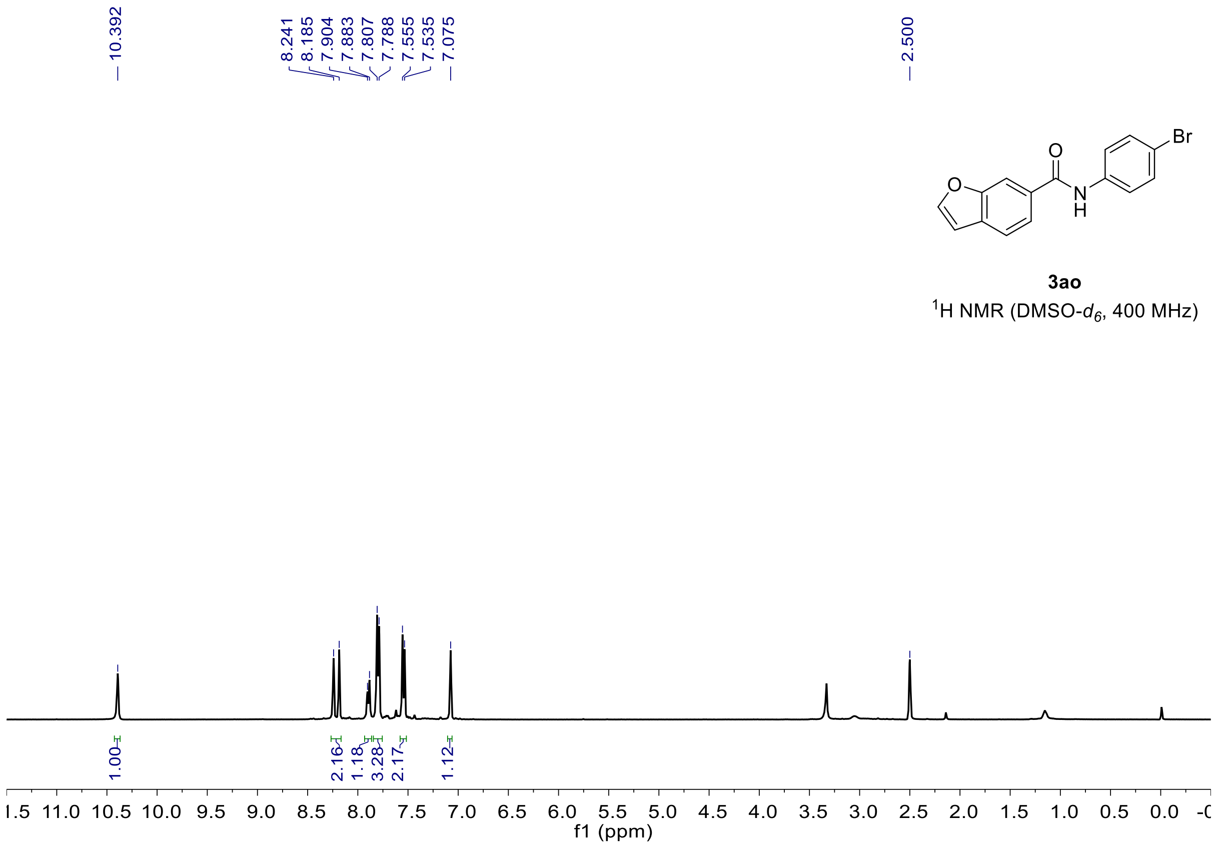

3ao
^{1}H NMR (DMSO-d_6, 400 MHz)

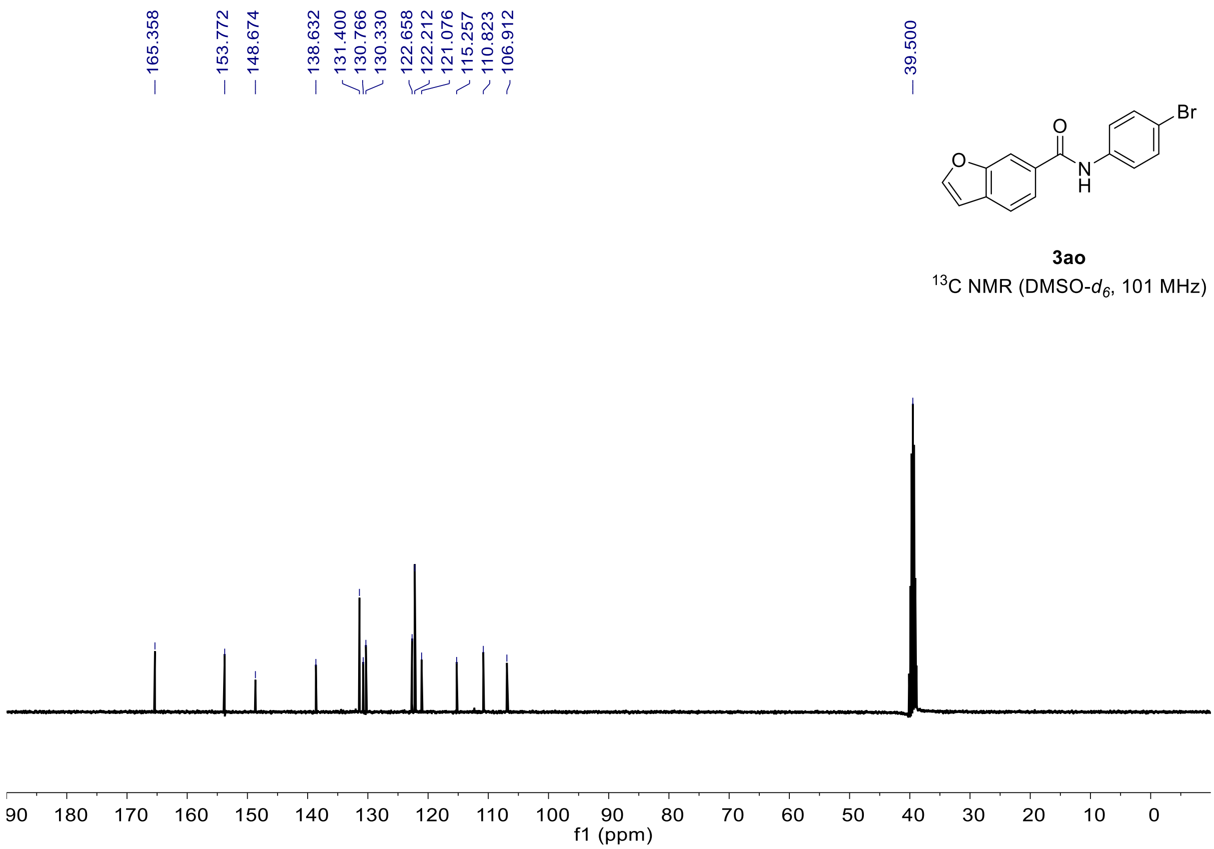

3ao
^{13}C NMR (DMSO-d_6, 101 MHz)

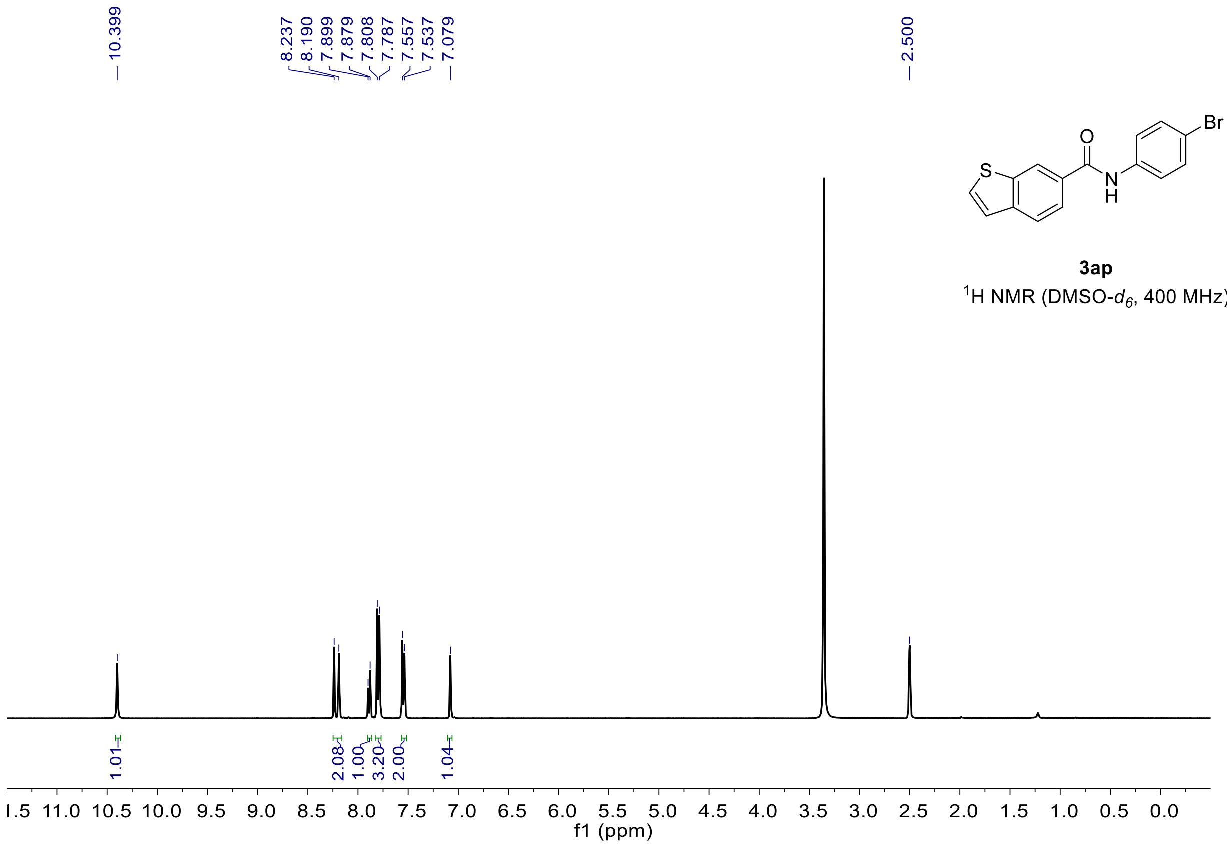

3ap

^{1}H NMR (DMSO-d_6, 400 MHz)

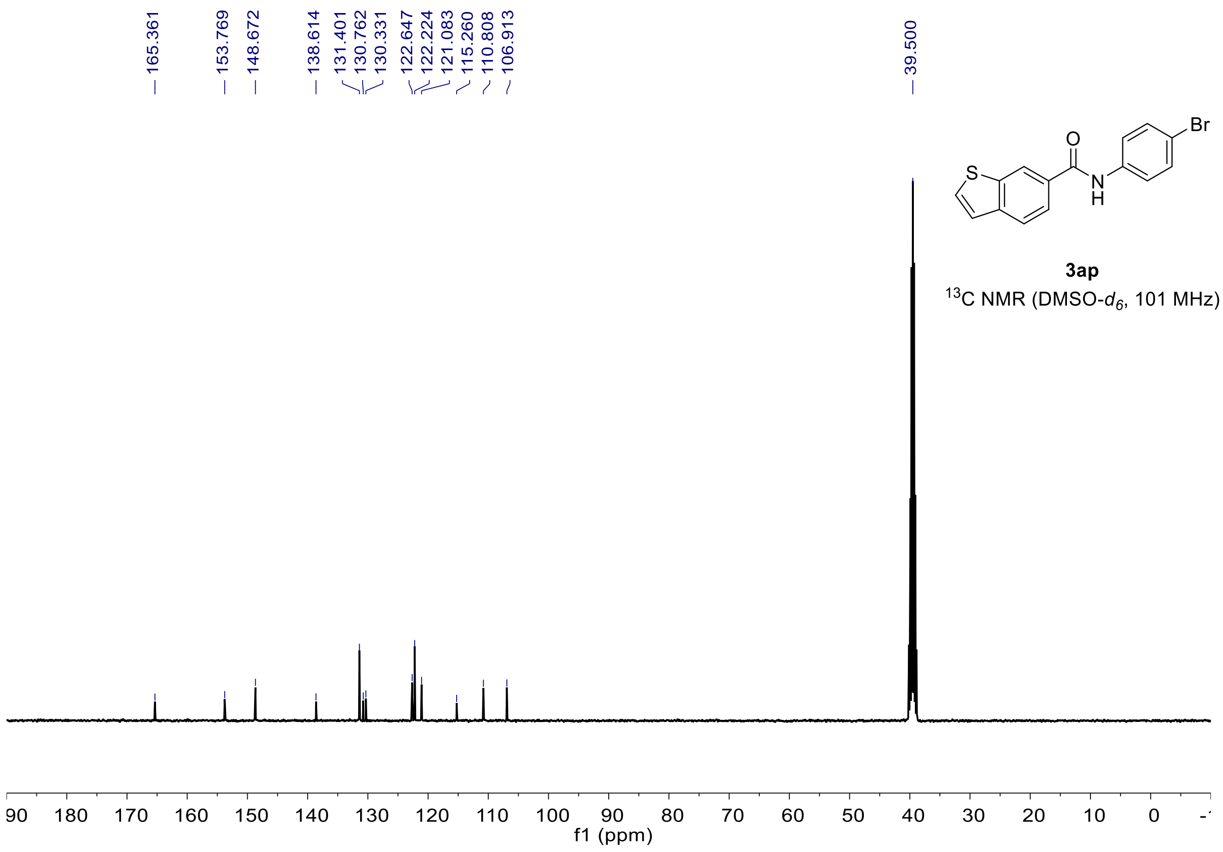

3ap

^{13}C NMR (DMSO-d_6, 101 MHz)

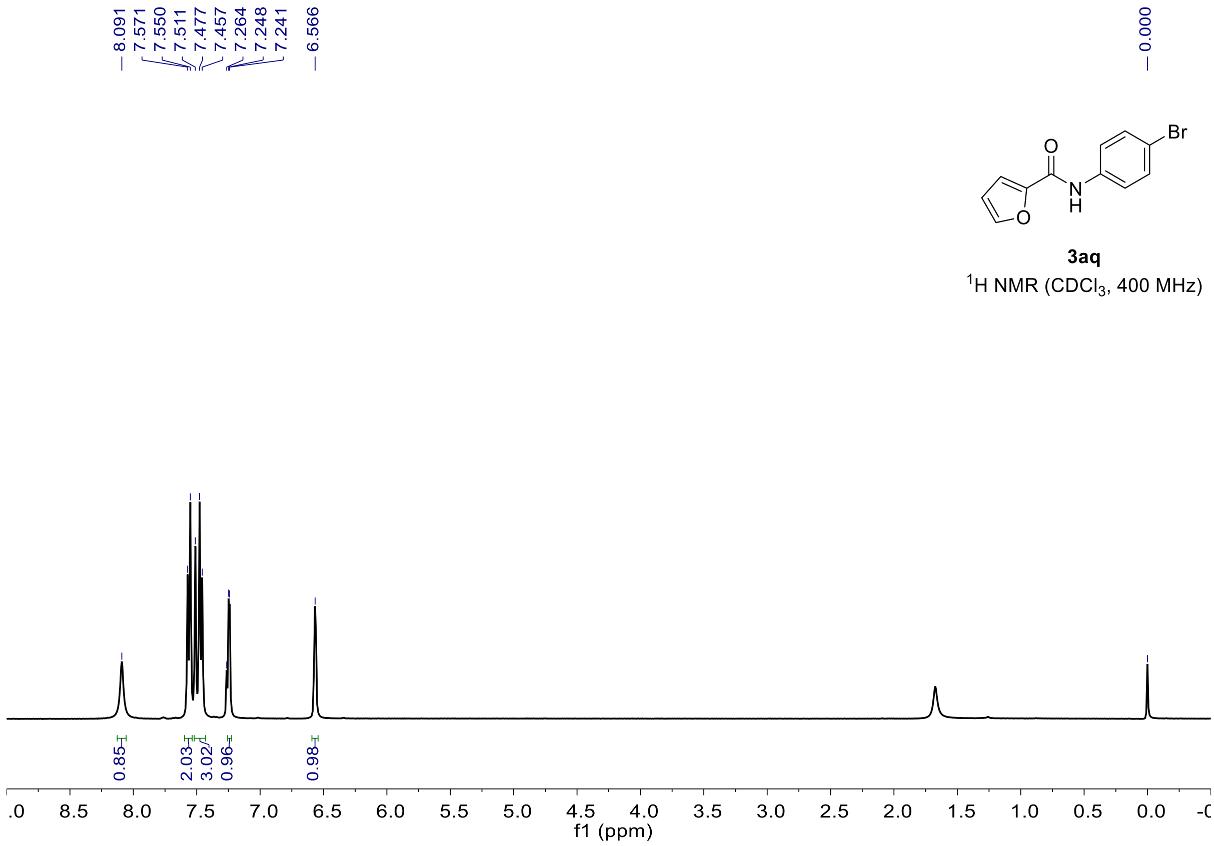
8.091
7.571
7.550
7.511
7.477
7.457
7.264
7.248
7.241
6.566
0.000
O
N
H
O
Br
3aq
^{1}H NMR ($CDCl_3$, 400 MHz)
0.85
2.03
3.02
0.96
0.98
f1 (ppm)

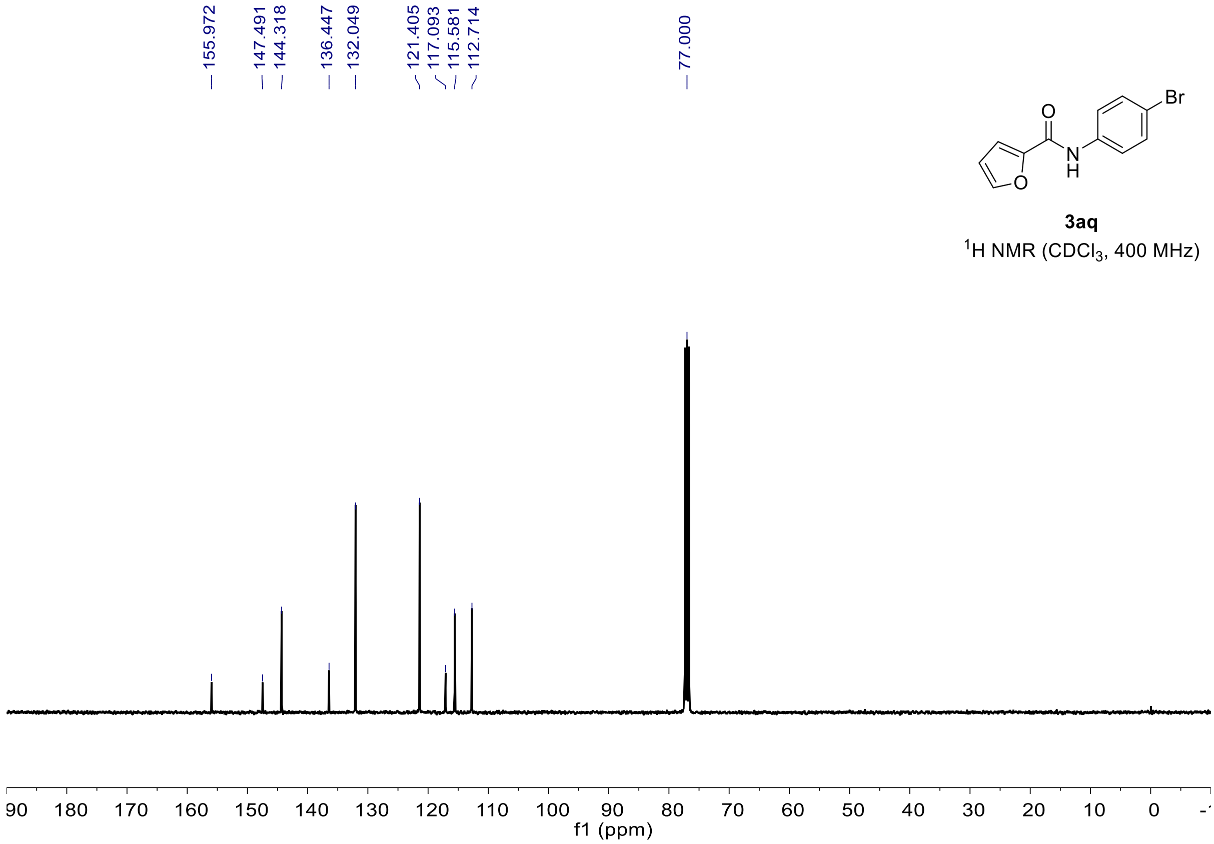
155.972
147.491
144.318
136.447
132.049
121.405
117.093
115.581
112.714
77.000
O
N
H
O
Br
3aq
^{1}H NMR ($CDCl_3$, 400 MHz)
f1 (ppm)

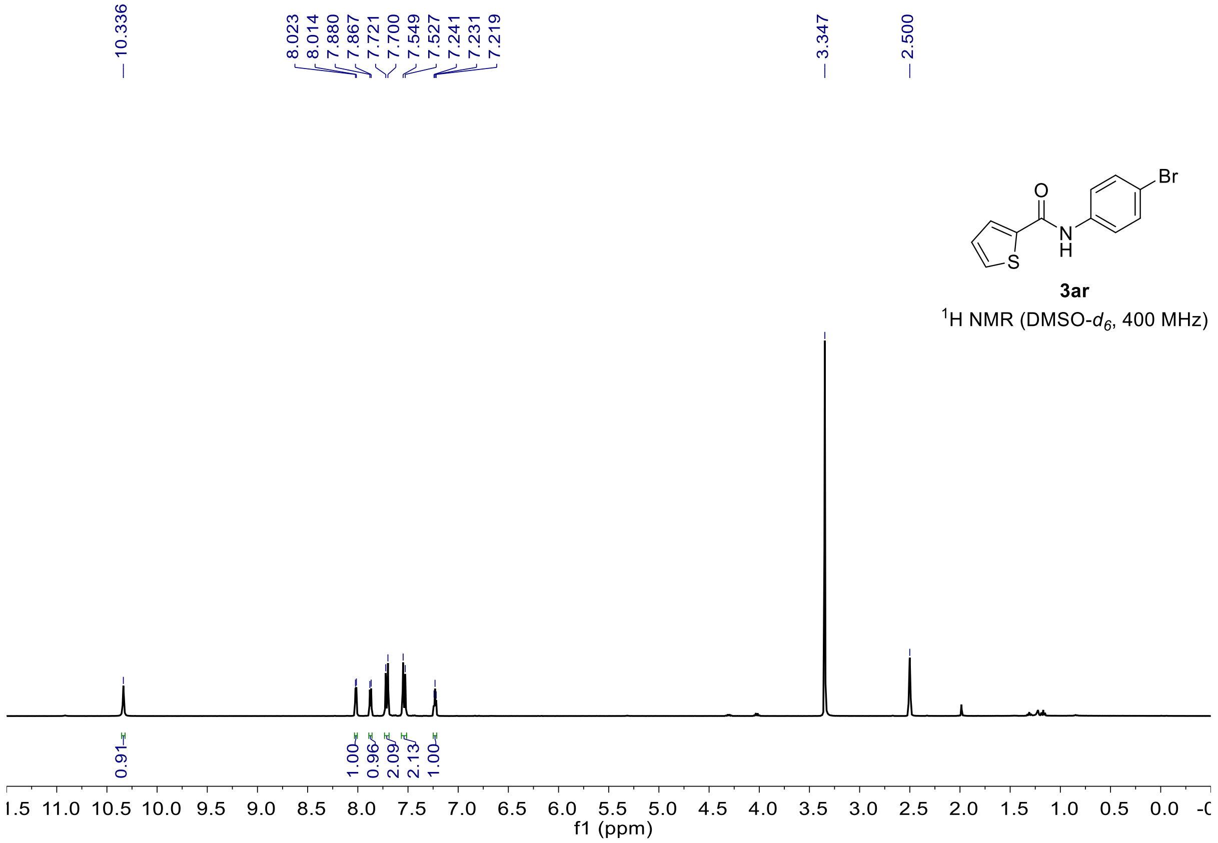
10.336
8.023
8.014
7.880
7.867
7.721
7.700
7.549
7.527
7.241
7.231
7.219
3.347
2.500
Br
O
N
H
S
3ar
1H NMR (DMSO-d6, 400 MHz)
0.91
1.00
0.96
2.09
2.13
1.00
f1 (ppm)

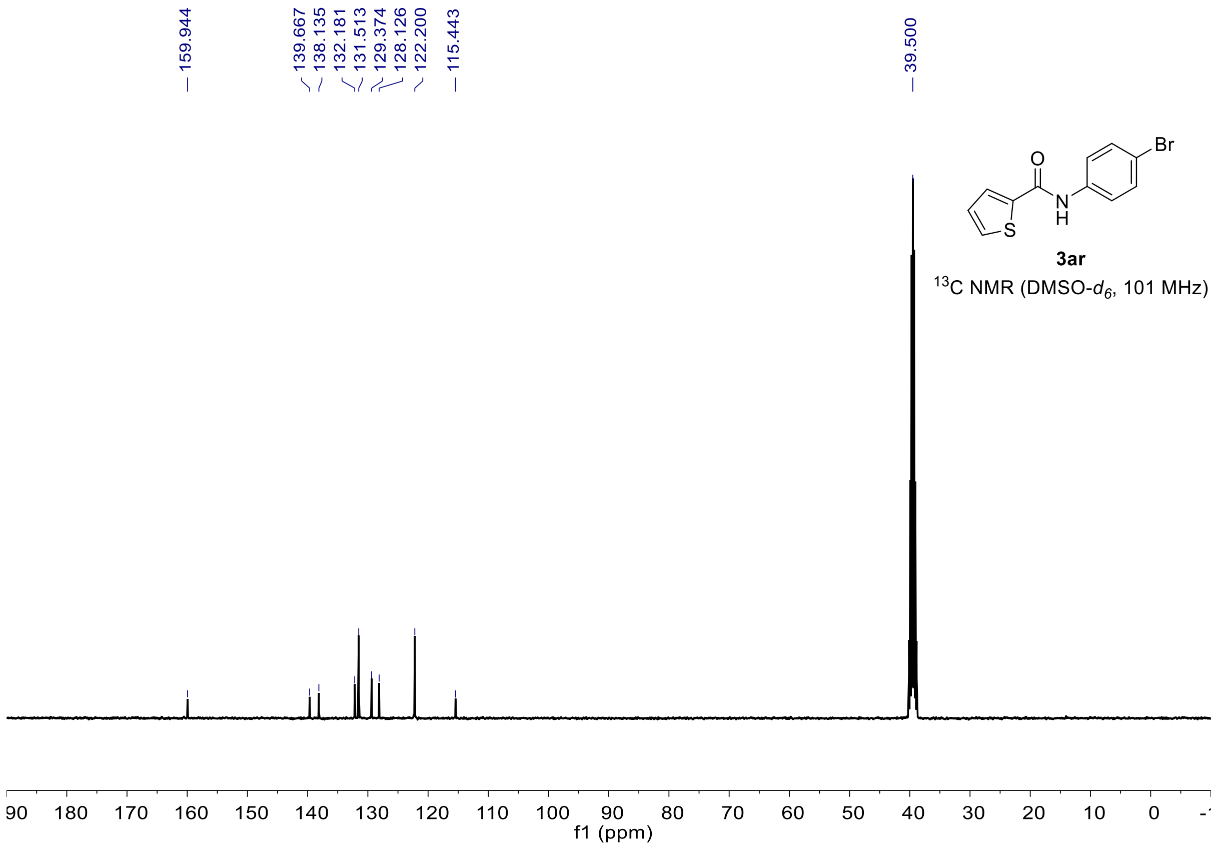
159.944
139.667
138.135
132.181
131.513
129.374
128.126
122.200
115.443
39.500
Br
O
N
H
S
3ar
13C NMR (DMSO-d6, 101 MHz)
f1 (ppm)

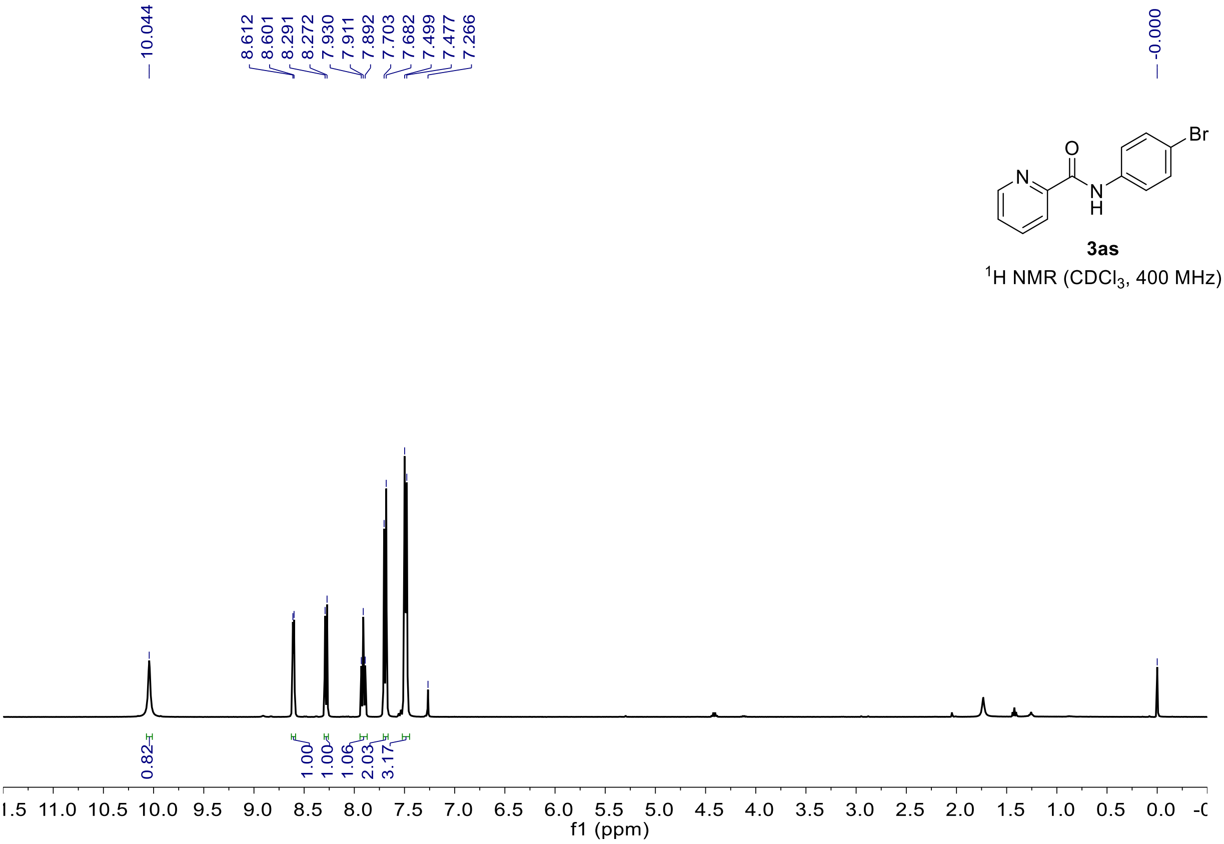

3as

^{1}H NMR ($CDCl_3$, 400 MHz)

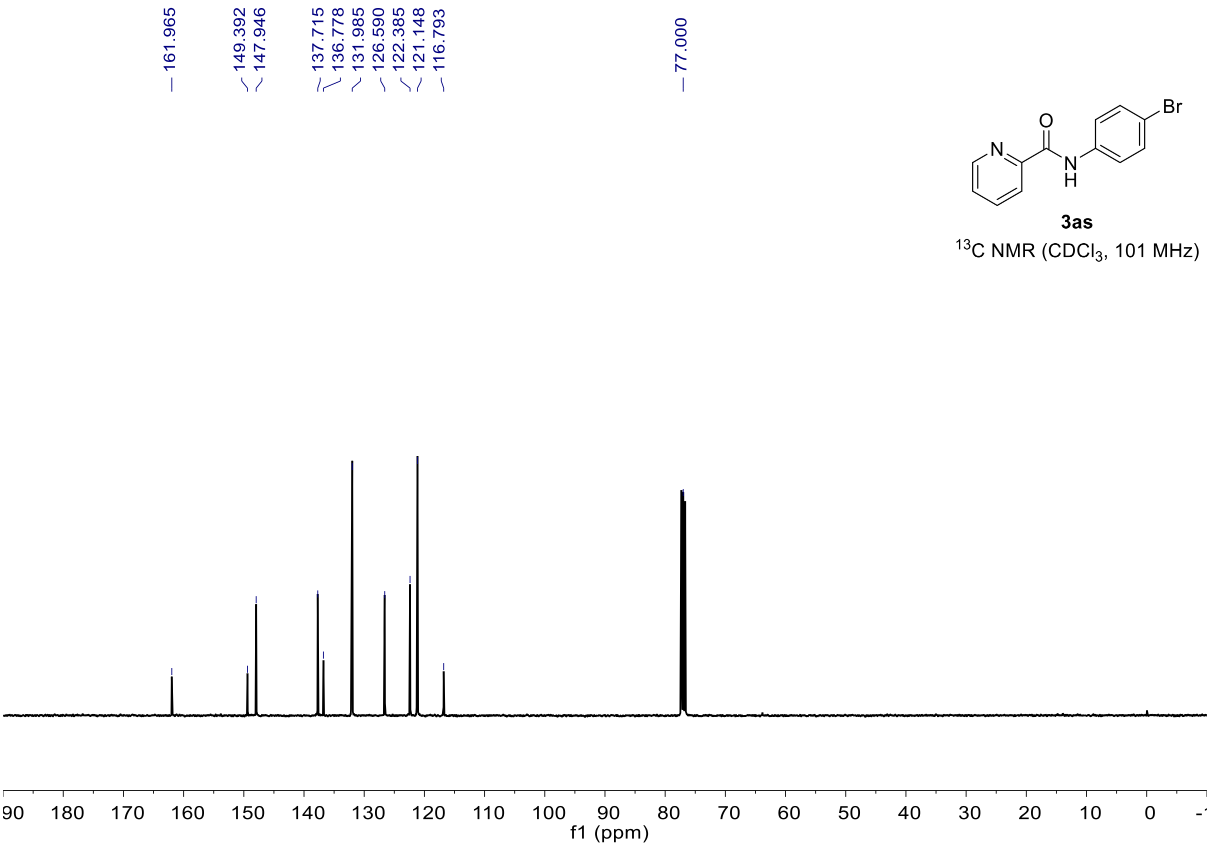

3as

^{13}C NMR ($CDCl_3$, 101 MHz)

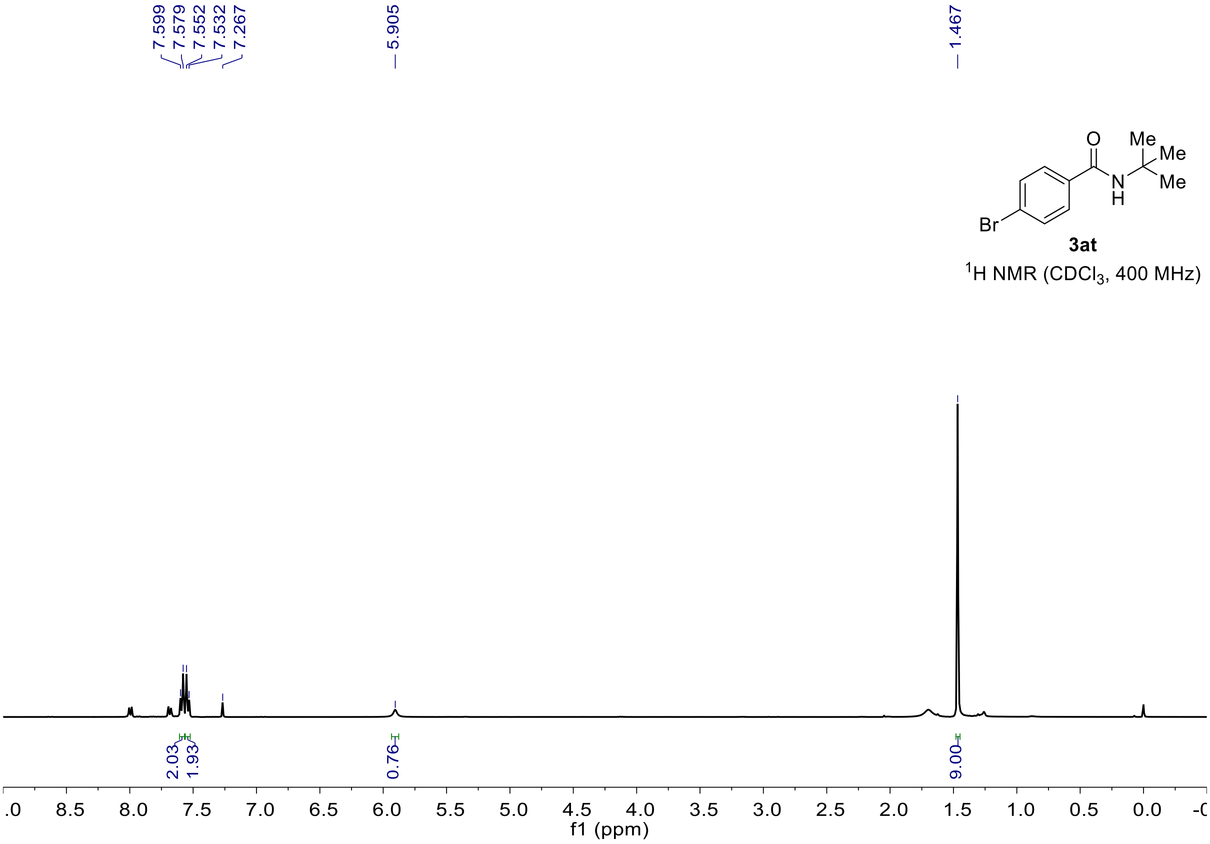

3at

^{1}H NMR ($CDCl_3$, 400 MHz)

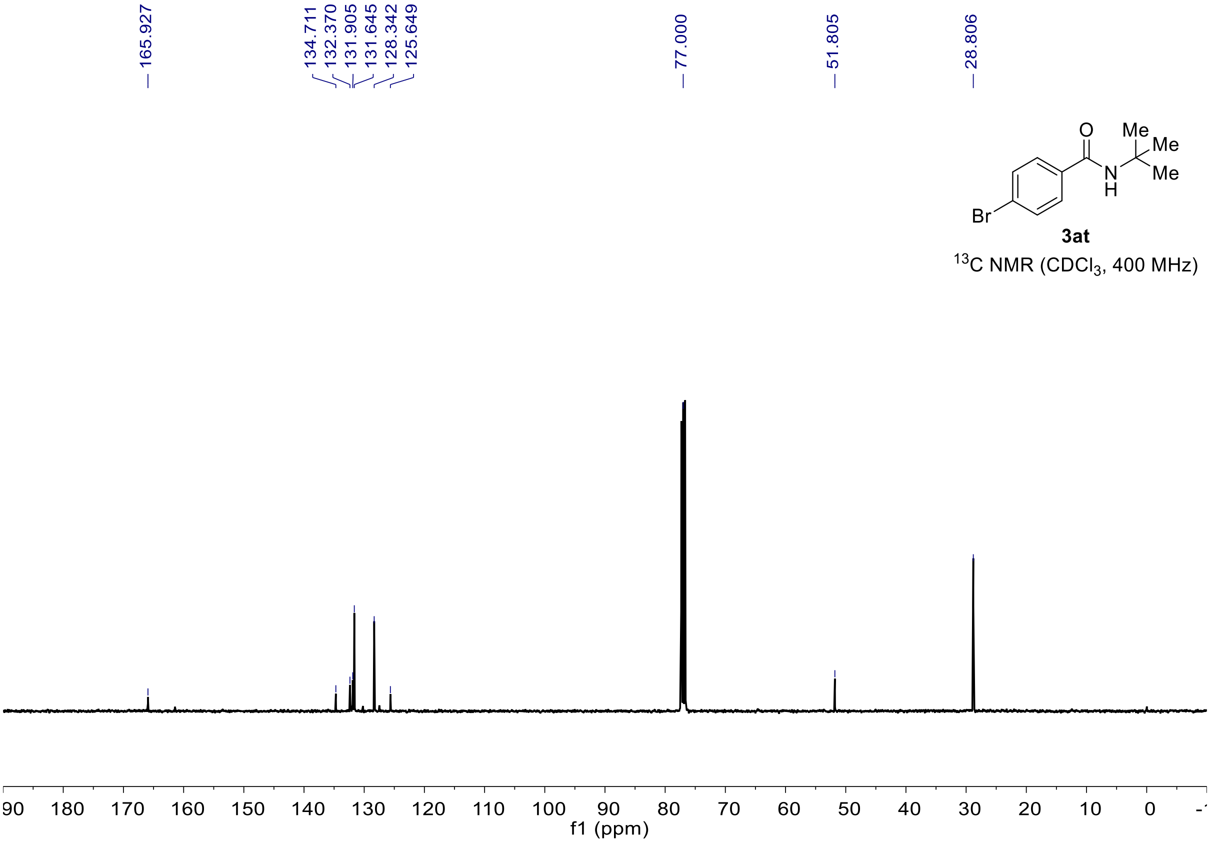

3at

^{13}C NMR ($CDCl_3$, 400 MHz)

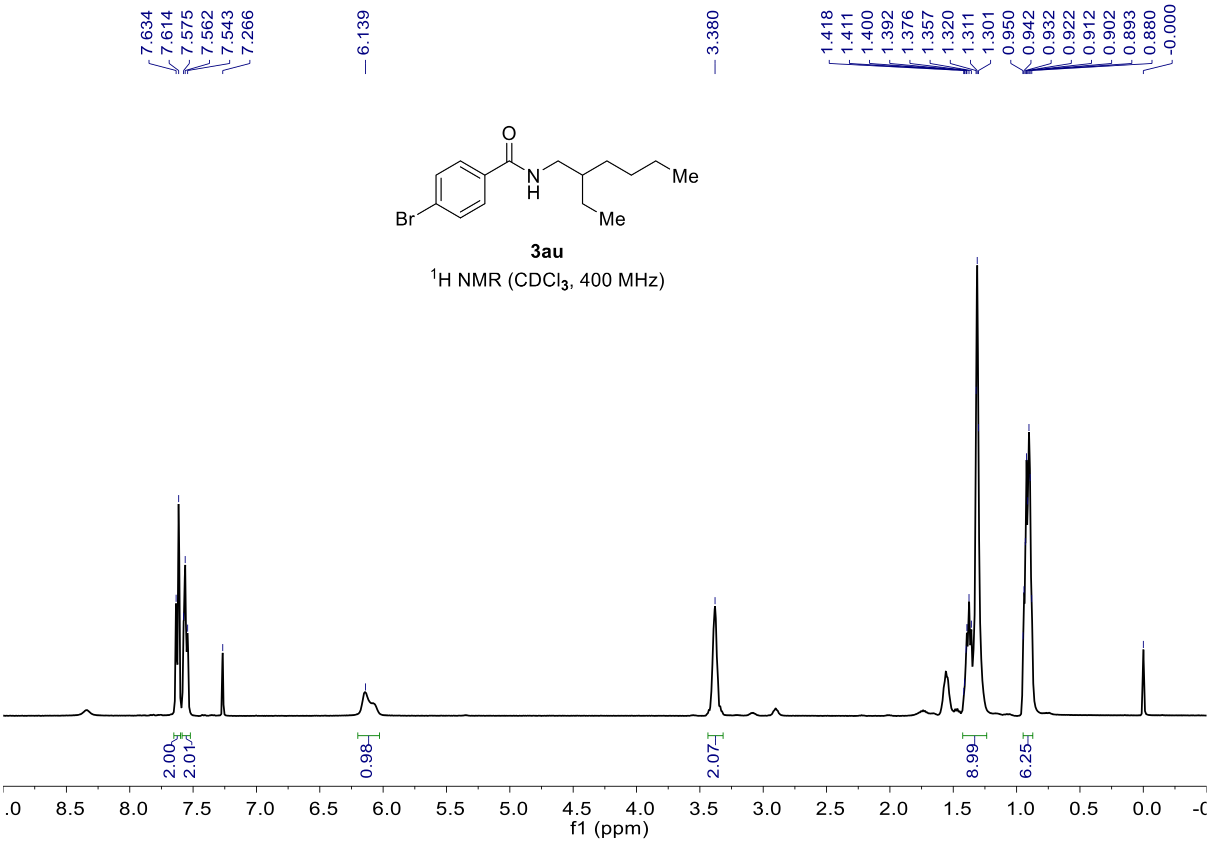
7.634
7.614
7.575
7.562
7.543
7.266
6.139
3.380
1.418
1.411
1.400
1.392
1.376
1.357
1.320
1.311
1.301
0.950
0.942
0.932
0.922
0.912
0.902
0.893
0.880
-0.000
O
N
H
Me
Br
Me
3au
1H NMR (CDCl3, 400 MHz)
2.00
2.01
0.98
2.07
8.99
6.25
8.5
8.0
7.5
7.0
6.5
6.0
5.5
5.0
4.5
4.0
3.5
3.0
2.5
2.0
1.5
1.0
0.5
0.0
f1 (ppm)

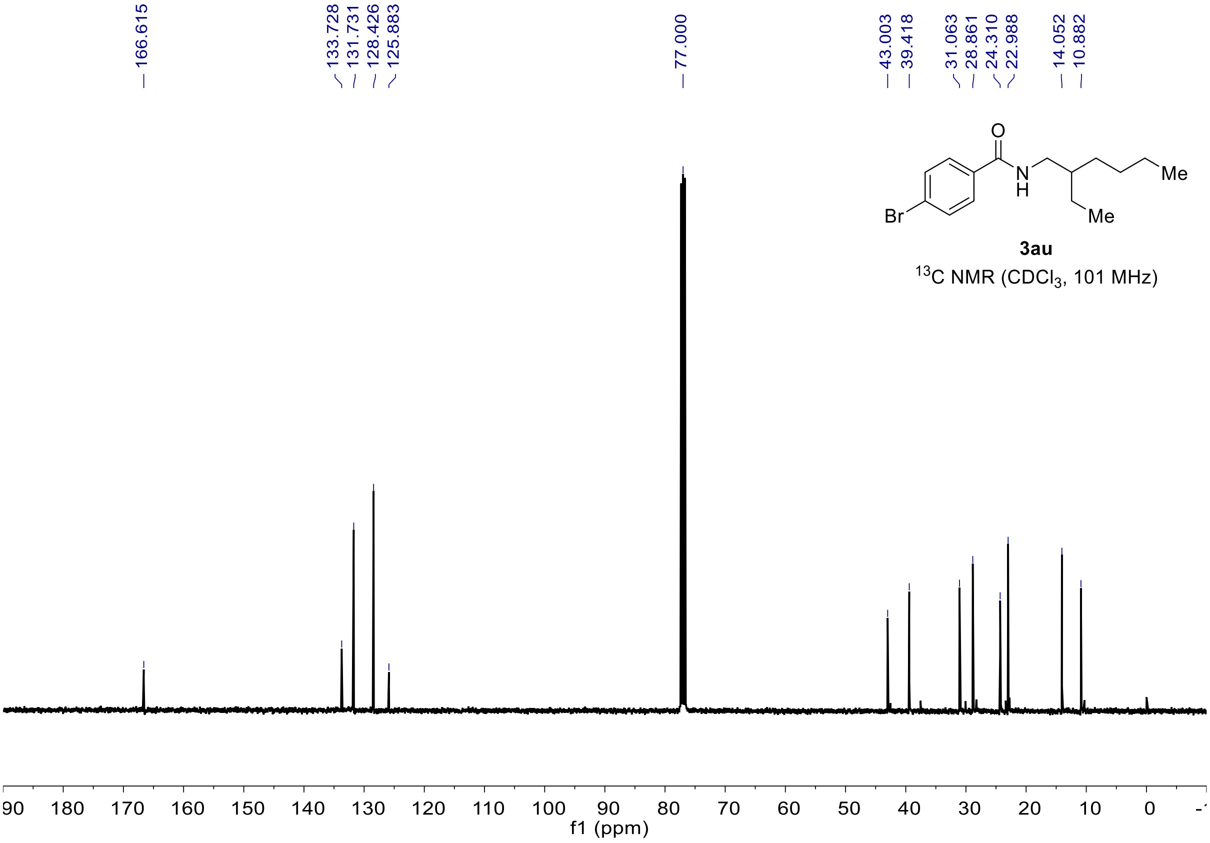
166.615
133.728
131.731
128.426
125.883
77.000
43.003
39.418
31.063
28.861
24.310
22.988
14.052
10.882
O
N
H
Me
Br
Me
3au
13C NMR (CDCl3, 101 MHz)
90
180
170
160
150
140
130
120
110
100
90
80
70
60
50
40
30
20
10
0
f1 (ppm)

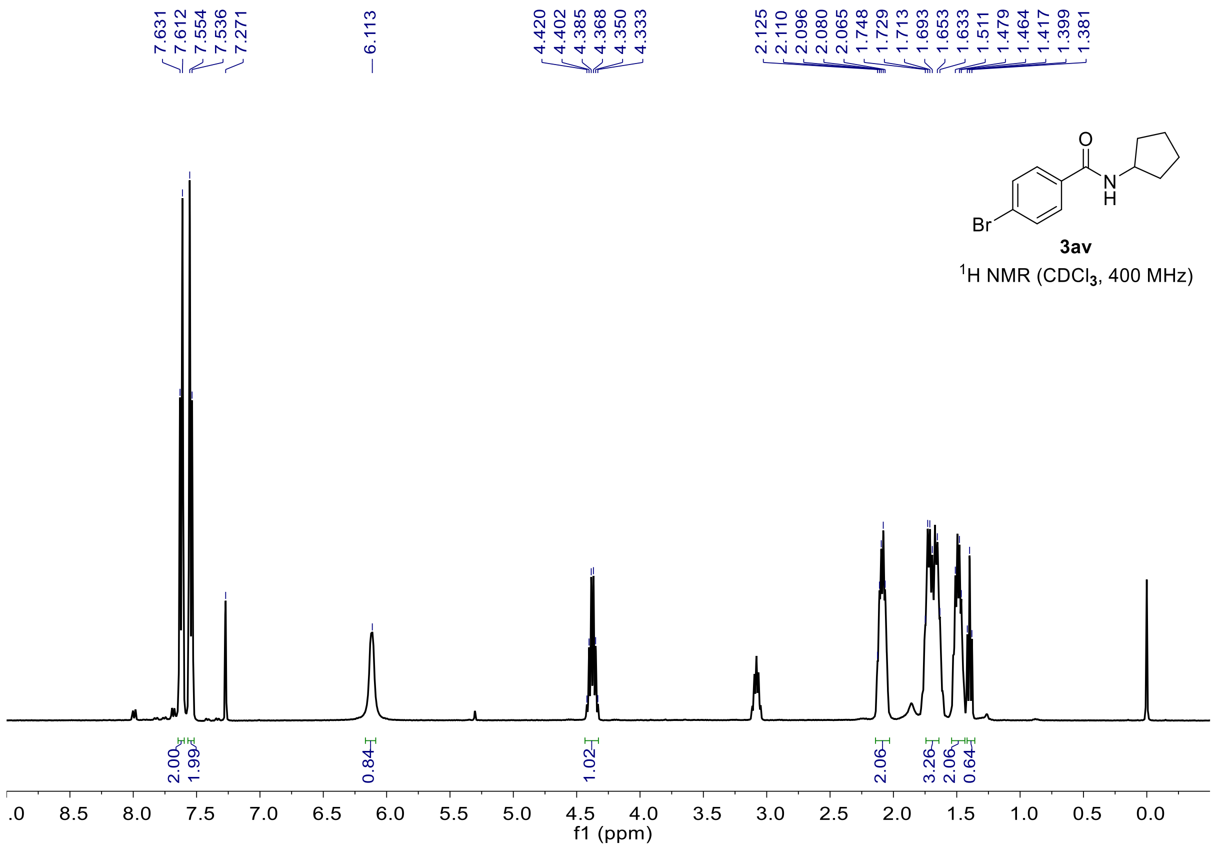
7.631
7.612
7.554
7.536
7.271
6.113
4.420
4.402
4.385
4.368
4.350
4.333
2.125
2.110
2.096
2.080
2.065
1.748
1.729
1.713
1.693
1.653
1.633
1.511
1.479
1.464
1.417
1.399
1.381
O
N
H
Br
3av
1H NMR (CDCl3, 400 MHz)
2.00
1.99
0.84
1.02
2.06
3.26
2.06
0.64
f1 (ppm)

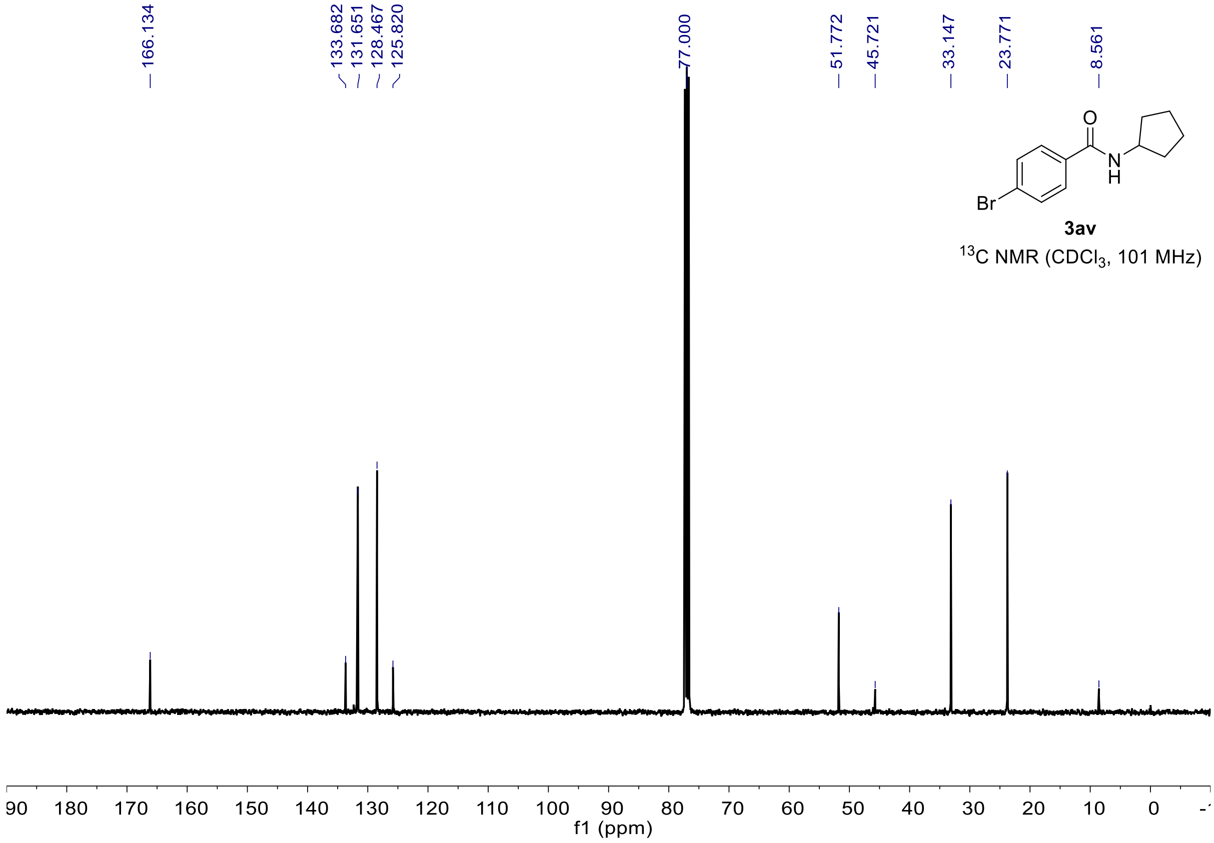
166.134
133.682
131.651
128.467
125.820
77.000
51.772
45.721
33.147
23.771
8.561
O
N
H
Br
3av
13C NMR (CDCl3, 101 MHz)
f1 (ppm)

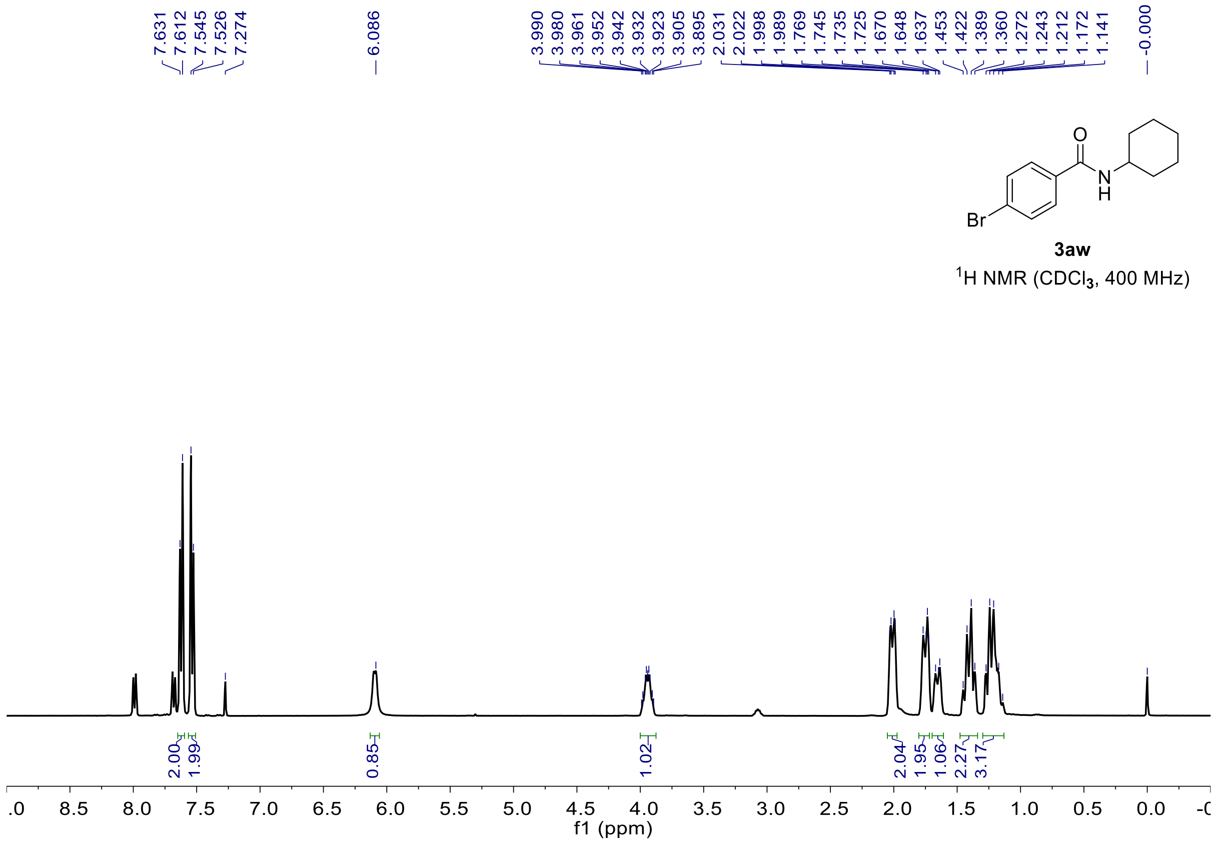

3aw
^{1}H NMR (CDCl$_3$, 400 MHz)
f1 (ppm)

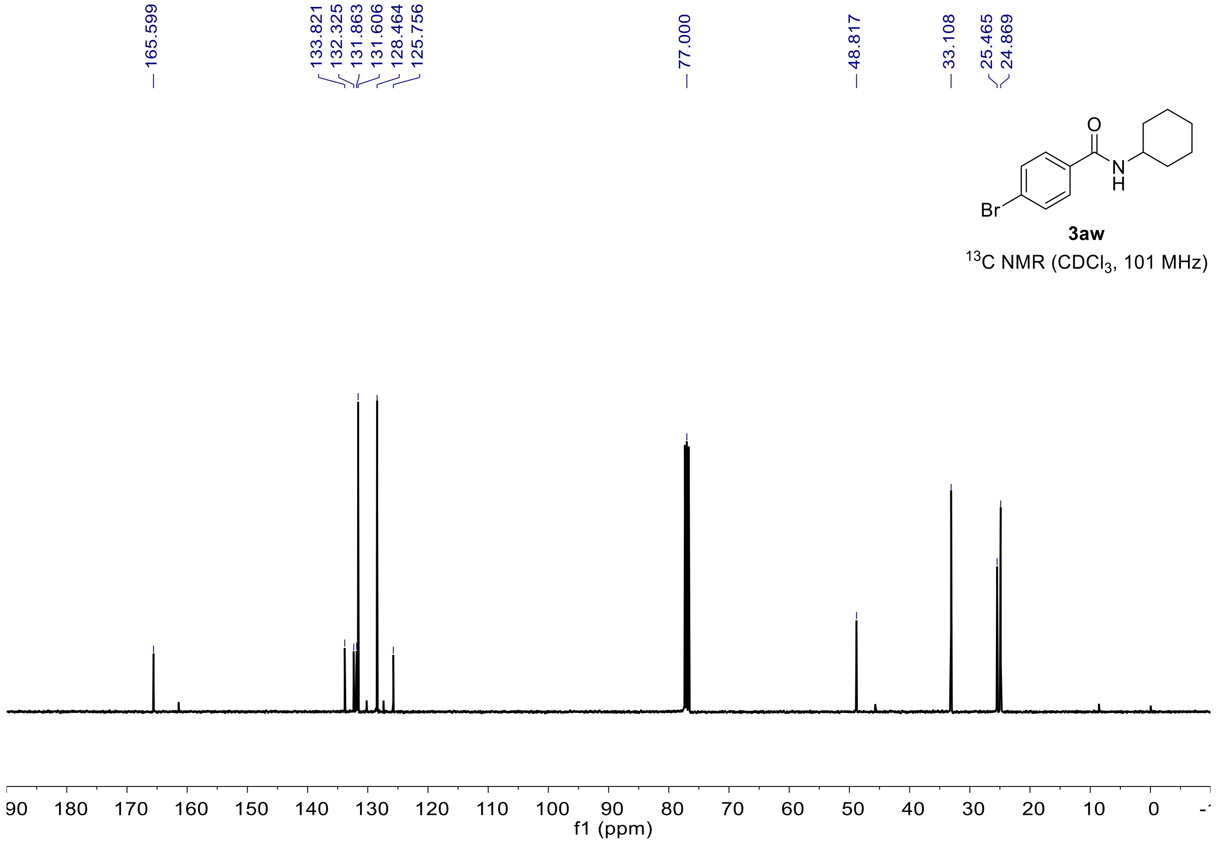

3aw
^{13}C NMR (CDCl$_3$, 101 MHz)
f1 (ppm)

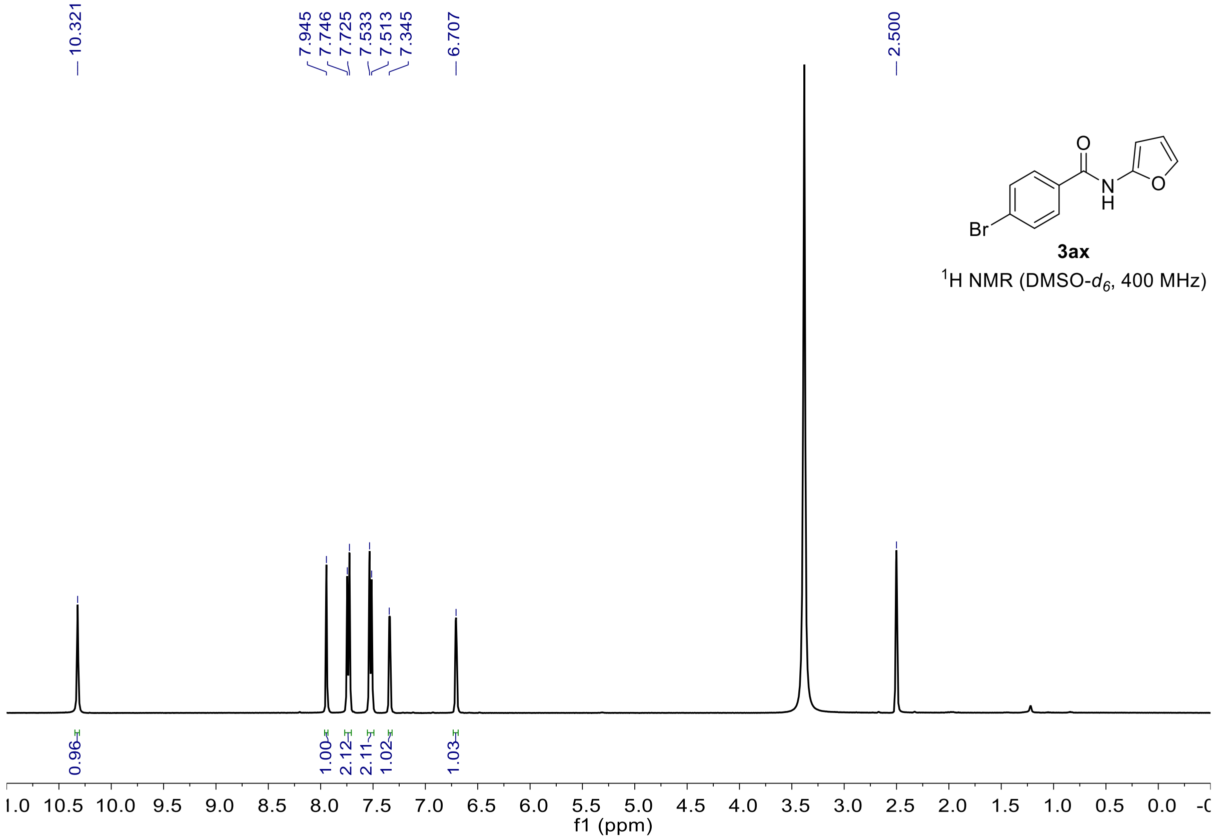

10.321
7.945
7.746
7.725
7.533
7.513
7.345
6.707
2.500
0.96
1.00
2.12
2.11
1.02
1.03
f1 (ppm)
O
N
H
O
Br
3ax
^{1}H NMR (DMSO-d_6, 400 MHz)

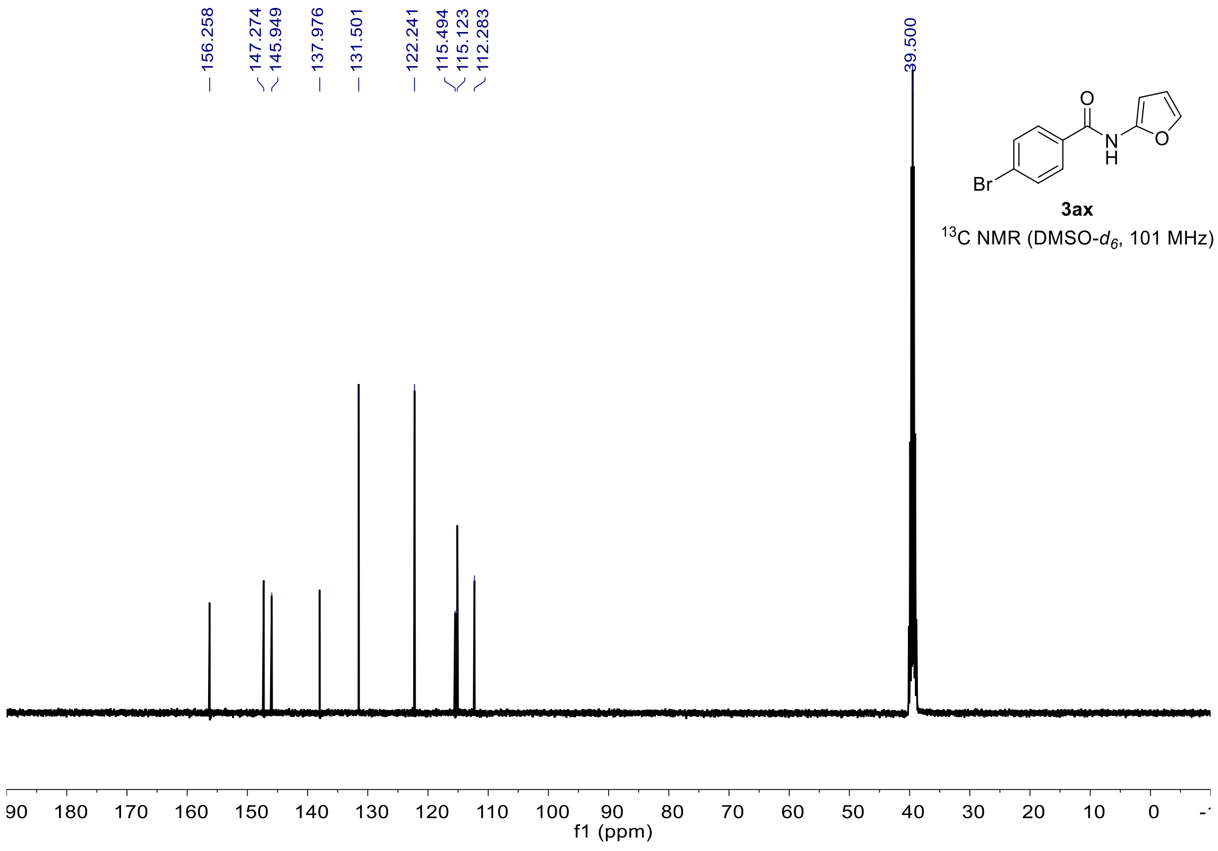

156.258
147.274
145.949
137.976
131.501
122.241
115.494
115.123
112.283
39.500
f1 (ppm)
O
N
H
O
Br
3ax
^{13}C NMR (DMSO-d_6, 101 MHz)

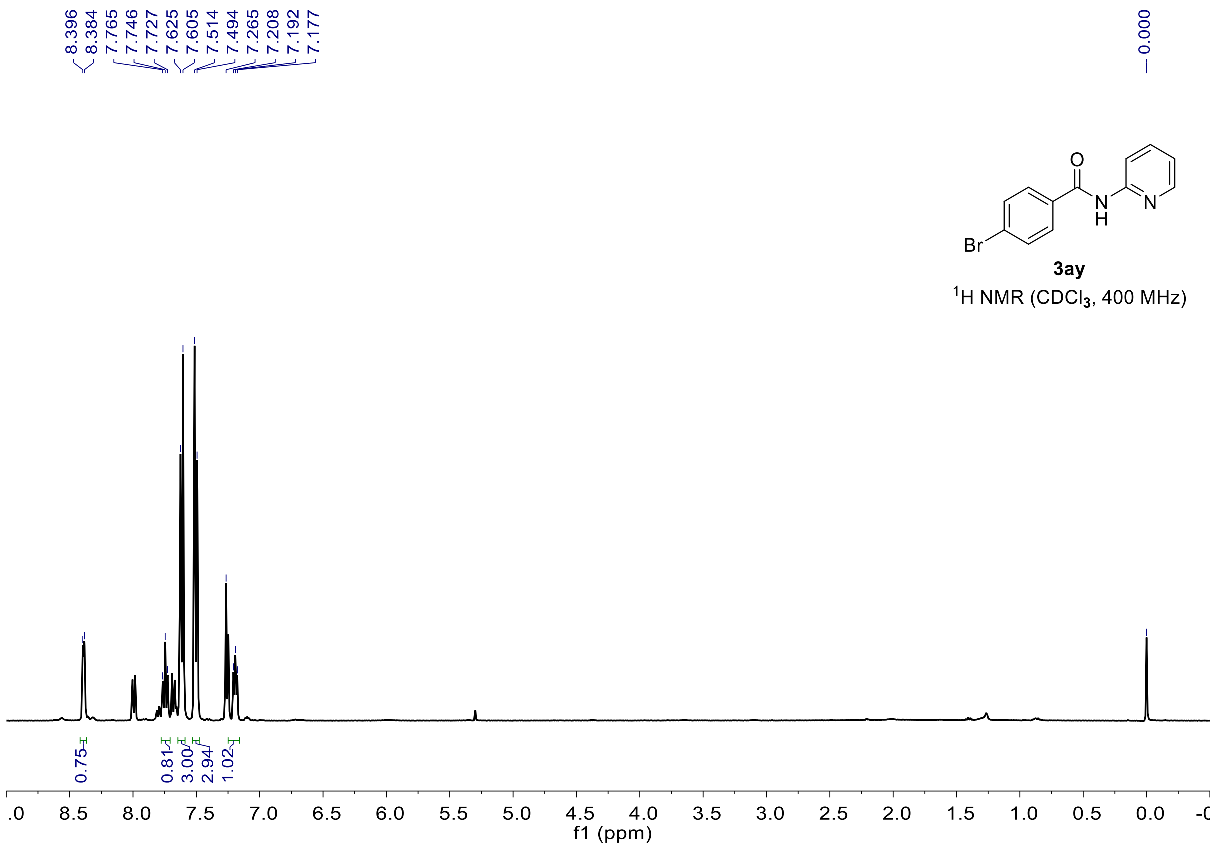

3ay

^{1}H NMR ($CDCl_3$, 400 MHz)

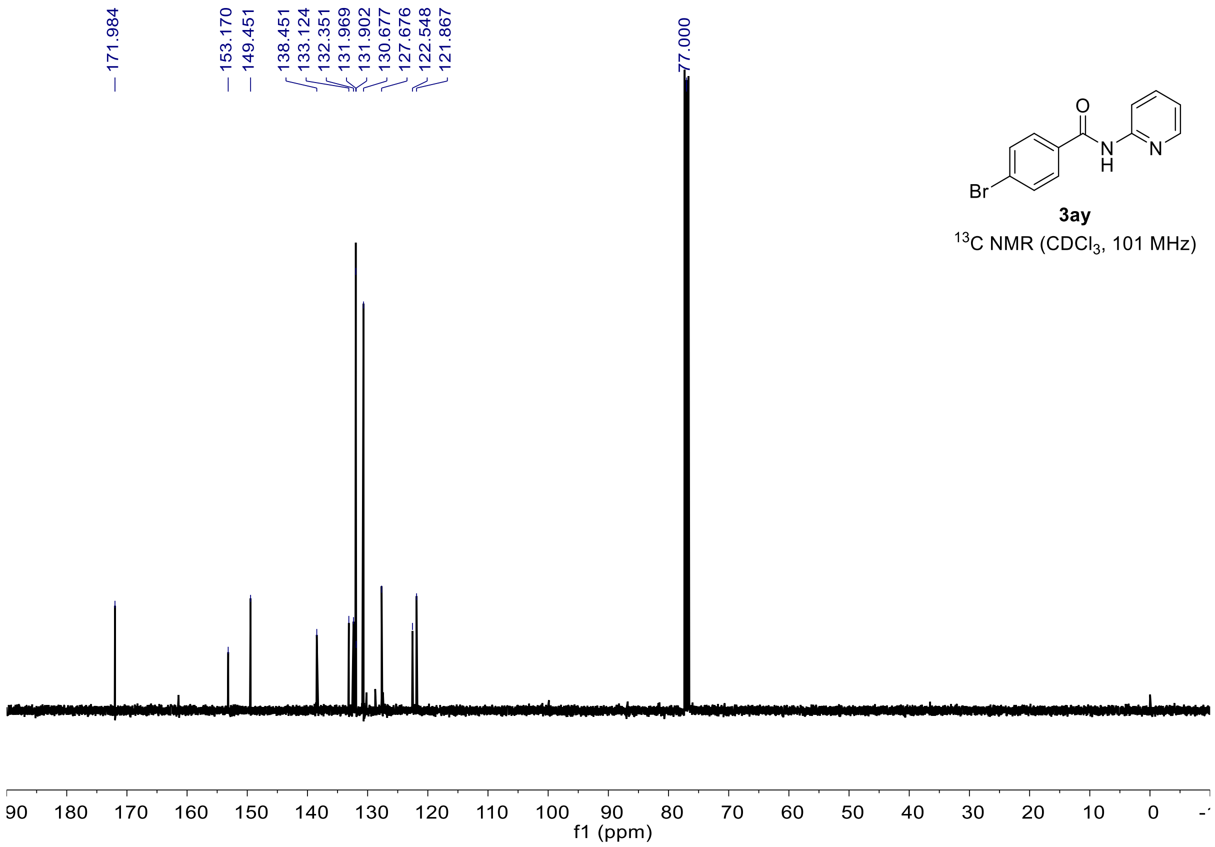

3ay

^{13}C NMR ($CDCl_3$, 101 MHz)

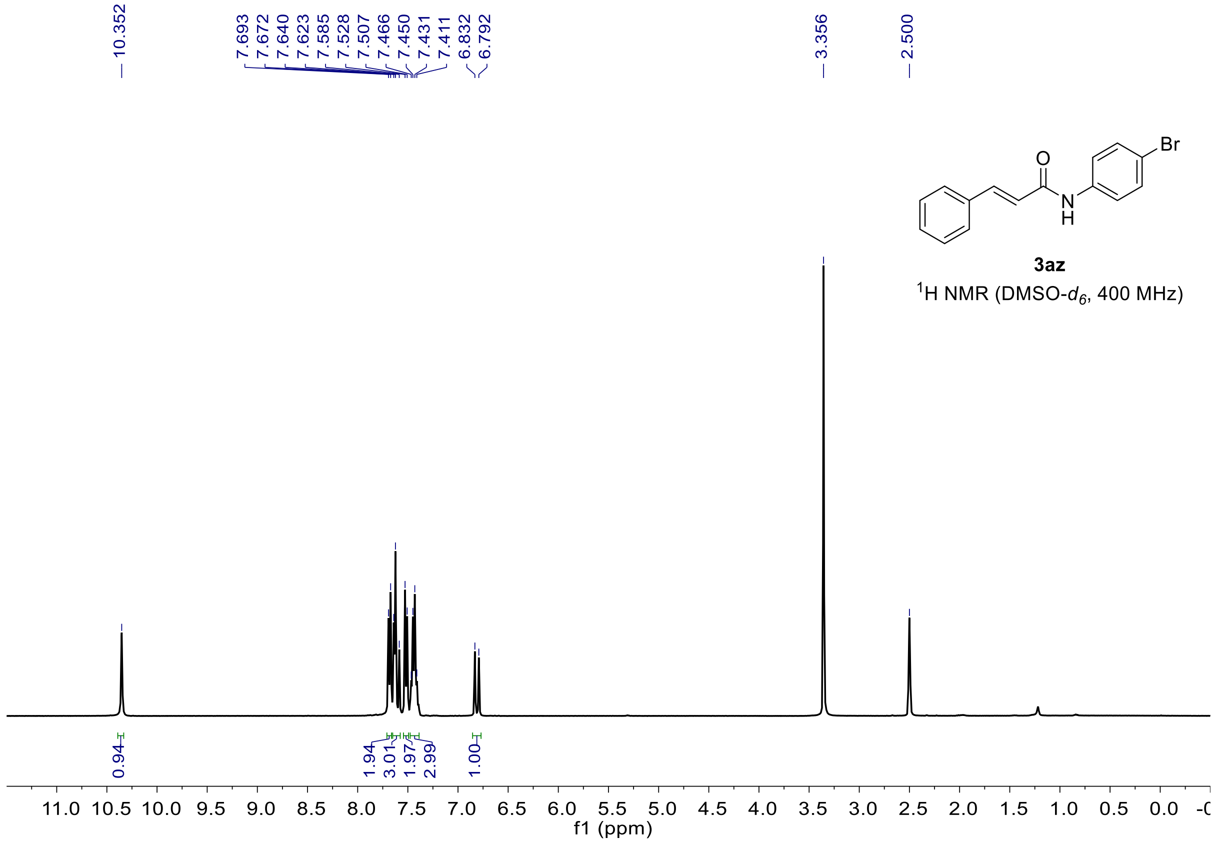
10.352
7.693
7.672
7.640
7.623
7.585
7.528
7.507
7.466
7.450
7.431
7.411
6.832
6.792
3.356
2.500
Br
O
N
H
3az
^{1}H NMR (DMSO-d_6, 400 MHz)
0.94
1.94
3.01
1.97
2.99
1.00
11.0 10.5 10.0 9.5 9.0 8.5 8.0 7.5 7.0 6.5 6.0 5.5 5.0 4.5 4.0 3.5 3.0 2.5 2.0 1.5 1.0 0.5 0.0
f1 (ppm)

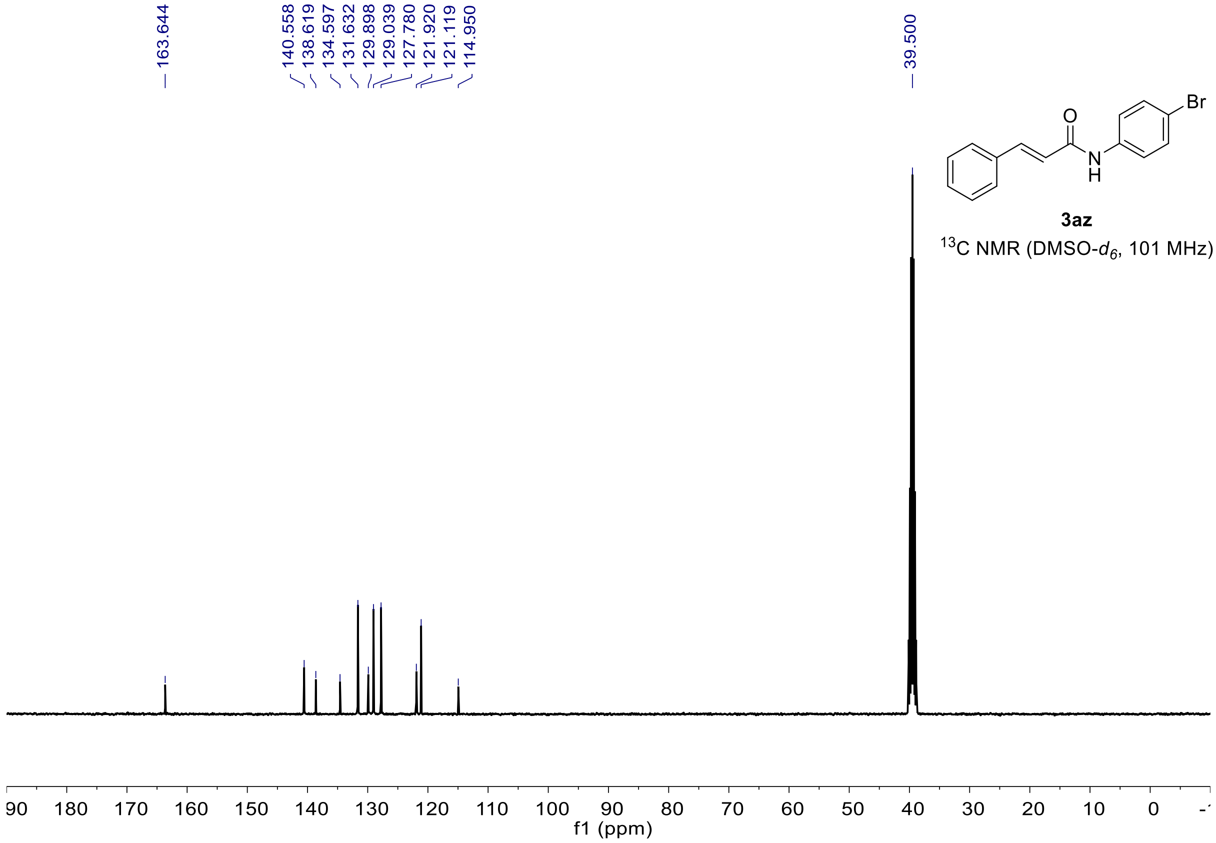
163.644
140.558
138.619
134.597
131.632
129.898
129.039
127.780
121.920
121.119
114.950
39.500
Br
O
N
H
3az
^{13}C NMR (DMSO-d_6, 101 MHz)
180 170 160 150 140 130 120 110 100 90 80 70 60 50 40 30 20 10 0
f1 (ppm)

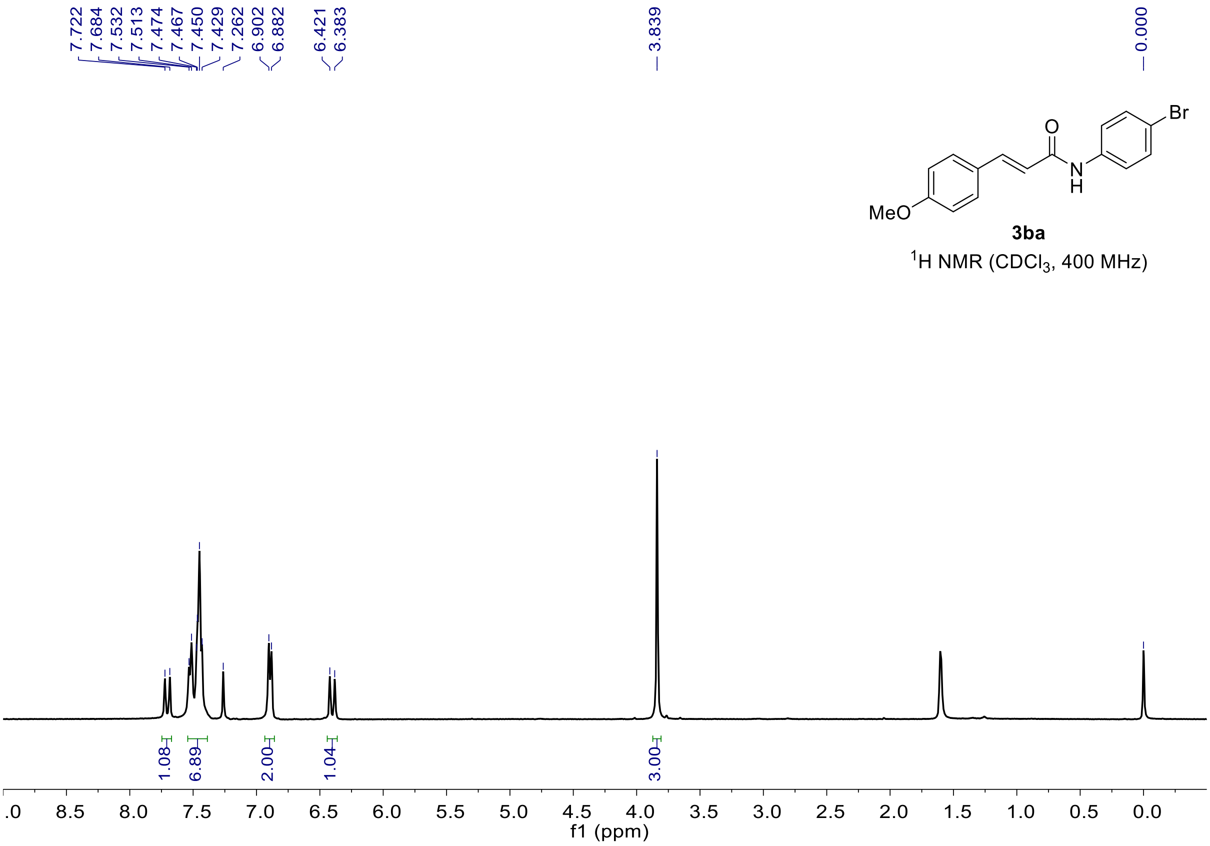

3ba

^1H NMR ($CDCl_3$, 400 MHz)

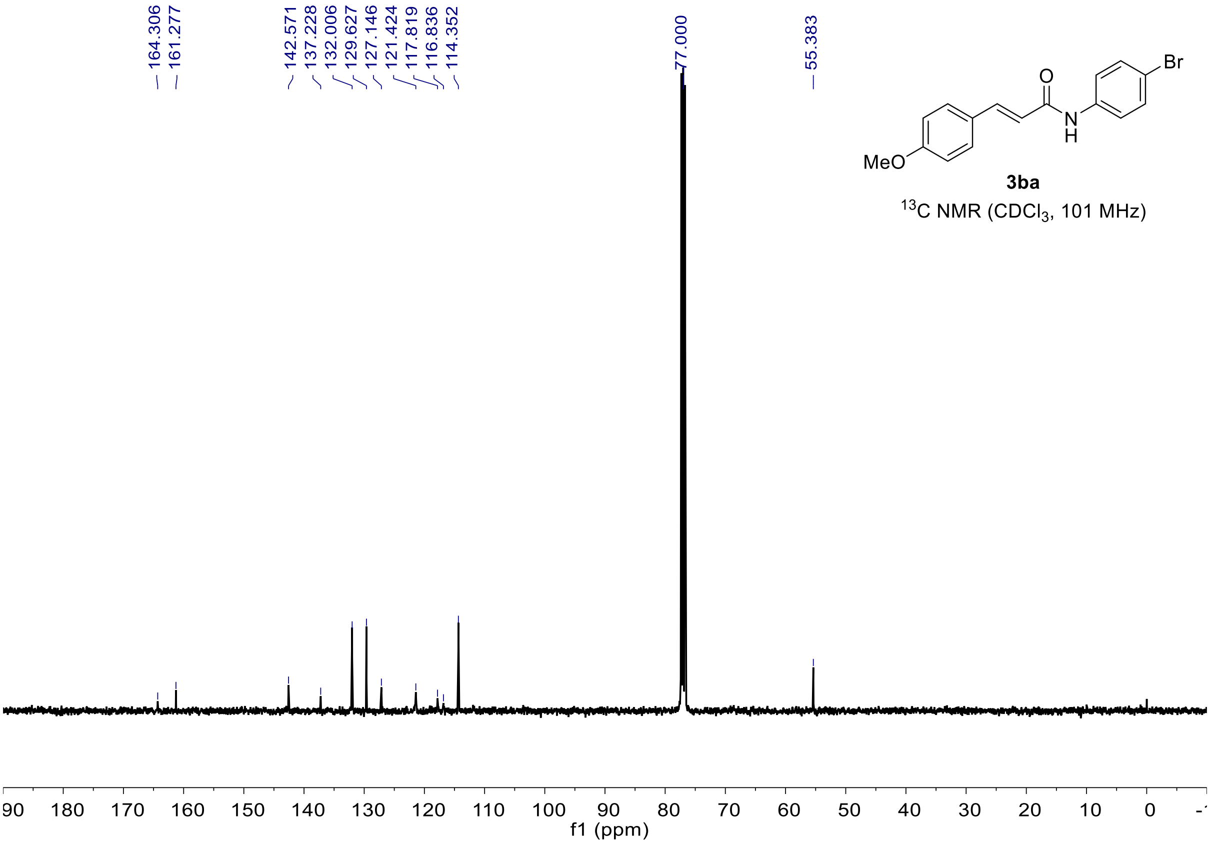

3ba

^{13}C NMR ($CDCl_3$, 101 MHz)

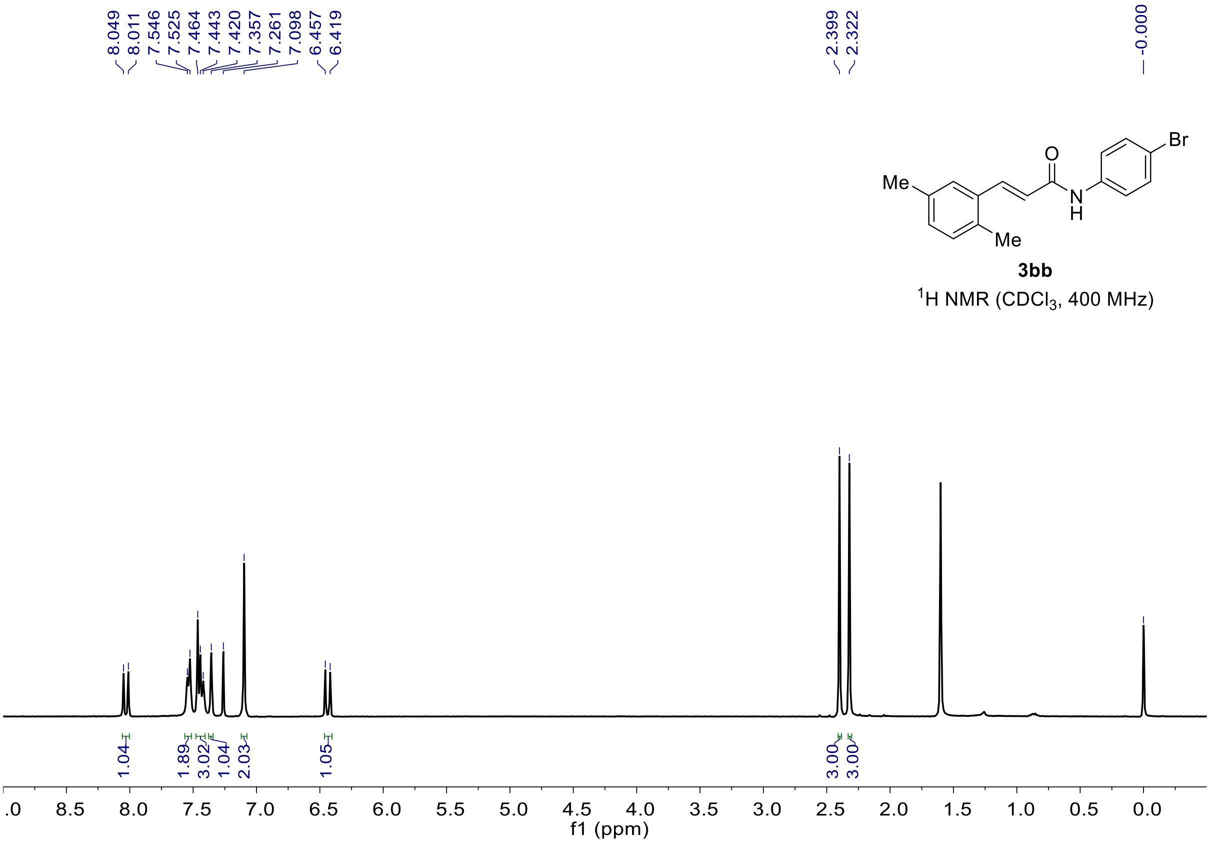
8.049
8.011
7.546
7.525
7.464
7.443
7.420
7.357
7.261
7.098
6.457
6.419
2.399
2.322
-0.000
Me
O
N
H
Br
Me
3bb
1H NMR (CDCl3, 400 MHz)
1.04
1.89
3.02
1.04
2.03
1.05
3.00
3.00
f1 (ppm)

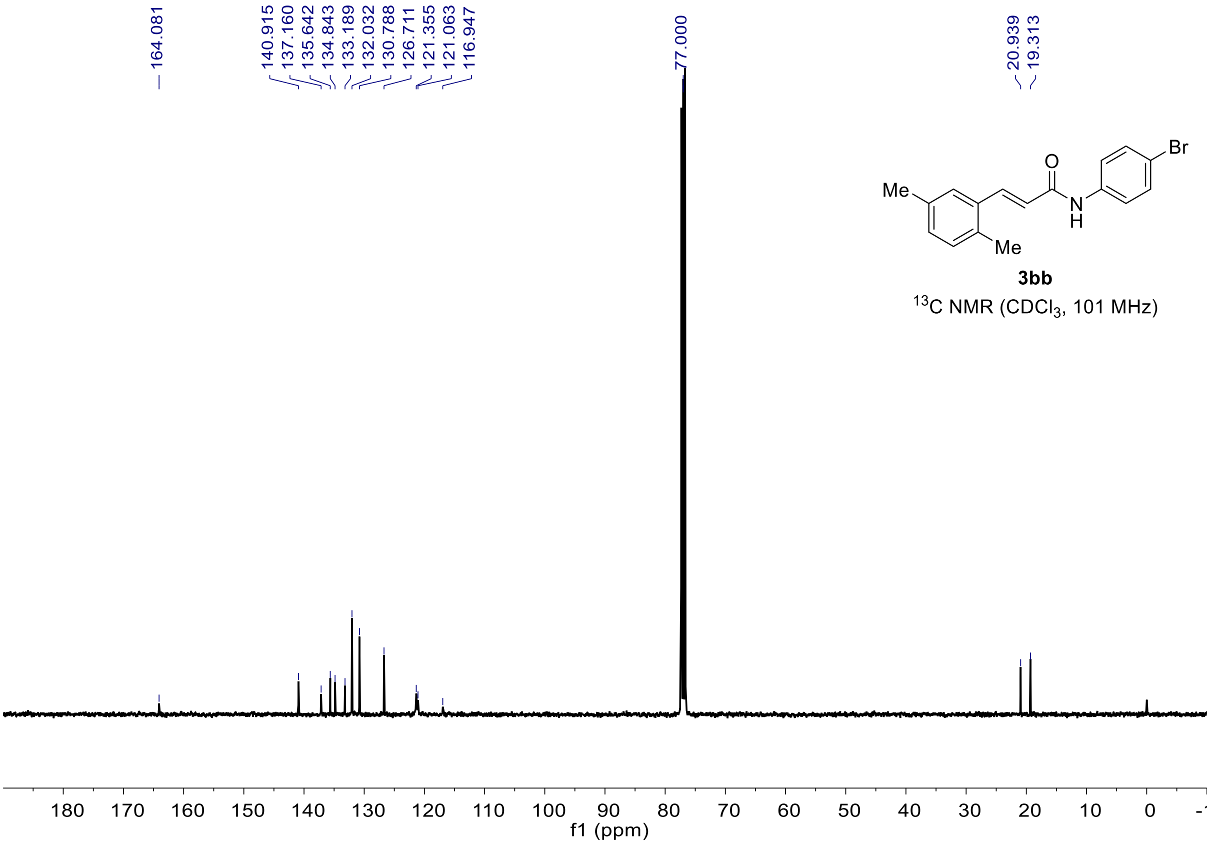
164.081
140.915
137.160
135.642
134.843
133.189
132.032
130.788
126.711
121.355
121.063
116.947
77.000
20.939
19.313
Me
O
N
H
Br
Me
3bb
13C NMR (CDCl3, 101 MHz)
f1 (ppm)

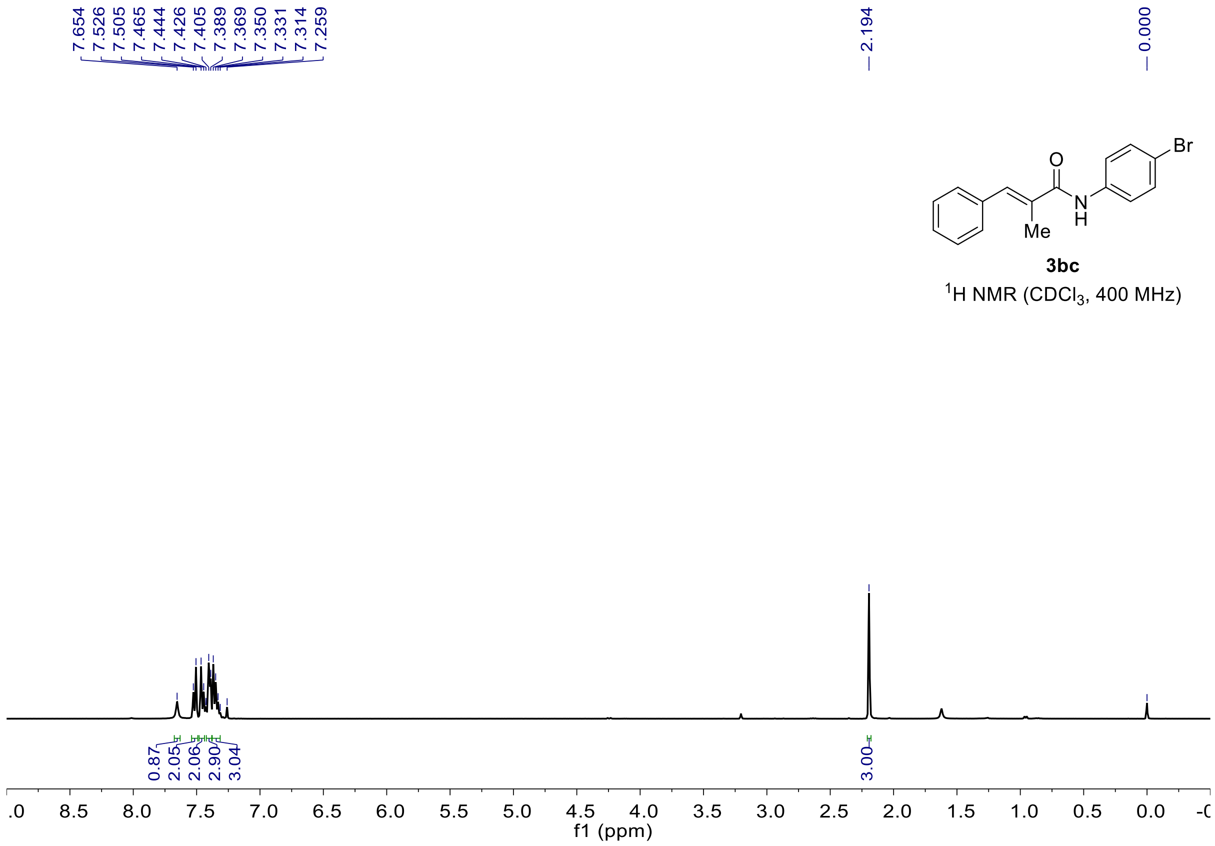
7.654
7.526
7.505
7.465
7.444
7.426
7.405
7.389
7.369
7.350
7.331
7.314
7.259
2.194
0.000
Br
O
N
H
Me
3bc
^{1}H NMR (CDCl$_3$, 400 MHz)
0.87
2.05
2.06
2.90
3.04
3.00
f1 (ppm)

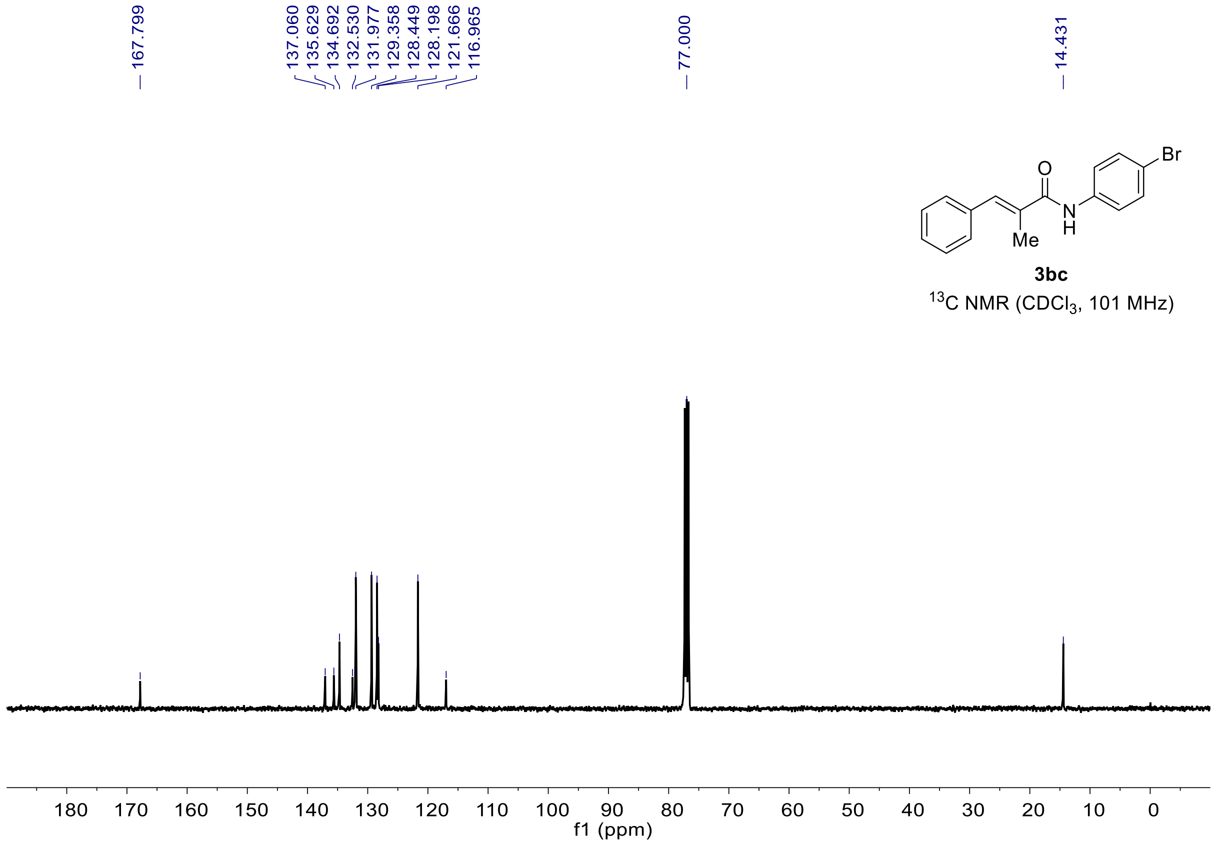
167.799
137.060
135.629
134.692
132.530
131.977
129.358
128.449
128.198
121.666
116.965
77.000
14.431
Br
O
N
H
Me
3bc
^{13}C NMR (CDCl$_3$, 101 MHz)
f1 (ppm)

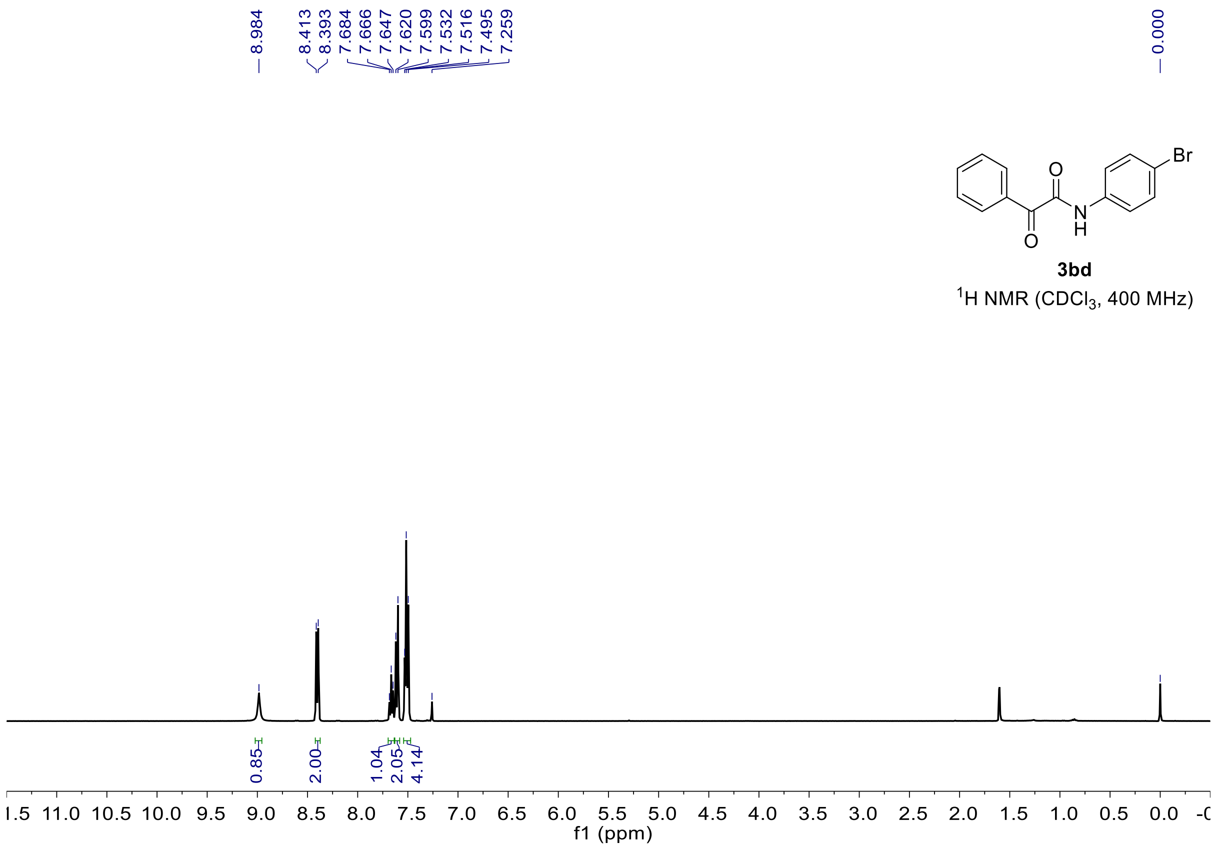
8.984
8.413
8.393
7.684
7.666
7.647
7.620
7.599
7.532
7.516
7.495
7.259
0.000
Br
O
N
H
O
3bd
^{1}H NMR ($CDCl_3$, 400 MHz)
0.85
2.00
1.04
2.05
4.14
f1 (ppm)

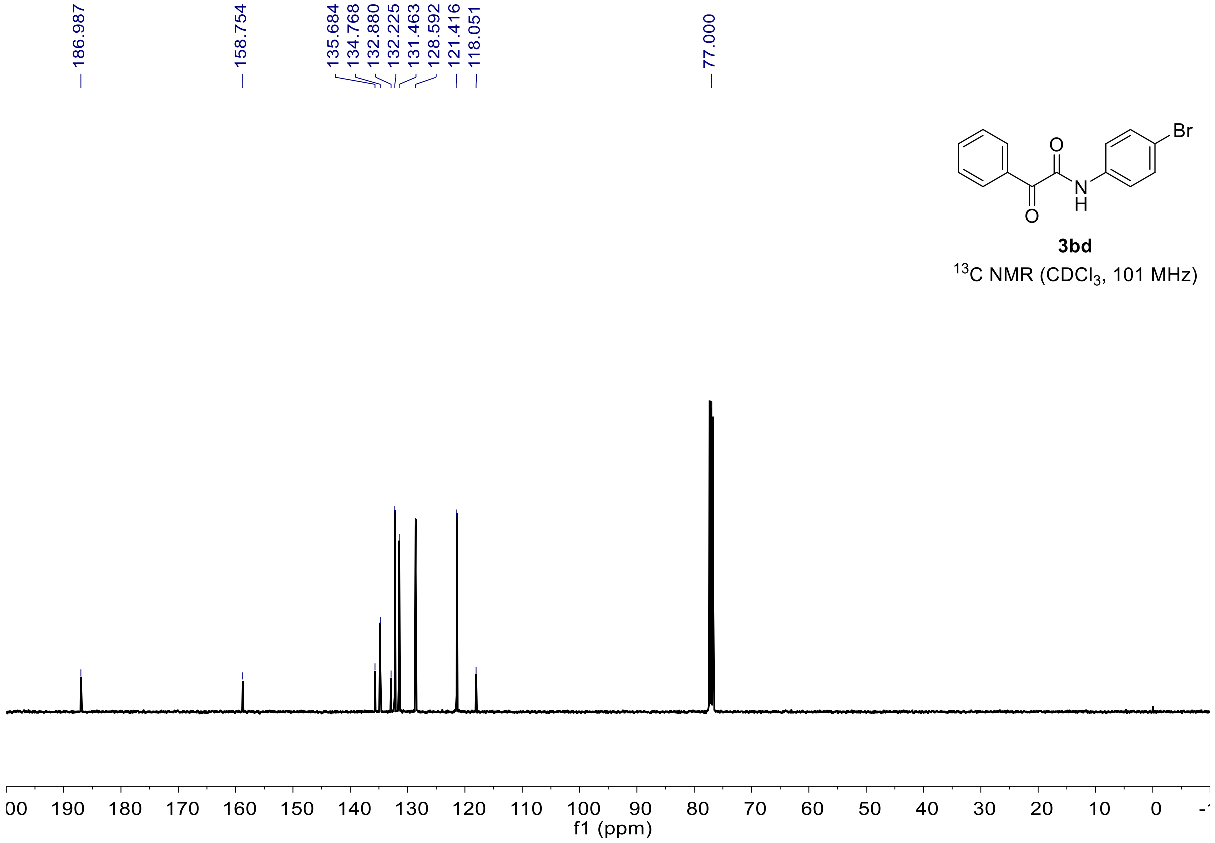
186.987
158.754
135.684
134.768
132.880
132.225
131.463
128.592
121.416
118.051
77.000
Br
O
N
H
O
3bd
^{13}C NMR ($CDCl_3$, 101 MHz)
f1 (ppm)

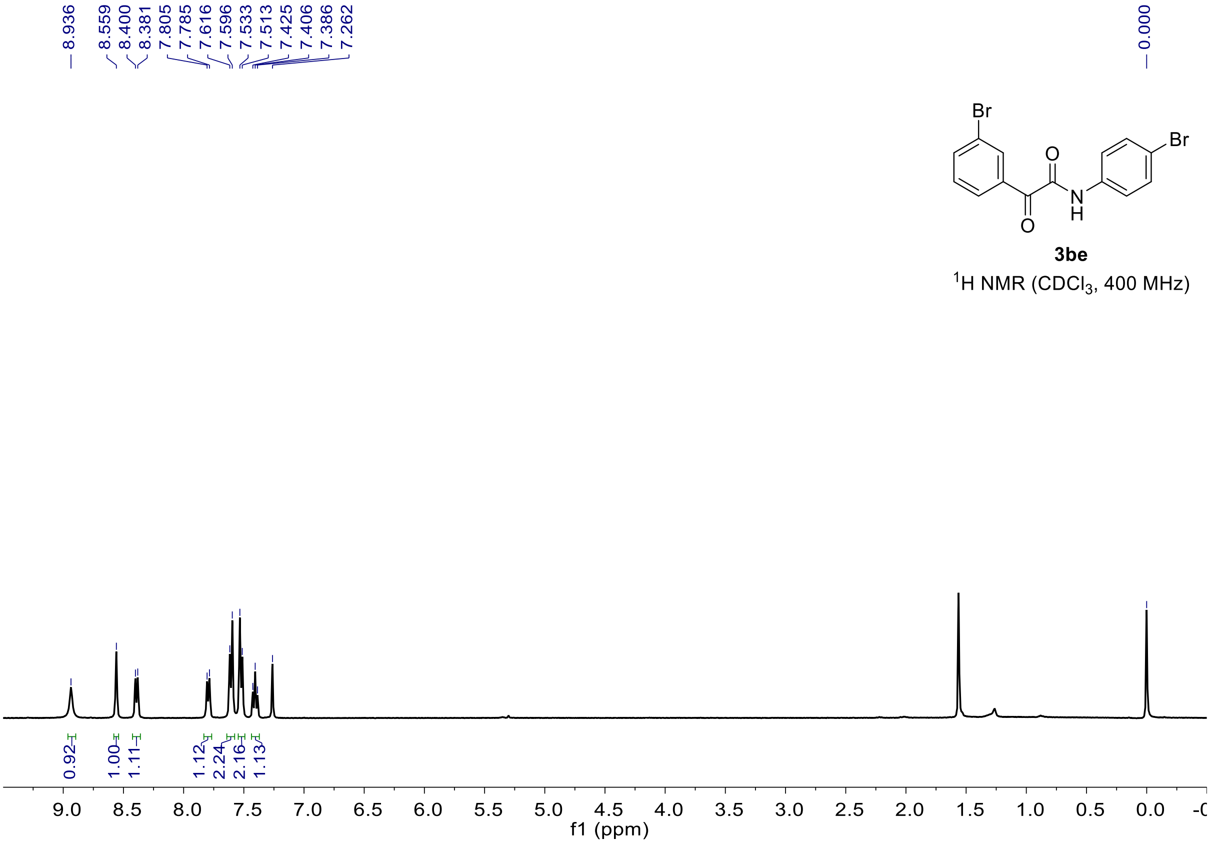

3be

^{1}H NMR ($CDCl_3$, 400 MHz)

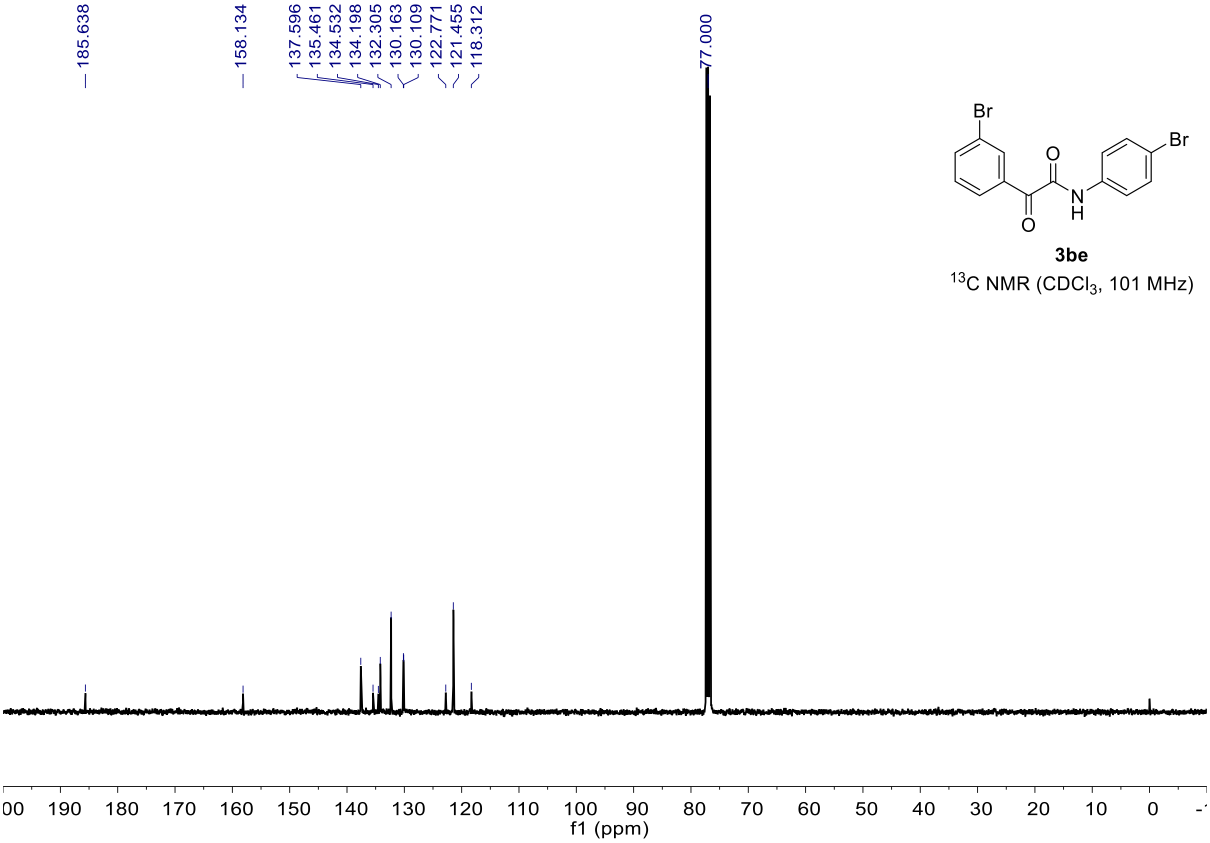

3be

^{13}C NMR ($CDCl_3$, 101 MHz)

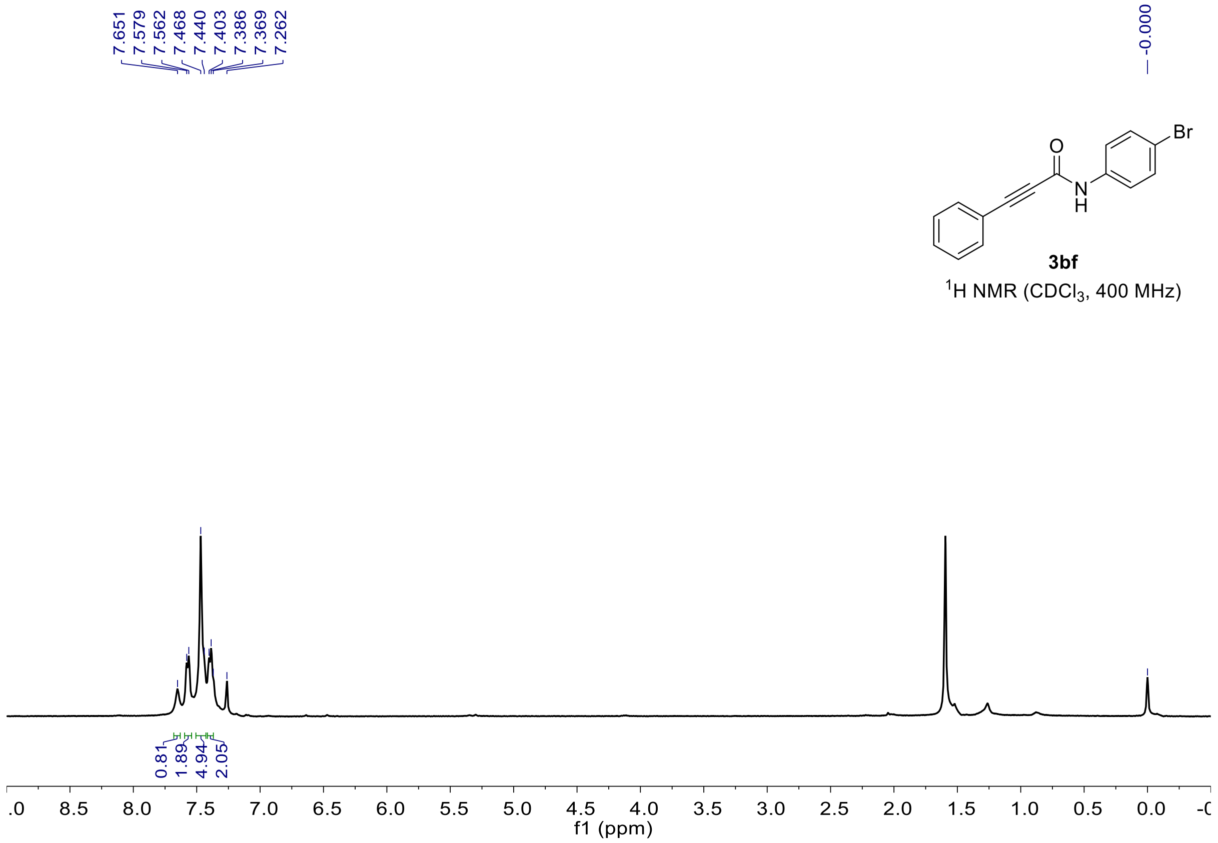
7.651
7.579
7.562
7.468
7.440
7.403
7.386
7.369
7.262
-0.000
O
N
H
Br
3bf
1H NMR (CDCl3, 400 MHz)
0.81
1.89
4.94
2.05
f1 (ppm)

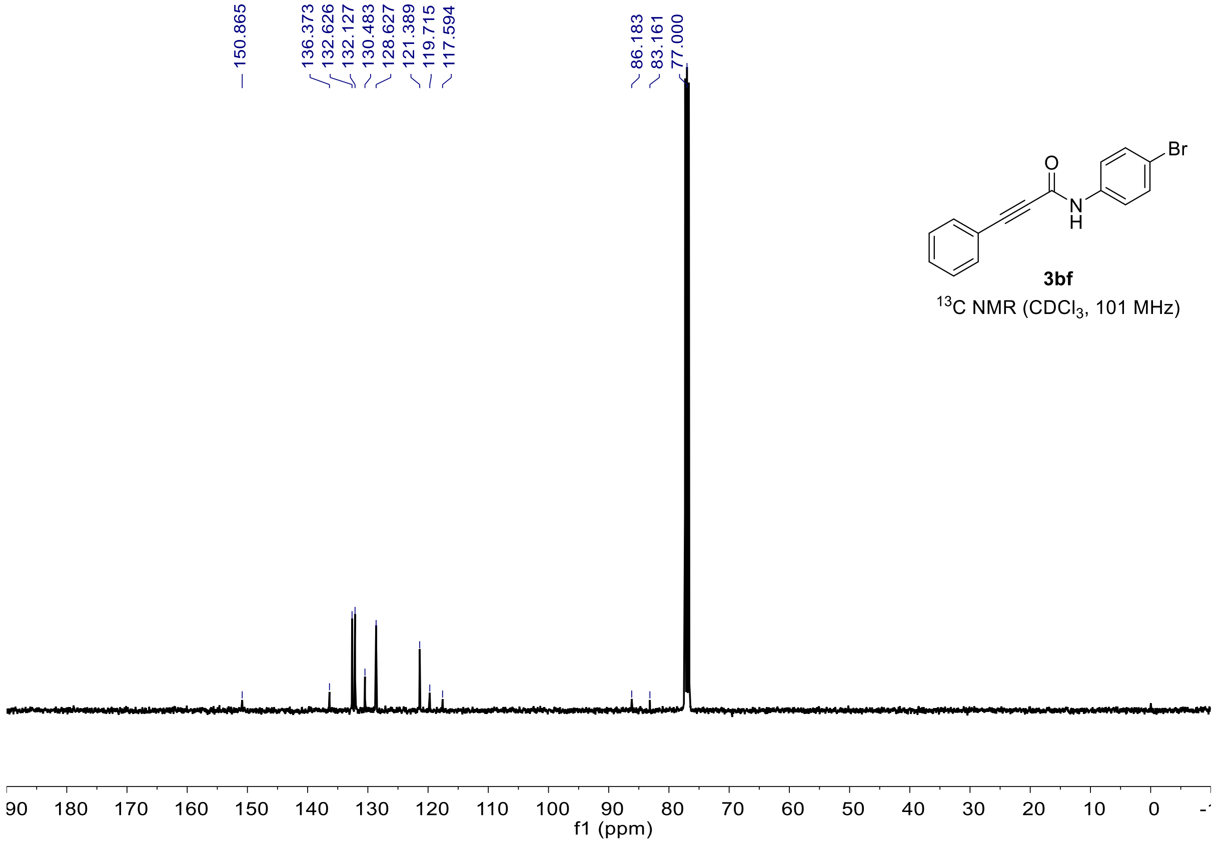
150.865
136.373
132.626
132.127
130.483
128.627
121.389
119.715
117.594
86.183
83.161
77.000
O
N
H
Br
3bf
13C NMR (CDCl3, 101 MHz)
f1 (ppm)

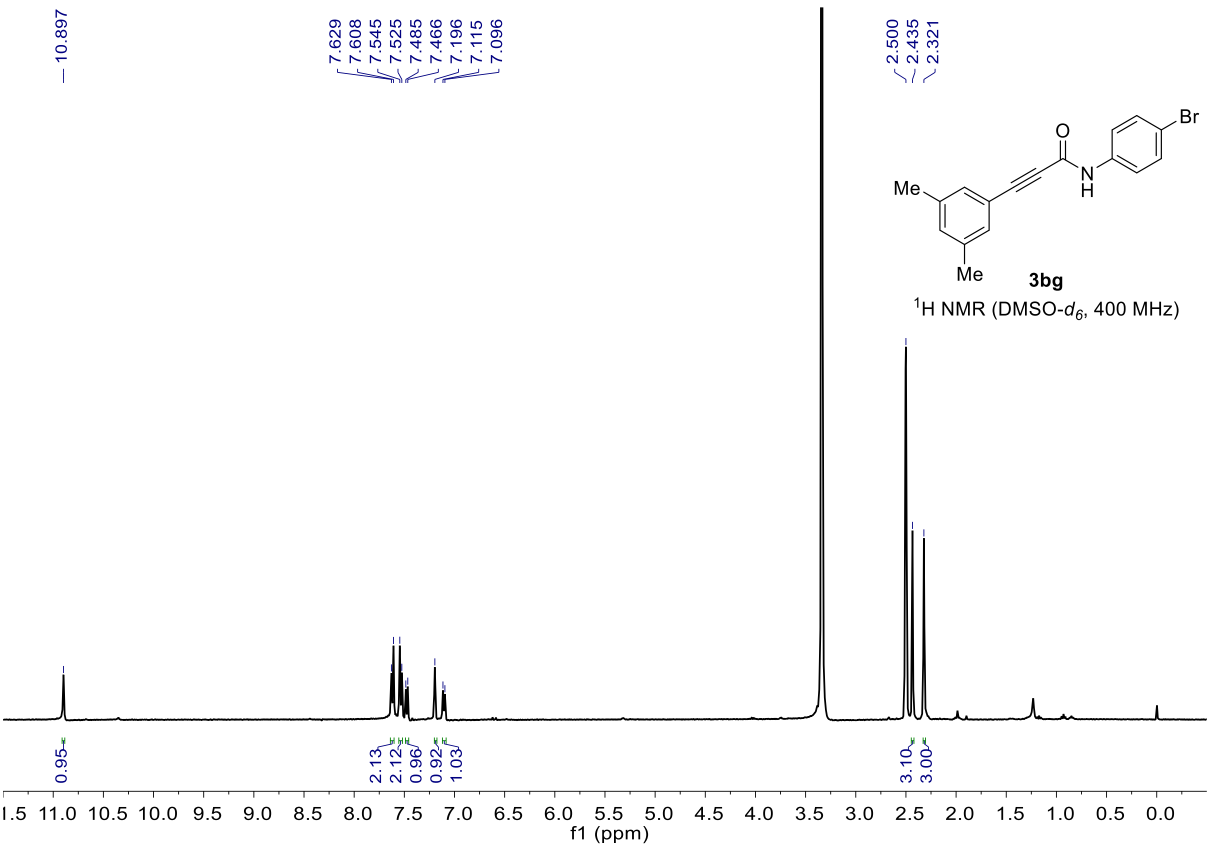

^{1}H NMR (DMSO-d_6, 400 MHz)

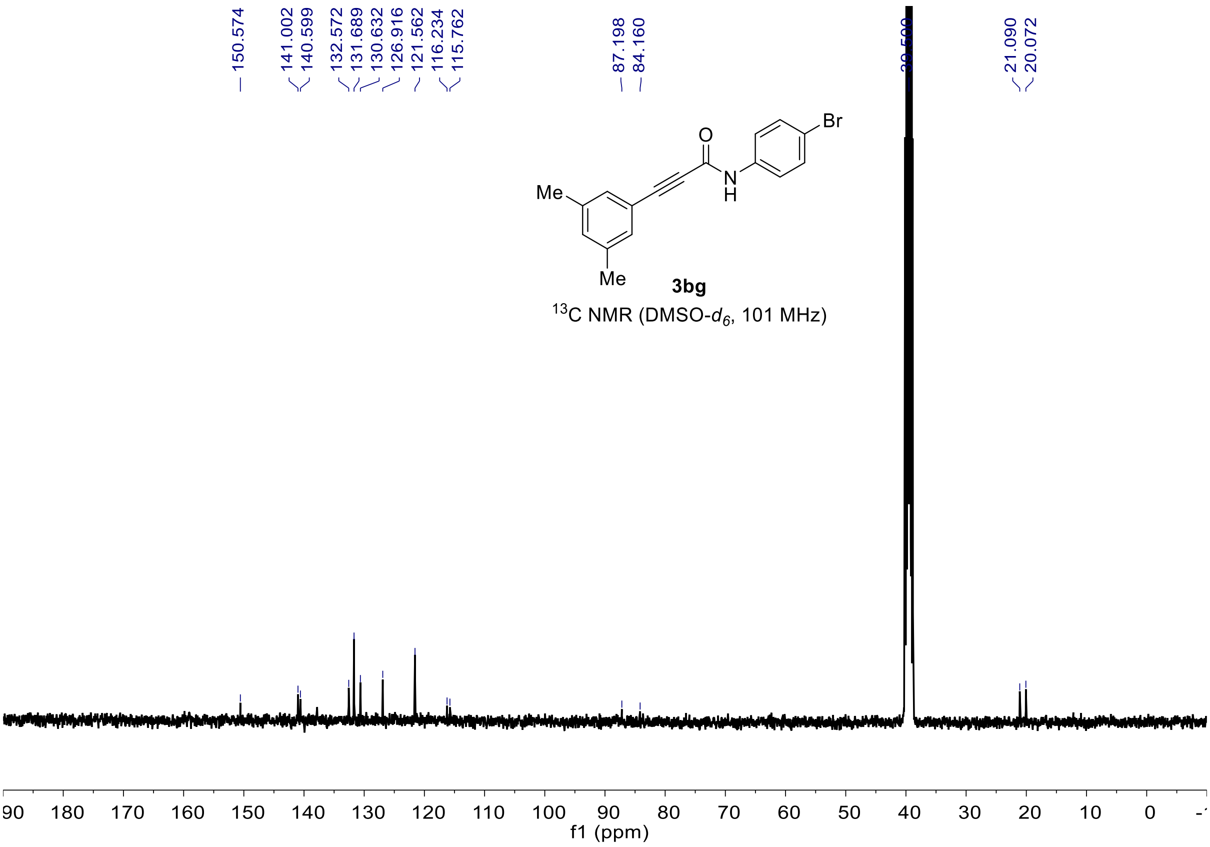

^{13}C NMR (DMSO-d_6, 101 MHz)

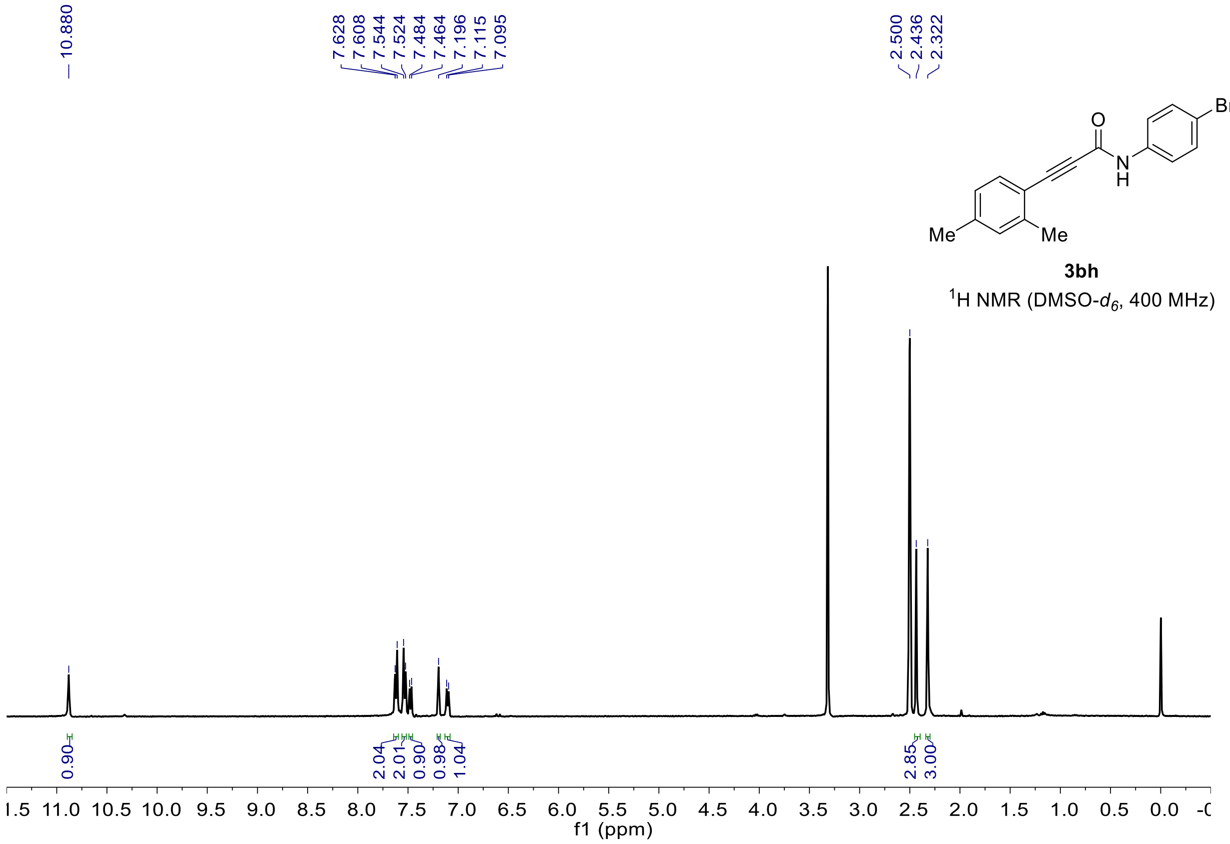
10.880
7.628
7.608
7.544
7.524
7.484
7.464
7.196
7.115
7.095
2.500
2.436
2.322
0.90
2.04
2.01
0.90
0.98
1.04
2.85
3.00
f1 (ppm)
O
N
H
Br
Me
Me
3bh
^{1}H NMR (DMSO-d_6, 400 MHz)

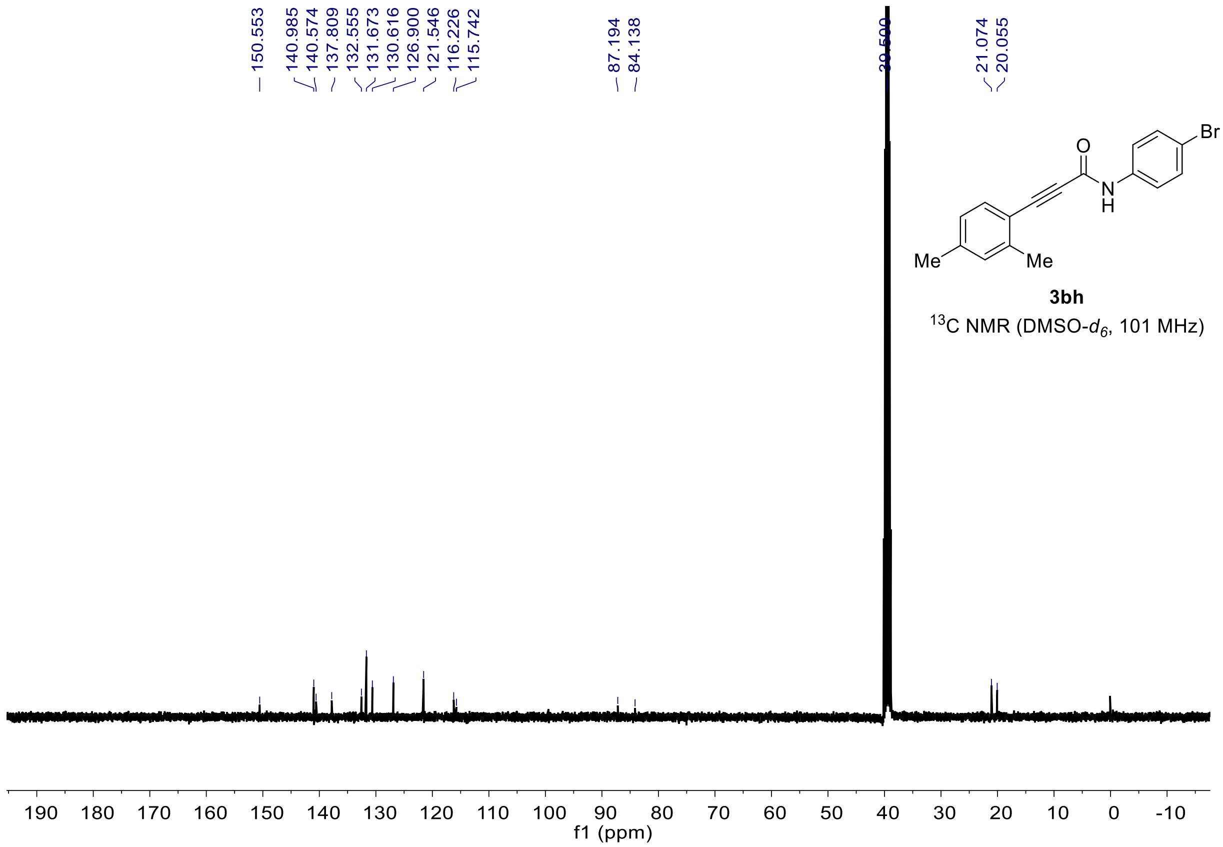
150.553
140.985
140.574
137.809
132.555
131.673
130.616
126.900
121.546
116.226
115.742
87.194
84.138
21.074
20.055
f1 (ppm)
O
N
H
Br
Me
Me
3bh
^{13}C NMR (DMSO-d_6, 101 MHz)

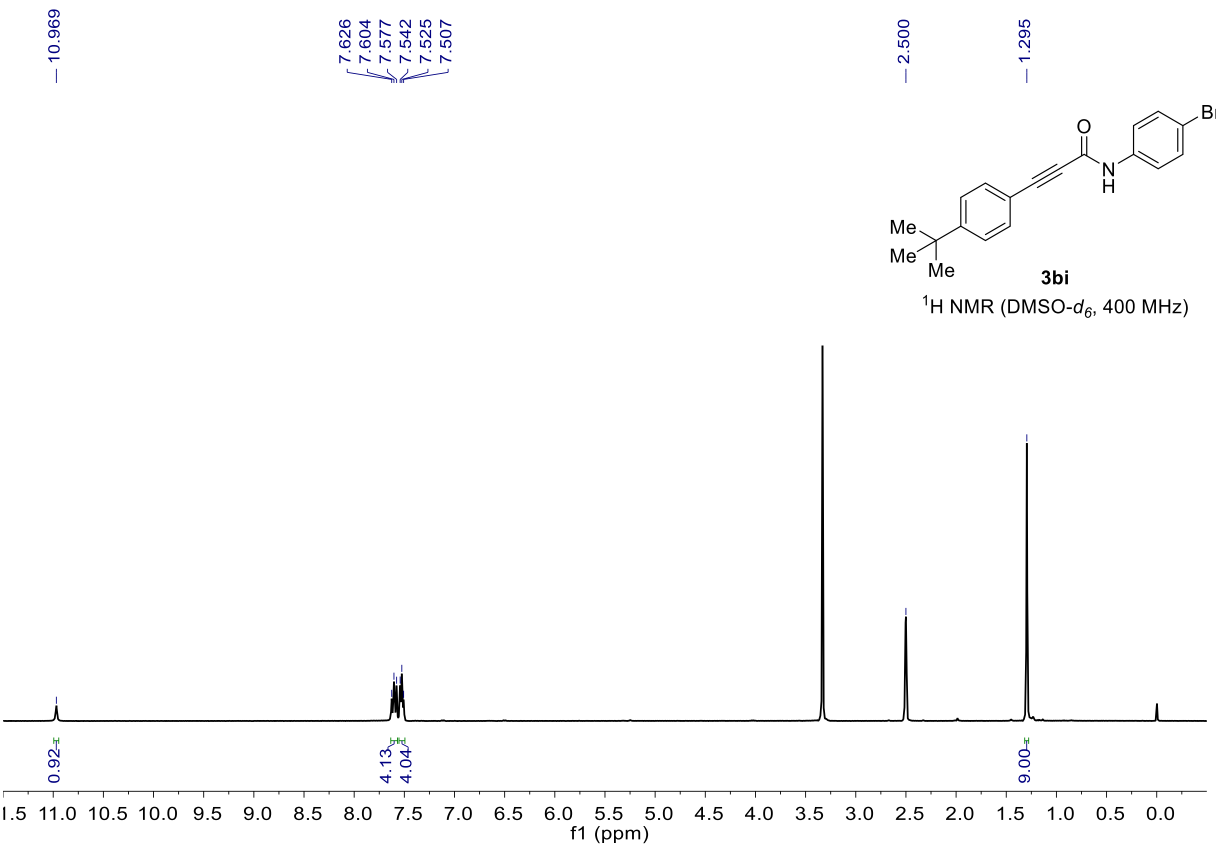
10.969
7.626
7.604
7.577
7.542
7.525
7.507
2.500
1.295
O
N
H
Br
Me
Me
Me
3bi
^{1}H NMR (DMSO-d_6, 400 MHz)
0.92
4.13
4.04
9.00
f1 (ppm)

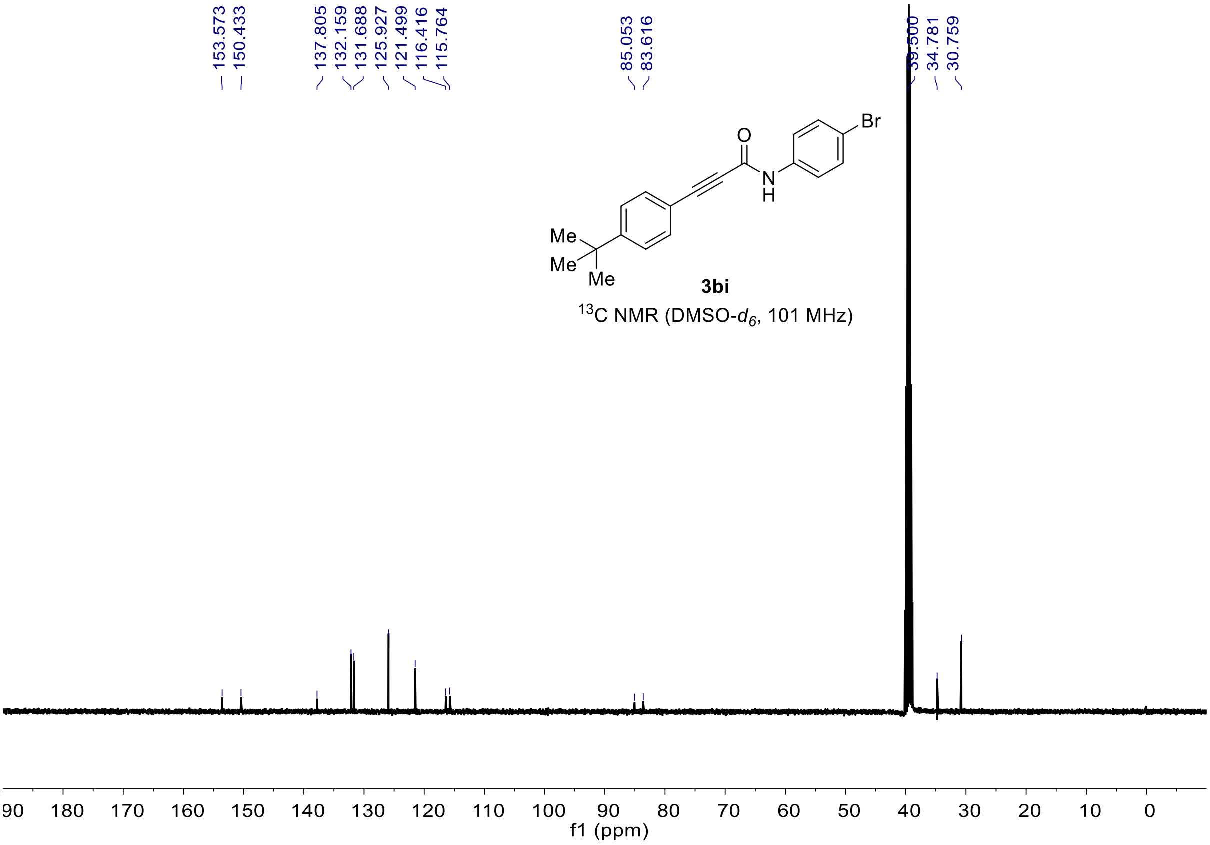
153.573
150.433
137.805
132.159
131.688
125.927
121.499
116.416
115.764
85.053
83.616
39.500
34.781
30.759
O
N
H
Br
Me
Me
Me
3bi
^{13}C NMR (DMSO-d_6, 101 MHz)
f1 (ppm)

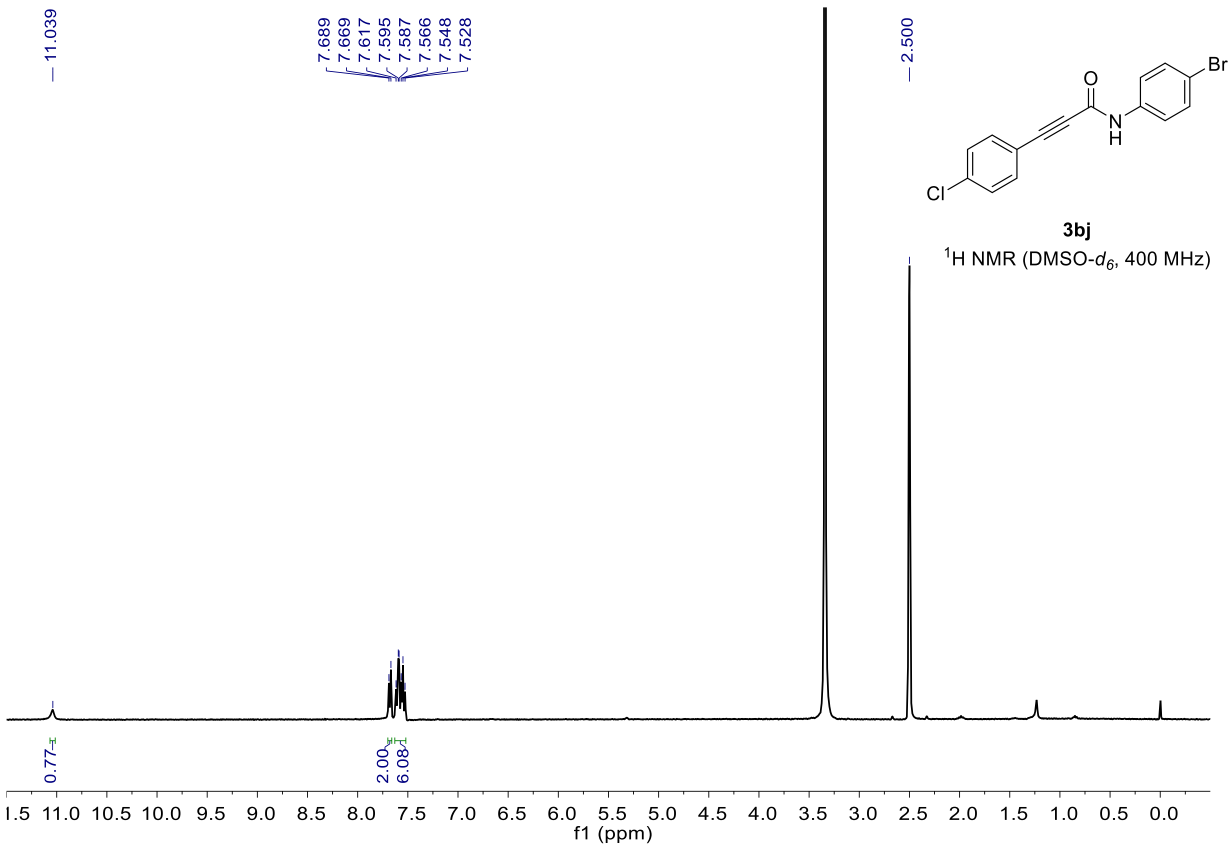

3bj

^{1}H NMR (DMSO-d_6, 400 MHz)

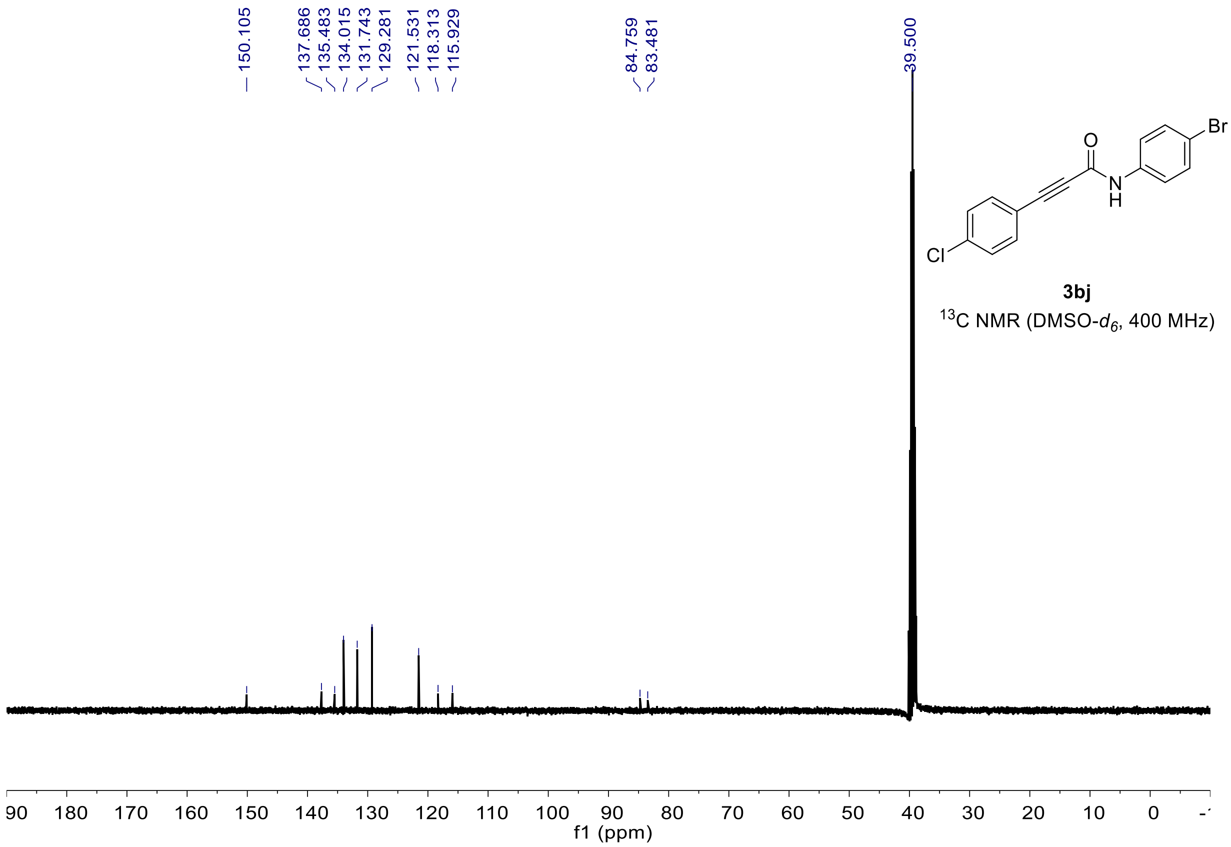

3bj

^{13}C NMR (DMSO-d_6, 400 MHz)

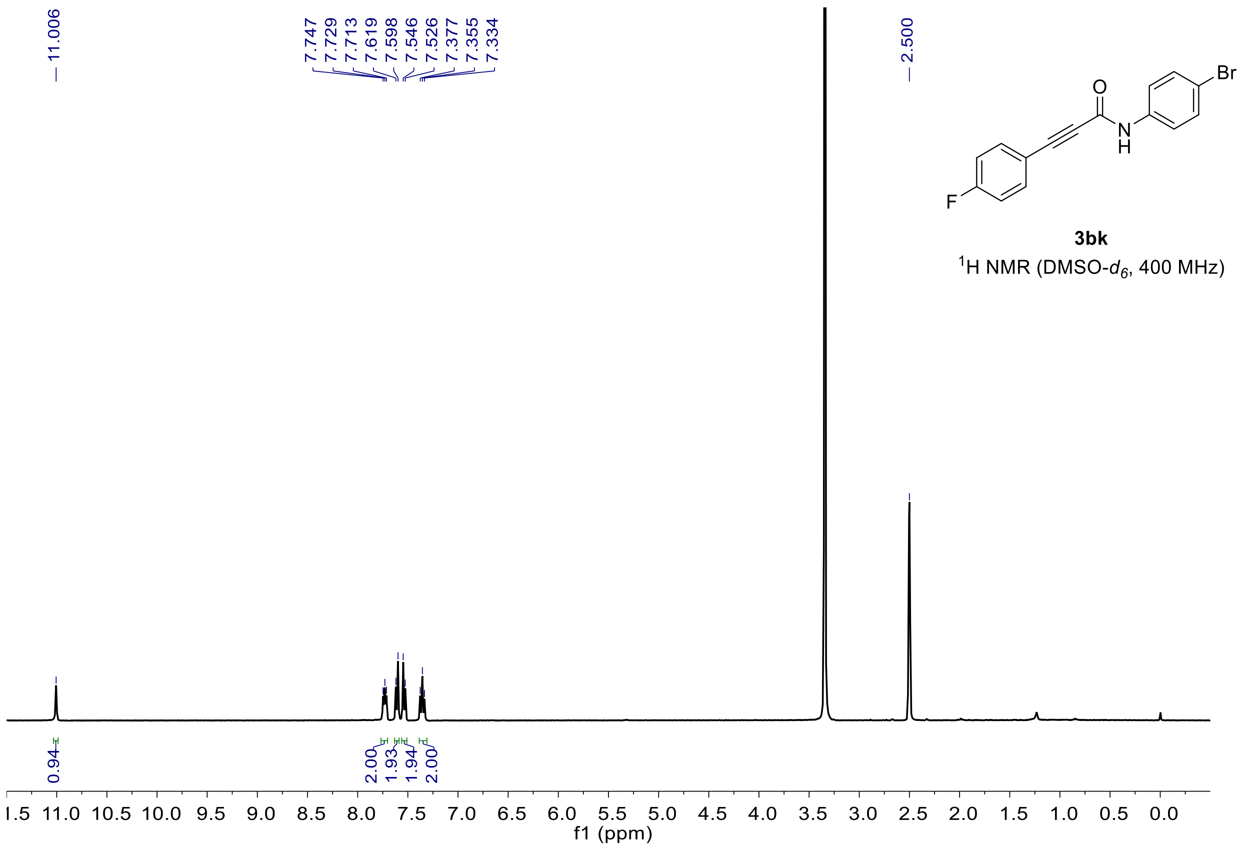
11.006
7.747
7.729
7.713
7.619
7.598
7.546
7.526
7.377
7.355
7.334
2.500
0.94
2.00
1.93
1.94
2.00
f1 (ppm)
3bk
^{1}H NMR (DMSO-d_6, 400 MHz)

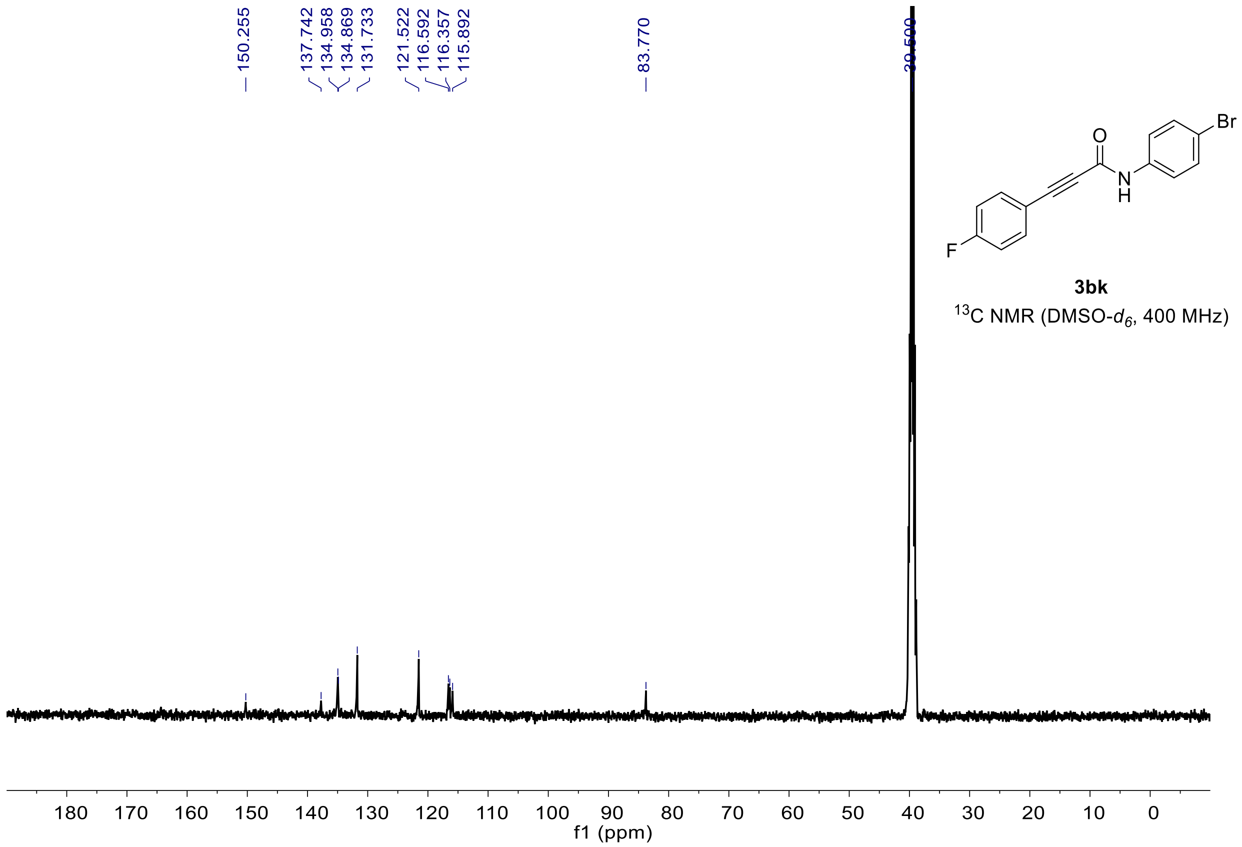
150.255
137.742
134.958
134.869
131.733
121.522
116.592
116.357
115.892
83.770
f1 (ppm)
3bk
^{13}C NMR (DMSO-d_6, 400 MHz)

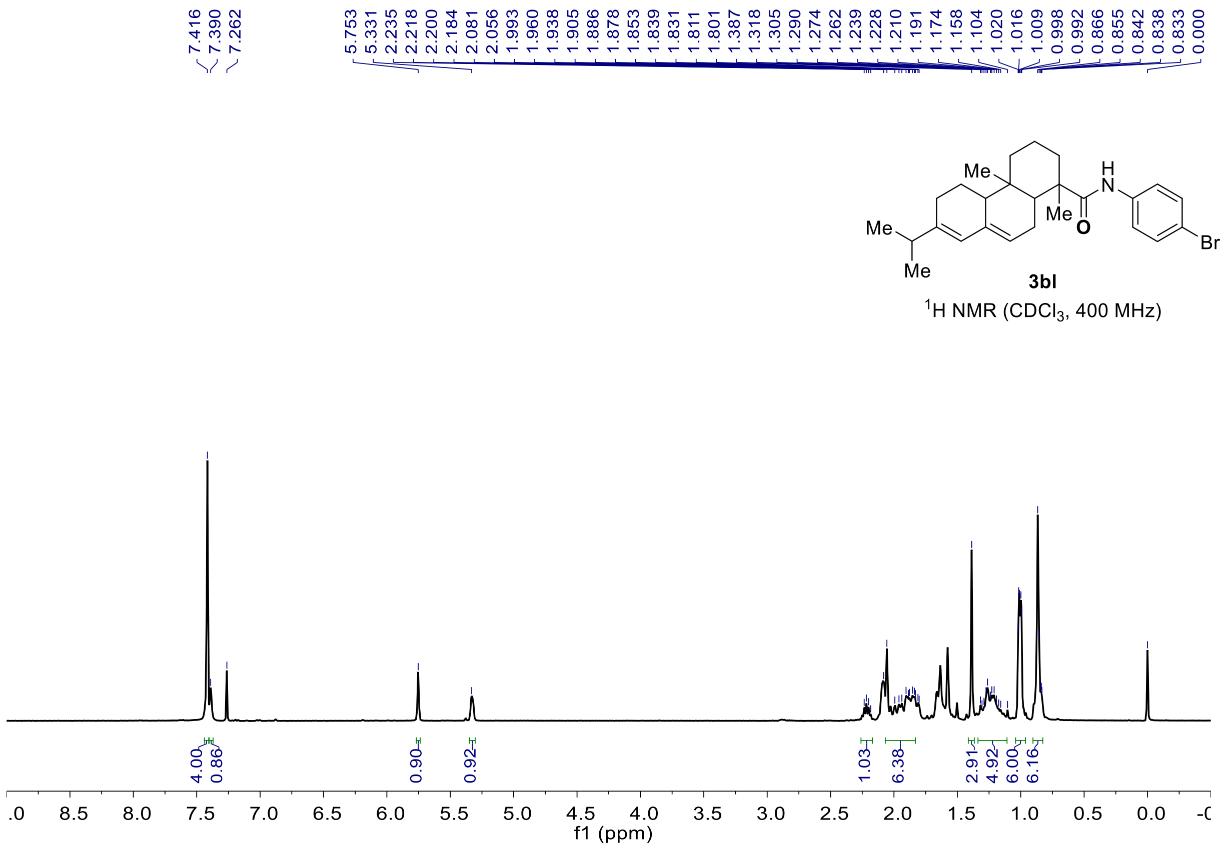
7.416
7.390
7.262
5.753
5.331
2.235
2.218
2.200
2.184
2.081
2.056
1.993
1.960
1.938
1.905
1.886
1.878
1.853
1.839
1.831
1.811
1.801
1.387
1.318
1.305
1.290
1.274
1.262
1.239
1.228
1.210
1.191
1.174
1.158
1.104
1.020
1.016
1.009
0.998
0.992
0.866
0.855
0.842
0.838
0.833
0.000
Me
H
N
Me
O
Me
Br
Me
3bl
^{1}H NMR ($CDCl_3$, 400 MHz)
4.00
0.86
0.90
0.92
1.03
6.38
2.91
4.92
6.00
6.16
f1 (ppm)

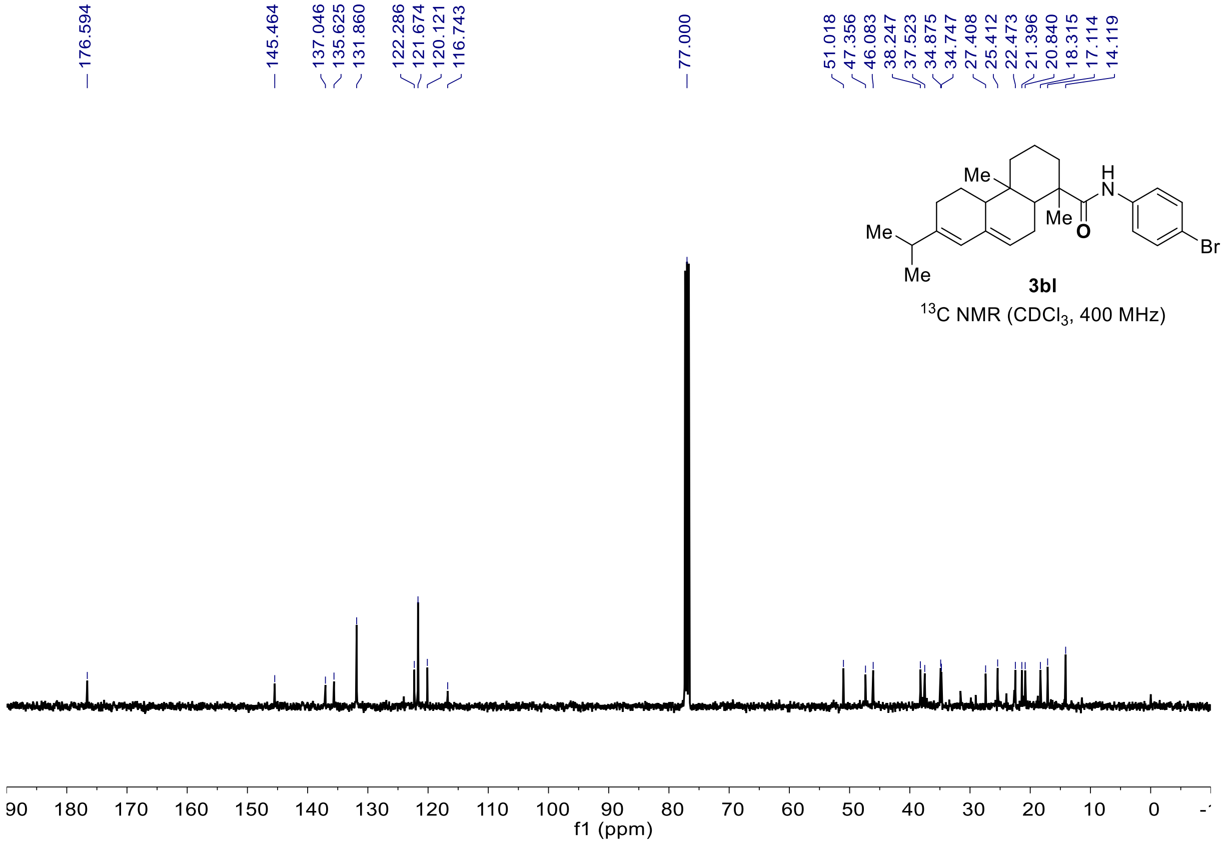
176.594
145.464
137.046
135.625
131.860
122.286
121.674
120.121
116.743
77.000
51.018
47.356
46.083
38.247
37.523
34.875
34.747
27.408
25.412
22.473
21.396
20.840
18.315
17.114
14.119
Me
H
N
Me
O
Me
Br
Me
3bl
^{13}C NMR ($CDCl_3$, 400 MHz)
f1 (ppm)

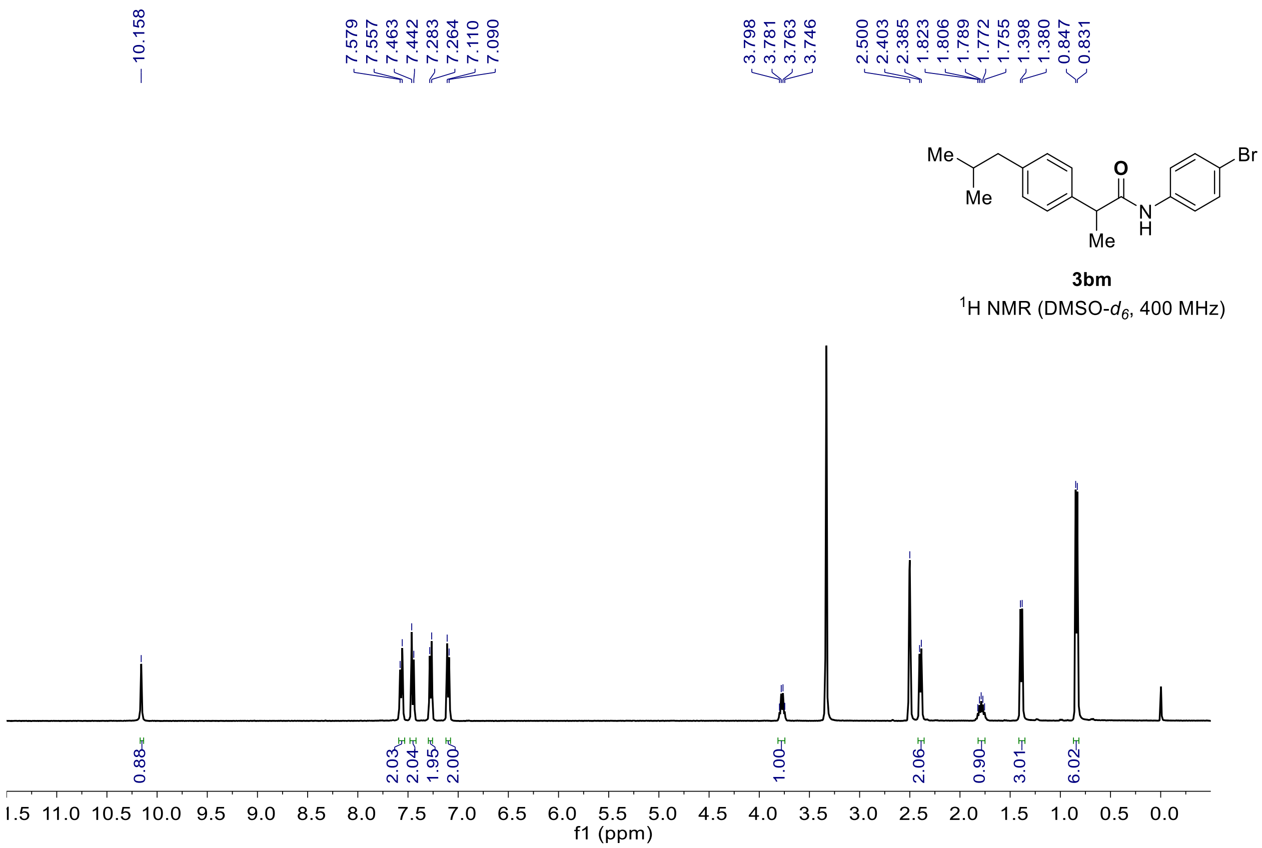
10.158
7.579
7.557
7.463
7.442
7.283
7.264
7.110
7.090
3.798
3.781
3.763
3.746
2.500
2.403
2.385
1.823
1.806
1.789
1.772
1.755
1.398
1.380
0.847
0.831
Me
Me
O
N
H
Br
Me
3bm
1H NMR (DMSO-d6, 400 MHz)
0.88
2.03
2.04
1.95
2.00
1.00
2.06
0.90
3.01
6.02
f1 (ppm)

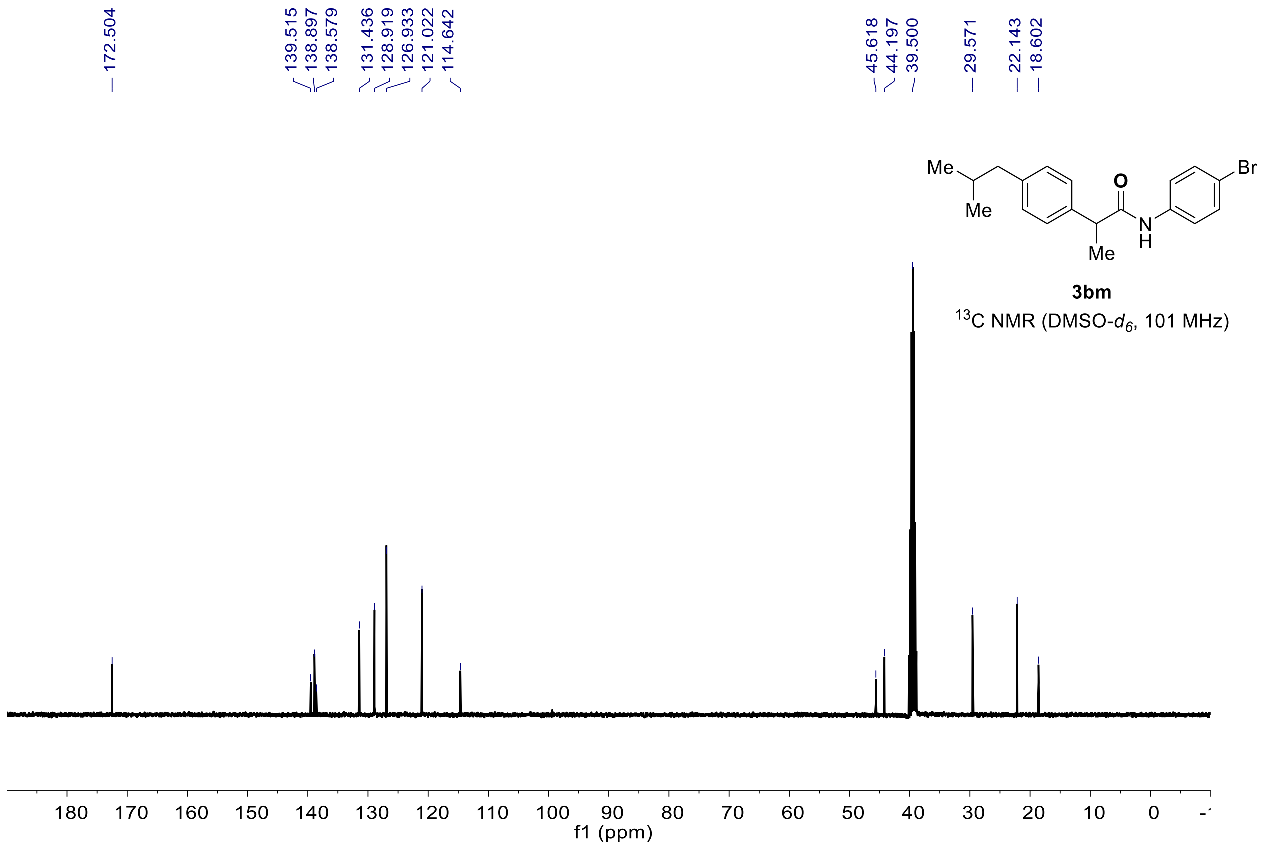
172.504
139.515
138.897
138.579
131.436
128.919
126.933
121.022
114.642
45.618
44.197
39.500
29.571
22.143
18.602
Me
Me
O
N
H
Br
Me
3bm
13C NMR (DMSO-d6, 101 MHz)
f1 (ppm)

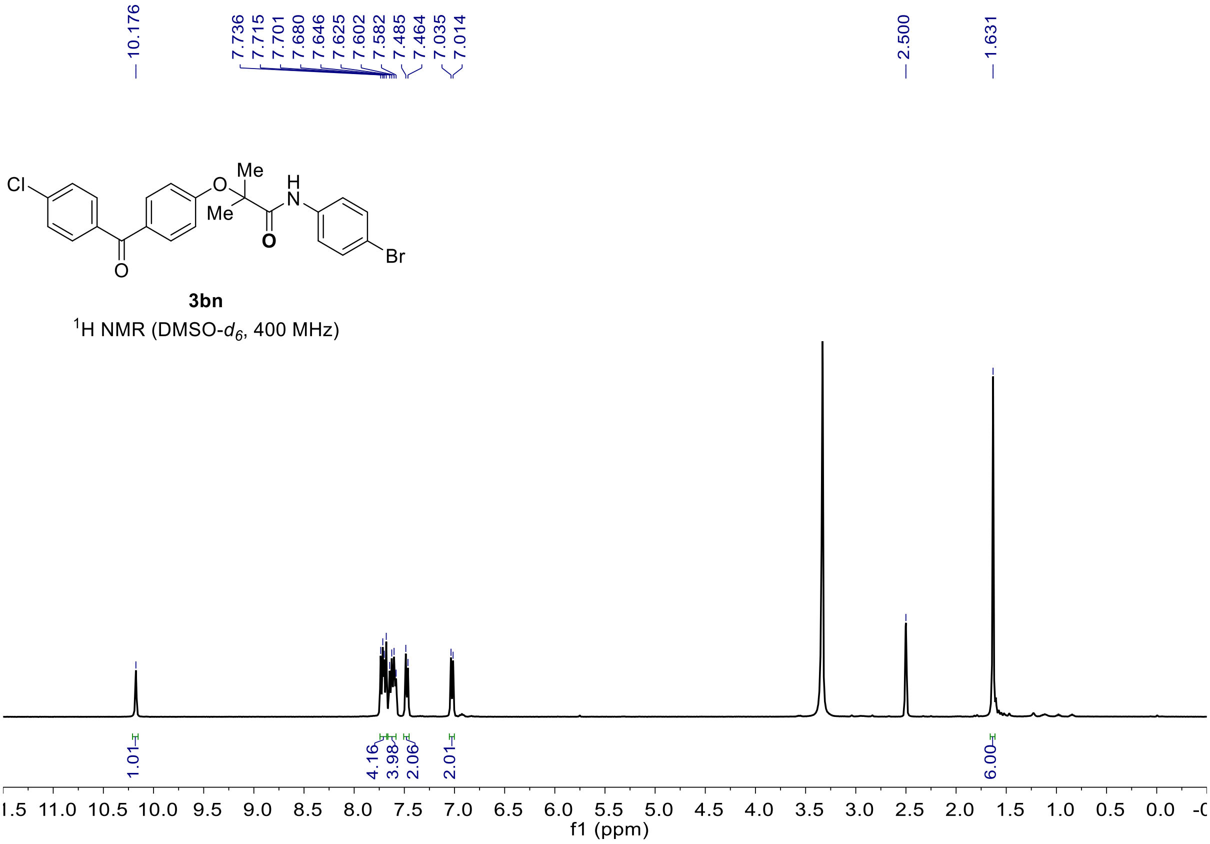
10.176
7.736
7.715
7.701
7.680
7.646
7.625
7.602
7.582
7.485
7.464
7.035
7.014
2.500
1.631
Cl
O
Me
Me
H
N
O
O
Br
3bn
^{1}H NMR (DMSO-d_6, 400 MHz)
1.01
4.16
3.98
2.06
2.01
6.00
f1 (ppm)

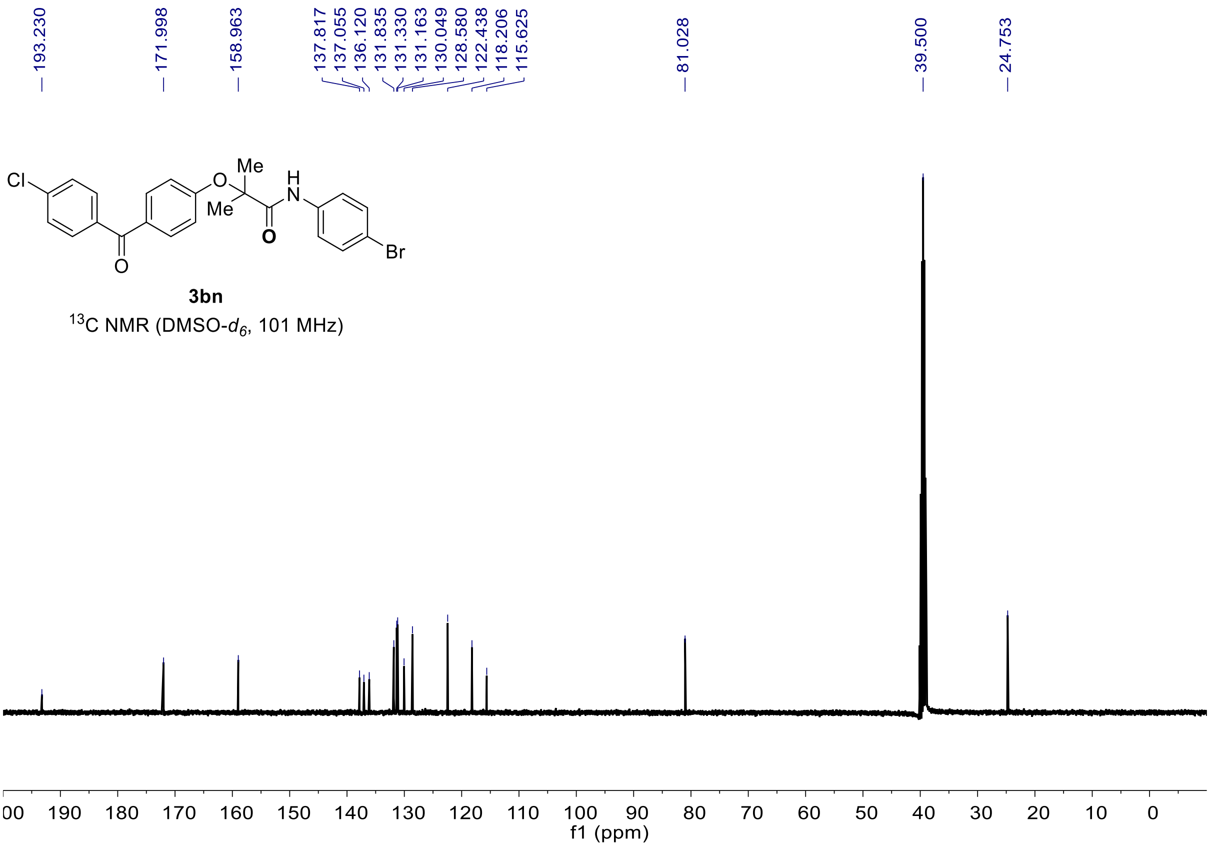
193.230
171.998
158.963
137.817
137.055
136.120
131.835
131.330
131.163
130.049
128.580
122.438
118.206
115.625
81.028
39.500
24.753
Cl
O
Me
Me
H
N
O
O
Br
3bn
^{13}C NMR (DMSO-d_6, 101 MHz)
f1 (ppm)

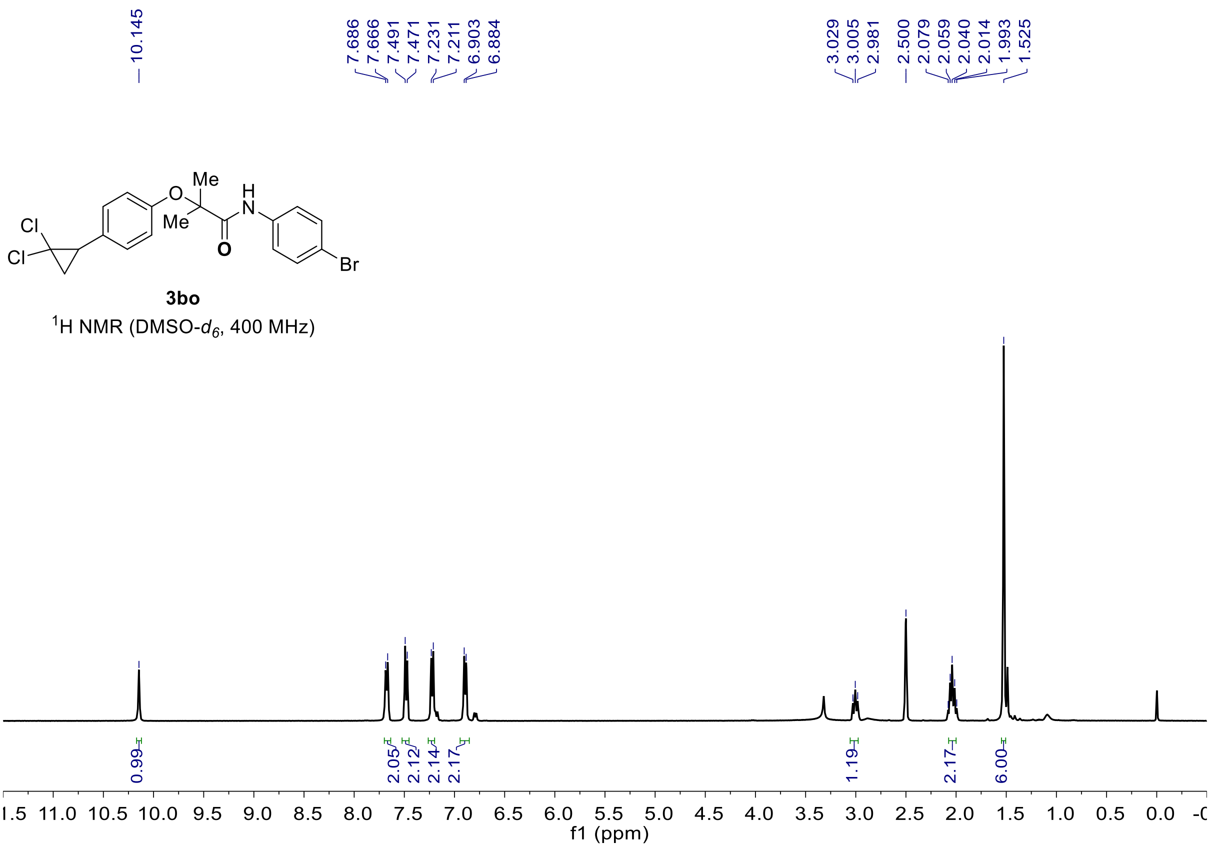

3bo

^{1}H NMR (DMSO-d_6, 400 MHz)

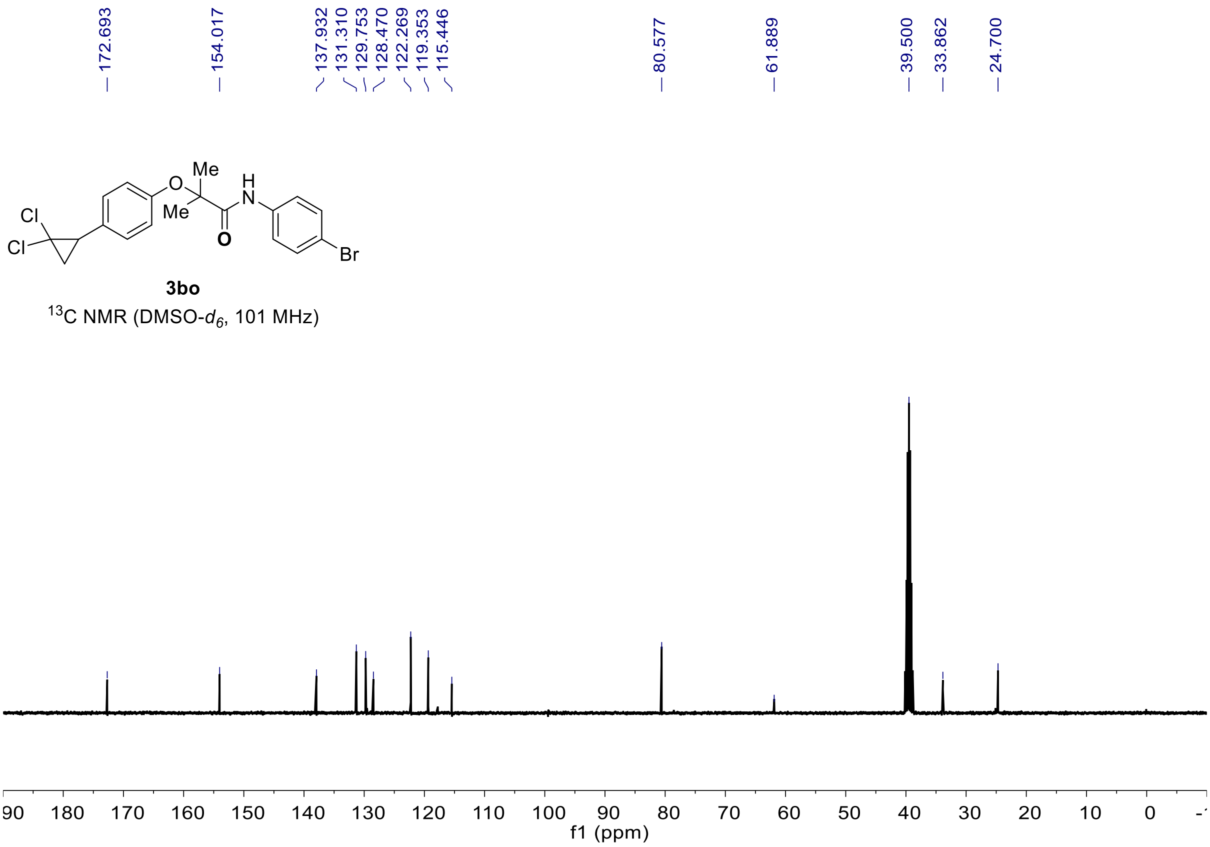

3bo

^{13}C NMR (DMSO-d_6, 101 MHz)

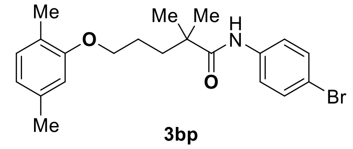

3bp

^{1}H NMR (DMSO-d_6, 400 MHz)

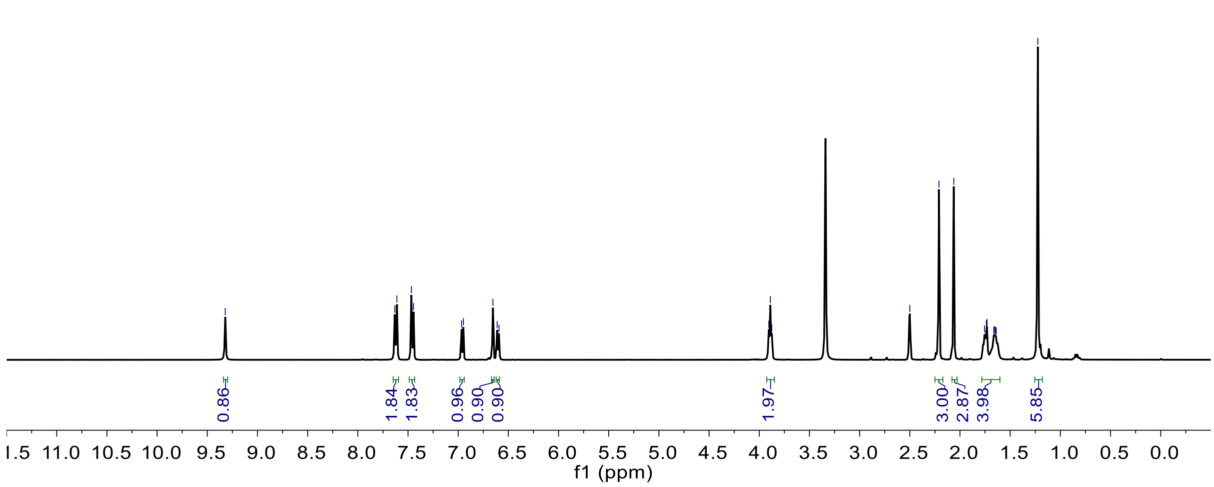

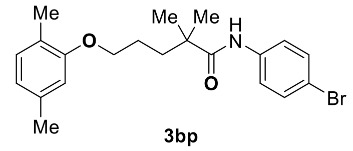

3bp

^{13}C NMR (DMSO-d_6, 101 MHz)

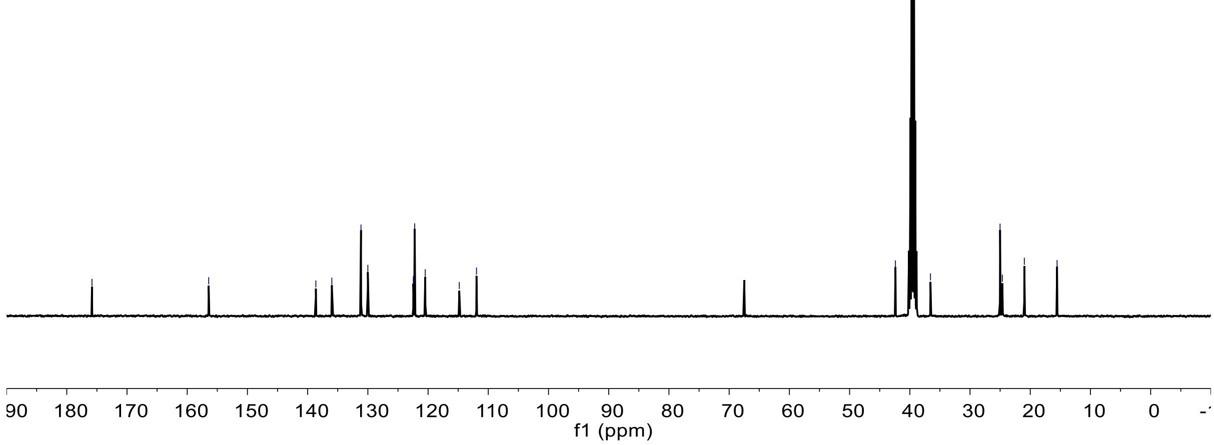

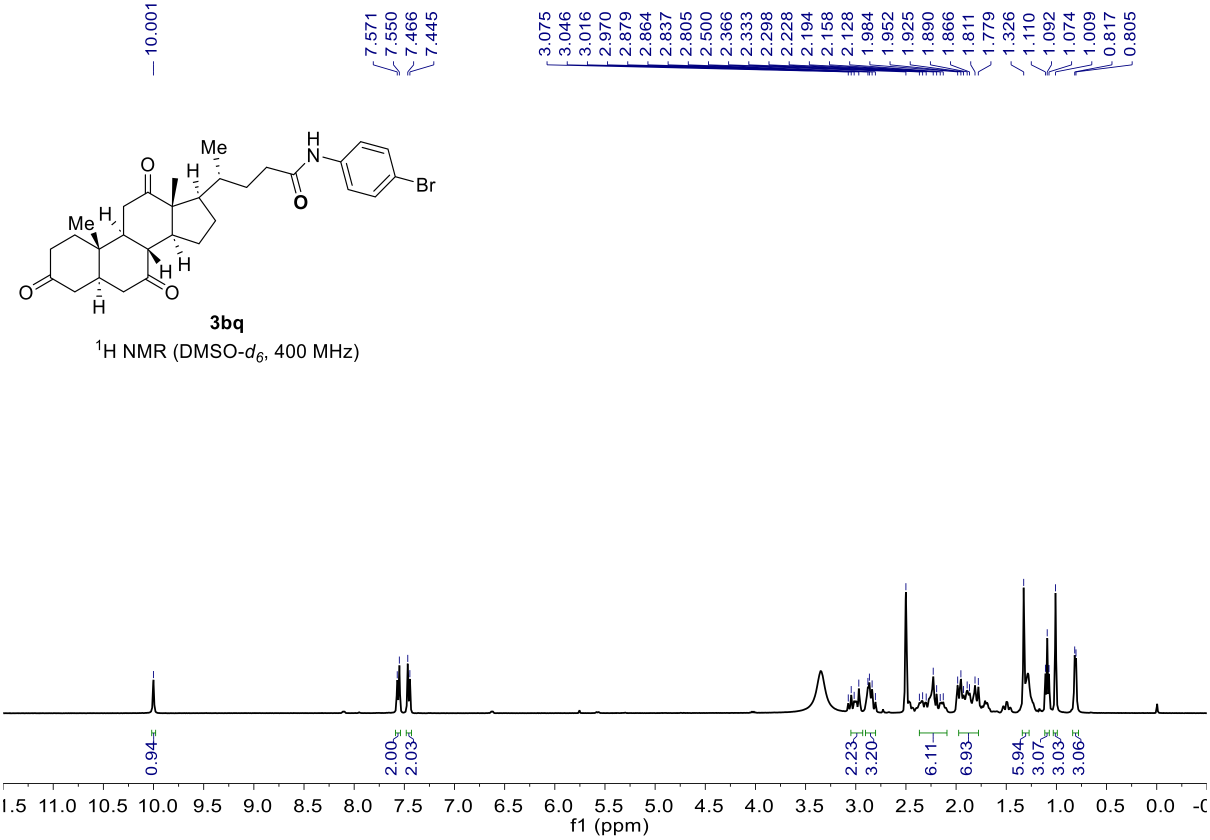
10.001
7.571
7.550
7.466
7.445
3.075
3.046
3.016
2.970
2.879
2.864
2.837
2.805
2.500
2.366
2.333
2.298
2.228
2.194
2.158
2.128
1.984
1.952
1.925
1.890
1.866
1.811
1.779
1.326
1.110
1.092
1.074
1.009
0.817
0.805
Me
H
N
O
Br
3bq
1H NMR (DMSO-d6, 400 MHz)
0.94
2.00
2.03
2.23
3.20
6.11
6.93
5.94
3.07
3.03
3.06
f1 (ppm)

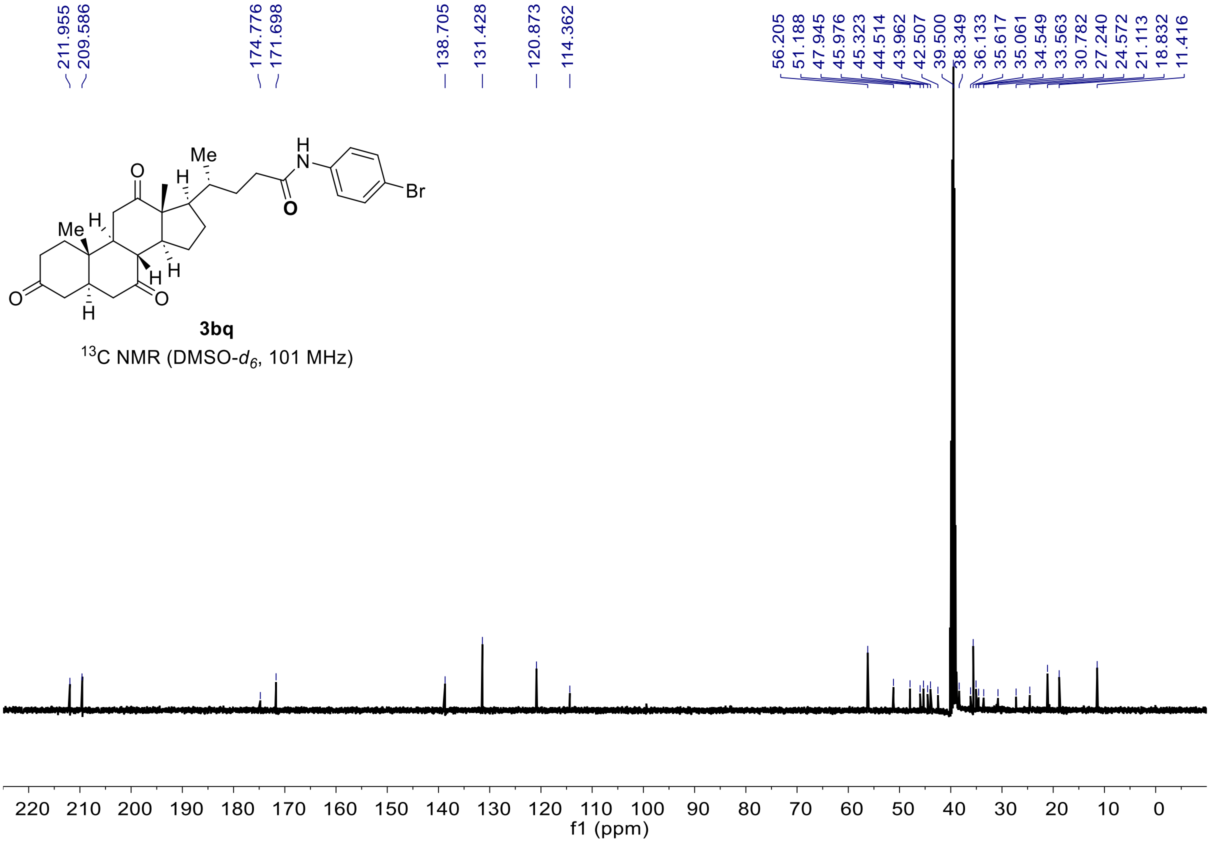
211.955
209.586
174.776
171.698
138.705
131.428
120.873
114.362
56.205
51.188
47.945
45.976
45.323
44.514
43.962
42.507
39.500
38.349
36.133
35.617
35.061
34.549
33.563
30.782
27.240
24.572
21.113
18.832
11.416
Me
H
N
O
Br
3bq
13C NMR (DMSO-d6, 101 MHz)
f1 (ppm)

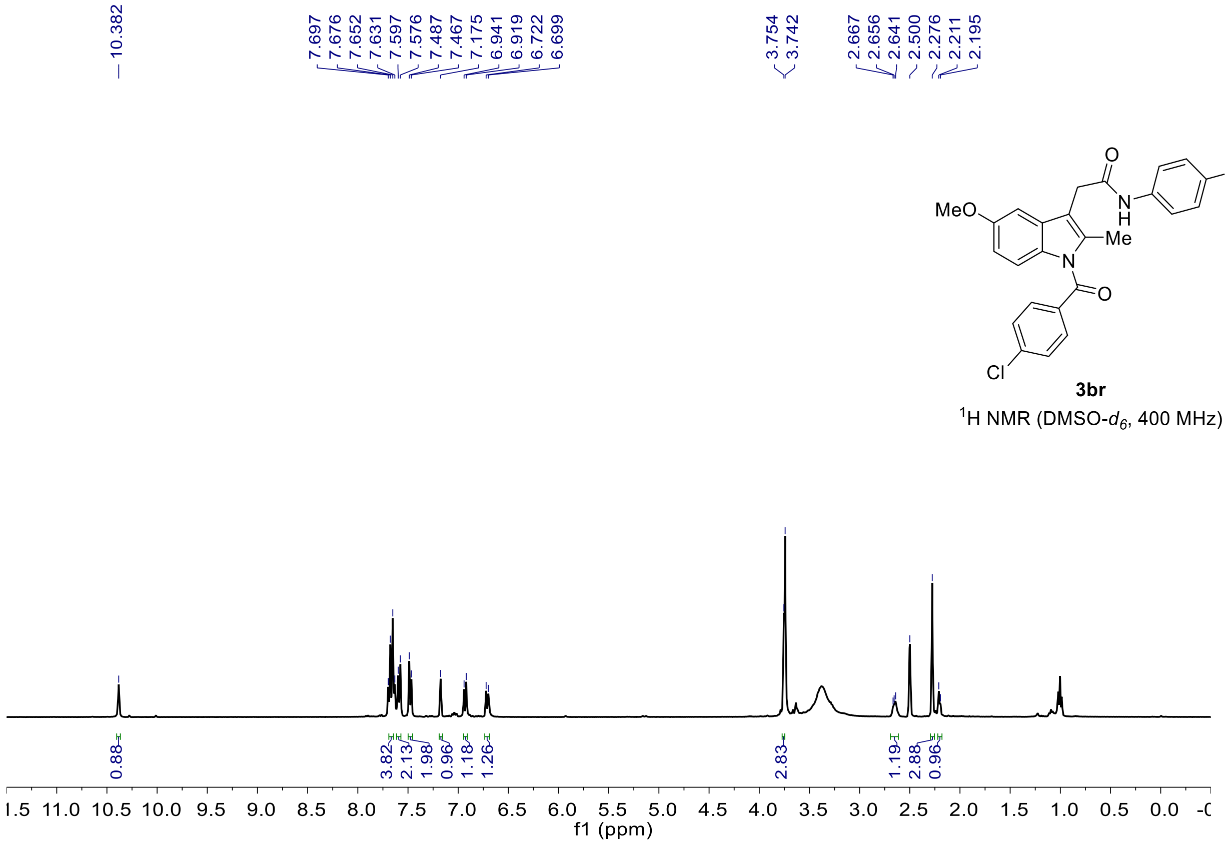
10.382
7.697
7.676
7.652
7.631
7.597
7.576
7.487
7.467
7.175
6.941
6.919
6.722
6.699
3.754
3.742
2.667
2.656
2.641
2.500
2.276
2.211
2.195
MeO
O
N
H
Br
Me
N
O
Cl
3br
^{1}H NMR (DMSO-d_6, 400 MHz)
0.88
3.82
2.13
1.98
0.96
1.18
1.26
2.83
1.19
2.88
0.96
f1 (ppm)

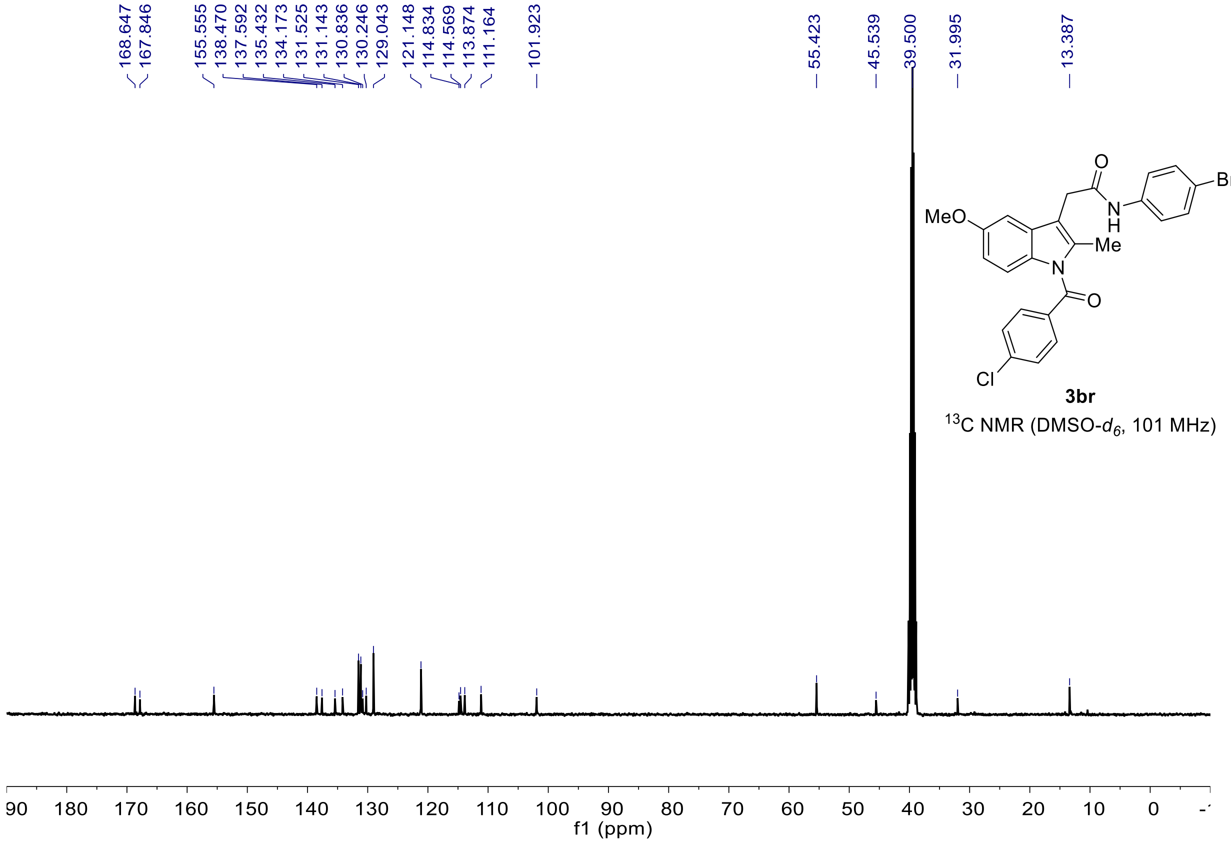
168.647
167.846
155.555
138.470
137.592
135.432
134.173
131.525
131.143
130.836
130.246
129.043
121.148
114.834
114.569
113.874
111.164
101.923
55.423
45.539
39.500
31.995
13.387
MeO
O
N
H
Br
Me
N
O
Cl
3br
^{13}C NMR (DMSO-d_6, 101 MHz)
f1 (ppm)

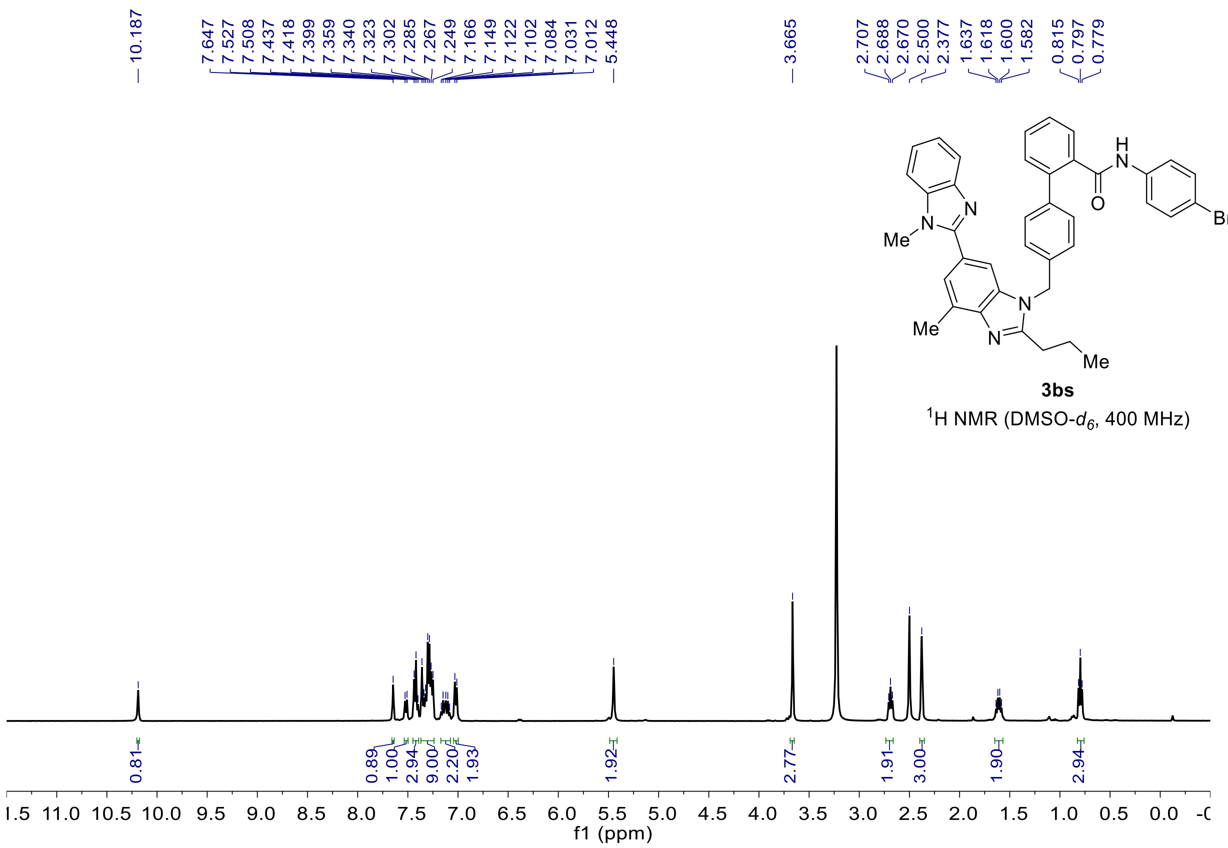
10.187
7.647 7.527 7.508 7.437 7.418 7.399 7.359 7.340 7.323 7.302 7.285 7.267 7.249 7.166 7.149 7.122 7.102 7.084 7.031 7.012 5.448
3.665
2.707 2.688 2.670 2.500 2.377 1.637 1.618 1.600 1.582 0.815 0.797 0.779
H
N
O
Br
N
N
Me
Me
N
N
Me
3bs
^{1}H NMR (DMSO-d_6, 400 MHz)
0.81 0.89 1.00 2.94 9.00 2.20 1.93 1.92 2.77 1.91 3.00 1.90 2.94
f1 (ppm)

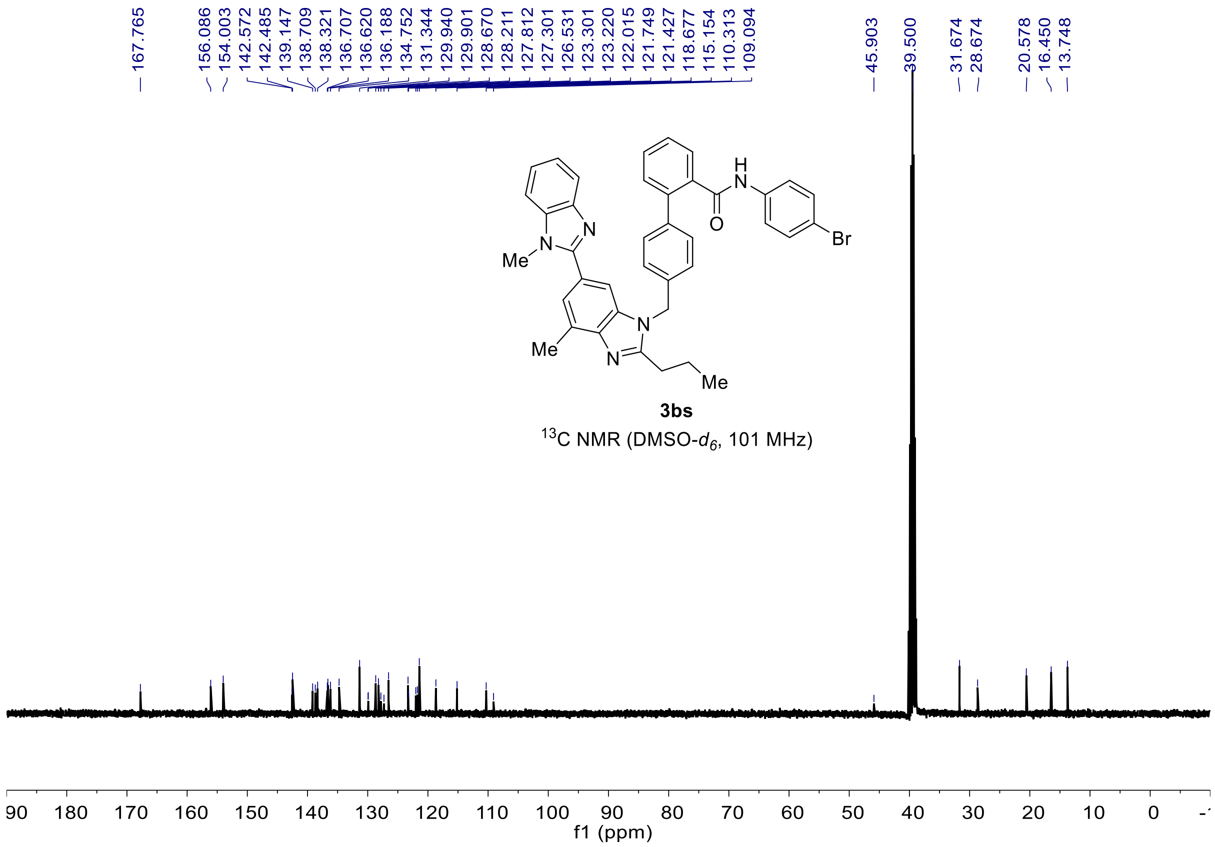
167.765
156.086 154.003 142.572 142.485 139.147 138.709 138.321 136.707 136.620 136.188 134.752 131.344 129.940 129.901 128.670 128.211 127.812 127.301 126.531 123.301 123.220 122.015 121.749 121.427 118.677 115.154 110.313 109.094
45.903 39.500 31.674 28.674 20.578 16.450 13.748
H
N
O
Br
N
N
Me
Me
N
N
Me
3bs
^{13}C NMR (DMSO-d_6, 101 MHz)
f1 (ppm)

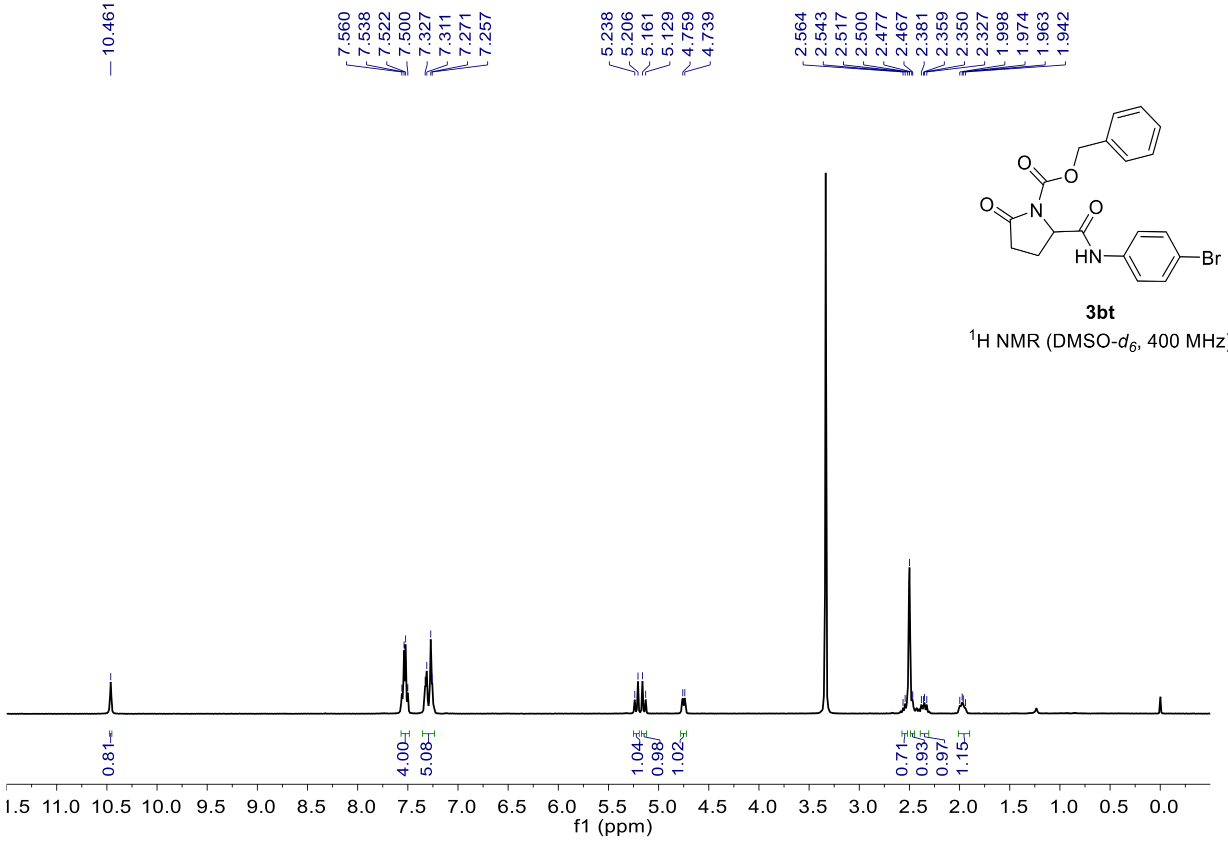

3bt

^1H NMR (DMSO-d_6, 400 MHz)

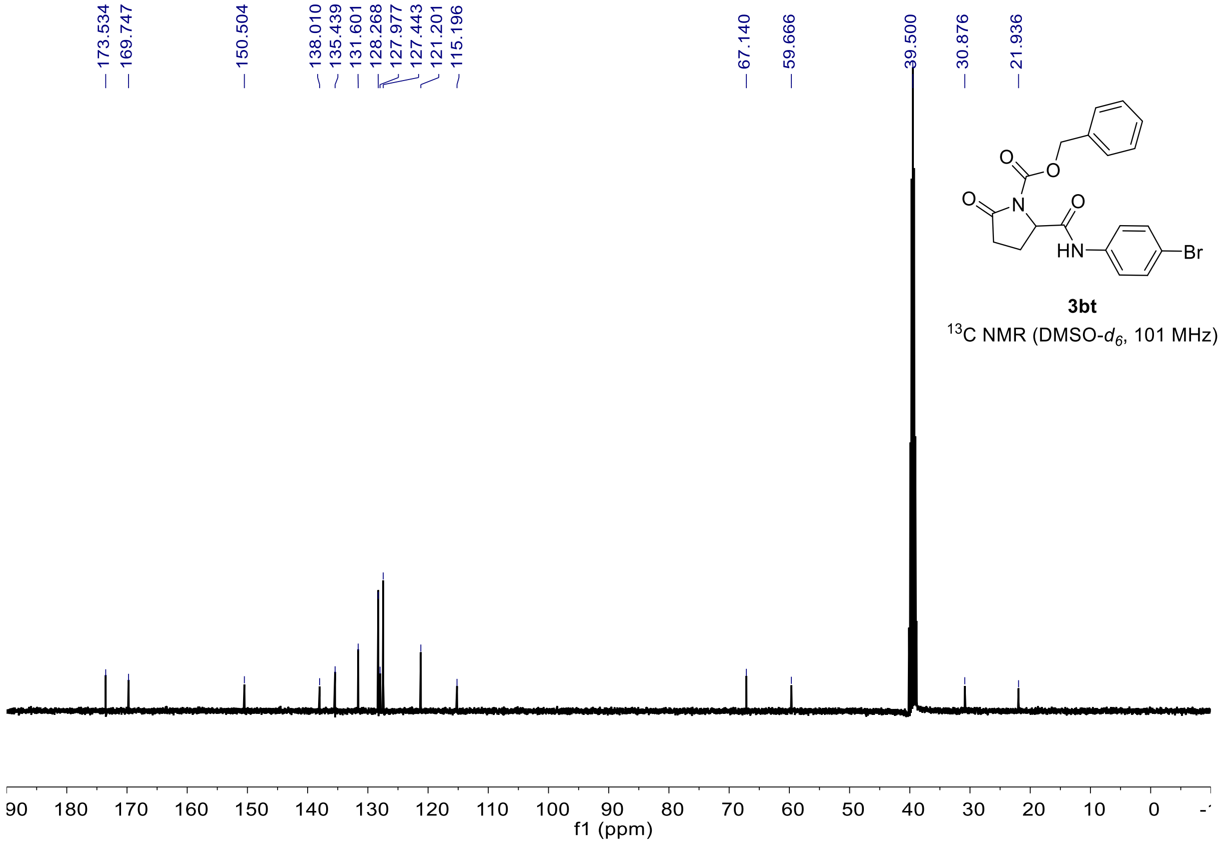

3bt

^{13}C NMR (DMSO-d_6, 101 MHz)

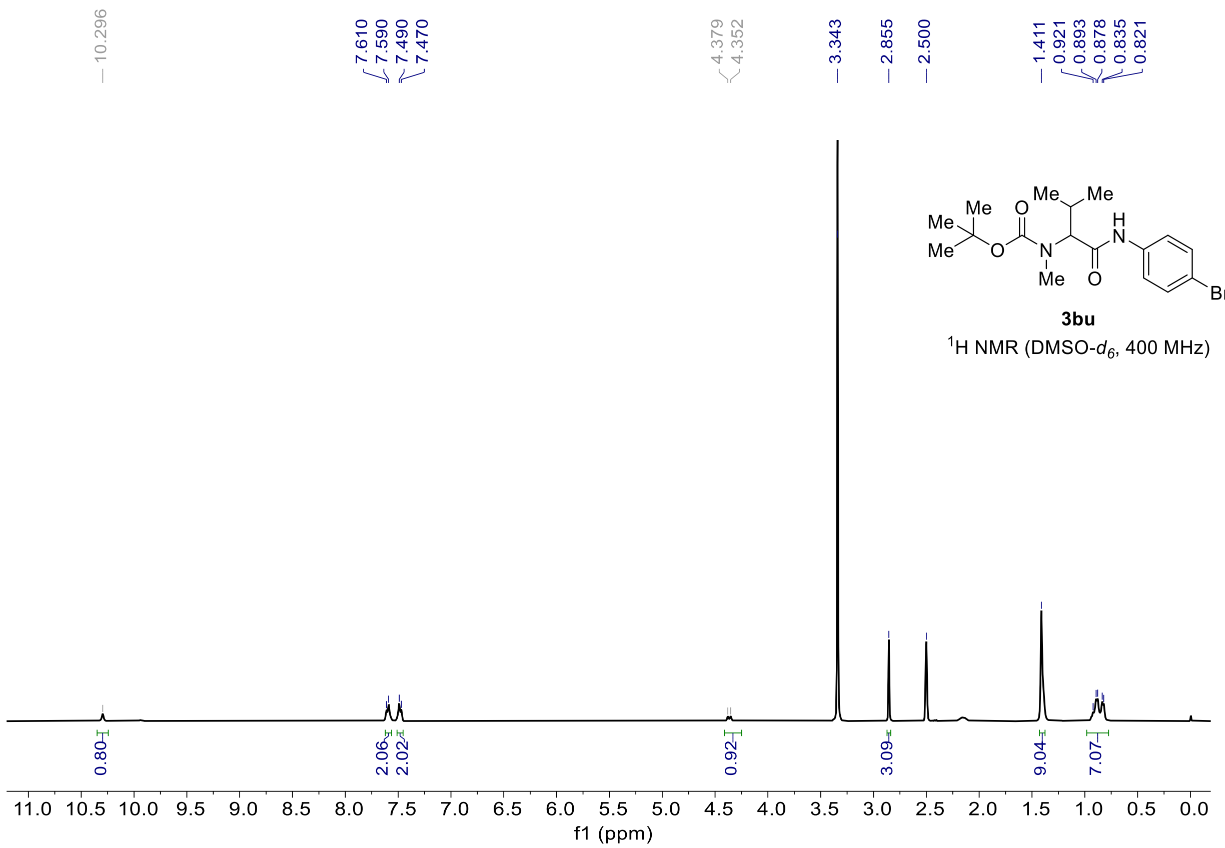
10.296
7.610
7.590
7.490
7.470
4.379
4.352
3.343
2.855
2.500
1.411
0.921
0.893
0.878
0.835
0.821
Me
Me
Me
Me
Me
O
O
N
H
N
Me
O
Br
3bu
1H NMR (DMSO-d6, 400 MHz)
0.80
2.06
2.02
0.92
3.09
9.04
7.07
11.0 10.5 10.0 9.5 9.0 8.5 8.0 7.5 7.0 6.5 6.0 5.5 5.0 4.5 4.0 3.5 3.0 2.5 2.0 1.5 1.0 0.5 0.0
f1 (ppm)

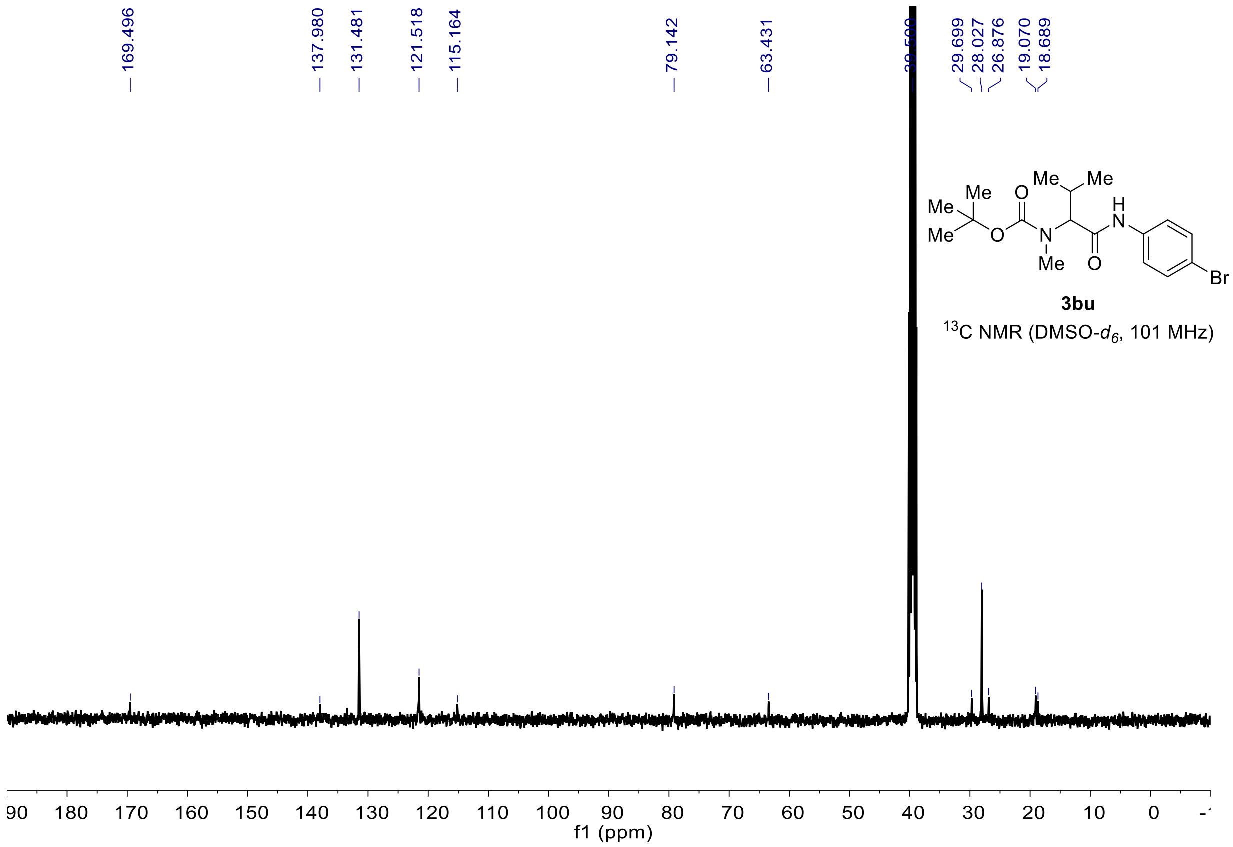
169.496
137.980
131.481
121.518
115.164
79.142
63.431
29.699
28.027
26.876
19.070
18.689
3bu
13C NMR (DMSO-d6, 101 MHz)
170 160 150 140 130 120 110 100 90 80 70 60 50 40 30 20 10 0
f1 (ppm)

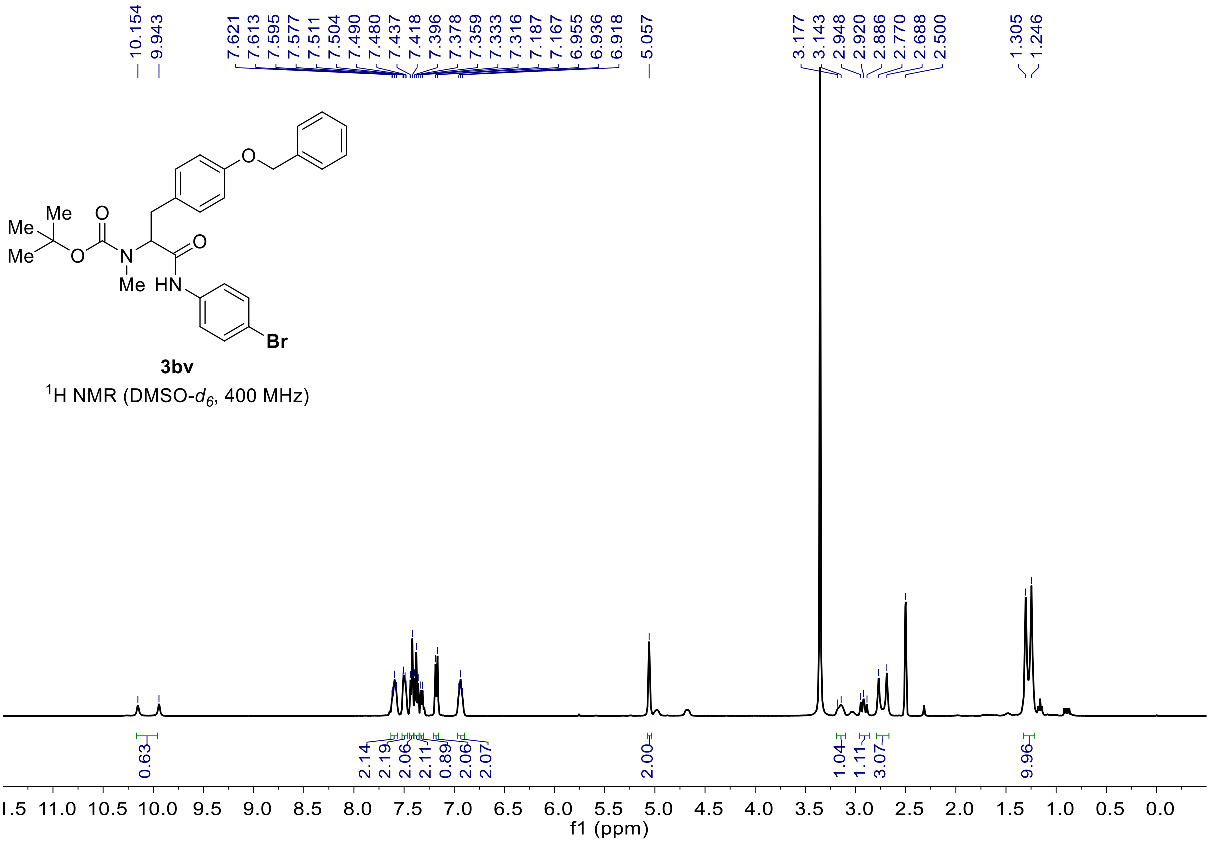
10.154
9.943
7.621 7.613 7.595 7.577 7.511 7.504 7.490 7.480 7.437 7.418 7.396 7.378 7.359 7.333 7.316 7.187 7.167 6.955 6.936 6.918
5.057
3.177 3.143 2.948 2.920 2.886 2.770 2.688 2.500
1.305 1.246
Me
O
O
Me
Me
O
N
Me
HN
Br
3bv
1H NMR (DMSO-d6, 400 MHz)
0.63
2.14 2.19 2.06 2.11 0.89 2.06 2.07
2.00
1.04 1.11 3.07
9.96
f1 (ppm)

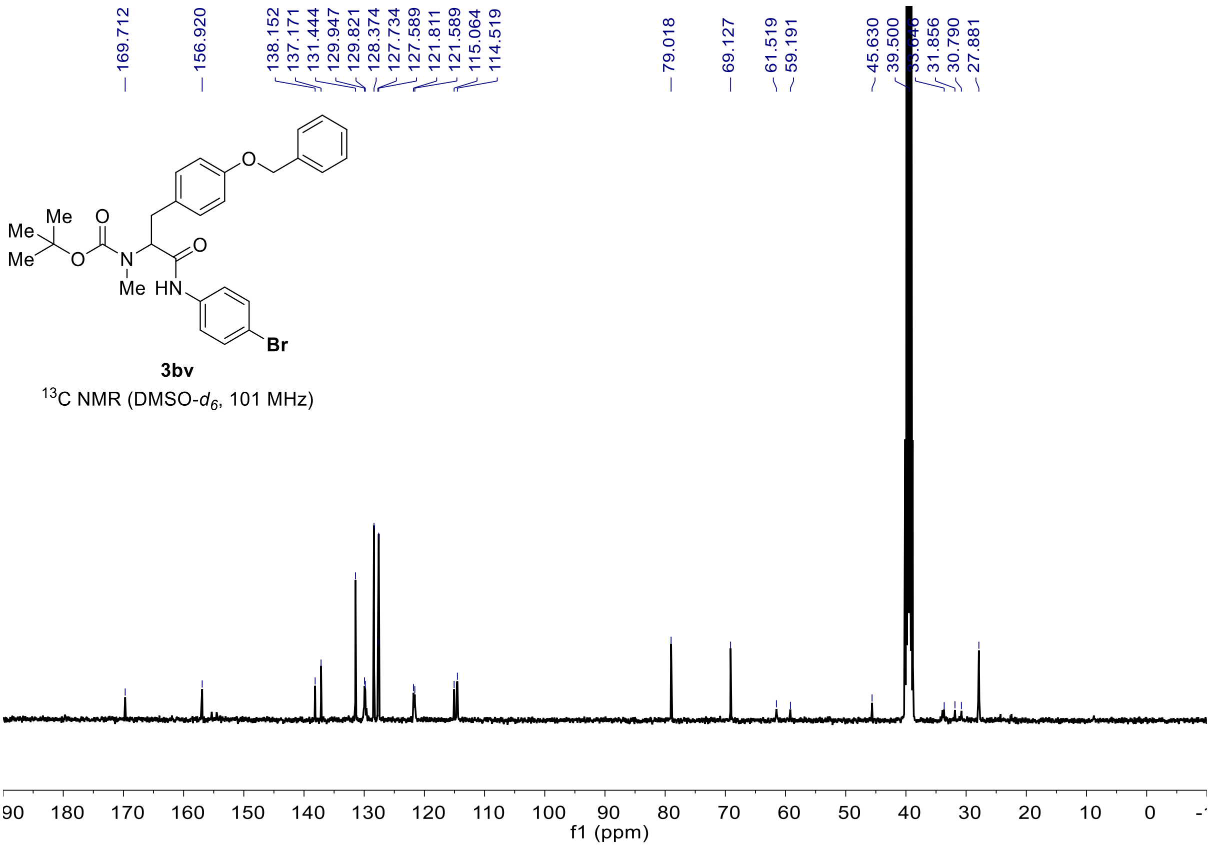
169.712
156.920
138.152 137.171 131.444 129.947 129.821 128.374 127.734 127.589 121.811 121.589 115.064 114.519
79.018
69.127
61.519 59.191
45.630 39.500 31.856 30.790 27.881
3bv
13C NMR (DMSO-d6, 101 MHz)
f1 (ppm)

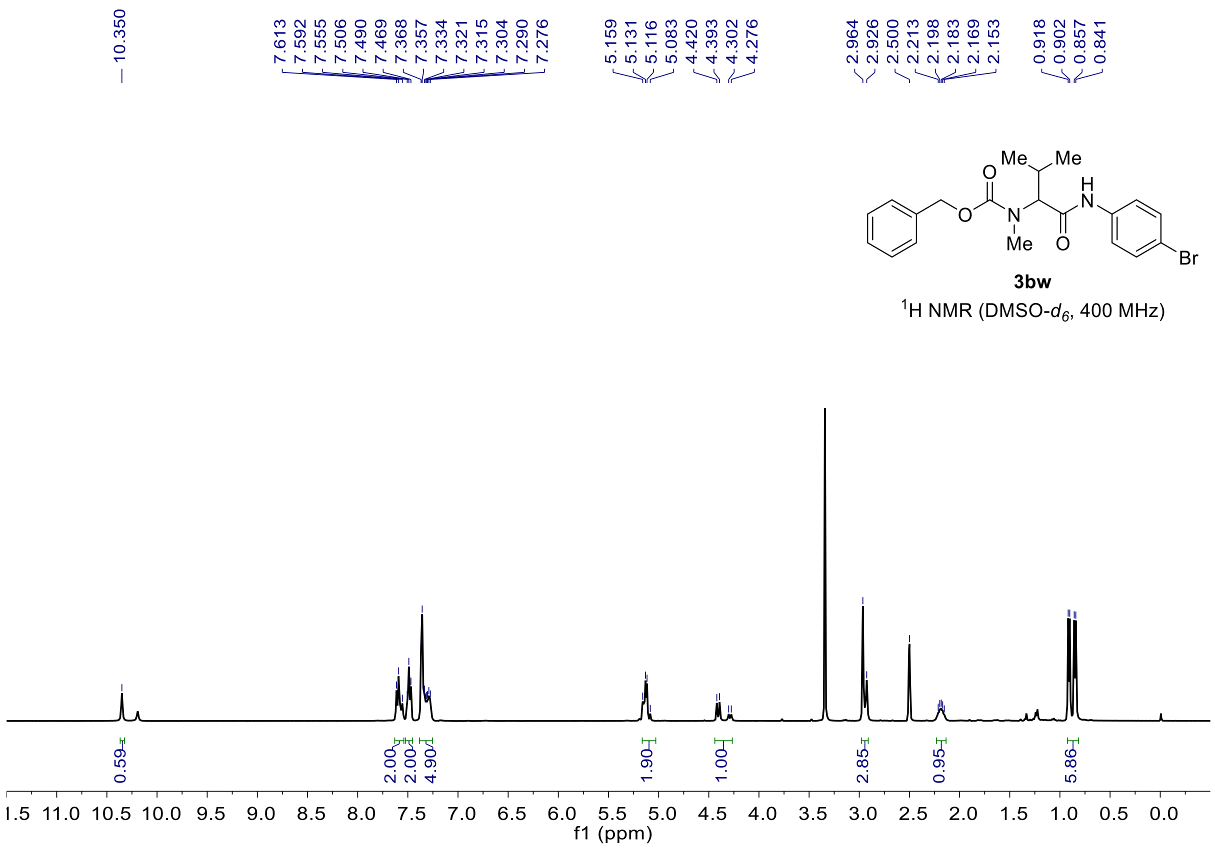

3bw

^{1}H NMR (DMSO-d_6, 400 MHz)

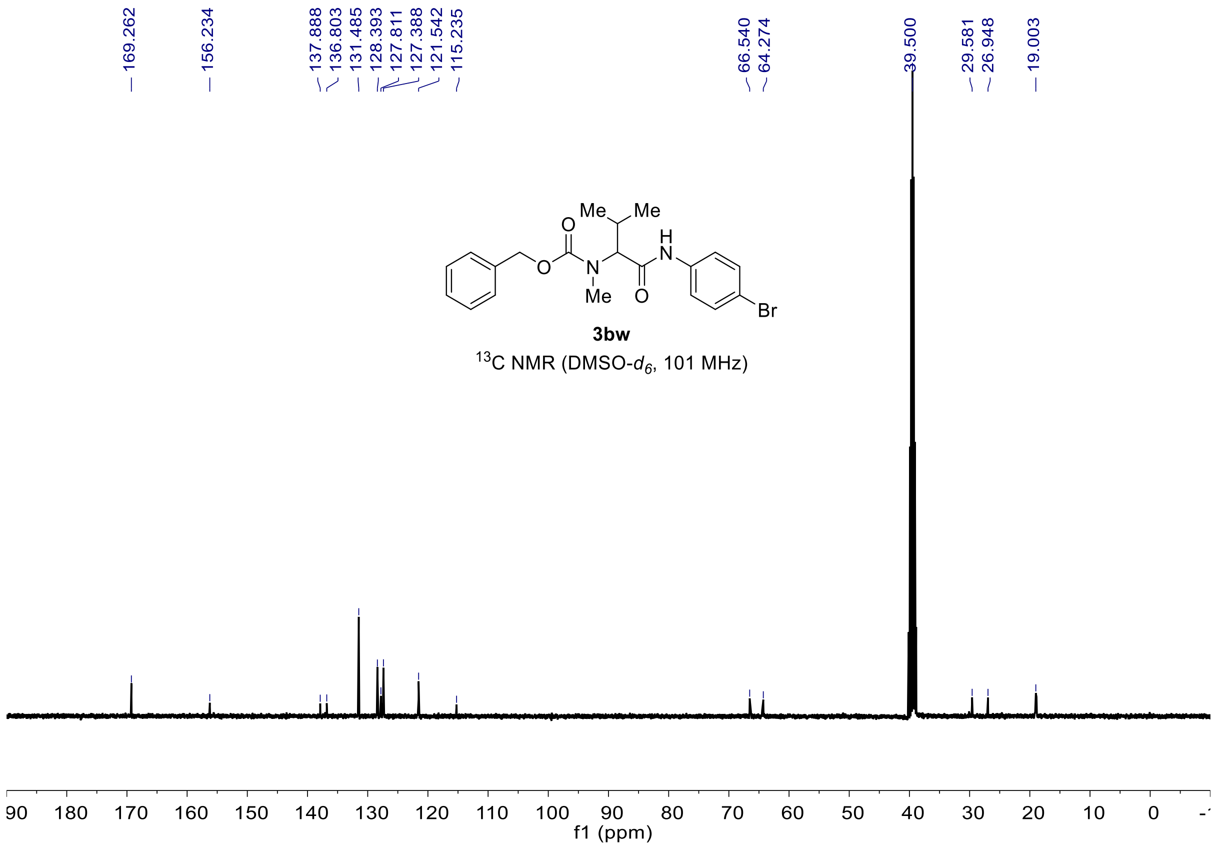

3bw

^{13}C NMR (DMSO-d_6, 101 MHz)

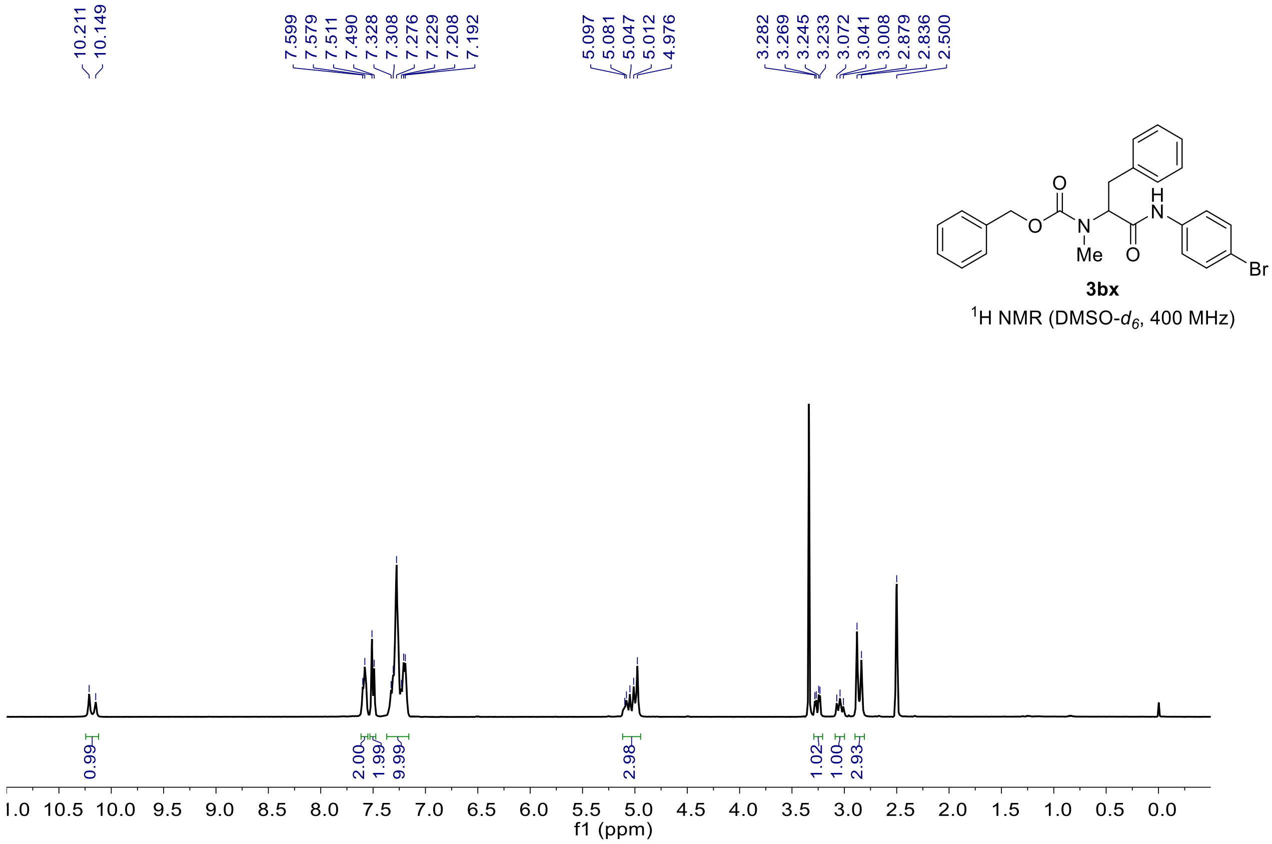

3bx

^{1}H NMR (DMSO-d_6, 400 MHz)

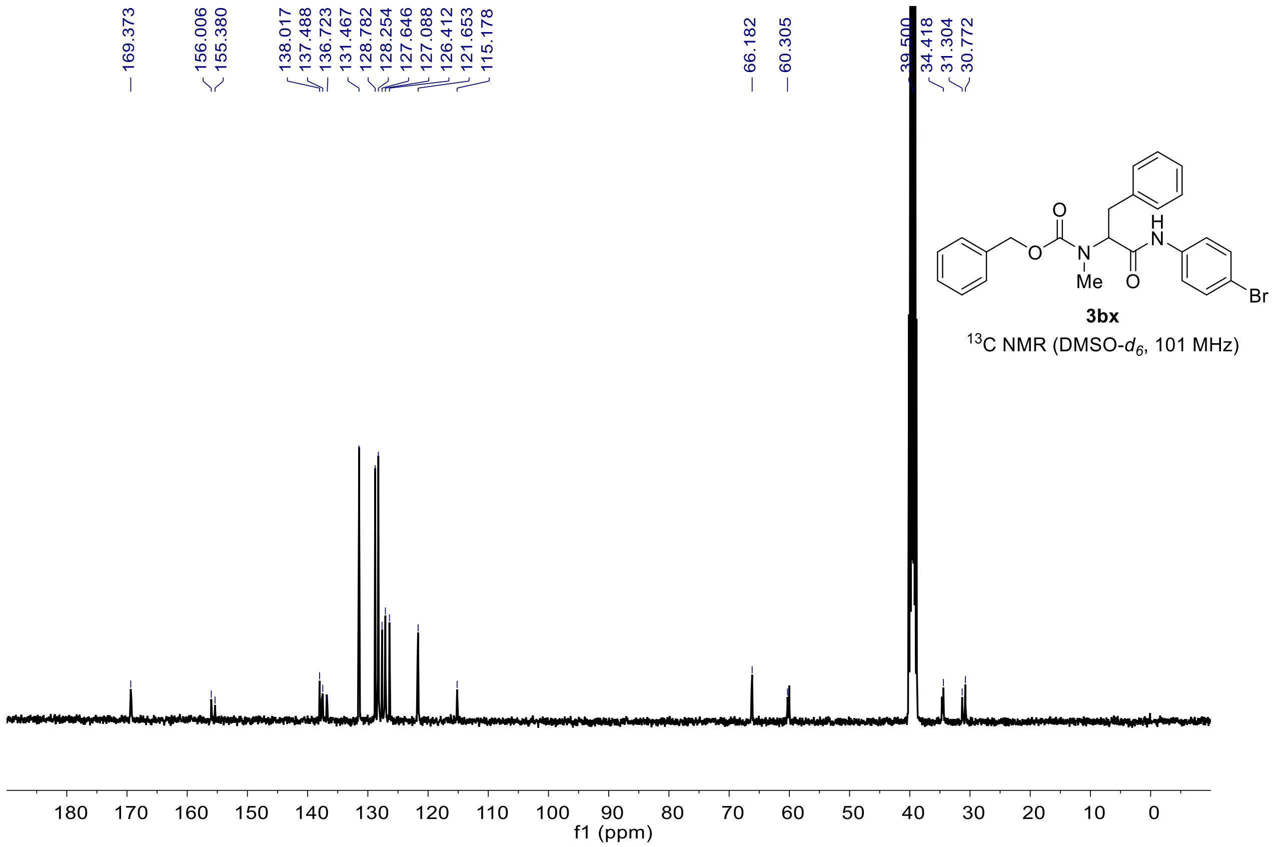

3bx

^{13}C NMR (DMSO-d_6, 101 MHz)

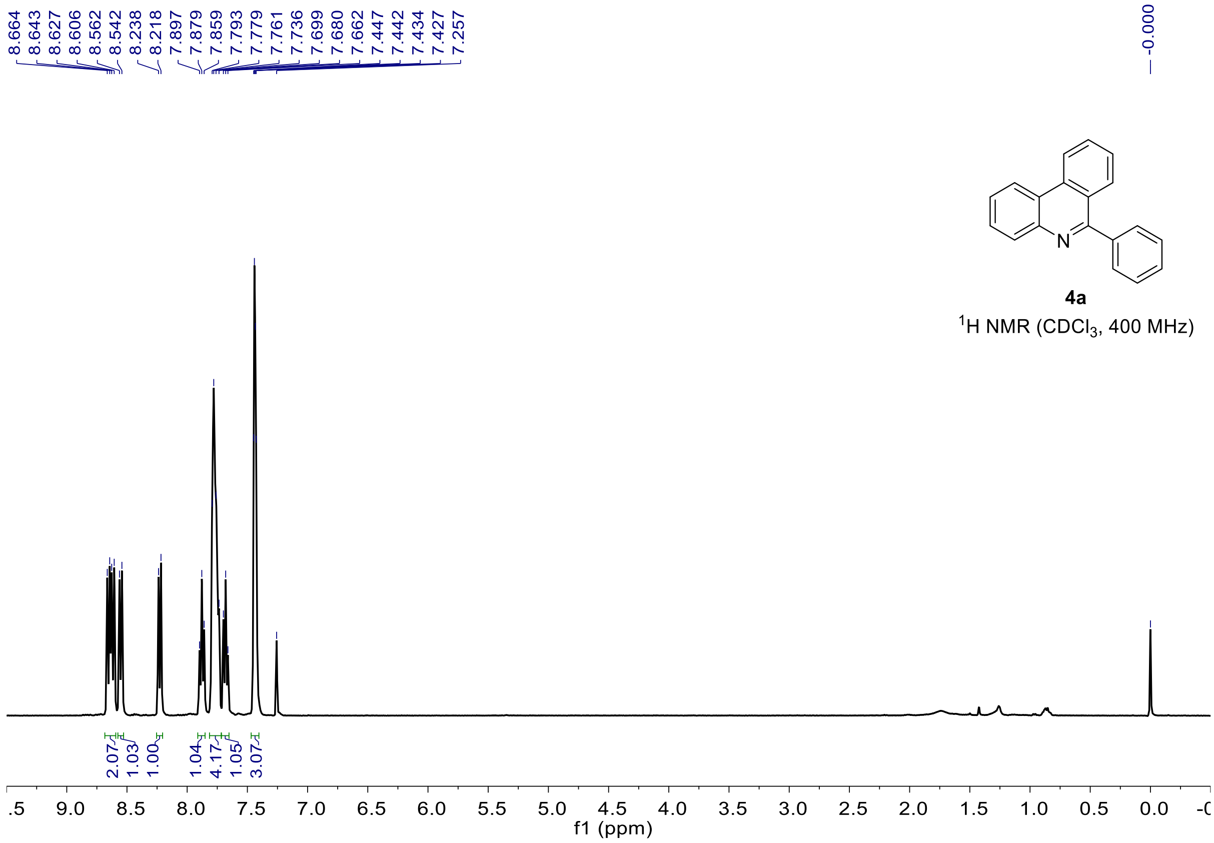

4a

^{1}H NMR ($CDCl_3$, 400 MHz)

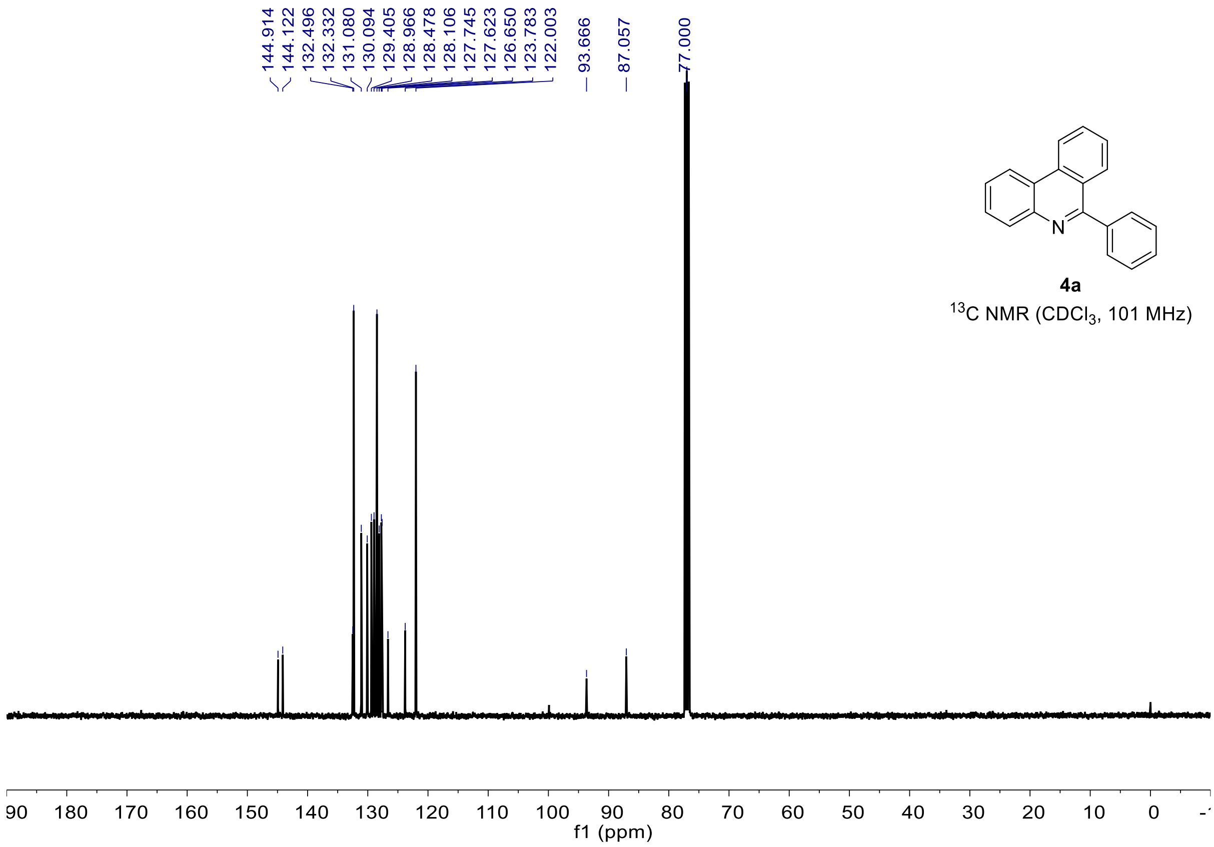

4a

^{13}C NMR ($CDCl_3$, 101 MHz)

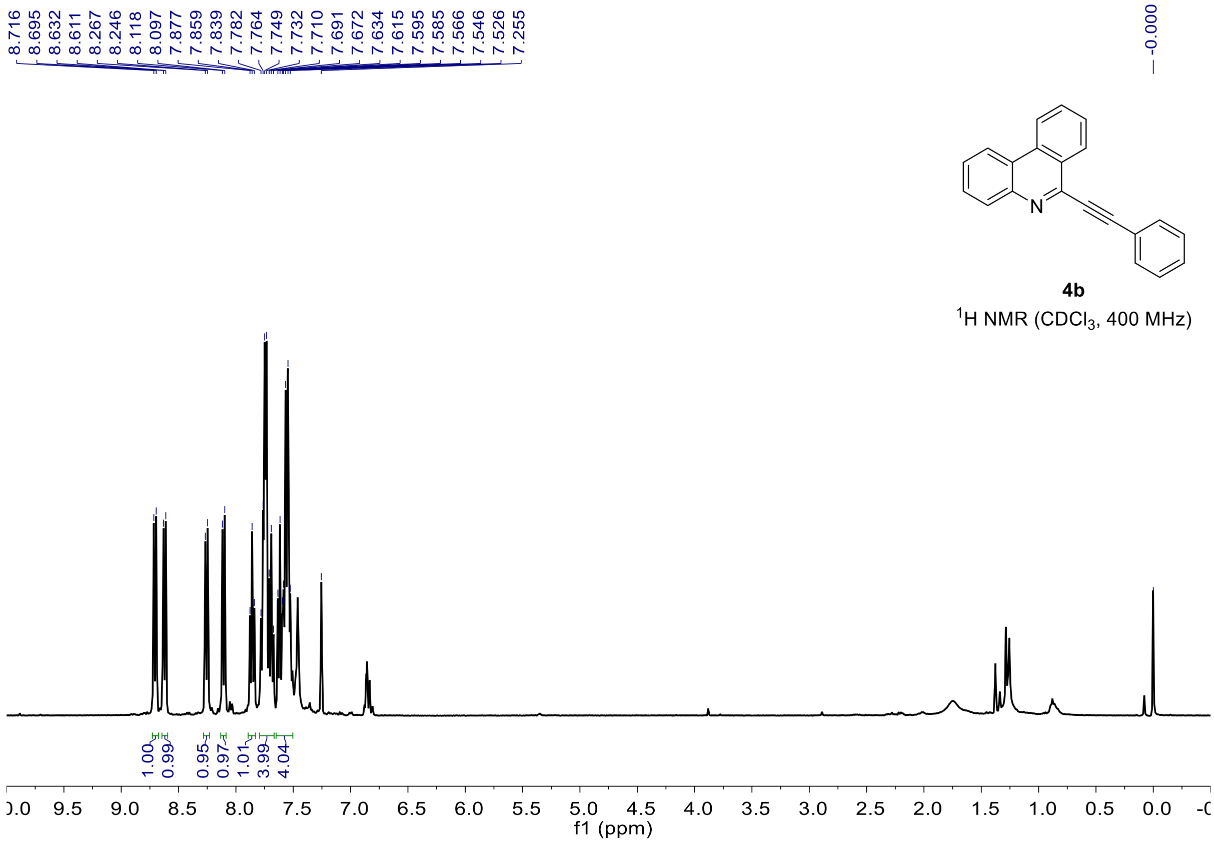

4b

^{1}H NMR ($CDCl_3$, 400 MHz)

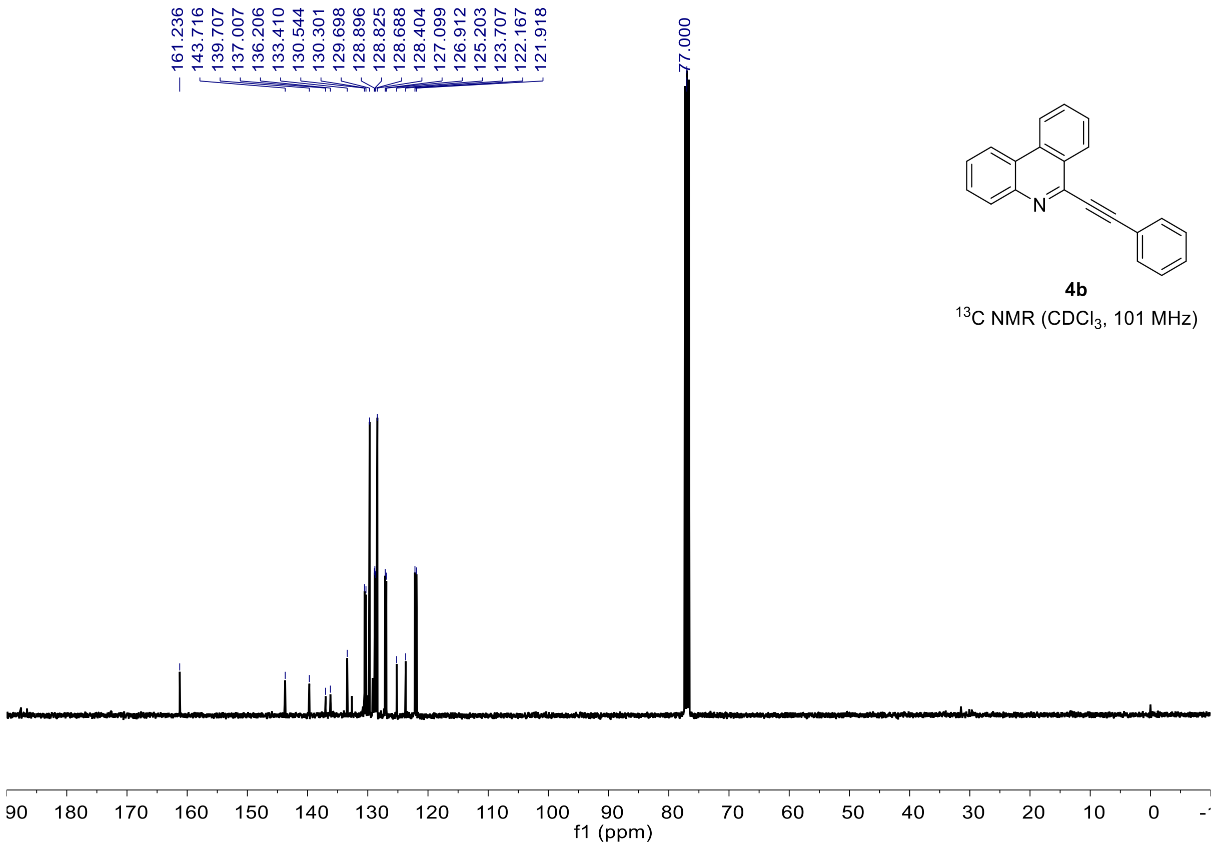

4b

^{13}C NMR ($CDCl_3$, 101 MHz)

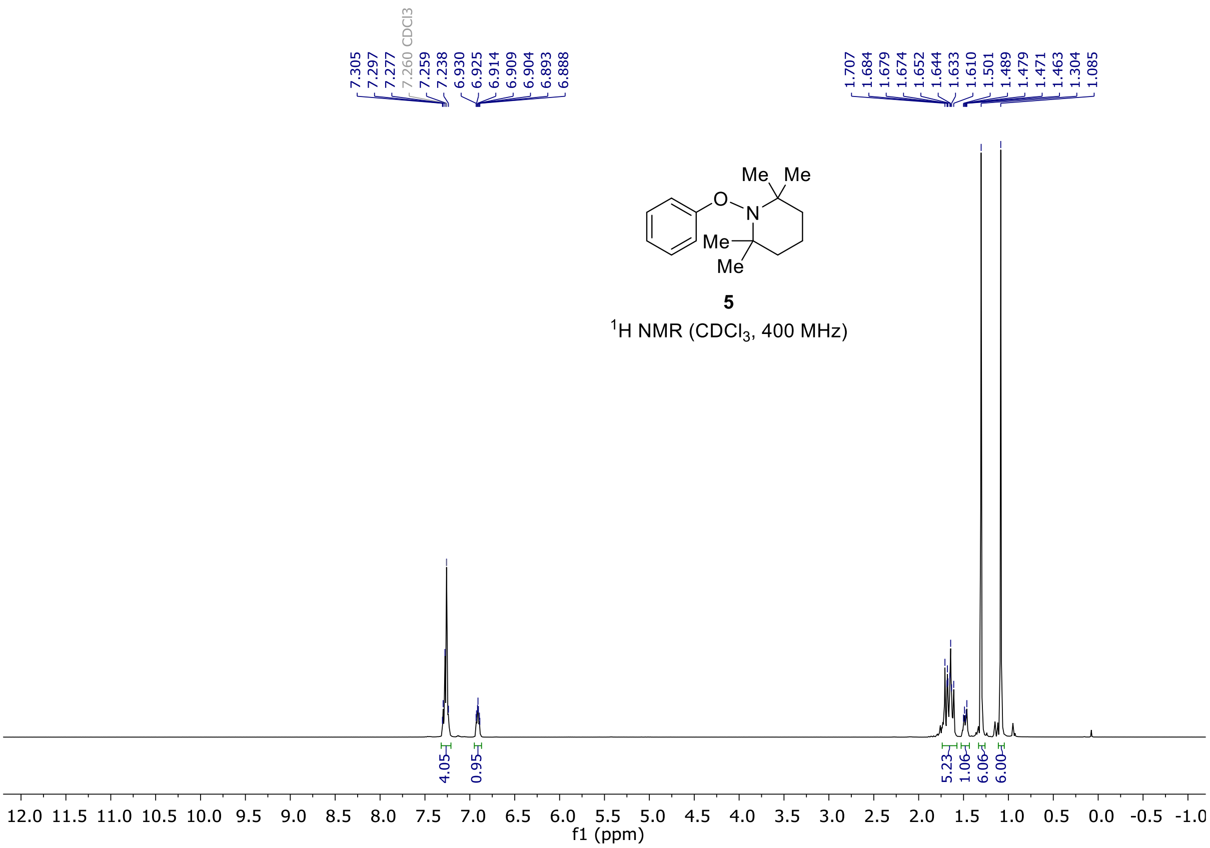

5

^{1}H NMR ($CDCl_3$, 400 MHz)

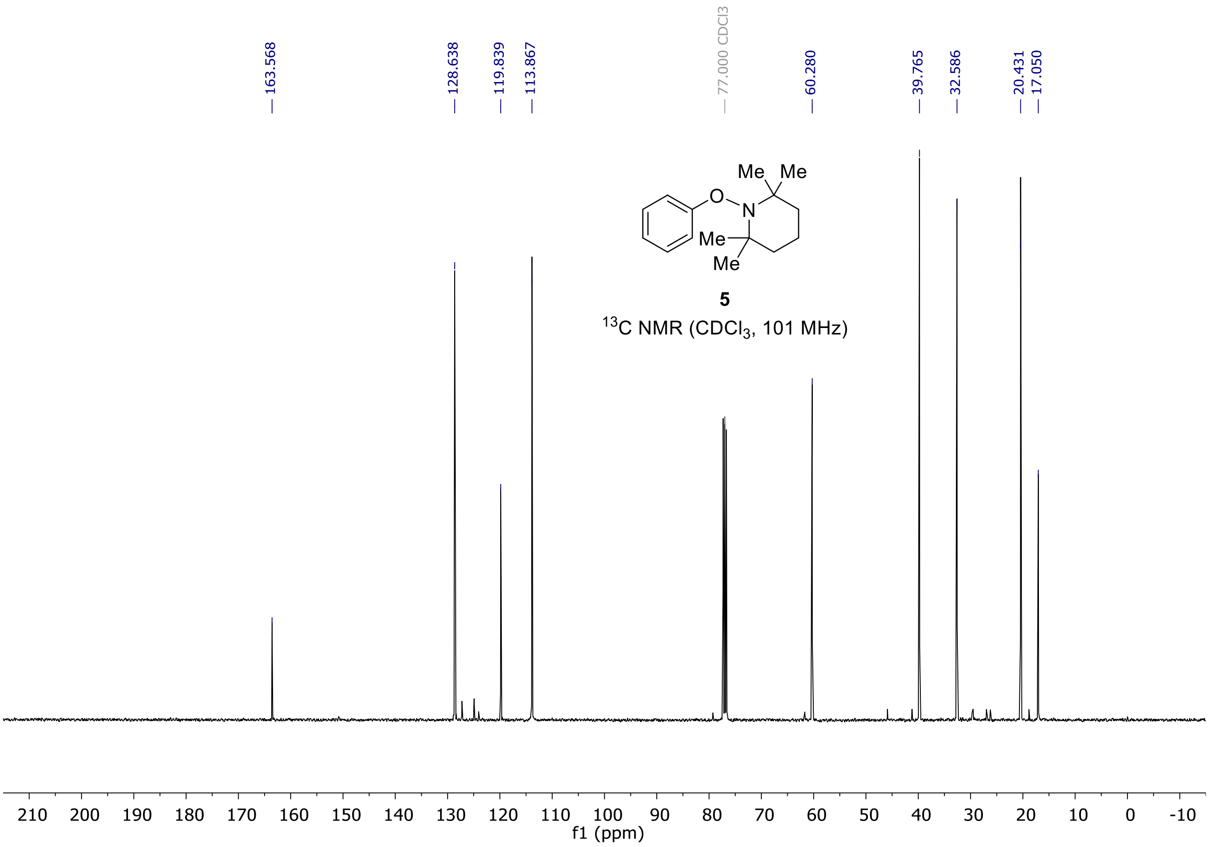

5

^{13}C NMR ($CDCl_3$, 101 MHz)

V. References

(1) Lu, L.; Qiu, F.; Alhumade, H.; Zhang, H.; Lei, A. Tuning the oxidative mono- or double-carbonylation of alkanes with CO by choosing a Co or Cu catalyst. *ACS Catal.* **2022**, *12*, 9664–9669.

(2) Ling, L.; Chen, C.; Luo, M.; Zeng, X. Chromium-catalyzed activation of acyl C–O bonds with magnesium for amidation of esters with nitroarenes. *Org. Lett.* **2019**, *21*, 1912–1916.

(3) Zhang, H.; Dai, C.; Liu, L.; Ma, H.; Bu, H.; Li, Y. Nickel(II)-catalyzed efficient aminocarbonylation of unreactive alkanes with formanilides—exploiting the deformylation behavior of imides. *Tetrahedron* **2018**, *74*, 3712–3718.

(4) Singh, H.; Sen, C.; Sahoo, T.; Ghosh, S. C. A visible light-mediated regioselective halogenation of anilides and quinolines by using a heterogeneous Cu-MnO catalyst. *Eur. J. Org. Chem*. **2018**, 4748–4753.

(5) Zheng, Y.; Dong, M.; Qu, E.; Bai, J.; Wu, X.-F. Pd-catalyzed carbonylative synthesis of 4*H*-benzo[*d*][1,3]oxazin-4-ones using nenzene-1,3,5-triyl triformate as the CO source. *Chem. Eur. J*. **2021**, *27*, 16219–16224.

(6) Yuan, H.; Liu, Z.; Shen, Y.; Zhao, H.; Li, C.; Jia, X.; Li, J. Iron-catalyzed oxidative coupling reaction of isocyanides and simple alkanes towards amide synthesis. *Adv. Synth. Catal.* **2019**, *361*, 2009–2013.

(7) Forni, J.; Micic, N.; Connell, T.; Weragoda, G.; Polyzos, A. Tandem photoredox catalysis: Enabling carbonylative amidation of aryl and alkylhalides. *Angew. Chem., Int. Ed*. **2020**, *59*, 18646–18654.

(8) Ai, H.-J.; Zhao, F.; Wu, X.-F. SET or TET? Iron-catalyzed aminocarbonylation of unactivated alkyl halides with amines, amides, and indoles via a substrate dependent mechanism. *Chin. J. Catal.* **2023**, *47*, 121–128.

(9) Despotović, V.; Kordić, B.; Kovačević, M.; Petrović, S.; Jović, B. Investigation of N–H···O interactions in N-monosubstituted caproamide–ether systems: FT–IR and FT–NIR spectroscopic study. *J. Mol. Struct*. **2019**, *1181*, 19–24.

(10) Huang, B.; Zhao, Y.; Yang, C.; Gao, Y.; Xia, W. Combining Eosin Y with Selectfluor: a regioselective brominating system for *para*-bromination of aniline derivatives. *Org. Lett*. **2017**, *19*, 3799–3802.

(11) Zhao, T.-F.; Xu, X.-L.; Sun, W.-Y.; Lu, Y. Construction of benzoxazinones from anilines and their derivatives. *Org. Lett*. **2023**, *25*, 4968–4973.

(12) Chen, L.-J.; Kuo, C.-J.; Liang, C.-F. Synthesis of aryl amides from acyl-bunte salts and aryl azides. *J. Org. Chem*. **2023**, *88*, 10501–10507.

(13) Xia, C.; Wang, K.; Wang, G.; Duan, G. Iron-catalyzed *ortho* trifluoromethylation of anilines *via* picolinamide assisted photoinduced C–H functionalization. *Org. Biomol. Chem*. **2018**, *16*, 2214–2218.

(14) Green, R.; Pletcher, D.; Leach, S.; Brown, R. *N*-Heterocyclic carbene-mediated microfluidic oxidative electrosynthesis of amides from aldehydes. *Org. Lett*. **2016**, *18*, 1198–1201.

(15) Cho, D.; Jang, D. Indium-mediated mild and facile method for the synthesis of amides. *Tetrahedron Lett*. **2004**, *45*, 2285–2287.

(16) Liu, Y.; Sun, H.; Huang, Z.; Ma, C.; Lin, A.; Yao, H.; Xu, J; Xu, S. Metal-free synthesis of *N*-(pyridine-2-yl)amides from ketones via selective oxidative cleavage of C(O)–C(alkyl) bond in water. *J. Org. Chem*. **2018**, *83*, 14307–14313.

(17) Zhang, R.; Gu, Z.-Y.; Wang, S.-Y.; Ji, S.-J. Co(II)/Ag(I) synergistically catalyzed monoinsertion reaction of isocyanide to terminal alkynes with H_2O: synthesis of alkynamide derivatives. *Org. Lett*. **2018**, *20*, 5510–5514.

(18) Lu, S.; Gong, Y.; Zhou, D. Transition metal-free oxidative radical decarboxylation/cyclization for the construction of 6-alkyl/aryl phenanthridines. *J. Org. Chem*. **2015**, *80*, 9336–9341.

(19) Shan, L.; Li, H.; Min, L.; Weng, Y.; Wang, X.; Hu, Y. Bischler–Napieralski synthesis of 6-alkynyl phenanthridines based on Tf_2O-promoted electrophilic activation of *N*-aryl-2-propynamides. *J. Org. Chem*. **2021**, *86*, 15726–15732.
