# Supplementary figures and images for: General Approach to Amides through Decarboxylative Radical Cross-Coupling of Carboxylic Acids and Isocyanides
Source: Org Lett. 2024 Apr 12;26(16):3380–5. doi: 10.1021/acs.orglett.4c00872 (PMC11059110; doi:10.1021/acs.orglett.4c00872)

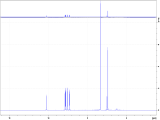

Supplement: Supplementary file 2 — ol4c00872_si_002.zip [file ol4c00872_si_002.zip › FID/3ai/3ai-1H/pdata/1/thumb.png]
